# The Hot Pepper (*Capsicum annuum*) MicroRNA Transcriptome Reveals Novel and Conserved Targets: A Foundation for Understanding MicroRNA Functional Roles in Hot Pepper

**DOI:** 10.1371/journal.pone.0064238

**Published:** 2013-05-30

**Authors:** Dong-Gyu Hwang, June Hyun Park, Jae Yun Lim, Donghyun Kim, Yourim Choi, Soyoung Kim, Gregory Reeves, Seon-In Yeom, Jeong-Soo Lee, Minkyu Park, Seungill Kim, Ik-Young Choi, Doil Choi, Chanseok Shin

**Affiliations:** 1 Department of Agricultural Biotechnology, Seoul National University, Seoul, Republic of Korea; 2 Department of Plant and Environmental Sciences, New Mexico State University, Las Cruces, New Mexico, United States of America; 3 Department of Plant Science, College of Agriculture and Life Sciences, Seoul National University, Seoul, Republic of Korea; 4 National Instrumentation Center for Environmental Management, College of Agriculture and Life Sciences, Seoul National University, Seoul, Republic of Korea; 5 Plant Genomics and Breeding Institute, Seoul National University, Seoul, Republic of Korea; Cankiri Karatekin University, Turkey

## Abstract

MicroRNAs (miRNAs) are a class of non-coding RNAs approximately 21 nt in length which play important roles in regulating gene expression in plants. Although many miRNA studies have focused on a few model plants, miRNAs and their target genes remain largely unknown in hot pepper (*Capsicum annuum*), one of the most important crops cultivated worldwide. Here, we employed high-throughput sequencing technology to identify miRNAs in pepper extensively from 10 different libraries, including leaf, stem, root, flower, and six developmental stage fruits. Based on a bioinformatics pipeline, we successfully identified 29 and 35 families of conserved and novel miRNAs, respectively. Northern blot analysis was used to validate further the expression of representative miRNAs and to analyze their tissue-specific or developmental stage-specific expression patterns. Moreover, we computationally predicted miRNA targets, many of which were experimentally confirmed using 5′ rapid amplification of cDNA ends analysis. One of the validated novel targets of miR-396 was a domain rearranged methyltransferase, the major *de novo* methylation enzyme, involved in RNA-directed DNA methylation in plants. This work provides the first reliable draft of the pepper miRNA transcriptome. It offers an expanded picture of pepper miRNAs in relation to other plants, providing a basis for understanding the functional roles of miRNAs in pepper.

## Introduction

MicroRNAs (miRNAs), initially discovered in *C. elegans*, are non-coding RNAs that can play regulatory roles in animals and plants by negatively affecting gene expression at the post-transcriptional level through mRNA cleavage or translation repression, depending on complementarity between miRNA and the target mRNA. Many studies have shown that miRNAs regulate many biological processes in plants, including developmental phase transition, metabolism, hormone signaling, and stress responses [1]–[3]. Plant miRNAs mostly direct the cleavage of target messages with full complementarity, and their target sites are primarily found in coding regions [1], [3], inducing a significantly robust effect on gene regulation. Recent studies have showed that plant miRNAs often repress translation via a slicer-independent mechanism [4], [5], and therefore plant miRNA-mediated post-transcriptional gene silencing entails a combination of slicing and translation inhibition.

Plant miRNA genes are distinct noncoding transcriptional units which are transcribed by RNA polymerase II as primary miRNAs and are subsequently cleaved into precursor miRNA (pre-miRNAs) by the RNaseIII-type enzyme DICER-LIKE1 (DCL1) to produce the precursor miRNAs (pre-miRNAs). These pre-miRNAs are further cleaved into miRNA:miRNA* duplexes, again by DCL1, in the nucleus. These miRNA duplexes are stabilized by 2′-O-methylation which is catalyzed by Hua Enhancer 1 [6] and transported from the nucleus to the cytoplasm by HASTY [7]. One of the strands is finally incorporated into the RNA-induced silencing complex (RISC), where it negatively regulates target mRNA expression.

There are two major approaches for identifying miRNAs in plants: experimental and bioinformatic approaches. Experimental approaches have included forward genetics, direct cloning, and next generation high-throughput sequencing. High-throughput sequencing technology shows significant promise for small RNA identification and has become commonly available and affordable. A large number of miRNAs have been identified by means of high-throughput sequencing and can be found on an online database (http://www.mirbase.org, release 19.0, August 2012), which currently consists of 21,643 mature miRNAs from 168 species, including 4,677 mature miRNAs from 52 plant species. The majority of miRNAs identified so far have been obtained from only a few model plant species, such as *Arabidopsis thaliana* (328), *Oryza sativa* (661), *Glycine max* (395) and *Medicago truncatula* (674). Solanaceae, whose annotated miRNAs are still very limited [8]–[10], is one of the largest families in the plant kingdom, consisting of more than 3,000 species [11]. Pepper (*Capsicum annuum*), which belongs to the *Solanaceae* family, is one of the most economically important crops cultivated worldwide, especially in South and Central America and Asia [12]. It is essential to understand the function of pepper miRNAs, considering the importance of pepper for food, health products and medicines. The study of the miRNAs in pepper has previously been reported using an *in silico* approach [13]. However, no research using high-throughput sequencing approaches has been performed on pepper so far.

In this study, we employed high-throughput sequencing technology to sequence and identify pepper miRNAs by taking advantage of an ongoing pepper sequencing project [14]. We were effectively able to identify and characterize 29 and 35 of conserved and novel miRNA families, respectively, from 10 different libraries. We also employed small RNA northern blot analysis to further validate and analyze the expression patterns of many of the representative miRNAs. Additionally, we made a detailed analysis of pepper miRNA targets, many of which were experimentally validated using 5′ RACE analysis [15]. Most targets of conserved miRNA were validated, and one of novel targets of conserved miRNAs that we first discovered was domain rearranged methyltransferase (DRM), considered to be one of the most important epigenetic marks in plants. On the other hand, we found that most of non-conserved miRNAs were weakly expressed and tend to lack experimentally validated targets. On the whole, our work serves to expand existing knowledge of pepper miRNAs and to give a better comparison between pepper miRNAs and those found in other plants, especially within Solanaceae.

## Materials and Methods

### Construction of small RNA library

A small RNA sequencing library was constructed using the Small RNA Sample Prep Kit (Illumina, CA, US), according to the manufacturer's instructions. Briefly, 5 µg of total RNA from hot pepper tissue samples (Mexican pepper landrace, Criollo de Morelos 334, CM334), including, leaf, stem, root, flower, fruit-1 (6 DAP; DAP for days after pollination), fruit-2 (16 DAP), fruit-MG (36 DAP; MG for mature green), fruit-B (38 DAP), fruit-B5 (43 DAP) and fruit-B10 (48 DAP) were ligated to a 3′ adaptor and a 5′ adaptor sequentially and then converted to cDNA by RT-PCR. The resulting cDNAs were then amplified by PCR, gel-purified and submitted for Illumina/Solexa sequencing. The GEO accession number for our series is GSE 41654.

### MicroRNA identification

A miRNA prediction pipeline was written by Python scripting language. High-quality small RNA reads were obtained from raw reads through filtering out poor quality reads and removing adaptor sequences using FAXTX toolkit [16]. These clean sequences were then queried against non-coding RNAs (rRNA, tRNA, snRNA, snoRNA) from the Rfam database (http://www.sanger.ac.uk/software/Rfam) and the tomato genome database, ITAG 2.3 Release (http://solgenomics.net/organism/Solanum_lycopersicum/genome) [17]. Any small RNA read matches to these sequences were excluded from further analysis. The reads between 18–26 nucleotide in length were selected and aligned with a draft contig sequence of an ongoing pepper genome project [14] using MicroRazerS program [18]. The contig numbers used for mapping miRNAs are available in Table S5 and S6. The perfect matched sequences were selectively chosen and mapped to the maximum of 25 loci per sequence.

To obtain the precursor sequences, potential miRNA sequences (reads≥50) were extended upstream and downstream of 100 to 500 nt with a step size of 100 nt. Each putative precursor sequence was folded using RNAfold from the Vienna RNA software package [19], and the potential miRNA* sequences were selected with a mismatch ratio of 0.3 or less. The region of these putative precursor sequences with the addition of 15 nt marginal sequences were re-folded using RNAfold [19] to check whether miRNA/miRNA* duplex was suited to primary criteria for annotation of plant miRNAs [20]. All of the small RNA reads in the selected putative precursor region were mapped to examine the strand bias whether the reads of sense strands accounted for 90% of total reads. The miRNA candidates were essentially grouped into families by mature sequence similarity and/or loci. By the similarity search with miRBase release 18.0 (http://www.mirbase.org), all members of miRNA candidate families were classified to either the known miRNAs or the novel miRNA candidates. The normalizing factors were calculated using the DESeq library [21] in the R statistical software package (R Development Core Team, 2009).

### MicroRNA target prediction

The putative target sites of miRNAs were identified by aligning mature miRNA sequences with a draft genome sequence [14] using TargetFinder, (http://carringtonlab.org/resources/targetfinder). miRNA targets were computationally predicted essentially as described [22]–[24]. Briefly, potential targets from FASTA searches were scored using a position-dependent, mispair penalty system. Penalties were assessed for mismatches, bulges, and gaps (+1 per position) and G∶U pairs (+0.5 per position). Penalties were doubled if the mismatch, bulge, gap, or G∶U pair occurred at positions 2 to 13 relative to the 5′ end of the miRNAs. Only one single-nucleotide bulge or single-nucleotide gap was allowed, and targets with penalty scores of four or less were considered to be putative miRNA targets (Table S1).

### Northern blot analysis

Total RNA was extracted from different tissues using TriReagent (Ambion). A total amount of 20 µg of RNA from leaf, stem, root, inflorescence, fruit and seedling was individually separated in a 15% UREA polyacrylamide gel, electrophoretically transferred to Hybond-NX membrane (GE healthcare), and was chemical cross-linked via 1-ethyl-3-(3-dimethylaminopropyl) carbodiimide (EDC) [25]. For labeling reaction of probes, 2 µl of 10 µM oligo, 2 µl of 10X T4 PNK buffer (Takara), 2.5 µl of [γ-^32^P] ATP, >7000Ci/mmole (∼150 µCi/µl), 12.5 µl of dH_2_O and 1 µl of T4 Poly Nucleotide Kinase (Takara) were added in a 20 µl reaction for 1 hour at 37°C. The labeled probes were further purified from unincorporated labels with PERFORMA Spin Columns (Edge Bio) according to the manufacturers' instruction. Probe sequences used for northern blot analysis were shown in Table S2. Hybridization and washing procedures were performed essentially as described [26]. The membranes were exposed to a phosphorimager, and signals were analyzed using BAS-2500 (Fuji).

### MicroRNA target validation assays

For miRNA target validation, gene-specific 5′ RNA ligase-mediated rapid amplification of cDNA ends (5′ RLM-RACE) was performed using the GeneRacer Kit (Invitrogen). Five micrograms of total RNA from mixed tissues of seedling, root and flower were ligated to 0.25 µg of the GeneRacer RNA oligo adapter (5′–CGACUGGAGCACGAGGACACUGACAUGGACUGAAGGAGUAGAAA–3′). The combination of oligo(dT) and random hexamers were then used to prime the first strand of cDNA synthesis in a reverse transcription reaction. The resulting cDNA was PCR-amplified with the GeneRacer 5′ primer (5′-CGACTGGAGCACGAGGACACTGA-3′) and each respective gene-specific primer (shown in Table S3). The PCR product was further amplified by nested PCR using the GeneRacer 5′ nested primer (5′-GGACACTGACATGGACTGAAG GAGTA-3′) and each respective gene-specific primer (shown in Table S3). The final PCR product was gel-purified and finally cloned into pGEM-T Easy vector (Promega) for sequencing.

### Quantitative Real-time PCR

For first strand synthesis, 5 µg of total RNA was reverse-transcribed with Superscript III (Invitrogen) and oligo(dT) primers. Quantitative real time PCR was performed using the LightCycler® 480 Real-Time PCR System (Roche). Each PCR reaction was carried out with following reaction mixture: 5 µl of the LightCycler® 480 SYBR Green I Master (Roche), 0.5 µM of forward and reverse primers and 1 µl cDNA in a total volume of 10 µl. PCR cycles were as follows: 1 cycle of 95°C for 5 min; 45 cycles of 95°C for 10 sec; 60°C for 10 sec and 72°C for 10 sec. Results were normalized against *18s rRNA* or *actin*. All the reactions were run in triplicate in each of three independent experiments. The data from three independent experiments were analyzed using the comparative C_t_ method [27]. Primer sequences used for qRT-PCR analysis are as follows, *Squamosa promoter-binding protein-1* (contig100837): 5′-ACTGCTTCCCTGGAGTCTCA-3′, 5′-CCCCATTGAGGACCTGAGTA-3′; *F-box family protein 1* (contig77869): 5′-CATTGTGGGTCCTGTCAGTG-3′, 5′-ACGCCAGTCCAATAGACACC-3′; *actin*: 5′-CCACCTCTTCACTCTCTGCTCT-3′, 5′-ACTAGGAAAAACAGCCCTTGGT-3′; *18s rRNA*: 5′-CTGCCAGTAGTCATATGCTTGTC, 5′-GTGTAGCGCGCGTGCGGCCC-3′.

## Results

### Small RNA analysis in pepper

To obtain endogenous small RNAs in pepper, we used high-throughput sequencing to generate small RNA sequences from leaf, stem, root, flower, fruit-1, fruit-2, fruit-MG, fruit-B, fruit-B5 and fruit-B10, which yielded a total of 542,938,815 reads (Table 1). After removing adaptor sequences and filtering out low quality tags, a total of 524,201,950 clean reads were obtained, which were 18–26 nt in length (Table 1). After further removal of tRNAs, rRNAs, and snRNAs and snoRNAs, a total of 306,372,865 reads remained.

**Table 1:** Distribution of small RNAs in pepper from 10 different libraries.

		Redundant		Non-redundant	
		Reads	Matching pepper genome	Reads	Matching pepper genome
**Leaf**	Raw reads	87,761,235			
	Adapter removed	74,310,576		14,057,223	
	rRNA/tRNA removed	38,704,582	24,558,036	11,709,178	8,086,024
	Match known miRNAs ≥1	2,274,251	2,167,895	3,905(62)	796(58)
	Match known miRNAs ≥50	2,273,930	2,167,618	3,823(41)	724(39)
	Conserved miRNAs from predicted hairpins with abundance ≥1		2,197,192		352(33)
	Conserved miRNAs from predicted hairpins with abundance ≥50		2,195,901		266(31)
	New miRNAs from predicted hairpins with abundance ≥1		261,257		541(161)
	New miRNAs from predicted hairpins with abundance ≥50		256,460		286(96)
**Stem**	Raw reads	52,765,696			
	Adapter removed	51,913,006		16,192,994	
	rRNA/tRNA removed	32,233,648	25,845,658	13,530,265	9,934,033
	Match known miRNAs ≥1	1,830,323	1,753,729	4,264(63)	805(58)
	Match known miRNAs ≥50	1,830,033	1,753,411	4,167(40)	711(37)
	Conserved miRNAs from predicted hairpins with abundance ≥1		1,749,505		366(33)
	Conserved miRNAs from predicted hairpins with abundance ≥50		1,747,201		232(31)
	New miRNAs from predicted hairpins with abundance ≥1		212,480		561(164)
	New miRNAs from predicted hairpins with abundance ≥50		207,500		305(118)
**Root**	Raw reads	50,434,157			
	Adapter removed	50,072,353		15,224,050	
	rRNA/tRNA removed	30,969,423	25,681,254	12,874,799	9,776,038
	Match known miRNAs ≥1	3,498,172	3,404,672	3,681(62)	833(57)
	Match known miRNAs ≥50	3,497,889	3,404,377	3,591(40)	751(38)
	Conserved miRNAs from predicted hairpins with abundance ≥1		3,393,030		353(33)
	Conserved miRNAs from predicted hairpins with abundance ≥50		3,390,712		225(32)
	New miRNAs from predicted hairpins with abundance ≥1		170,849		554(165)
	New miRNAs from predicted hairpins with abundance ≥50		164,805		243(106)
**Flower**	Raw reads	61,873,756			
	Adapter removed	61,476,055		16,877,140	
	rRNA/tRNA removed	40,888,746	28,328,301	15,028,664	11,552,651
	Match known miRNAs ≥1	2,316,757	2,258,587	3,135(62)	714(55)
	Match known miRNAs ≥50	2,316,430	2,258,373	3,003(39)	623(38)
	Conserved miRNAs from predicted hairpins with abundance ≥1		2,281,533		364(33)
	Conserved miRNAs from predicted hairpins with abundance ≥50		2,278,748		190(31)
	New miRNAs from predicted hairpins with abundance ≥1		120,484		587(167)
	New miRNA from predicted hairpins with abundance ≥50		114,722		281(107)
**Fruit-1**	Raw reads	62,988,553			
	Adapter removed	62,293,585		19,478,268	
	rRNA/tRNA removed	39,270,026	31,930,008	16,737,872	12,375,524
	Match known miRNAs ≥1	1,687,948	1,614,810	3,391(71)	677(57)
	Match known miRNAs ≥50	1,687,612	1,614,436	3,270(36)	546(34)
	Conserved miRNAs from predicted hairpins with abundance ≥1		1,602,497		333(33)
	Conserved miRNAs from predicted hairpins with abundance ≥50		1,599,969		157(29)
	New miRNAs from predicted hairpins with abundance ≥1		115,256		557(170)
	New miRNAs from predicted hairpins with abundance ≥50		109,329		216(89)
**Fruit-2**	Raw reads	43,032,671			
	Adapter removed	42,289,844		17,537,387	
	rRNA/tRNA removed	31,504,608	25,518,067	15,580,091	11,752,392
	Match known miRNAs ≥1	1,243,288	1,193,441	3,098(62)	659(57)
	Match known miRNAs ≥50	1,242,853	1,193,103	2,959(36)	554(35)
	Conserved miRNAs from predicted hairpins with abundance ≥1		1,184,731		348(33)
	Conserved miRNAs from predicted hairpins with abundance ≥50		1,181,695		146(29)
	New miRNAs from predicted hairpins with abundance ≥1		115,541		592(172)
	New miRNAs from predicted hairpins with abundance ≥50		109,397		284(117)
**Fruit-MG**	Raw reads	46,725,193			
	Adapter removed	46,424,679		12,849,765	
	rRNA/tRNA removed	22,000,286	17,766,991	11,296,813	8,585,158
	Match known miRNAs ≥1	730,015	701,563	2,472(59)	580(50)
	Match known miRNAs ≥50	729,629	701,281	2,328(34)	480(33)
	Conserved miRNAs from predicted hairpins with abundance ≥1		702,823		335(33)
	Conserved miRNAs from predicted hairpins with abundance ≥50		699,794		118(26)
	New miRNAs from predicted hairpins with abundance ≥1		91,716		566(167)
	New miRNAs from predicted hairpins with abundance ≥50		85,099		224(87)
**Fruit-B**	Raw reads	47,761,557			
	Adapter removed	46,847,384		15,359,015	
	rRNA/tRNA removed	26,156,391	21,046,683	13,820,419	10,470,918
	Match known miRNAs ≥1	569,133	545,286	2,343(59)	596(55)
	Match known miRNAs ≥50	568,863	545,024	2,237(36)	501(34)
	Conserved miRNAs from predicted hairpins with abundance ≥1		547,838		340(33)
	Conserved miRNAs from predicted hairpins with abundance ≥50		544,977		123(29)
	New miRNAs from predicted hairpins with abundance ≥1		102,788		569(171)
	New miRNAs from predicted hairpins with abundance ≥50		96,081		235(90)
**Fruit-B5**	Raw reads	38,168,067			
	Adapter removed	37,640,821		11,019,173	
	rRNA/tRNA removed	19,591,941	15,809,606	10,046,174	7,616,358
	Match known miRNAs ≥1	742,857	714,548	2,422(58)	566(51)
	Match known miRNAs ≥50	742,549	714,256	2,330(33)	479(31)
	Conserved miRNAs from predicted hairpins with abundance ≥1		731,273		325(33)
	Conserved miRNAs from predicted hairpins with abundance ≥50		728,288		111(26)
	New miRNAs from predicted hairpins with abundance ≥1		83,461		561(167)
	New miRNAs from predicted hairpins with abundance ≥50		76,608		203(86)
**Fruit-B10**	Raw reads	51,427,930			
	Adapter removed	50,933,647		13,459,462	
	rRNA/tRNA removed	25,053,214	20,260,032	12,366,889	9,400,255
	Match known miRNAs ≥1	897,174	863,929	2,716(62)	627(58)
	Match known miRNAs ≥50	896,827	863,631	2,589(34)	522(32)
	Conserved miRNAs from predicted hairpins with abundance ≥1		885,773		328(33)
	Conserved miRNAs from predicted hairpins with abundance ≥50		883,130		136(27)
	New miRNAs from predicted hairpins with abundance ≥1		107,247		566(170)
	New miRNAs from predicted hairpins with abundance ≥50		102,035		268(101)
**Total**	Raw reads	542,938,815			
	Adapter removed	524,201,950			
	rRNA/tRNA removed	306,372,865			
	Conserved miRNAs from predicted hairpins with abundance ≥50		15,250,415		
	New miRNAs from predicted hairpins with abundance ≥50		1,322,036		

The number in parenthesis denotes the unique sequence.

**Figure 1:**
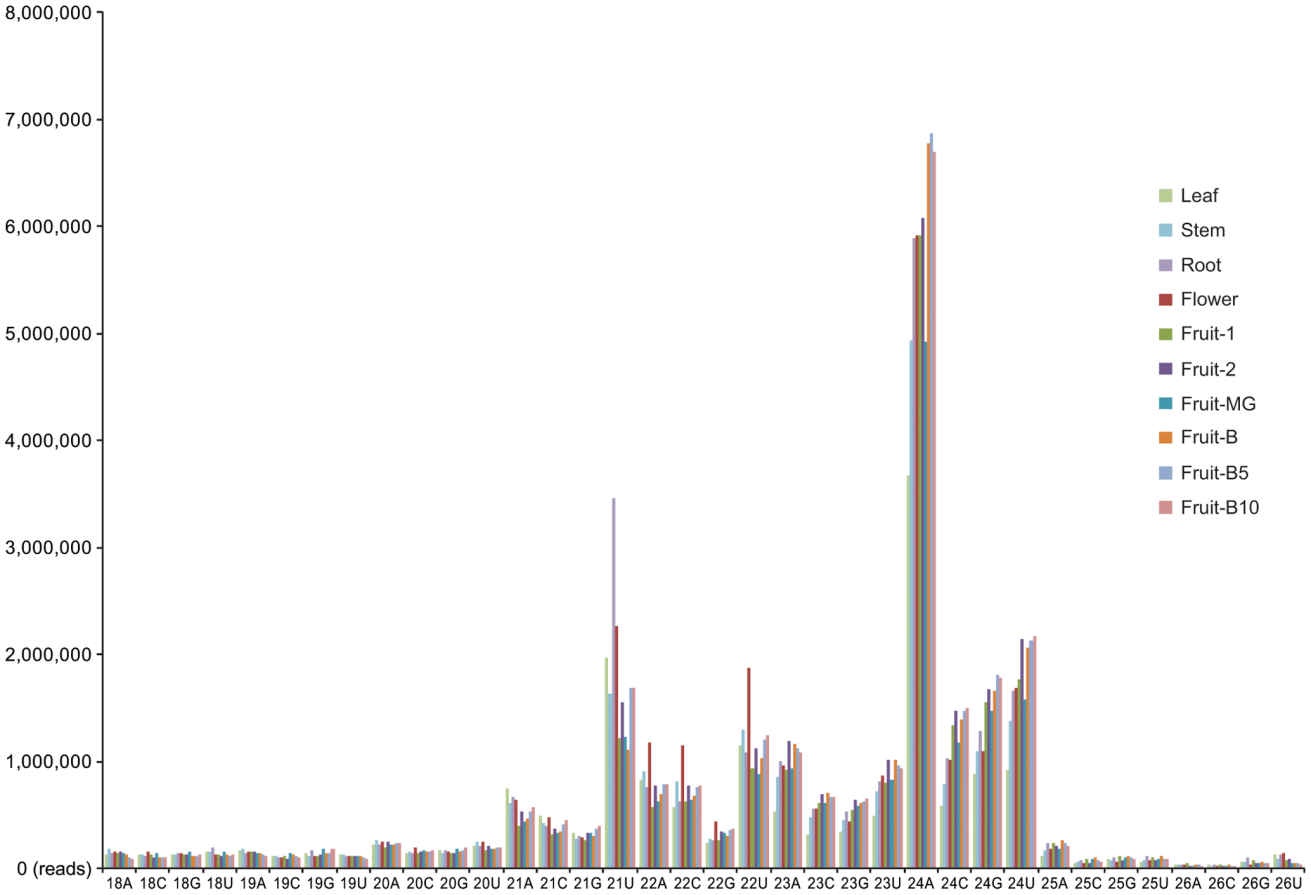
Length distribution and 5′ end analysis of small RNAs in pepper. The read numbers (y-axis) and length & 5′ terminal nucleotides (x-axis) are shown for each of the 10 different libraries, which includes leaf, stem, root, flower, fruit-1 (6 DAP), fruit-2 (16 DAP), fruit-MG (36 DAP), fruit-B (38 DAP), fruit-B5 (43 DAP) and fruit-B10 (48 DAP) in pepper, DAP for days after pollination, MG for mature green. The normalizing factors were calculated using the DESeq library in the R statistical software package.

These small RNA sequences were mapped to draft pepper genome sequences to determine whether they could be considered candidate miRNAs. These candidate miRNAs were further selected based on strict criteria for annotation of plant miRNA [20], including the presence of miRNA* sequences. Consequently, conserved and novel miRNAs were identified and represented in Tables 2 and 3, respectively. Total reads of conserved miRNAs were 15,250,415 whereas those of novel miRNAs were 1,322,036 (Table 1), suggesting that novel miRNAs are weakly expressed in general. More detailed information regarding the statistics of the small RNA sequences from 10 different libraries is shown in Table 1. Furthermore, the length distribution and 5′ end analysis were conducted for the genome-aligned small RNAs (Figure 1). The majority of small RNAs were 24 nt long and accounted for 46.91% of all small RNAs, followed by 21 nt (14.16%), 22 nt (13.99%) and 23 nt (13.65%). Most of these small RNAs had a 5′ terminal U or A, which are indicative of canonical small RNAs [28].

**Table 2:** Conserved miRNAs in pepper.

Normalized read counts of miRNA sequences from 10 different libraries													
Conserved miRNA	Sequence (5′→3′)	Leaf	Stem	Root	Flower	Fruit-1	Fruit-2	Fruit-MG	Fruit-B	Fruit-B5	Fruit-B10	Length	Hp#
can-miR156a-c	UUGACAGAAGAUAGAGAGCAC	698	2407	7589	2815	32	225	1210	2715	6494	23305	21	3
can-miR156d-g	UGACAGAAGAGAGUGAGCAC	10	94	1166	32	4	20	52	53	91	152	20	4
can-miR156h	UUGACAGAAGAGAGUGAGCAU	84	176	72	74	53	89	40	52	65	105	21	1
can-miR159a-c	UUUGGAUUGAAGGGAGCUCUA	117204	61460	54057	64159	38667	29312	29700	56716	76148	101967	21	3
can-miR160	UGCCUGGCUCCCUGUAUGCCA	2724	4284	938	856	365	517	741	371	783	894	21	1
can-miR162a-e	UCGAUAAACCUCUGCAUCCAG	1484	4525	10282	3839	2061	4702	2101	2360	2832	2573	21	5
can-miR162f	UCGAUAAACCUCUGCAUCCGG	671	1368	4541	3319	3433	5796	3231	2933	3119	1747	21	1
can-miR164a-b	UGGAGAAGCAGGGCACGUGCA	95	381	118	293	699	458	3642	2902	18082	16181	21	2
can-miR164c	UGGAGAAGCAGGGCGCGUGCA	114	169	4	4	4	4	11	2	11	8	21	1
can-miR166a-h	UCGGACCAGGCUUCAUUCCCC	268932	307399	211712	468628	450083	588387	335556	220638	423083	315156	21	8
can-miR166i-j	UCUCGGACCAGGCUUCAUUCC	286713	355950	2481041	916101	245209	233061	244844	196621	333460	296253	21	2
can-miR166k	UCGAACCAGGCUUCAUUCCCC	133	304	164	268.8	360	469	276	217	345	271	21	1
can-miR166l	UCGGACCAGGCUUCAUUCCUC	109232	42342	15520	34410	38463	12901	12758	9383	17920	23174	21	1
can-miR167a-c	UGAAGCUGCCAGCAUGAUCUA	1921	249	548	1845	1940	3059	1819	1455	1666	1849	21	3
can-miR167d	UGAAGCUGCCAGCAUGAUCUGG	6379	249	2550	185	17	92	262	235	460	790	22	1
can-miR168a-b	UCGCUUGGUGCAGGUCGGGAC	3495	6790	8629	2794	1529	5060	2596	2144	3729	4012	21	2
can-miR168c	UCGCUUGGUGCAGGUCGGGAA	2998	2496	2447	955	720	1150	425	297	460	389	21	1
can-miR169a-g	UAGCCAAGGAUGACUUGCCU	89	765	3070	58	8	38	23	36	13	3	20	7
can-miR169h	UAUCGGCAGGUCAUCCUUGGC	7	17	56	14	4	2	2	0	2	1	21	1
can-miR171a-e	UGAUUGAGCCGUGCCAAUAUC	781	724	454	462	703	380	190	255	244	251	21	5
can-miR171f-g	UGAUUGAGCCGUGUCAAUAUC	179	73	3	83	20	9	12	24	10	10	21	2
can-miR171h	UGAGCCGAACCAAUAUCACUC	183	446	4003	813	994	404	172	104	85	86	21	1
can-miR171i	UUGAGCCGCGCCAAUAUCACU	79	263	243	8	30	27	11	8	8	5	21	1
can-miR171j	UUGAGCCGCGCCAAUAUCAUU	8	61	253	3	8	4	4	4	2	3	21	1
can-miR171k	UUGAGCCGUGCCAAUAUCACGU	337	124	104	47	55	15	11	46	80	81	22	1
can-miR171l	UUGAGCCGCGCCAAUAUCACG	1475	293	56	358	244	226	817	619	782	806	21	1
can-miR172a-d	AGAAUCUUGAUGAUGCUGCAU	472	324	53	210	52	81	110	161	206	155	21	4
can-miR172e	UGAAUCUUGAUGAUGCUGCAU	65	67	43	47	13	42	8	13	26	37	21	1
can-miR172f	AGAAUCUUGAUGAUGCUGCAG	1	4	2	285	20	12	14	62	241	475	21	1
can-miR172g	GGAAUCUUGAUGAUGCUGC	9	44	1	260	44	48	181	190	426	468	19	1
can-miR172h	AGAAUCUUGAUGCUGCUGCAU	3199	336	2	139	72	23	4	0	6	0	21	1
can-miR172i	GGAAUCUUGAUGAUGCUGCAG	7	85	5	545	56	174	383	583	1112	1569	21	1
can-miR319a	CUUGGACUGAAGGGAGCUCCC	588	64598	13611	41664	1719	730	2920	2704	3384	6198	21	1
can-miR319b-c	UUGGACUGAAGGGAGCUCCCU	178	143377	16249	5076	711	776	2872	2502	3285	3443	21	2
can-miR319d	UCUUGGACUGAAGGGUUCCCU	7	135	134	6142	14	26	122	241	616	1017	21	1
can-miR319e	UUGGACUGAAGGGAGCUCCC	669	50859	20520	25530	3858	1126	4486	4004	6198	7384	20	1
can-miR319f	UUGGACUGAAGGGAGCUCCUU	341	5936	2364	2375	500	448	816	916	1120	1228	21	1
can-miR319g	UUGGACUGAAGGGAGCUCCU	181	2819	783	1183	252	203	339	431	349	478	20	1
can-miR390a-b	AAGCUCAGGAGGGAUAGCGCC	258	471	1712	784	81	19	35	30	35	44	21	2
can-miR390c	AAGCUCAGGAGGGAUAGCACC	605	971	1241	125	42	364	64	75	93	162	21	1
can-miR393a-b	UCCAAAGGGAUCGCAUUGAUCC	3005	354	125	87	50	135	90	77	129	106	22	2
can-miR394a	UUGGCAUUCUGUCCACCUCC	1083	1717	406	581	184	71	107	114	99	122	20	1
can-miR394b	UUGGCAUUCUGUCUACCUCC	1481	930	78	542	47	151	290	179	132	111	20	1
can-miR395a-j	CUGAAGUGUUUGGGGGAACUC	124	54	117	150	10	28	39	74	45	39	21	10
can-miR396a	UUCCACAGCUUUCUUGAACUG	101458	18914	39283	8223	4475	20644	14973	12070	12491	10479	21	1
can-miR396b	UUCCACAGCUUUCUUGAACUU	16864	8481	7252	3095	1994	12496	1556	1324	2224	3435	21	1
can-miR396c	UUCCACAGCUUUCUUGAACUA	6618	3489	1041	180	147	863	145	138	134	141	21	1
can-miR397a	UCAUCUACGCUGCACUCAAUC	640	9	8	29	10	10	9	2	6	6	21	1
can-miR397b	AUUGAGUGCAGCGUUGAUGAC	57518	1047	2264	1621	757	1449	891	488	566	519	21	1
can-miR398a-b	UGUGUUCUCAGGUCACCCCUU	10	84	22	23	6	3	12	5	10	18	21	2
can-miR398c	UAUGUUCUCAGGUCACCCCUA	857	142	239	402	27	69	75	84	136	170	21	1
can-miR398d	UGUGUUCUCAGGUCGCCCCUG	67733	6350	4468	23607	30511	51585	19279	8530	7322	3941	21	1
can-miR399a-e	UGCCAAAGGAGAAUUGCCCUG	62	0	21	5	10	18	21	36	22	19	21	5
can-miR399f-g	UGCCAAAGGAGAGUUGCCCUG	423	20	91	131	278	188	258	465	303	326	21	2
can-miR399h	CGCCAAAGGAGAGCUGCCCUA	18	0	1	6	11	8	12	44	22	13	21	1
can-miR403	UUAGAUUCACGCACAAACUCG	8057	11621	19143	14241	8650	7548	6154	5391	6186	6813	21	1
can-miR408a	UGCACUGCCUCUUCCCUGGCU	76418	4108	2605	5224	4684	4980	3144	1334	1704	1715	21	1
can-miR408b	UGCACAGCCUCUUCCCUGGCU	17105	141	14	990	1026	1386	203	99	77	91	21	1
can-miR477	CCUCUCCCUCAAGGGCUUCUU	4	234	8115	6	0	101	16	63	19	11	21	1
can-miR482a	UUGCCGAUUCCGUCCAUACCGC	8254	4153	4415	1718	2583	2457	2387	1508	3290	3251	22	1
can-miR482b	UUUCCAAUUCCACCCAUUCCUA	2112	4529	1615	1543	3907	3278	1151	972	1147	1327	22	1
can-miR482c	UCUUGCCUACACCGCCCAUGCC	17638	23433	36831	7553	9871	17531	5334	4450	6747	8394	22	1
can-miR482d	UCUUACCGAUACCUCCCAUUCC	2016	3022	3637	1146	1413	752	500	452	815	879	22	1
can-miR482e	CUACCAACUCCACCCAUUCCUG	7	4	53	0	1	0	46	43	420	1660	22	1
can-miR482f	UCUUUCCUACUCCUCCCAUACC	166081	118677	105171	216948	173152	66770	46358	40194	66479	66781	22	1
can-miR482g	UUUCCUAUUCCACCCAUGCCAA	943	1235	1612	906	868	742	434	386	716	1013	22	1
can-miR530	UGCAUUUGCACCUGCACCUGU	12407	242	709	80	42	14	21	8	13	9	21	1
can-miR827	UUAGAUGAACAUCAACAAACA	4936	940	44	297	79	202	160	234	65	124	21	1
can-miR1446a-b	UGAACUCUCUCCCUCAAUGGCU	329	10	1195	35	3	2	6	6	5	28	22	2
can-miR4376	ACGCAGGAGAGAUGAUGCUGGA	2029	2249	301	90	168	113	49	36	42	21	22	1
can-miR4414a	AGCUGAUGACUCGUUGAUUCU	0	8	0	188	6	3	0	1	0	0	21	1
can-miR4414b	AGCUGCUGAAUCAUUGGUUCG	2	84	33	1716	10	5	49	95	81	40	21	1

**MG** stands for ‘mature green’. **Hp#(Hairpin#)** indicates the number of hairpin locus. The contig numbers used for mapping miRNAs are available in Table S5.

**Table 3:** Novel miRNAs in pepper.

Normalized read counts of miRNA sequences from 10 different libraries													
Novel miRNA	Sequence (5′→3′)	Leaf	Stem	Root	Flower	Fruit-1	Fruit-2	Fruit-MG	Fruit-B	Fruit-B5	Fruit-B10	Length	Hp#
can-miR-n001	UUUCUGUUUUGAUAGUAGGCCU	0	1025	976	1284	244	564	435	699	780	660	22	1
can-miR-n002a-c	UUGCAAACACACCUGAAUCGU	70358	30781	34831	323	55	239	309	298	405	660	21	3
can-miR-n003a	UGUAGUUGUAGCCAUUCUAUU	1379	1313	2138	2418	242	452	104	118	101	94	21	1
can-miR-n003b-d	UAGAGUGGCCACAACUAGAUG	1456	1132	1494	1256	860	619	214	171	201	142	21	3
can-miR-n004	UAAGAUCGAGCACAAGUUGUU	2798	2787	3656	3161	2170	2828	2507	2575	3389	3656	21	1
can-miR-n005	UUCGAUACGCACCUGAAUCGCC	76	9330	24545	268	5	76	218	112	145	120	22	1
can-miR-n006	CAACAAUCAUCCUUUGGGCUUU	57	308	223	856	655	15403	17379	19725	17193	12432	22	1
can-miR-n007	UUGAACCUUCAGGUGAGUUGC	640	896	2212	405	199	352	294	304	448	495	21	1
can-miR-n008	CGGCAUGAGAGAAAAUAUUGAGAA	0	0	4	4	0	420	953	1589	1433	4056	24	1
can-miR-n009a,b-5p	CCUGAACUGAACAAUACGAUC	7529	1197	1233	1059	1626	3154	1023	1197	452	557	21	2
can-miR-n009a,b-3p	UGGUAUUGUUCCGUUCAGGGA	2816	1287	780	575	391	1187	1250	1071	1302	842	21	2
can-miR-n010	UUAUGAGAUAAGUUCAACACG	1777	1179	1361	642	179	583	274	356	364	378	21	1
can-miR-n011	AGCAUCGAUUACAAUGACAAAAAG	29	76	79	170	74	410	431	698	1404	1284	24	1
can-miR-n012	UUCGGCUCAUCGUUGUUGCAGACG	300	775	492	400	683	874	849	794	970	533	24	1
can-miR-n013	UUUUAGCAAGAGUUGUUUUCC	671	578	912	618	696	357	145	225	199	136	21	1
can-miR-n014	CUGAAGUUCGUUACUGUUGUC	238	294	306	61	35	458	933	1622	945	744	21	1
can-miR-n015	UUCAGGCCUGAGAAACGAAAAACU	2397	11777	7357	12552	4329	4502	6435	9528	13191	11762	24	1
can-miR-n016a-b	AUCCAAUACAUCGUCCACAGC	8946	3982	958	1942	1952	1393	488	304	472	459	21	2
can-miR-n017	CUAAGCAACGCUAUGUCGAGU	13469	12610	5845	7831	7249	7142	4029	3330	3874	3730	21	1
can-miR-n018	AAUUGGACUGGCGCACAUCGGGAG	414	1597	1071	667	3192	2254	1715	1311	1923	2085	24	1
can-miR-n019a-d	UAAUAACUAGUAGUUGAGUGAU	0	3	1	1604	1	3	7	5	16	25	22	4
can-miR-n020	GGGGAUGUAGCUCAGAUGGUAGA	18719	7224	4696	1702	6942	1877	1842	3305	5541	3730	23	1
can-miR-n021	AUGGAUGAACAAUGCUGAAACAUU	173	1288	453	374	296	477	238	339	187	182	24	1
can-miR-n022a-c	UGAACAAGUAGACACAUGCGUCCU	307	237	217	252	199	228	240	280	479	2654	24	3
can-miR-n023	AUGACUUACUUUGACUUGGCACA	824	1424	611	808	819	752	915	999	935	824	23	1
can-miR-n024	UCAACUGCAAACCUGUAAGCCU	2392	5907	3492	11515	5334	5767	8755	9367	9857	9612	22	1
can-miR-n025	AGGAAGGAACUCCACGUCAUUGCU	57	12	12	124	331	3906	424	501	147	101	24	1
can-miR-n026	UGUCACAAUGAACUCCAUCCCA	11593	7823	4814	2852	1659	3427	2623	2672	3802	3961	22	1
can-miR-n027	AUGAAAGUUGUCGAUGCCAAC	6596	6076	10157	2132	2774	3073	1358	1096	1434	1273	21	1
can-miR-n028a-b	UCAUCCGAGAUCGUUUCGCUGA	1273	5468	1195	5568	4510	1967	3181	3131	3836	2915	22	2
can-miR-n029	UUAGAGUGAGCUCAACAGAGU	49	18	27	9	4	104	741	1605	1682	3318	21	1
can-miR-n030	UUCGAGGACCGUCAGUAGCAUA	1512	1	0	3	1	0	0	0	0	1	22	1
can-miR-n031	UUCCCAGUCCAGGCAUUCCAAC	2140	909	1063	1128	811	882	682	624	1000	1142	22	1
can-miR-n032	UGUUCCUGUAGAUAAGCCACU	524	319	374	360	213	733	1286	2260	2377	2543	21	1
can-miR-n033	UAGAGAAAGCAUGGCUUCAGGU	5101	7725	6752	4808	2785	2836	2713	3261	5975	5194	22	1
can-miR-n034	ACGGAUGAACUCGCAGAAGGACGA	59	8	372	125	100	928	1574	981	662	656	24	1
can-miR-n035	CUUCGAACUAACUCCUUCUUGACA	0	24	23	9	15	1049	812	1495	927	824	24	1

**MG** stands for ‘mature green’. **Hp#(Hairpin#)** indicates the number of hairpin locus. The contig numbers used for mapping miRNAs are available in Table S6.

### Identification of conserved miRNAs in pepper

**Figure 2:**
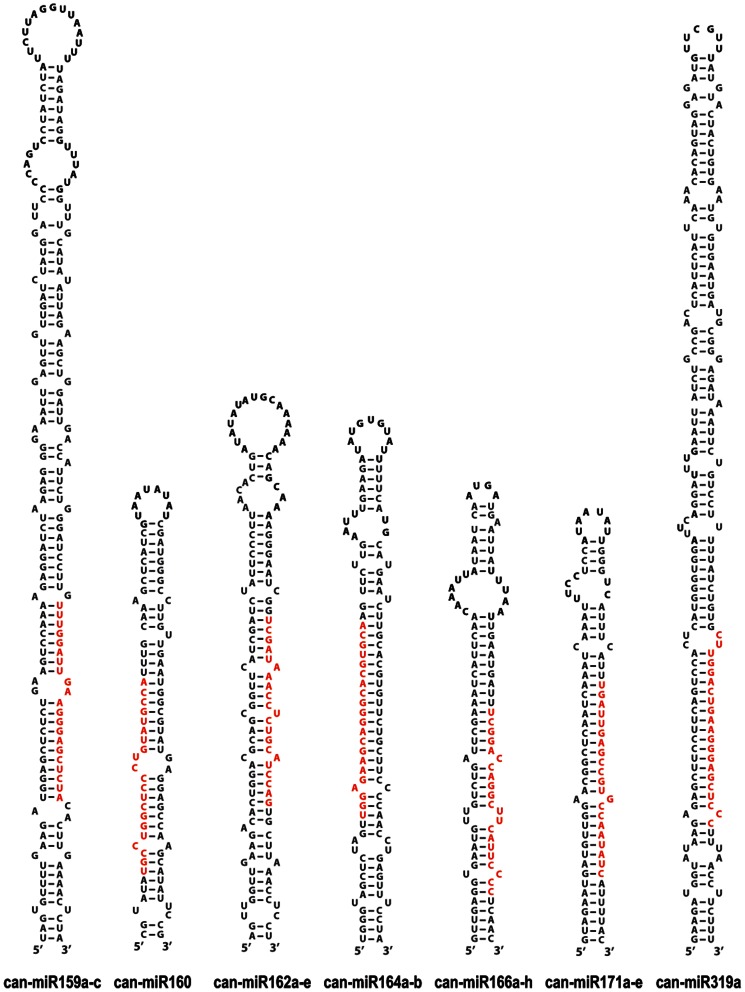
Representative hairpin precursors of conserved miRNA families. Some examples of hairpin precursors of conserved miRNAs are shown. Mature miRNAs are colored in red.

With the purpose of identifying conserved miRNAs in pepper, genome-aligned small RNA sequences were BLASTN searched against currently known miRNAs in the miRbase (Release 18), allowing one or two mismatches between sequences. The BLASTN searches identified 128 conserved miRNAs corresponding to 29 miRNA families (Table 2). Examples of representative hairpin structures are shown in Figure 2 and the full list of secondary structures is provided in Dataset S1.

The sequencing frequencies of miRNAs from ten different libraries might be used to estimate the tissue- or developmental stage-dependent expression patterns and their possible roles. As a result, Illumina/Solexa sequencing revealed that the majority of conserved miRNAs showed tissue-specific or developmental stage-specific expression. For instance, can-miR156d-g had the highest expression in root, and had lower expression in leaf, stem, flower and fruit. can-miR164a-b was expressed more abundantly in red fruits (fruit-B, B5 and B10) than in green fruits (fruit-1, 2 and MG) and other tissues. can-miR156a-c especially showed clear developmental stage-dependent expression patterns; the expression level gradually increased from fruit-1 (early stage of green fruit) to fruit-B10 (late stage of red fruit). The expression level of the can-miR168 family was higher in leaf and lower in other tissues. can-miR390 family had low expression in both green and red fruits, compared to other tissues. However, the expression level of a few miRNAs were similarly high (e.g., can-miR159 and can-miR166) or low (e.g., can-miR171f-g and can-miR172e) in all tissues.

### Identification of novel miRNAs in pepper

**Figure 3:**
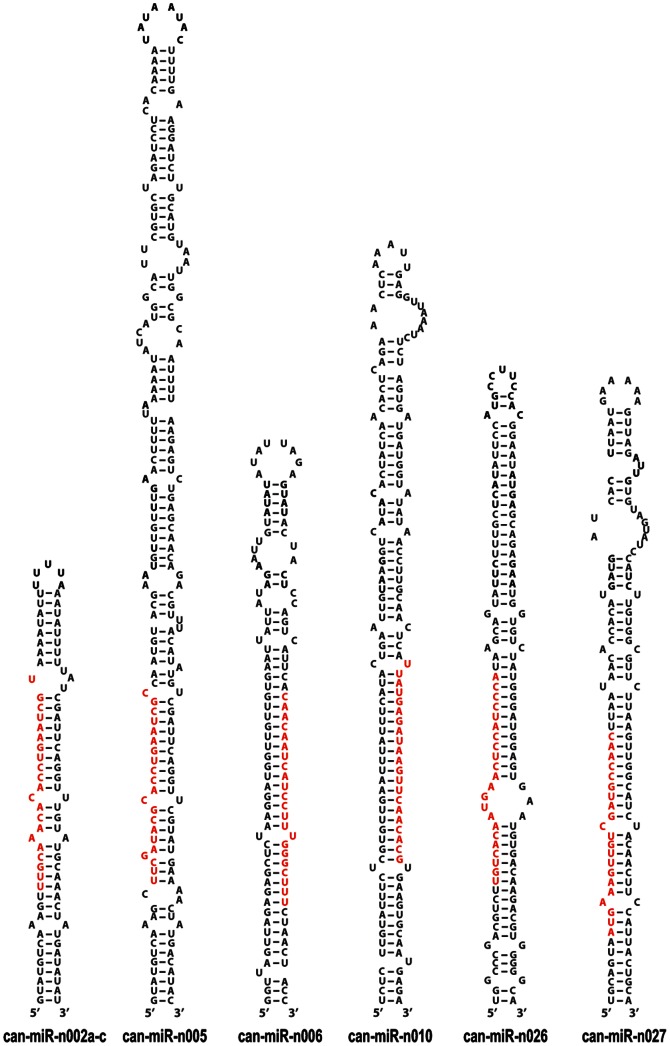
Representative hairpin precursors of novel miRNA families. Some examples of hairpin precursors of novel miRNAs are shown. Mature miRNAs are colored in red.

For the identification of novel miRNAs, the genome-aligned small RNAs, exclusive of conserved miRNAs, were analyzed according to several criteria; first, only the miRNAs of which precursors were folded into stem-loop structures in hairpin prediction were selected for novel miRNA candidates. Precursors of these novel miRNAs had negative folding free energies ranging from −355.30 to −19.80 according to RNAfold. The average free energy of these novel miRNAs was about −104.85, much lower that of other plant miRNA precursors (−59.5 kcal/mole in *A. thaliana* and −71.0 kcal/mol in *O. sativa*). We next applied base-paring criteria [20] between the miRNA and the other arm of the hairpin; (1) mismatched miRNA bases are four or fewer, (2) asymmetric bulges are two or less in size, and (3) one or less in frequency. In addition, we investigated whether these novel miRNA candidates contained both miRNA and miRNA* sequences in our sequencing libraries since the presence of miRNA* is strong evidence of precise biogenesis [20]. Finally, miRNAs showing high expression levels with a total frequency of 1,000 or higher were chosen to be studied for more strict identification of novel miRNAs. As a result, we identified 50 novel miRNAs that belong to 35 families (Table 3). The largest family was can-miR-n019 with four members, and most of the novel miRNAs were mapped to a single locus in the pepper genome, contrary to multiple loci of conserved families (Table 3). Some of the representative secondary structures of their precursors were shown in Figure 3 and the full list of secondary structures was provided in Dataset S2.

Classification of a large number of miRNAs from high-throughput sequencing data is usually faced with difficulty in some cases where multiple miRNAs accumulate from the same precursor. For example, there is a case where miRNA and miRNA* species are sequenced approximately at the same level, as is the case for ath-miR832 [29]. In the present study, likewise, can-miR-n009-5p and can-miR-n009-3p accumulated to approximately equal levels in most tissues examined, except the leaf (Table 3).

The sequencing frequencies of these novel miRNAs also showed that their expression had clear tissue-specific or developmental stage-specific patterns. For example, can-miR-n001 was sequenced in more than 1,000 reads in stem, root and flower, but not sequenced in leaf. can-miR-n002 was most abundantly in leaf (70,358 reads; the highest frequency of novel miRNAs in our libraries), and was moderately found in stem and root and was lowest in flower and fruit. can-miR-n026 had a similar expression pattern; abundant expression in leaf, moderate expression in stem and root, and low expression in flower and fruit. In contrast, can-miR-n006 was expressed abundantly in fruit and rarely in leaf, stem, root and flower. However, several novel miRNAs had similar expression patterns in all tissues (e.g., can-miR-n004), similar to some of the conserved miRNA families such as can-miR159, 160, 166, 171 and 172.

### Expression patterns of miRNAs in pepper

It has been reported that a large number of miRNAs in plants have tissue-specific and developmental stage-specific expression patterns [26], [30]–[32], some of which might have a wide range of crucial roles during development and stress adaptation [3], [33]. Since it has been reported that there were biases inherent in next-generation sequencing (NGS) technologies [34], northern blot analysis was expected to reveal more accurate *in vivo* expression patterns. Furthermore, detection of miRNAs by northern blot analysis could provide more direct evidence for their expression. Therefore, in this study, we performed northern blot analysis to observe tissue-specific or developmental stage-specific expression patterns in different tissues; leaf (L), stem (S), root (R), inflorescence (I), fruit (F) and seedling (Se).

**Figure 4:**
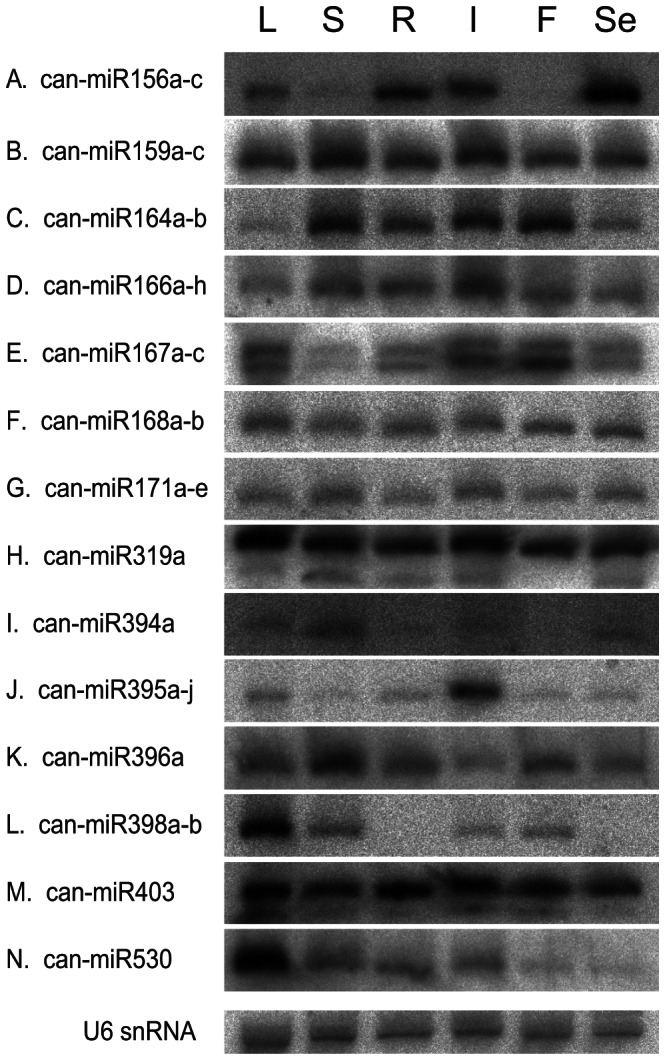
Expression patterns of conserved miRNAs in pepper. Total RNA was extracted from different tissues including, leaf (L), stem (S), root (R), inflorescence (I), mixed developmental stages of fruits (F) and seedling (Se). One of the blots probed for U6 snRNA is represented as a loading control here.

Among the 29 conserved miRNA families, 19 miRNA families were tested and 15 were detected as discrete bands (Figure 4). Four miRNA families (can-miR162, can-miR390, can-miR408, and can-miR477) were not detected by northern blot analysis. Expression levels of these miRNAs appeared not high enough to be detected by northern blot analysis [35]. As a result, tissue-specific and developmental stage-specific expression patterns were observed for some of conserved miRNAs. For instance, can-miR156 was expressed abundantly in the seedling, moderately in the root, inflorescence and leaf, but weakly in the stem and fruit (Figure 4A). Similarly, tissue-specific or developmental stage-specific expressions were observed in can-miR164, can-miR166, can-miR167, can-miR394, can-miR395, can-miR396, can-miR398, and can-miR530 (Figure 4C, D, E, I, J, K, L and N). The expression level of can-miR164 was high in the stem, root, inflorescence and fruit, and low in the leaf and seedling (Figure 4C). can-miR166 and can-miR396 were expressed lowly in the leaf and inflorescence, respectively (Figure 4D, K). can-miR167 was expressed highly in the leaf, inflorescence and fruit and lowly in the stem, root and seedling (Figure 4E). The expression level of can-miR394 was higher in stem than in other tissues (Figure 4I). can-miR398 and can-miR-530 accumulated in leaf (Figure 4L and N), and can-miR395 was exclusively expressed in the inflorescence (Figure 4J). However, can-miR159, can-miR168, can-miR171, can-miR319, and can-miR403 had no tissue-specific or developmental stage-specific expression patterns (Figure 4B, F, G, H, and M). They appeared to be expressed ubiquitously in all tissues. Some miRNAs, including can-miR167, can-miR319 and can-miR403 appeared as a doublet or triplet on northern blot analysis in all or part of the tissues.

The expression profiles obtained by northern blot analysis usually agreed with the sequencing data, as in the cases of can-miR159, can-miR167, can-miR168, can-miR171, can-miR394, can-miR395, can-miR396, can-miR403 and can-miR530 (Figure 4B, E, F, G, I, J, K, M and N, Table 1). However, we observed a discrepancy between northern blot analysis and sequencing frequencies although it was restricted to one or two tissues. Specifically, can-miR156 and can-miR398 were expressed more abundantly in leaf than stem although they were sequenced much less in leaf than stem (Figure 4A and L, Table 1). This reverse expression pattern between northern blot and sequencing data was observed for can-miR166 expression in leaf and root (Figure 4D, Table 1). In addition, can-miR164 expression showed a significant difference between leaf and root in spite of nearly same read number, and can-miR319 expression in leaf was as abundant as in other tissues despite its much lower sequenced frequency than other tissues (Figure 4C and H, Table 1).

**Figure 5:**
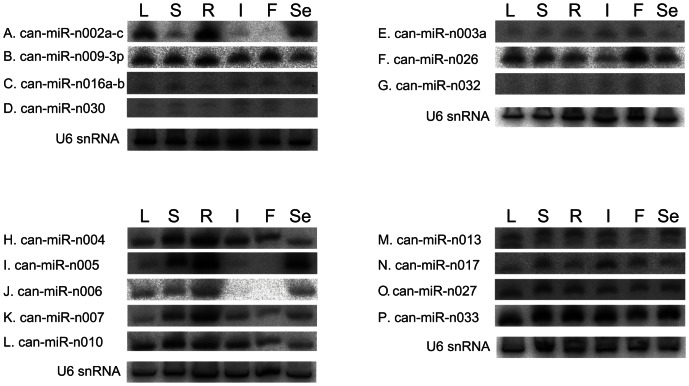
Expression patterns of novel miRNAs in pepper. Total RNA was extracted from different tissues, including leaf (L), stem (S), root (R), inflorescence (I), mixed stage of fruits (F) and seedling (Se). U6 snRNAs are shown as a loading control.

Subsequently, the expression patterns of novel miRNA families were analyzed by northern blot analysis. On the whole, 19 out of 35 novel miRNA families were subjected to northern blot analysis, and all of them (19) were detected. Representative results of the northern blot analysis for 15 novel miRNAs were shown in Figure 5. Overall, the expression level of many miRNAs was similarly high or low in all tissues; can-miR-n003, can-miR-n013, can-miR-n016, can-miR-n027, can-miR-n030, can-miR-n032 and can-miR-n033 (Figure 5E, M, C, O, D, G and P). Although these seven miRNAs did not show tissue-specific or developmental stage-specific expression patterns, the sequencing frequencies from different libraries suggested that four miRNAs (can-miR-n016, n027, n030 and n032) showed apparent tissue or developmental stage-dependent expression patterns (Table 3). In contrast, eight other miRNAs had tissue-specific or developmental stage-specific expression patterns. Specifically, can-miR-n002 was expressed abundantly in leaf, root and seedling, and weakly in stem, inflorescence and fruit (Figure 5A). can-miR-n026 was expressed ubiquitously in all of the tissues, but was especially high in fruit (Figure 5F). can-miR-n004 was more highly expressed in stem, root and inflorescence than leaf, fruit and seedling (Figure 5H). can-miR-n005 was expressed highest in root, moderately in stem and seedling, and lowest in leaf (Figure 5I). can-miR-n006 was abundantly expressed in leaf, stem, root and seedling, but not detected in inflorescence and fruit (Figure 5J). The expression level of can-miR-n007 was higher in stem and root, and lower in leaf, inflorescence, fruit and seedling (Figure 5K). The expression of can-miR-n010 was higher in leaf, stem, root and inflorescence, and lower in fruit and seedling (Figure 5L). can-miR-n017 was expressed moderately in stem and inflorescence, and slightly in leaf, root, fruit and seedling (Figure 5N). In the case of can-miR-n013 and can-miR-n030, a doublet was observed in all or part of the tissues (Figure 5M and D). Most of evolutionarily young miRNAs, such as species-specific or non-conserved miRNAs, have been reported to be expressed weakly [36], [37] whereas highly conserved miRNAs are expressed at a higher level [29], [36], [38]. This was observed for several novel miRNAs, which showed weak expression in northern blot analysis although they were selected by strict criteria, including a high frequency of 1,000 or higher. In contrast, some of the novel miRNAs were expressed at a high level in northern blot analysis as well (Figure 5A, H, I, K, L, F and P), suggesting that they could play more roles in pepper-specific biology than the other weakly expressed novel miRNAs. In some cases, there were inconsistencies between expression levels obtained by Illumina/Solexa sequencing and northern blot analysis. As mentioned above, it is possible that sequencing technology could cause biases for certain sequences. Another possibility is that the probes used for northern blot analysis might have captured heterogeneous miRNAs.

### Predicted targets of miRNAs in pepper

Assuming that plant miRNAs have a perfect or nearly-perfect match to their target mRNAs, FASTA searches were performed to predict targets of conserved miRNAs, and the potential targets were chosen by a position-dependent penalty scoring system in which predicted targets with penalty scores of four or less were selected. As a result, we identified 334 potential target genes from 26 out of 29 conserved miRNA families (Table S1), and the representative targets for each miRNA family are listed in Table 4.

**Table 4:** Representative targets of conserved miRNAs.

Conserved miRNA	S	Predicted targets	Contig	Reference species
can-miR156a-c	2	transcription factor squamosa promoter binding protein-like	contig18156	*Eucalyptus grandis*
can-miR159a-c	2.5	GAMYB-like1	contig47618	*Solanum lycopersicum*
can-miR160	1	auxin response factor 10	contig39065	*Solanum lycopersicum*
can-miR164a-b	3	NO APICAL MERISTEM	contig71511	*Solanum lycopersicum*
can-miR166i-j	4	Cyclic nucleotide-gated ion channel, putative	contig89952	*Ricinus communis*
can-miR167a-c	2.5	multidrug resistance pump, putative	contig82999	*Ricinus communis*
can-miR168c	3	AGO1	contig45783	*Nicotiana benthamiana*
can-miR169a-g	4	beta-mannosidase enzyme	contig68856	*Solanum lycopersicum*
can-miR171a-e	1	GRAS family transcription factor	contig12271	*Populus trichocarpa*
can-miR172g	1	transcription factor APETALA2	contig61069	*Vitis vinifera*
can-miR319g	4	TCP family transcription factor	contig35018	*Cyclamen persicum*
can-miR390a-b	2.5	serine/threonine-protein kinase bri1, putative	contig30760	*Ricinus communis*
can-miR393a-b	2.5	TIR1-like protein	contig11388	*Solanum lycopersicum*
can-miR394a	1	F-box family protein	contig90221	*Citrus trifoliata*
can-miR395a-j	2	sulfate/bicarbonate/oxalate exchanger and transporter sat-1	contig93668	*Populus trichocarpa*
can-miR396a	3	AtGRF4	contig34945	*Arabidopsis thaliana*
can-miR397a	1.5	diphenol oxidase	contig79248	*Nicotiana tabacum*
can-miR398c	3.5	myb family transcription factor	contig67120	*Arabidopsis thaliana*
can-miR399f-g	2.5	inorganic phosphate transporter	contig116462	*Solanum lycopersicum*
can-miR408a	4	phosphoglucomutase, putative	contig34741	*Ricinus communis*
can-miR482c	3	resistance protein PSH-RGH6	contig23981	*Solanum tuberosum*
can-miR530	1	TZP; DNA binding/nucleic acid binding/zinc ion binding	contig77398	*Arabidopsis thaliana*
can-miR827	4	chaperone protein dnaJ-related	contig15445	*Arabidopsis thaliana*
can-miR1446a-b	3	GRAS family transcription factor	contig32571	*Populus trichocarpa*
can-miR4376	4	casein kinase, putative	contig20369	*Ricinus communis*
can-miR4414b	0	peroxin-3 family protein	contig36139	*Arabidopsis thaliana*

**S**: Total penalty score.

In general, most of the targets predicted for conserved miRNAs in pepper were transcription factors (Figure 4). For instance, predicted targets such as SQUAMOSA promoter binding proteins (SBP), auxin response factors (ARF), growth regulating factors (GRF), no apical meristem (NAM) and MYB family belong to transcription factors (Table 4). Therefore, similar to other plants [3], pepper miRNAs appear to play a significant role in plant development and growth.

We were able to predict the functions of miRNAs in pepper based on the functions known in other plants; we found most of the predicted targets of conserved miRNAs in pepper also had a conserved function with miRNA targets in other plants. The miRNA families having conserved targets were: can-miR156, can-miR159, can-miR160, can-miR164, can-miR167, can-miR171, can-miR172, can-miR319, can-miR394, can-miR395, can-miR396, and can-miR482 (Table 4; See Table S1 for total predicted target list). For example, SBP transcription factors, known to regulate flowering time in plants, were previously reported to contain complementary sequence of miR156 in other plants [39]. Our results suggested that SBP transcription factors can be considered as the targets of can-miR156 as well. MYB transcription factors, which are known to regulate meristem formation and seed development [39]–[42], are conserved targets of miR159/319 families. We identified homologous transcripts of MYB genes in pepper, which were considered as the target of can-miR159 and can-miR319 families. ARF genes, which are known to play important roles in plant development as activators or repressors of auxin-responsive transcription [43], are conserved targets of miR160/167 families in *Arabidopsis* [39], [40]. Our results also indicate that ARFs are presumed to be targeted by the can-miR160 and can-miR167 families. The miR482-mediated regulation of NBS-LRR disease resistance proteins, recognizing specific pathogen effectors and trigger resistance responses, are conserved in several plant species [44]–[46]. Our results suggest that NBS-LRR disease resistance proteins in pepper were also presumed to be targeted by can-miR482 families. Similarly, many other targets, including GRF, F-box, TCP transcription factors, and NAM are potential targets of can-miR396, can-miR394, can-miR319, and can-miR164, respectively.

Among 35 novel miRNA families, we identified a total of 57 potential target genes from 19 novel miRNA families (Table 5). Unlike conserved miRNAs, the targets of novel miRNAs were not enriched in transcription factors; only a target, WRKY transcription factor, was predicted. The largest group of predicted targets was F-box family proteins, predicted to be targeted by can-miR-n002 and can-miR-n005, both of which showed similar expression patterns in terms of high expression levels in root and seeding (see Figure 5). The second largest group was composed of the following two groups: (i) disease resistance-related genes such as verticillium wilt disease resistance proteins, late blight resistance proteins and NBS-LRR type disease resistance proteins; (ii) protein kinase genes. Among the protein kinase group, leucine-rich repeat protein kinases were known to be critical components of PTI (PAMP-triggered immunity), one of the plant immune systems triggered by pathogen-associated molecules [47], [48]. Therefore, miRNAs predicted to target these kinases may also be involved in the immune system of pepper through cooperation with other miRNAs related to regulate disease resistance gene expression in times with and without pathogen infection. The fourth largest group was GDSL-lipase like chlorogenate-dependent caffeoyltransferase precursors, known to have multifunctional properties and be related in lipid metabolism [49].

**Table 5:** Predicted targets of novel miRNAs.

Novel miRNA	S	Predicted targets	Contig	Reference species
can-miR-n001	4	3-hydroxy-3-methylglutaryl coenzyme A reductase	contig22068	*Withania somnifera*
can-miR-n001	3.5	dehydroquinate dehydratase	contig58368	*Solanum tuberosum*
can-miR-n002a-c	0	F-box family protein-1	contig77869	*Populus trichocarpa*
can-miR-n002a-c	1	F-box family protein-2	contig76085	*Populus trichocarpa*
can-miR-n002a-c	3	F-box family protein-3	contig57088	*Populus trichocarpa*
can-miR-n002a-c	3	F-box family protein-4	contig51570	*Populus trichocarpa*
can-miR-n002a-c	4	F-box family protein-5	contig72152	*Populus trichocarpa*
can-miR-n002a-c	4	F-box family protein-6	contig115337	*Populus trichocarpa*
can-miR-n003a	4	HGWP repeat containing protein-like	contig2445	*Oryza sativa*
can-miR-n003b-d	3	S-adenosylmethionine-dependent methyltransferase	contig51699	*Ricinus communis*
can-miR-n003b-d	3.5	pentatricopeptide repeat-containing protein-1	contig48839	*Ricinus communis*
can-miR-n003b-d	3.5	pentatricopeptide repeat-containing protein-2	contig174374	*Ricinus communis*
can-miR-n004	3.5	SPla/RYanodine receptor (SPRY) domain-containing protein	contig31594	*Arabidopsis thaliana*
can-miR-n005	0	F-box family protein-7	contig33836	*Populus trichocarpa*
can-miR-n005	4	F-box family protein-8	contig15361	*Populus trichocarpa*
can-miR-n006	4	ATL2; protein binding/zinc ion binding	contig44288	*Arabidopsis thaliana*
can-miR-n006	4	ATFH8 (formin 8)	contig148991	*Arabidopsis thaliana*
can-miR-n006	4	WRKY27; transcription factor	contig127277	*Arabidopsis thaliana*
can-miR-n006	4	cytochrome b5 isoform Cb5-D	contig23616	*Vernicia fordii*
can-miR-n007	4	B-block binding subunit of TFIIIC	contig93287	*Arabidopsis thaliana*
can-miR-n007	3	IBS1 (IMPAIRED IN BABA-INDUCED STERILITY 1)	contig45885	*Arabidopsis thaliana*
can-miR-n007	4	PIP5K9	contig23532	*Arabidopsis thaliana*
can-miR-n007	4	serine-threonine protein kinase, plant-type	contig47921	*Ricinus communis*
can-miR-n007	4	EMB2730 (EMBRYO DEFECTIVE 2730); 3′-5′-exoribonuclease	contig35994	*Arabidopsis thaliana*
can-miR-n009a,b-5p	3	pumilio	contig17550	*Ricinus communis*
can-miR-n009a,b-3p	4	salt-inducible protein-like	contig108504	*Arabidopsis thaliana*
can-miR-n009a,b-3p	3	leucine-rich repeat receptor-like protein kinase	contig11126	*Arabidopsis thaliana*
can-miR-n009a,b-3p	4	protein serine/threonine kinase	contig4968	*Arabidopsis thaliana*
can-miR-n009a,b-3p	4	Hcr9-OR2C	contig43762	*Solanum pimpinellifolium*
can-miR-n009a,b-3p	3	verticillium wilt disease resistance protein Ve2	contig93899	*Solanum lycopersicum*
can-miR-n009a,b-3p	2.5	peru 1	contig5949	*Solanum peruvianum*
can-miR-n010	4	leucine-rich repeat transmembrane protein kinase, putative	contig79370	*Arabidopsis thaliana*
can-miR-n013	2.5	putative disease resistance protein	contig8396	*Solanum tuberosum*
can-miR-n013	4	NBS-LRR type disease resistance protein	contig71033	*Ipomoea batatas*
can-miR-n013	3.5	nbs-lrr resistance protein	contig79295	*Populus trichocarpa*
can-miR-n013	3.5	subtilisin-like protein	contig24063	*Arabidopsis thaliana*
can-miR-n013	4	leucine-rich repeat-containing protein, putative	contig9724	*Ricinus communis*
can-miR-n013	3	NRC1	contig8119	*Solanum lycopersicum*
can-miR-n014	3.5	serine/threonine protein kinase	contig13939	*Ricinus communis*
can-miR-n014	4	Putative late blight resistance protein	contig58329	*Solanum demissum*
can-miR-n017	4	phytochrome A	contig57587	*Solanum tuberosum*
can-miR-n019a-d	4	F-box family protein-9	contig153047	*Populus trichocarpa*
can-miR-n024	2	Methyltransferase FkbM	contig137730	*Limnobacter sp. MED105*
can-miR-n024	4	mitochondrial processing peptidase-like	contig20814	*Solanum tuberosum*
can-miR-n026	4	MAPKKK19	contig61678	*Arabidopsis thaliana*
can-miR-n026	4	serine/threonine-protein kinase bri1	contig79519	*Ricinus communis*
can-miR-n026	4	esterase	contig62897	*Zea mays*
can-miR-n026	2	GDSL lipase-like chlorogenate-dependent caffeoyltransferase precursor-1	contig135160	*Solanum lycopersicum*
can-miR-n026	2	GDSL lipase-like chlorogenate-dependent caffeoyltransferase precursor-2	contig77881	*Solanum lycopersicum*
can-miR-n026	4	GDSL lipase-like chlorogenate-dependent caffeoyltransferase precursor-3	contig151849	*Solanum lycopersicum*
can-miR-n027	4	short-chain dehydrogenase/reductase (SDR) family protein	contig25643	*Arabidopsis thaliana*
can-miR-n031	4	calmodulin binding protein-like protein	contig34878	*Arabidopsis thaliana*
can-miR-n031	4	neutral leucine aminopeptidase protein	contig48994	*Solanum lycopersicum*
can-miR-n032	4	phytoene desaturase	contig69939	*Nicotiana benthamiana*
can-miR-n032	4	small multi-drug export protein	contig48930	*Zea mays*
can-miR-n033	4	nbs-lrr resistance protein	contig47332	*Populus trichocarpa*
can-miR-n033	3.5	Putative late blight resistance protein	contig14378	*Solanum demissum*

**S**: Total penalty score.

### Validation of miRNA-directed target cleavage

**Figure 6:**
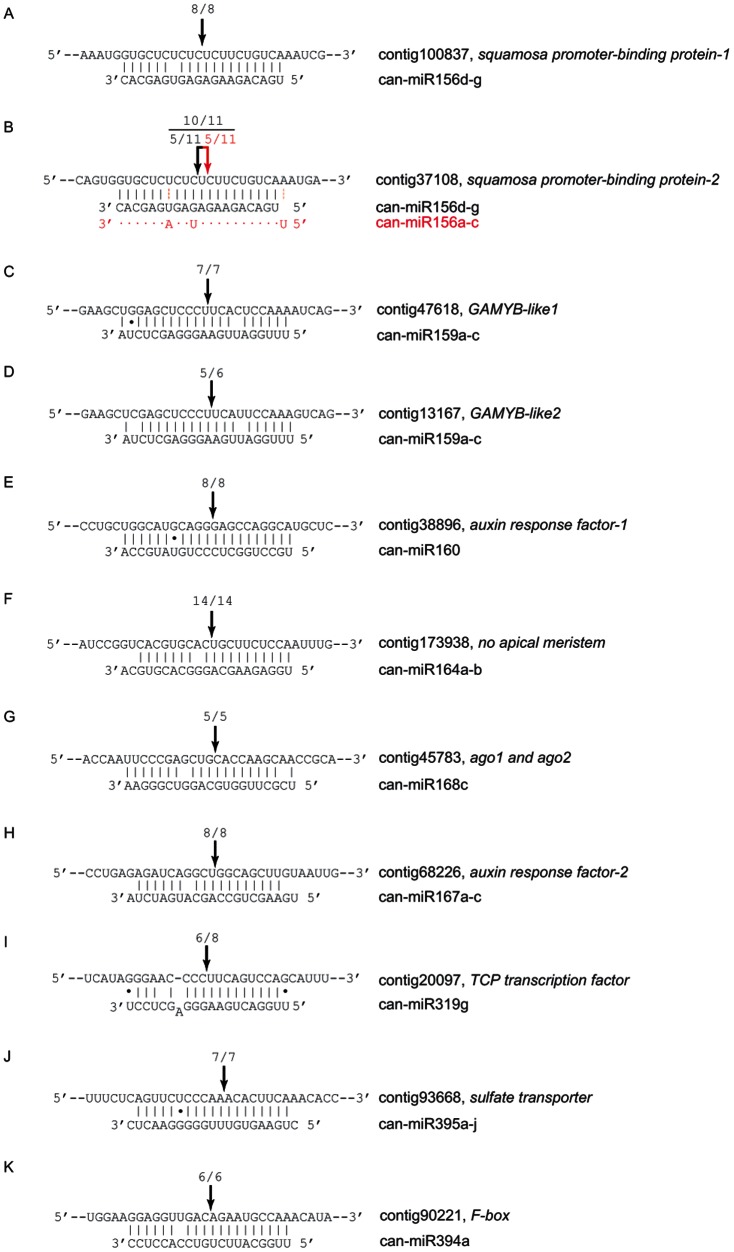
Validation of conserved miRNA-directed target cleavage in pepper 5′ RACE-PCR products terminating at a given position indicated above each miRNA-target duplex. Fractions refer to the number of independently cloned 5′ RACE products whose 5′ end terminated at the indicated position (numerator) over the total number of sequenced clones (denominator). Note that different cleavage sites were mapped to the same target gene, depending on sequence specificity of can-miR156 (Figure 6B).

It is necessary to experimentally validate whether computationally predicted targets are actually regulated by miRNAs in pepper by means of miRNA-directed cleavage of these targets because plant miRNAs regulate their target genes mainly by cleaving them [3], [15]. Therefore, 5′ RACE, universally used for detecting miRNA-directed target cleavage, was carried out for some of the predicted targets of conserved miRNAs; SBP, ARF, NAM, F-box, MYB, Argonuate, TCP, and sulphate transporter (Figure 6). We noticed that one of the SBP transcription factor was targeted only by can-miR156d-g (Figure 6A), whereas another SBP transcription factor was targeted both by can-miR156a-c and can-miR156d-g (Figure 6B). In addition, two cleavage sites of the SBP transcription factor were mapped on the mRNA target, owing to a single length difference between can-miR156a-c and can-miR156d-g (Figure 6B). Likewise, the miRNA-directed target cleavages of ARF, NAM, F-box, MYB, Argonuate, TCP, and sulphate transporter were validated as well (Figure 6C, D, E, F, G, H, I, J and K).

**Figure 7:**
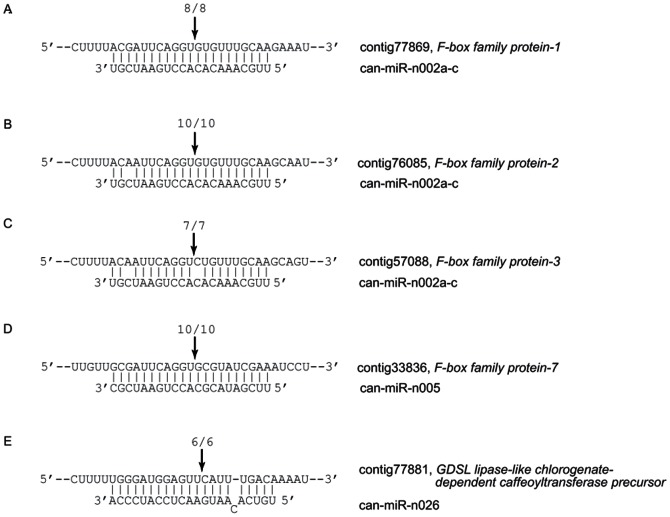
Validation of novel miRNA-directed target cleavage in pepper. 5′ RACE-PCR products terminating at a given position indicated above the each miRNA-target duplex with the frequency of clones. Fractions refer to the number of independently cloned 5′ RACE products whose 5′ end terminated at the indicated position (numerator) over the total number of sequenced clones (denominator).

We also carried out 5′ RACE analysis for many of the novel miRNA targets. Of the 57 predicted targets of novel miRNAs, 26 targets were tested. Five of them were validated, but most of the remaining targets (21) could not be (Table S4). Overall, we noticed that most of the predicted targets of novel miRNAs had high-penalty scores (49 out of 58, 86% had penalty scores of 3 or 4) (i.e., low sequence complementarity to miRNAs), especially for targets which could not be validated. On the contrary, the validated targets generally had lower penalty scores (i.e., higher sequence complementarity to miRNAs) (Table S4). The novel miRNA-directed target cleavage was validated for F-box family proteins and the GDSL lipase-like chlorogenate-dependent caffeoyltransferase precursor (Figure 7). F-box family proteins are known to play a role in protein degradation as one component of SCF complex [50] and also associated with regulation of cell cycle and signal transduction [51]. Among the predicted novel miRNA targets, F-box family proteins were the largest group (Table 5) and were predicted to be targeted by can-miR-n002 and can-miR-n005, both of which had a similar expression pattern; abundant expression in root and seedling (Figure 5). Additionally, F-box family proteins share higher sequence complementarity (i.e., low penalty score) to can-miR-002 and can-miR-005. Consequently, we obtained positive results of four F-box family proteins (Figure 7A, B, C and D). GDSL lipase-like chlorogenate-dependent caffeoyltransferase precursor, another validated target, had a single nucleotide bulge on can-miR-n026, but the miRNA-mediated cleavage was still observed (Figure 7E).

In summary, we experimentally validated that conserved and novel miRNAs in pepper negatively regulate the expression of many transcription factors (SBP, ARF, MYB, NAM, F-box and TCP), small RNA biogenesis protein (AGO), sulphate transporter and GDSL lipase-like chlorogenate-dependent caffeoyltransferase precursor by the miRNA-directed cleavage mechanism (Figure 6 and 7).

### Predicted novel targets of conserved miRNAs

In our target prediction analysis, some putative target genes of conserved miRNAs were assumed to be a pepper-specific. Unlike conserved targets, the novel pepper-specific targets were not enriched in transcription factors, but included mRNAs encoding the gypsy/Ty-3 retroelement polyprotein, splicing factor, DNA methyltransferase, helicase protein, and copper-transporter, indicating that the corresponding novel targets of conserved miRNAs may be involved in specific biological processes in pepper (Table S1).

**Figure 8:**
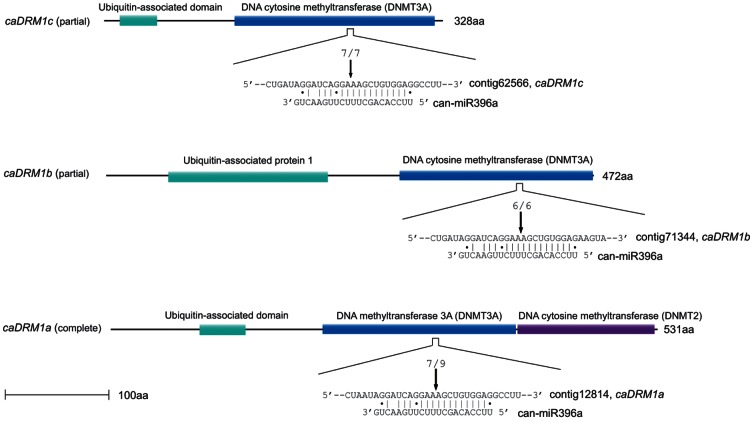
Novel targets of conserved miRNAs, DRM methyltransferase proteins, are regulated by microRNA-directed cleavage in pepper. Diagrammatic representations of the miR396a-directed cleavage sites of full- or partial-length DRM methyltransferase proteins. The sequences corresponding to the miR396a interaction site in DRM methyltransferase are shown. The fraction of cloned 5′ RACE PCR products terminating at a given position is indicated above each duplex. Fractions refer to the number of independently cloned 5″ RACE products whose 5′ end terminated at the indicated position (numerator) over the total number of sequenced clones (denominator). The domains of target protein were predicted using HHpred, and the regions that encode conserved domains in the DRM methyltransferase family are indicated by the same color.

Among them, one target gene is worth describing specifically. Some DNA methyltransferase genes were predicted to be targeted by miR396 in pepper, and these putative target genes were validated by the 5′ RACE assay (Figure 8). In order to make a detailed analysis of these target genes, the predicted target protein sequence was submitted as a query in the HHpred server (http://toolkit.tuebingen.mpg.de/hhpred) to search for a putative domain [52]. We identified a C-terminal catalytic domain showing sequence similarity to mammalian DNA methyltransferase 3A (DNMT3) and unique N-terminal ubiquitin associated (UBA) domains which are specifically present in DRM methyltransferase in plants (Figure 8). Since DRM methyltransferase is the plant ortholog of DNMT3 and has a unique N-terminal UBA domain [53], we assumed that this is the predicted target of a DNA methyltransferase belonging to the DRM protein family, the principle *de novo* methyltransferase in plants.

Furthermore, we performed a BLAST search against other plants genome databases to investigate patterns of conservation and phylogenetic relationships among many plant species. As a result, we found that protein sequences for DRM methyltransferase were highly conserved (shown as red in Figure S1 and closely related to other plants (Figure S2). We also found that miR396 families are extensively conserved through many other plant species (Figure S3A). Therefore, total RNAs from tobacco, tomato, potato and pepper were subjected to northern blot analysis to assess expression of miR396. Notably, we found that expression of miR396 was higher in tomato and pepper than that in tobacco and potato (Figure S3B).

In particular, with the help of computational target prediction, we were also able to identify some DRM methyltransferase genes sharing high sequence complementarity to the miR396 in other plant species, including tomato, potato and tobacco, implying the possibility that miR396 targeting is conserved in Solanaceae (Figure S3C). To see whether the function of miR396, which targets DRM methyltransferase genes, is conserved in Solanaceae, we experimentally tested miR396-directed cleavage of putative targets using the 5′ RACE assay. As a result, we found that similar miR396 - DRM methyltransferase pathway should exist in tomato (Figure S3D), but we failed to amplify a major PCR product as a diagnostic for miR396-directed cleavage in tobacco and potato (data not shown). As mentioned above, the northern blot analysis revealed that the expression of miR396 was higher in tomato and pepper than in tobacco and potato. Moreover, DRM methyltransferase genes shares higher sequence complementarity to miR396 (i.e., lower penalty score) in pepper and tomato than those in tobacco and potato. These might only explain the obvious reasons why DRM methyltransferase genes were only targeted by miR396 in pepper and in tomato. However, these observations still require further investigation to determine why miR396 shares conserved targets in pepper and tomato, but not in tobacco and potato, all of which belong to Solanaceae.

Taken together, at least in pepper and tomato, we assumed that the miR396 family members are possibly involved in *de novo* cytosine methylation, which is tightly linked to transcriptional silencing of repetitive sequences, including transposons and other mobile DNA elements.

### A potential link between miRNA expression and fruit development

**Figure 9:**
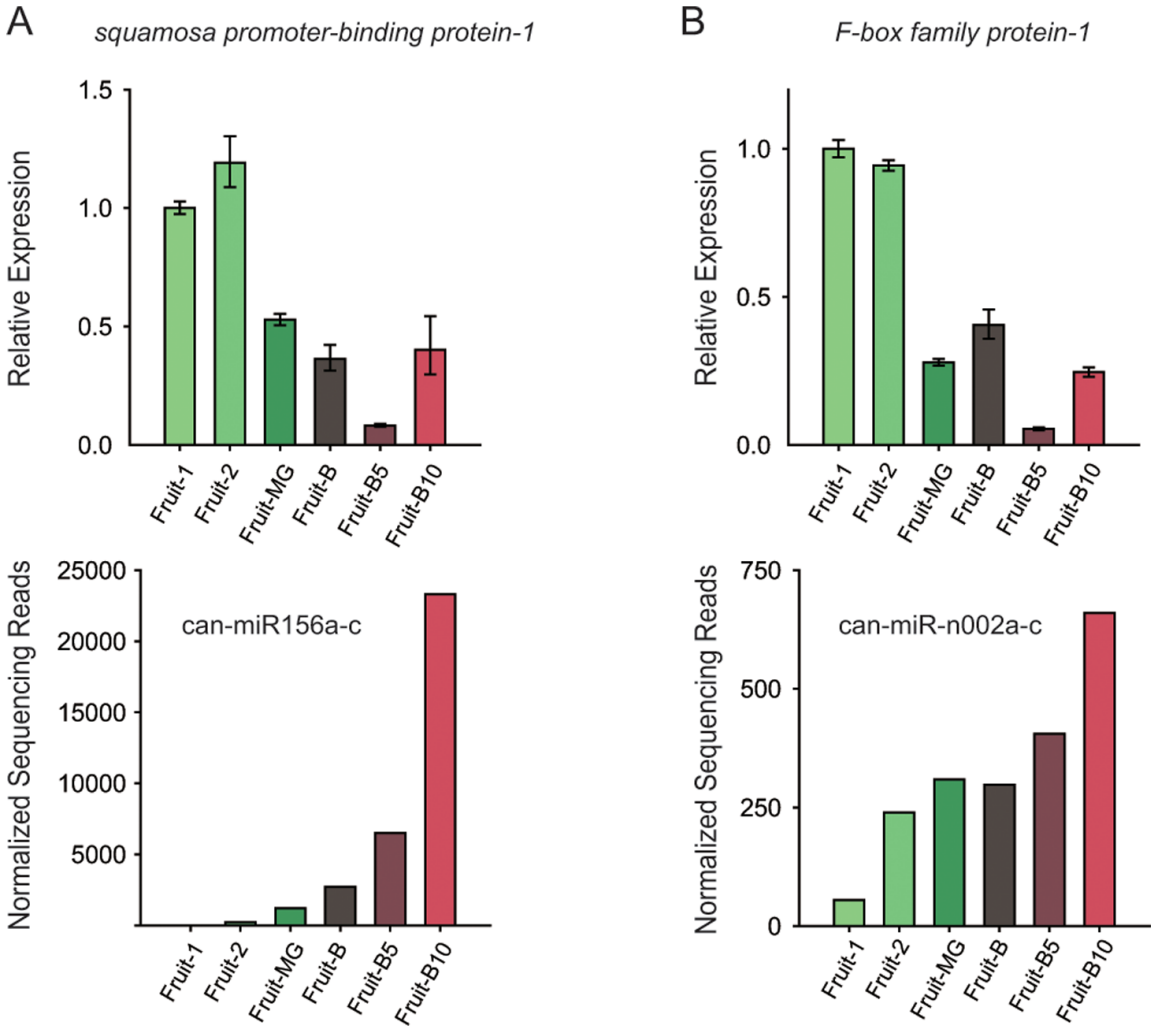
A potential link between miRNA expression and fruit development in pepper. (A–B) (Top Panel) qRT-PCR analysis of the SBP transcription factor (A) and F-box mRNA (B) expression from each development stage of fruits (from fruit-1 to fruit-B10). *18s rRNA* and *actin* were used as normalizing controls. Relative mean expression ± SD is shown. (Bottom Panel) The normalized sequencing reads of can-miR156a-c (A) and can-miR-n002a-c (B) from each development stage of fruits (from fruit-1 to fruit-B10) are shown.

We noticed that some miRNAs, whose targets were validated experimentally in this study, exhibited prominent changes in expression levels (based on the normalized sequencing reads) during fruit development stages (i.e., from fruit-1 to fruit-B10). In particular, it is worth pointing out that the accumulation of can-miR156a-c substantially increased during the fruit development of pepper (Figure 9A). In *Arabidopsis*, miR156 regulates SBP transcription factors, and thus, modulates the vegetative phase transition through its temporal expression [103]. Similarly, among the novel miRNAs, the expression of can-miR-n002a-c, whose target was validated as the F-box protein, was up-regulated upon development from early stage to late stage of fruits (Figure 9B).

To elucidate the potential regulatory roles of these miRNAs in pepper fruit development, we performed qRT-PCR analysis of the target mRNAs, including the SBP-transcription factor and F-box protein. As a result, we found that expression of these two target mRNAs gradually decreased in general during fruit development and hence was negatively correlated with the expression of their corresponding miRNAs (Figure 9A and 9B). The validation of miRNA-directed cleavage of these target mRNAs, combined with the results of qRT-PCR analysis, likely suggests that some miRNAs in pepper may play a role in fruit development.

Tomato has emerged as a useful model for fleshy fruit development and climacteric fruit ripening [54]. In tomato, the *SBP-box* gene residing at the colorless non-ripening (*CNR*) locus, which plays vital roles in fruit ripening [55] was shown to be targeted by miR156 [56]. The over-expression of miR156 resulted in down-regulation of *CNR* expression and caused the red fruit color lighter than wild-type [57], suggesting that the expression of the *CNR* mRNA is, in part, under the control of miR156 expression. In pepper, the *SBP-CNR* mRNA, a homologous gene to tomato, was predicted to be a target of miR156 as well (Table S1), but we did not find negative correlation of the expression levels between miR156 and *SBP-CNR* mRNA (data not shown).

The two F-box genes, *EBF1* and *EBF2* were shown to function in ethylene response by regulating the ubiquitin 26S proteasome-dependent EIN3/EIL turnover [58], and in tomato, co-silencing of both *SI-EBF*s (*EBF-1* and *-2*) resulted in ethylene-associated phenotype [59]; however, none of the miRNAs has previously been linked to these members of the F-box family so far, which is consistent with our miRNA target prediction results. Nevertheless, our results here provide a possible link that other members of SBP-box or the F-box family are under the control of miRNAs during the fruit ripening in pepper.

## Discussion

The study of plant miRNAs from non-model species and their functional roles are still in their infancy. However, many recent studies have demonstrated that plant miRNAs are involved in a variety of functional roles in developmental and morphogenetic processes [60]–[63]. Even though pepper is the most cultivated spice and one of the most economically significant vegetable crops worldwide, systematic study of pepper miRNAs has been reported scarcely. A previous study of pepper miRNAs using an *in silico* approach [13] was able to identify a very limited number of miRNAs. Many previous studies employed expressed sequence tag (EST) analysis approaches to identify miRNAs from other species [64], [65], especially for species whose genomes were poorly understood. There are often a limited number of ESTs available in databases, and therefore a great proportion of protein-coding genes are missed, which makes effective computational prediction of miRNAs and their target genes difficult. Here using draft genome sequences, we have extensively identified a large number of miRNAs in pepper from 10 different libraries. This work therefore provides the first reliable draft of the pepper miRNA transcriptome. We successfully differentiated our miRNAs from other small RNAs by extensive investigation of pre-miRNA fold-back structures. From the 10 different libraries, we found that many pepper miRNAs exhibited tissue-specific expression, as is commonly inferred [66]. We further validated and observed these patterns in the northern blot analysis as well.

### Conserved miRNAs in pepper

In *Arabidopsis*, ath-miR156, which is involved in floral development and phase change by targeting SBP transcription factors, is one of the most abundantly expressed miRNAs [24]. Expression of can-miR156 reached its highest level at the seedling stage as previously reported in other plants [67]–[69], suggesting it regulates juvenile-to-adult phase transition in pepper. Previous studies suggested that over-expression of ath-miR156 affected phase transition from vegetative growth to reproductive growth [70]–[72], including a decrease in apical dominance [70], [73], [74], which is in full agreement with our expression analysis where can-miR156 was barely expressed in stem and fruit. The 5′ RACE assay confirmed that some members of SBP transcription factor families are regulated by the miRNA-directed cleavage mechanism, depending on sequence specificity of can-miR156. Similar to can-miR156, can-miR164 was temporally regulated because its expression was low in the seedling and high in adult tissues except for the leaf. Expression of ath-miR164 negatively regulates cell death by repressing the NAC transcription factor *ORESARA1* (*ORE1*), a positive regulator of cell death [75], [76]. In addition, decreased expression of ath-miR164 leads to increased expression of *ORE1* with leaf age [76]. This correlates with our expression analysis where the expression of can-miR164 was barely detectable in adult leaves, suggesting an analogous role in repressing *ORE1* in pepper. ath-miR166, which targets the HD-ZIP gene family, is conserved in many land plants [77]. In *Arabidopsis*, over-expression of ath-miR166 resulted in seedling arrest and diverse phenotypic changes, especially in floral structure and in shoot apical meristem (SAM) formation [78], [79]. We found that can-miR166 was highly expressed in flower followed by stem and root, and weakly in the seedling and leaf which was closely associated with their over-expression consequences.

In *Arabidopsis*, ath-miR159 and ath-miR319 share 17 identical nucleotides in their 21 nt mature miRNA sequences, and they both play significant roles in plant development, morphogenesis and reproduction [80], [81]. Computational prediction suggests that ath-miR159 and ath-miR319 might potentially regulate both MYB and TCP transcription factors [80], [81] owing to their sequence similarity. However, when studied *in vivo*, over-expression of ath-miR159 specifically down-regulated *MYB* mRNA expression, and over-expression of ath-miR319 specifically down-regulated *TCP* mRNA expression in *Arabidopsis* [82]. Similarly, in pepper, can-miR159 and can-miR319 are seemingly related due to their similarity in mature miRNA sequences. The 5′ RACE assay confirmed that can-miR159 and can-miR319 specifically regulate MYB transcription factors and TCP transcription factors, respectively. This result is consistent with those reported for *Arabidopsis*. Expression of miR159 is abundant in all tissues and widespread over the whole plant [67]. Similarly, we found expression of can-miR159 is stably expressed in all tissues in pepper as well. However, miR319 is known to be expressed at much lower level and restricted to certain tissues and specific developmental stages in other plants [67], which seems to be different from can-miR319 whose expression is consistently high in all of the tissues tested.

A previous study demonstrated that miR395 was induced under sulfate starvation conditions [83]. Several genes, including sulfate transporter and ATP sulfurylases (APS), both of which are involved in the sulfur assimilation pathway, are regulated by miR395 in *Arabidopsis* [84], [85]. From our target prediction analysis, can-miR395 was also predicted to target sulfate transporter and APS. We tested the can-miR395-directed cleavage of sulfate transporter by the 5′ RACE assay. We found that cleavage in the position corresponding to 9^th^ and 10^th^ nucleotides within their complementary site was clearly detected while no other cleavage product was found. Although miRNAs are generally known to induce cleavage of their target mRNA between 10^th^ and 11^th^ nucleotides, non-canonical cleavage sites have also been reported in plants [24], [83], [86] and in mammals [87]. Interestingly, a recent study of miR395 in *Arabidopsis* reported the miR395-directed cleavage of the *APS3* mRNA in the position corresponding to 9^th^ and 10^th^ nucleotides in their complementary site [84], which is similar to our 5′ RACE analysis.

### Novel miRNAs in pepper

Many earlier studies repeatedly reported that non-conserved miRNAs are generally less abundant, more divergent, processed less precisely, and tend to lack of experimentally verifiable targets, suggesting that most are neutrally evolving [88]–[90]. Therefore, we expected that identification and characterization of novel miRNAs in pepper might be challenging, as there were many things to consider.

The variation in mature miRNA sequences, especially that of 5′ terminal nucleotides, often showed differential Argonaut protein association [91], [92], and hence the mature miRNA sequence itself is now considered to be an important determinant of proper sorting into functional Argonaut complex. Therefore, the imprecise excision of the mature miRNAs from the stem-loop precursor could produce a functionally unstable miRNAs. For these reasons, we employed very stringent standard for the identification of novel miRNA candidates, as followed: (1) one of the proofs of miRNA biogenesis; the presence of the miRNA star sequences, forming a miRNA/miRNA* duplex with two nucleotides, 3′ overhang; (2) the presence of reliable stem-loop structures with proper folding free-energy; (3) minimal in size and frequency of asymmetric bulges, along with four or fewer mismatched miRNA bases within miRNA/miRNA* duplex; (4) highly expressed small RNAs with frequency of 1,000 or higher in at least one or more from 10 different libraries examined.

As a result, we were able to discover a total of 47 highly reliable novel miRNAs candidates from various pepper tissues. These novel miRNAs had many predicted targets; consequently we performed a 5′ RACE assay to reveal miRNAs involved in pepper-specific biology, but most of them could not be validated, which are consistent with previous studies, where most of the non-conserved miRNAs had no experimentally supported targets [9], [93], in contrast to the high validation rate of conserved miRNA targets. There are several possible explanations for this observation. (1) The novel miRNA targets that gave negative results upon 5′ RACE analysis, other than technical failures, could be false positive prediction owing to the limited genome annotation currently available in pepper, which was the major hindrance to high confidence prediction of miRNA targets. (2) The non-conserved miRNAs and their target genes are not co-expressed in the same tissue during development [88]. (3) The abundance of novel miRNAs is substantially lower than that of conserved miRNAs [36], [37], as is observed in our case as well. In addition, we noticed that most of the predicted targets of novel miRNAs had high-penalty score; low sequence complementarity. (4) The non-conserved miRNAs might function mainly through translational inhibition [4], [5], which is also supported by the fact that the level of non-conserved miRNA target transcripts were largely unchanged in miRNA biogenesis mutants [22]. (5) Some of the putative novel miRNAs are not *bona fide* miRNAs. Although we provided one of the major proofs of biogenesis where the perfect miRNA star sequences were found, we still did not demonstrate DCL1 dependency of these novel miRNAs because a *dcl1* mutant is not yet available for pepper. To conclude, while none of these possibilities can be ruled out at this point, it is currently difficult to explain the lack of experimental verifiable targets of the majority of non-conserved miRNAs. The other possible regulatory mechanisms behind this still remain to be elucidated.

### can-miR396-directed cleavage of DRM methyltransferase

Recently, DNMT3 was defined as a potential target of hsa-miR143 in colorectal cancer cells [94]. Also in plants, methyltransferase2 (MET2), another class of DNA methytransferase, was suspected to be a potential target of ath-miR773 in the mature pollen of *Arabidopsis*, although ath-miR773-directed cleavage of MET2 could not be validated [95]. Interestingly, in pepper and tomato, we were able to validate miRNA-directed cleavage of DRM methyltransferse, an ortholog of the mammalian methyltransferase DNMT3, which is responsible for the *de novo* methylation in all sequence contexts [96], [97]. In higher eukaryotes, DNA methylation is a conserved epigenetic mechanism which plays a vital role in chromatin organization [97]. Another layer of complexity in gene regulation is added by the fact that DNA methylation in plants is reflected in the complicated interplay between DNA methylation and RNAi as well as histone methylation.

Furthermore, transcriptional gene silencing via RNA-directed DNA methylation (RdDM), in which DNA with sequence identity to heterochromatin siRNA is *de novo* methylated at its cytosine residues, is also present in plants [96], [98]. Previous studies have reported that DRM methyltransferase is not only required for *de novo* cytosine methylation but also for establishment of RdDM [53], [96] whose main function is to suppress the proliferation of transposons to maintain genome stability. Given the importance of DRM methyltransferase as a component of RdDM, our results raise an intriguing possibility that some miRNAs in pepper may be involved in silencing of transposons and other repetitive DNA elements. Most regions of the pepper genome are very rich in constitutive heterochromatin which consists mostly of repetitive DNA sequences and transposable elements [14], [99], [100]. Through our high-throughput sequencing data, we found that 24 nt siRNAs, which can be produced from heterochromatin repetitive sequences, account for about half of the small RNA transcriptome in pepper (See figure 1). Such a high percentage of 24 nt siRNAs, which are known to be involved in the RdDM pathway and histone modification of repetitive sequences and transposable elements [101], [102] as well as miRNA involvement in DRM methyltransferase, might altogether reflect the functional complexity of the pepper genome.

## Conclusion

In summary, this is the first study to identify conserved and non-conserved miRNAs in pepper, to analyze their expression in different tissues and to predict their targets, many of which were experimentally validated. Among them, our new finding of miR396-directed cleavage of DRM methyltransferase opens a new avenue in the field of epigenetic regulation in the complex pepper genome. Using high-throughput sequencing approaches, our work is the first to provide a comprehensive view of pepper miRNAs and their targets, establishing a foundation for future studies of miRNAs in pepper and Solanaceae.

## Supporting Information

Dataset S1 Full list of hairpin structures in conserved miRNAs.
(ZIP)

Dataset S2 Full list of hairpin structures in novel miRNAs.
(ZIP)

Figure S1 T-coffee alignment of DRM methyltransferase.
(JPG)

Figure S2 Phylogenetic tree of DRM methyltransferase among several plant species.
(EPS)

Figure S3 miR396 targeting DRM methyltransferase in Solanaceae.
(EPS)

Table S1 Predicted targets of conserved miRNAs.
(XLSX)

Table S2 Probe sequences used for northern blot analysis.
(XLSX)

Table S3 Gene sepcific primers used for 5 RACE.
(XLSX)

Table S4 5RACE results of novel miRNA targets.
(XLSX)

Table S5 Contig numbers of conserved miRNAs.
(XLSX)

Table S6 Contig numbers of novel miRNAs.
(XLSX)
