# Supplementary figures and images for: The Hot Pepper (Capsicum annuum) MicroRNA Transcriptome Reveals Novel and Conserved Targets: A Foundation for Understanding MicroRNA Functional Roles in Hot Pepper (part 2 of 2)
Source: PLoS One. 2013 May 30;8(5):e64238. doi: 10.1371/journal.pone.0064238 (PMC3667847; doi:10.1371/journal.pone.0064238)

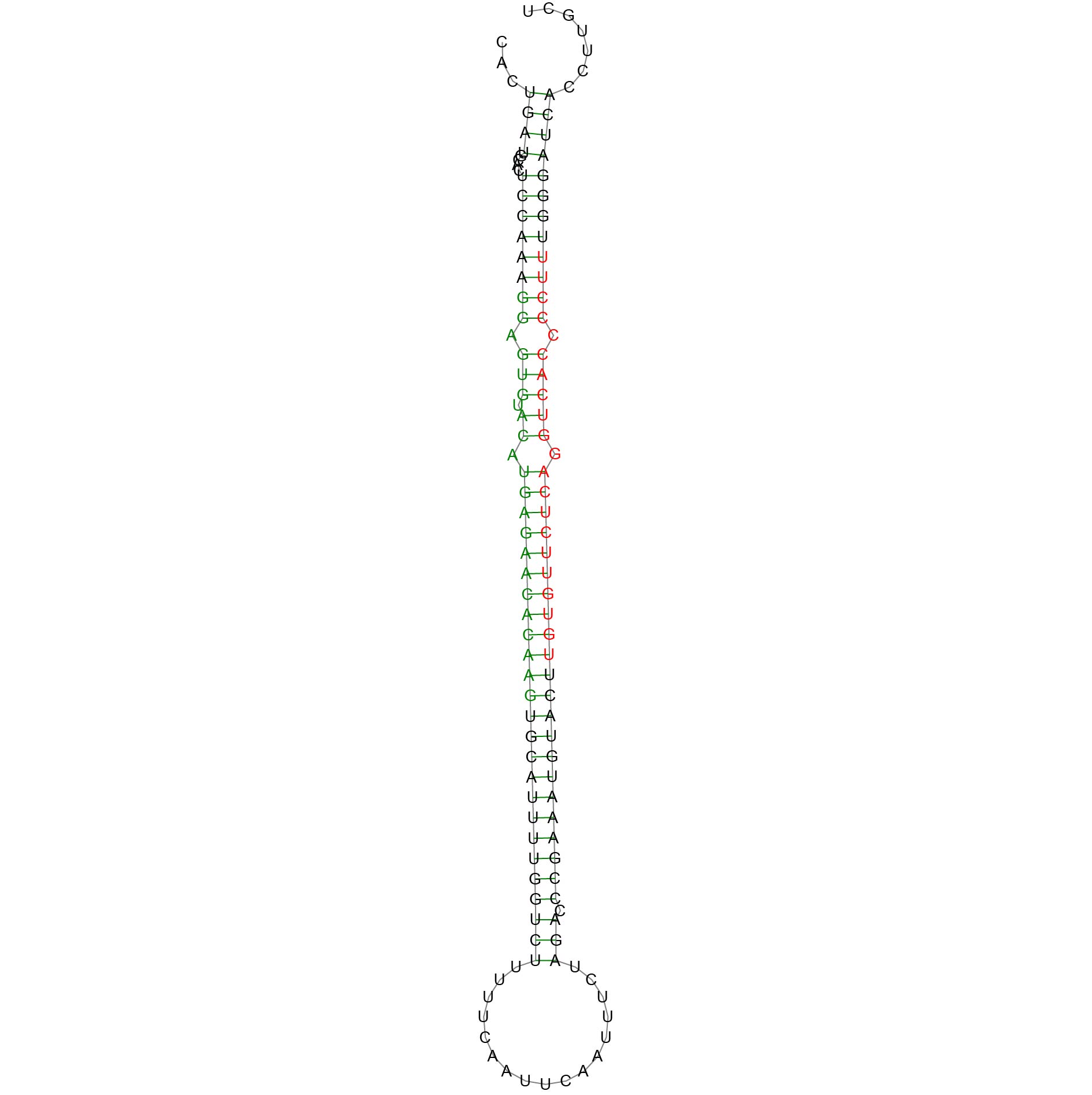

Supplement: Dataset S1 — Full list of hairpin structures in conserved miRNAs. (ZIP) [file pone.0064238.s001.zip › can-miR398a.jpg]

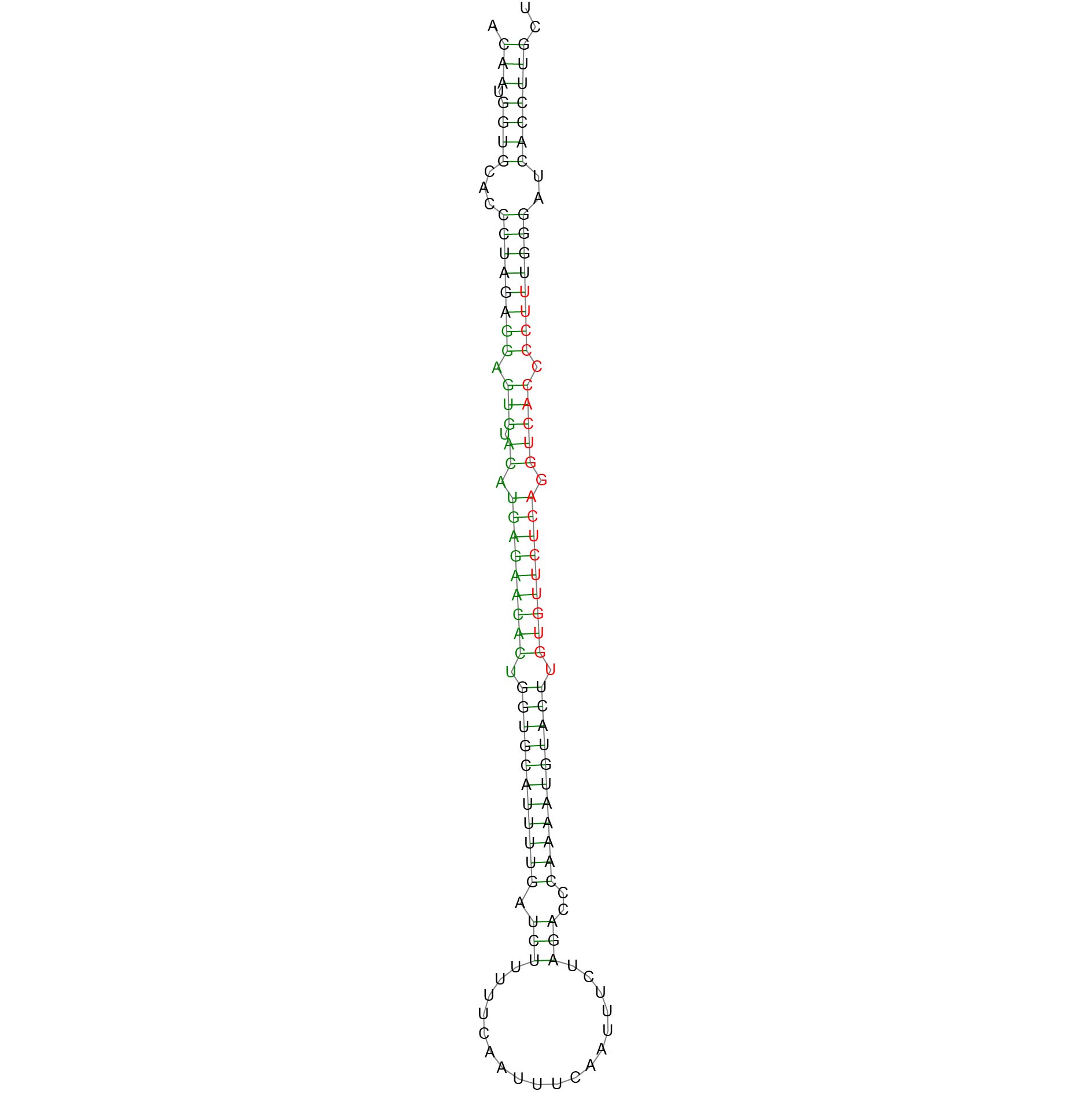

Supplement: Dataset S1 — Full list of hairpin structures in conserved miRNAs. (ZIP) [file pone.0064238.s001.zip › can-miR398b.jpg]

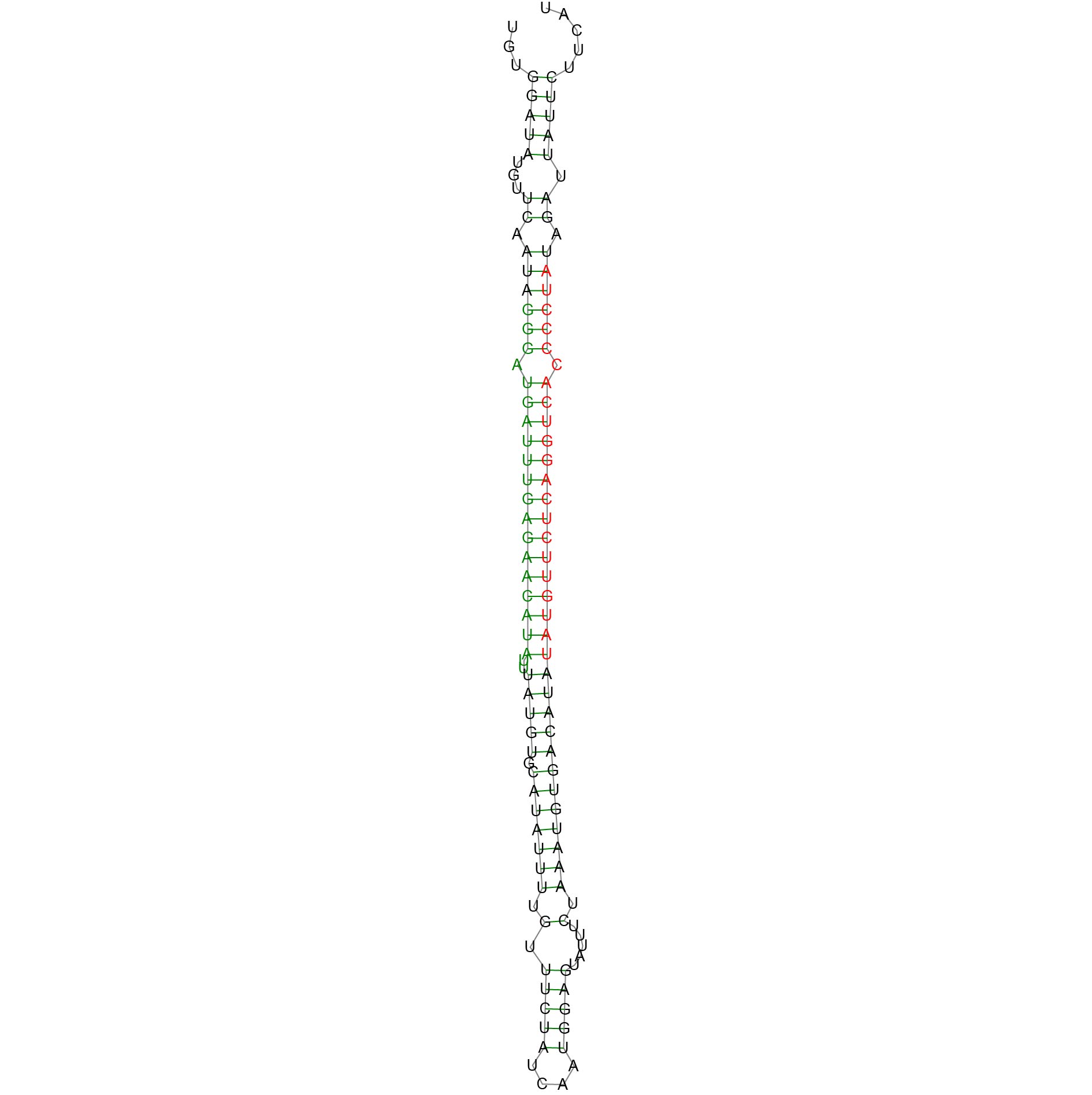

Supplement: Dataset S1 — Full list of hairpin structures in conserved miRNAs. (ZIP) [file pone.0064238.s001.zip › can-miR398c.jpg]

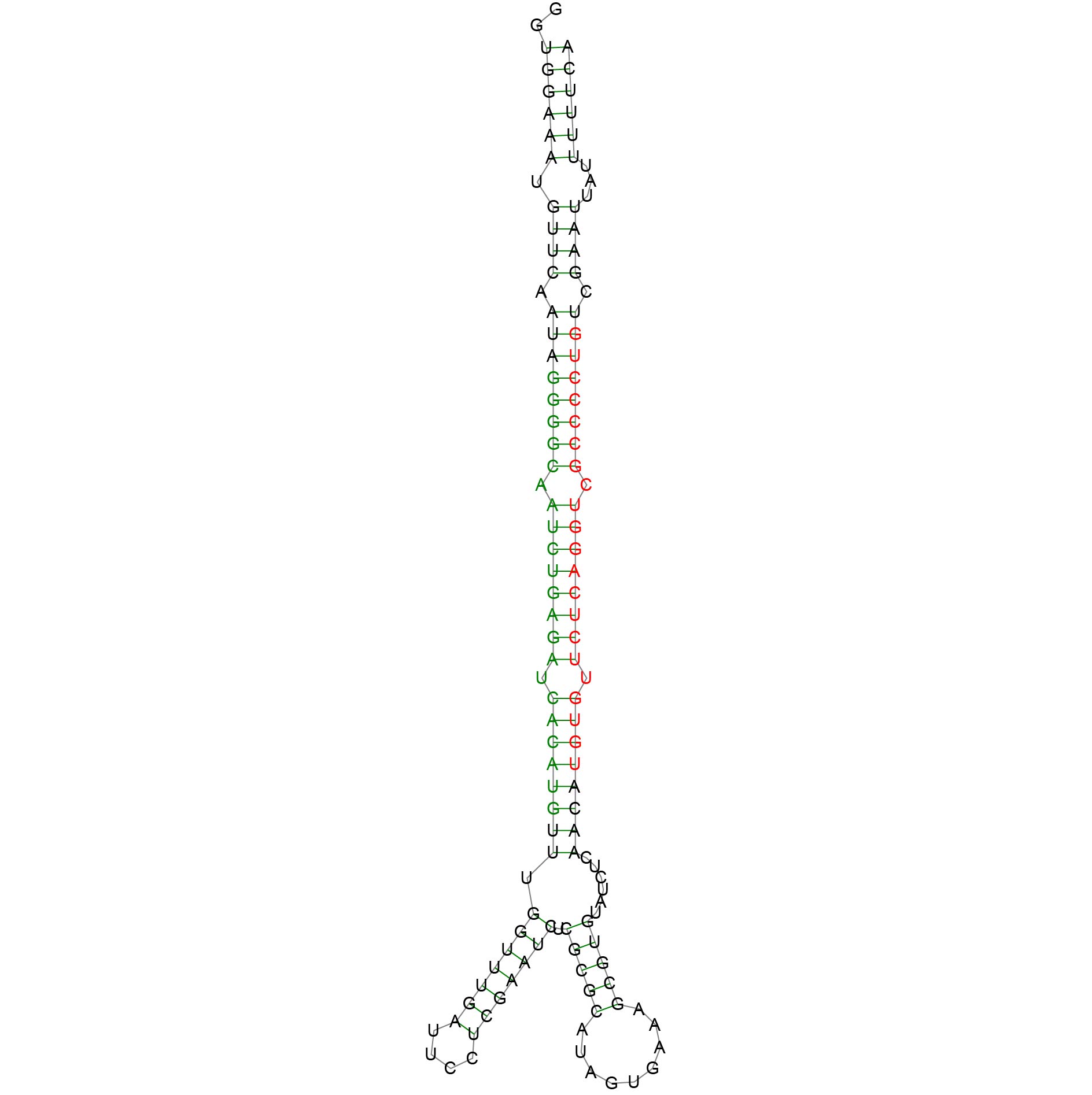

Supplement: Dataset S1 — Full list of hairpin structures in conserved miRNAs. (ZIP) [file pone.0064238.s001.zip › can-miR398d.jpg]

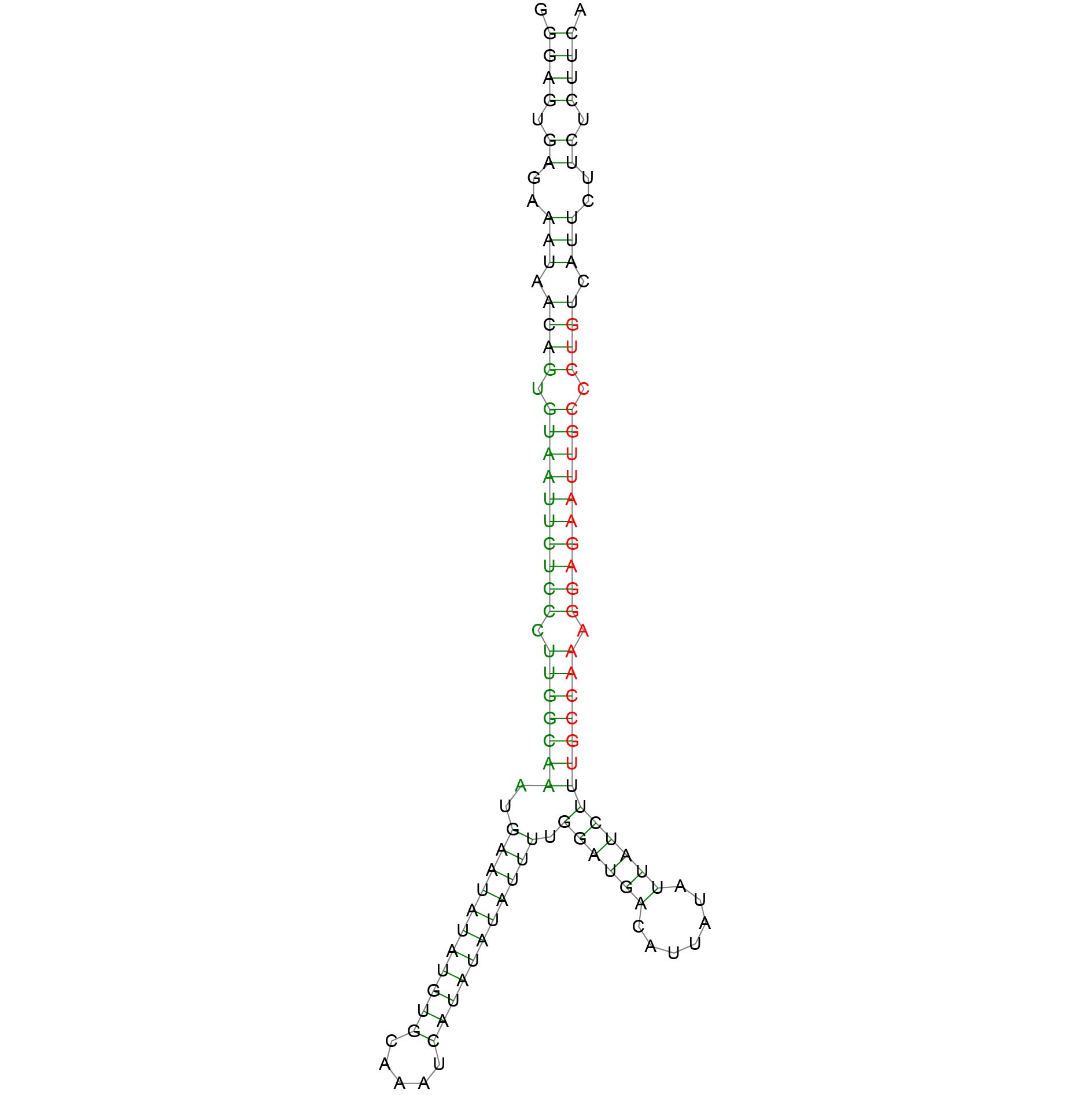

Supplement: Dataset S1 — Full list of hairpin structures in conserved miRNAs. (ZIP) [file pone.0064238.s001.zip › can-miR399a.jpg]

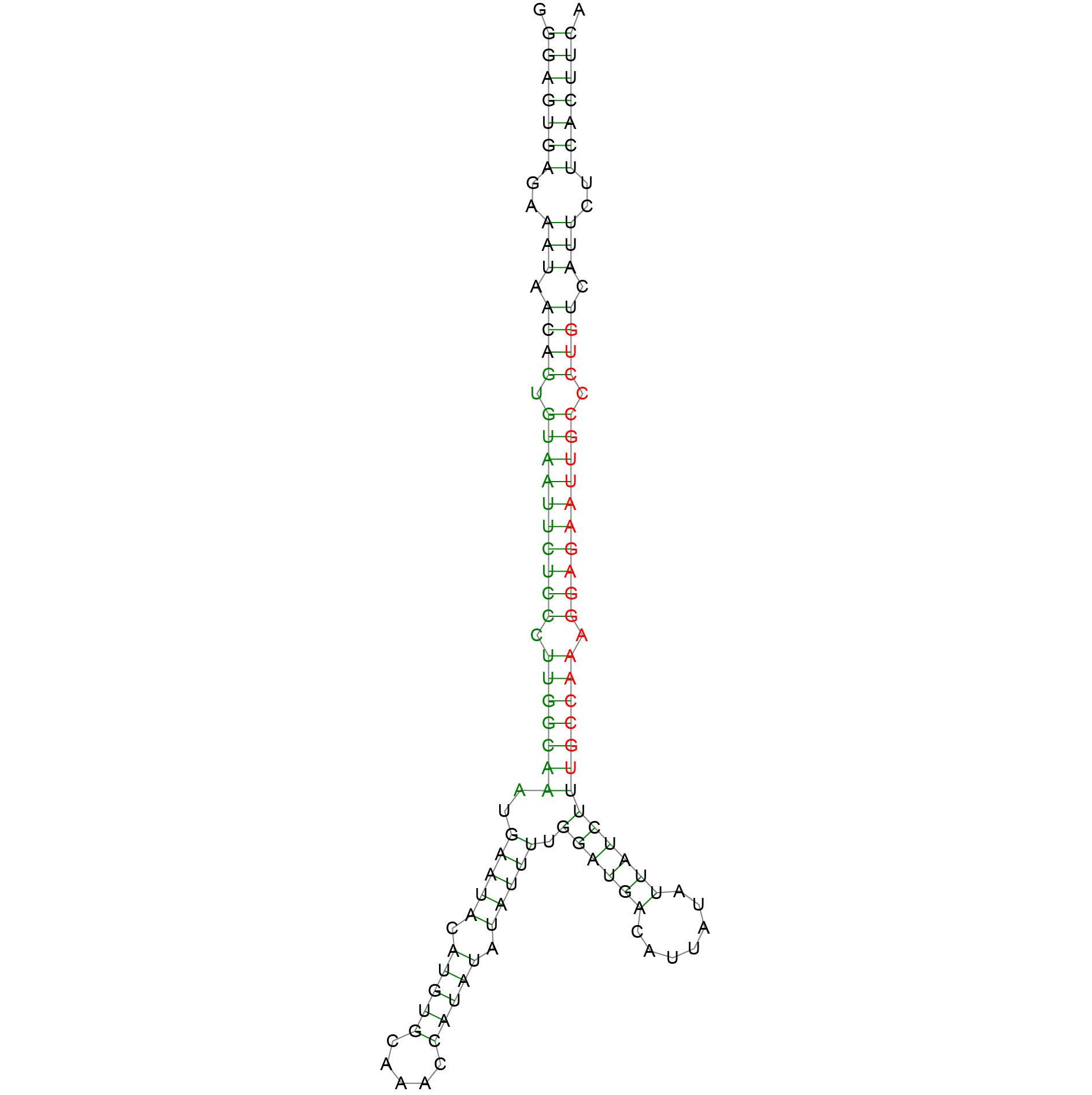

Supplement: Dataset S1 — Full list of hairpin structures in conserved miRNAs. (ZIP) [file pone.0064238.s001.zip › can-miR399b.jpg]

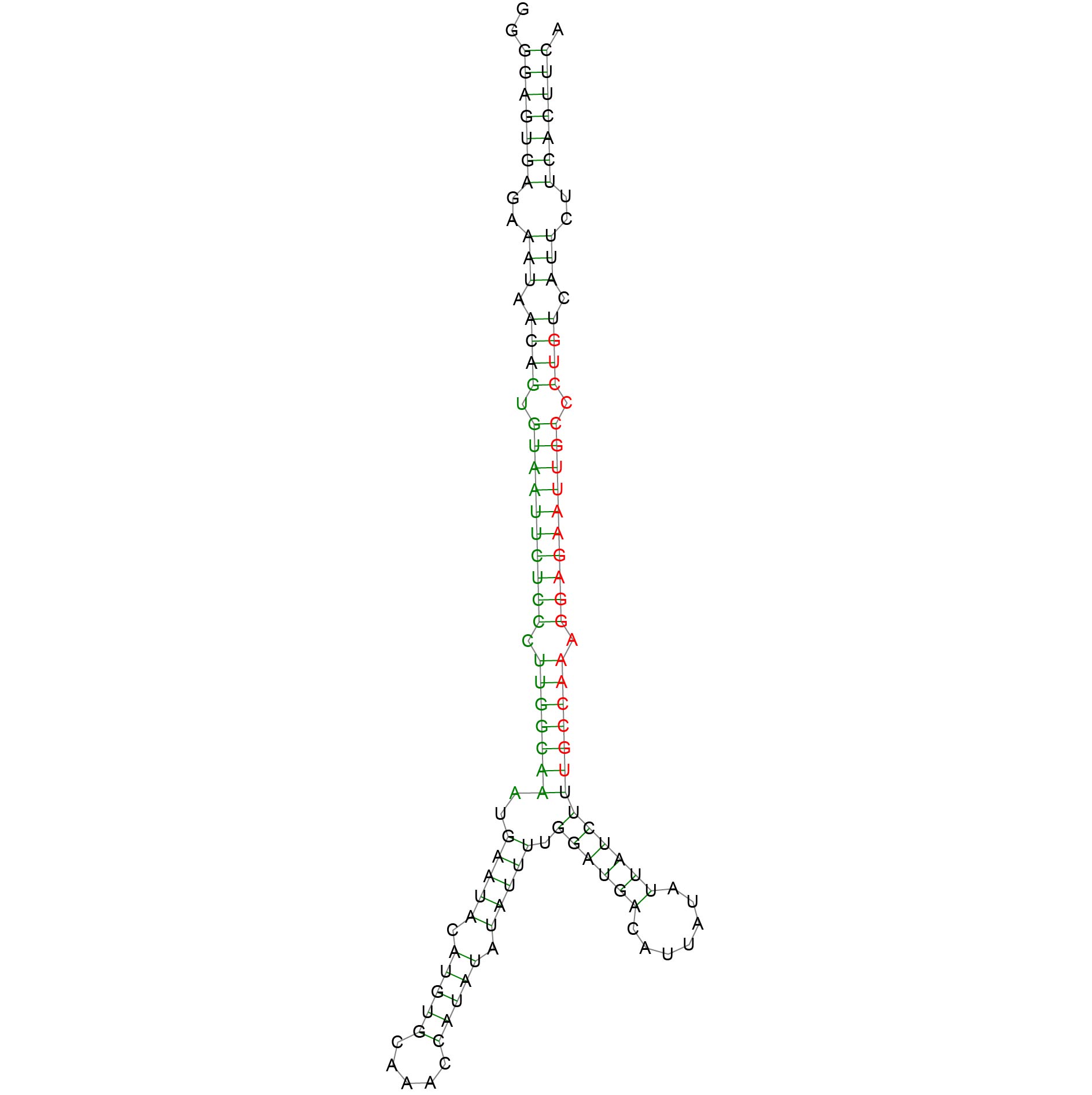

Supplement: Dataset S1 — Full list of hairpin structures in conserved miRNAs. (ZIP) [file pone.0064238.s001.zip › can-miR399c.jpg]

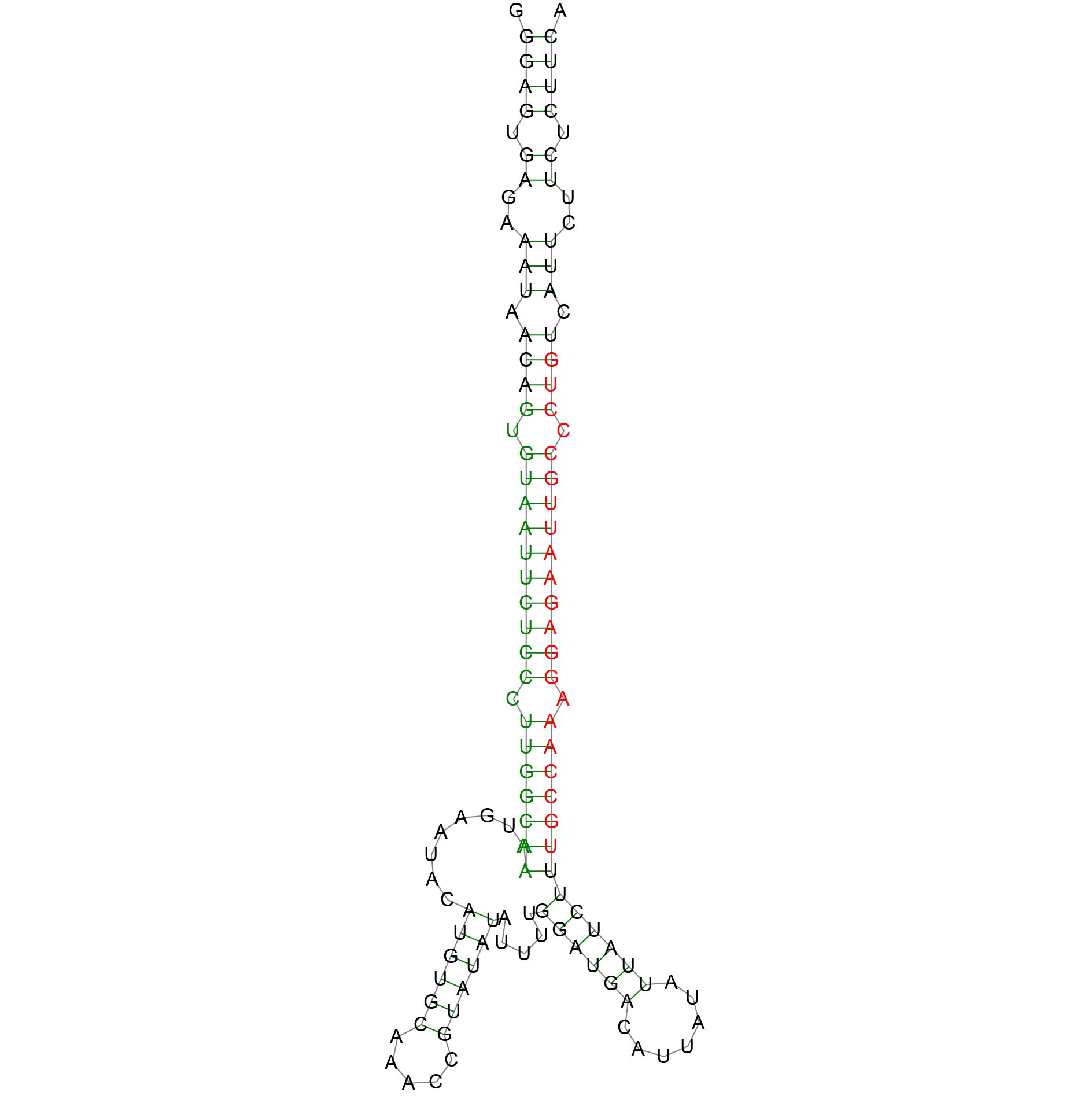

Supplement: Dataset S1 — Full list of hairpin structures in conserved miRNAs. (ZIP) [file pone.0064238.s001.zip › can-miR399d.jpg]

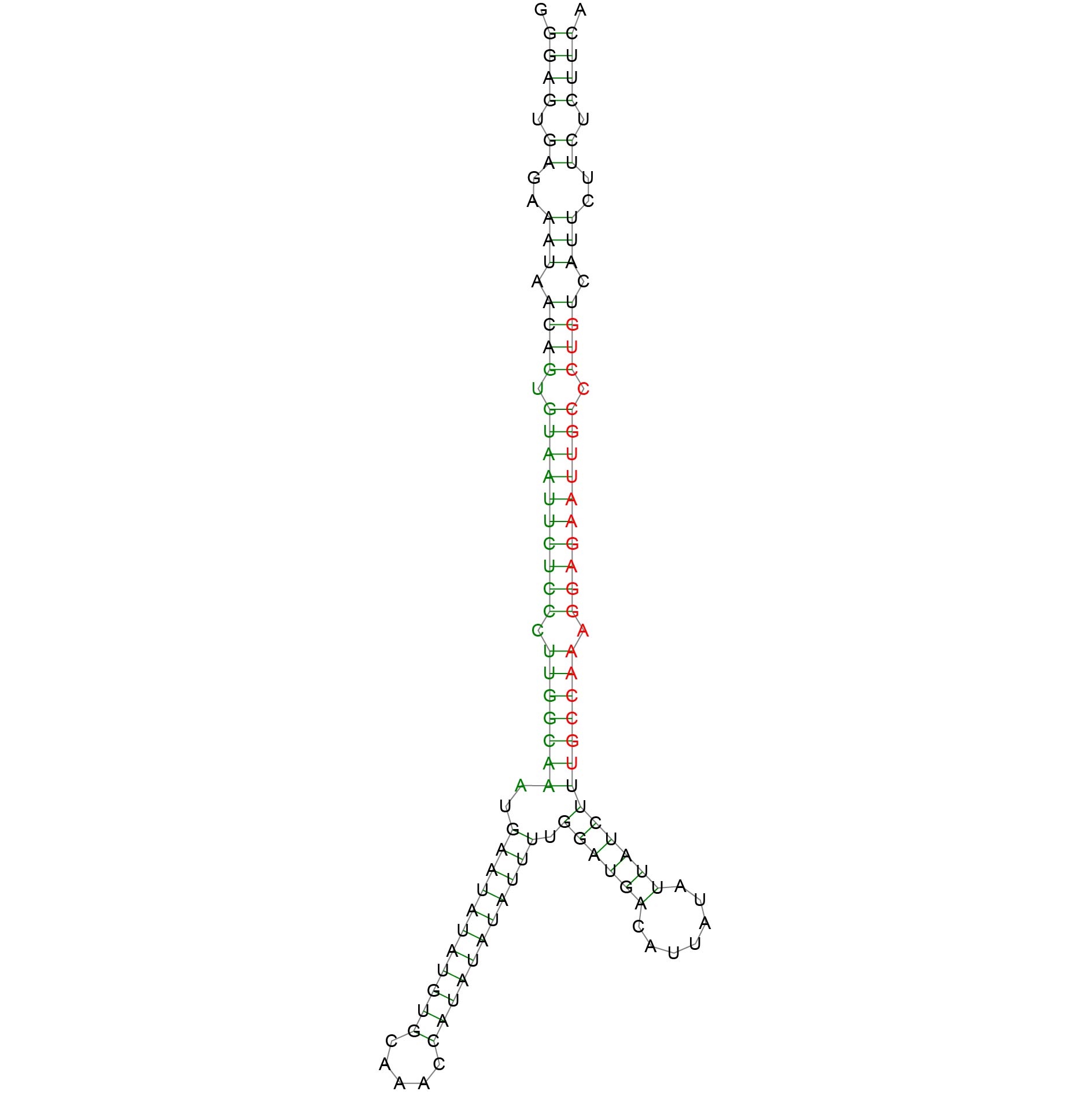

Supplement: Dataset S1 — Full list of hairpin structures in conserved miRNAs. (ZIP) [file pone.0064238.s001.zip › can-miR399e.jpg]

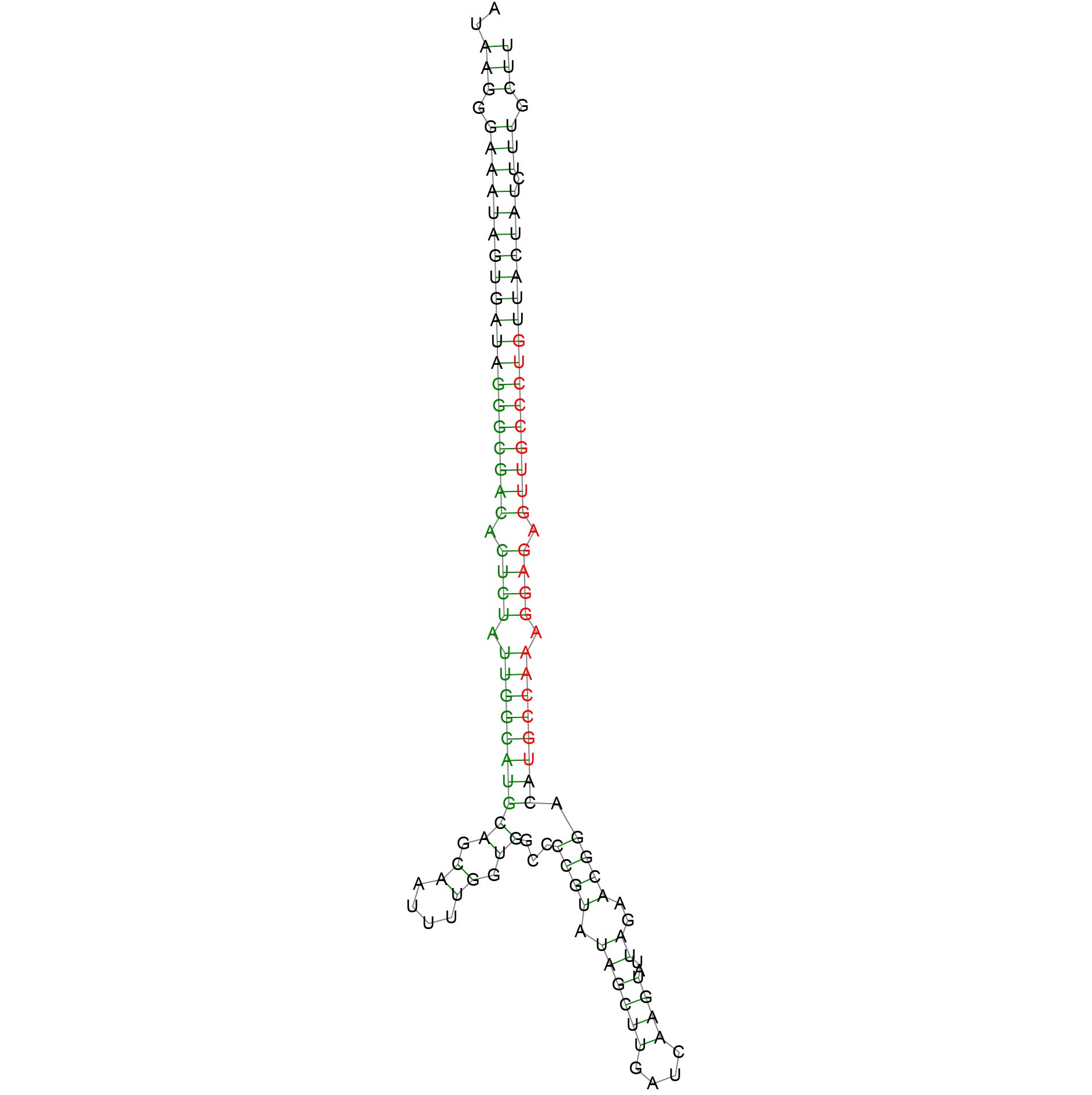

Supplement: Dataset S1 — Full list of hairpin structures in conserved miRNAs. (ZIP) [file pone.0064238.s001.zip › can-miR399f.jpg]

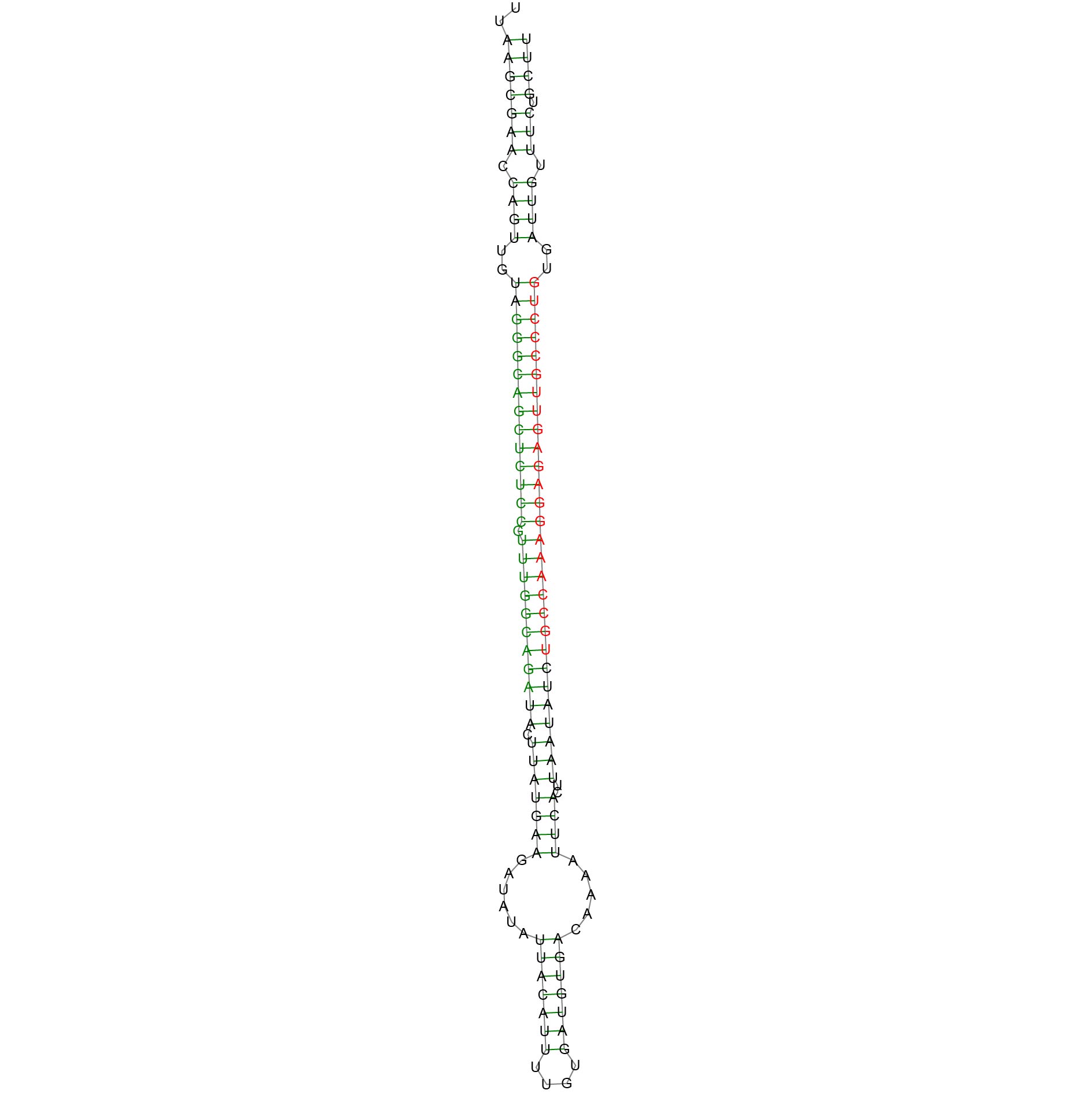

Supplement: Dataset S1 — Full list of hairpin structures in conserved miRNAs. (ZIP) [file pone.0064238.s001.zip › can-miR399g.jpg]

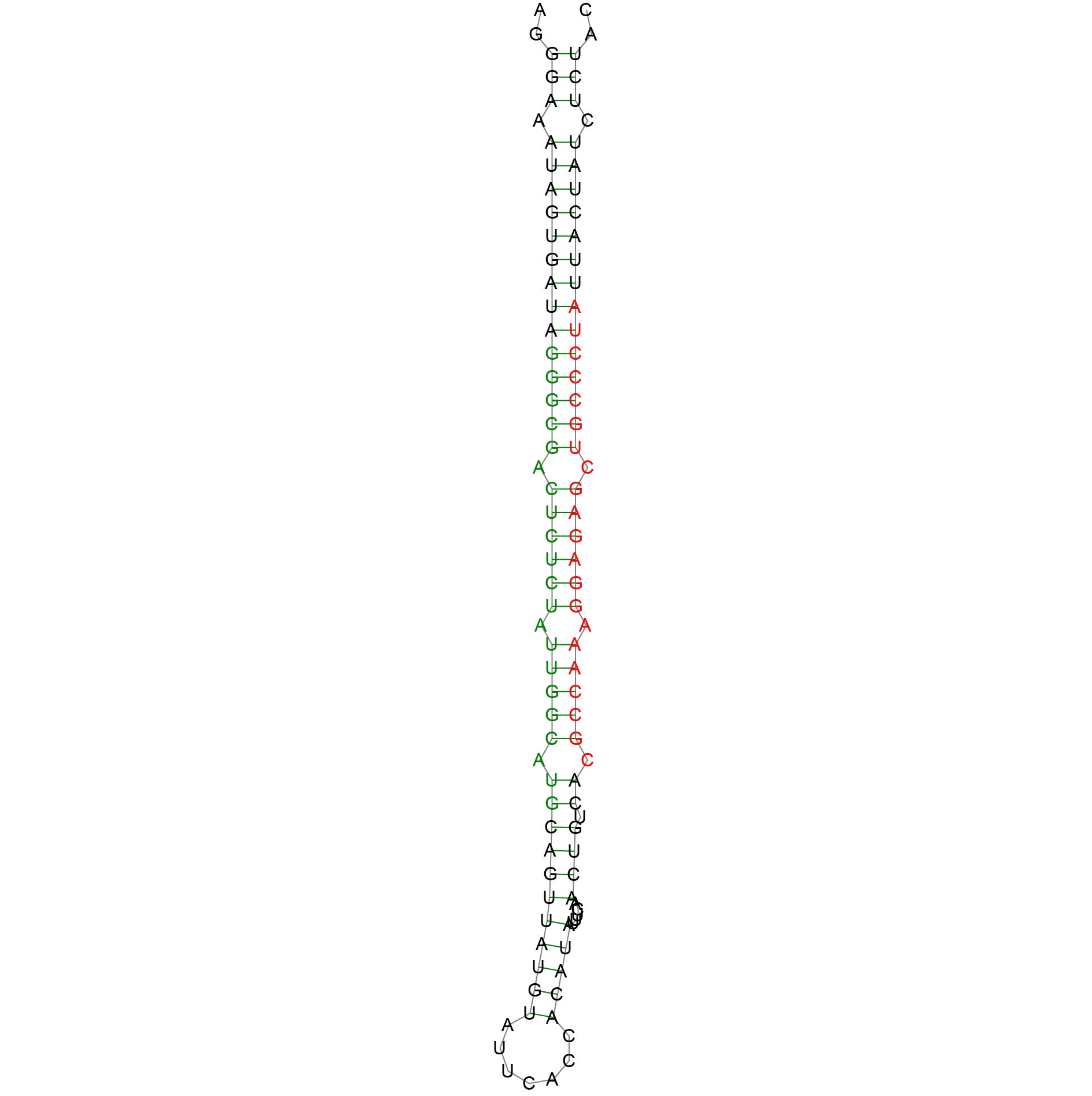

Supplement: Dataset S1 — Full list of hairpin structures in conserved miRNAs. (ZIP) [file pone.0064238.s001.zip › can-miR399h.jpg]

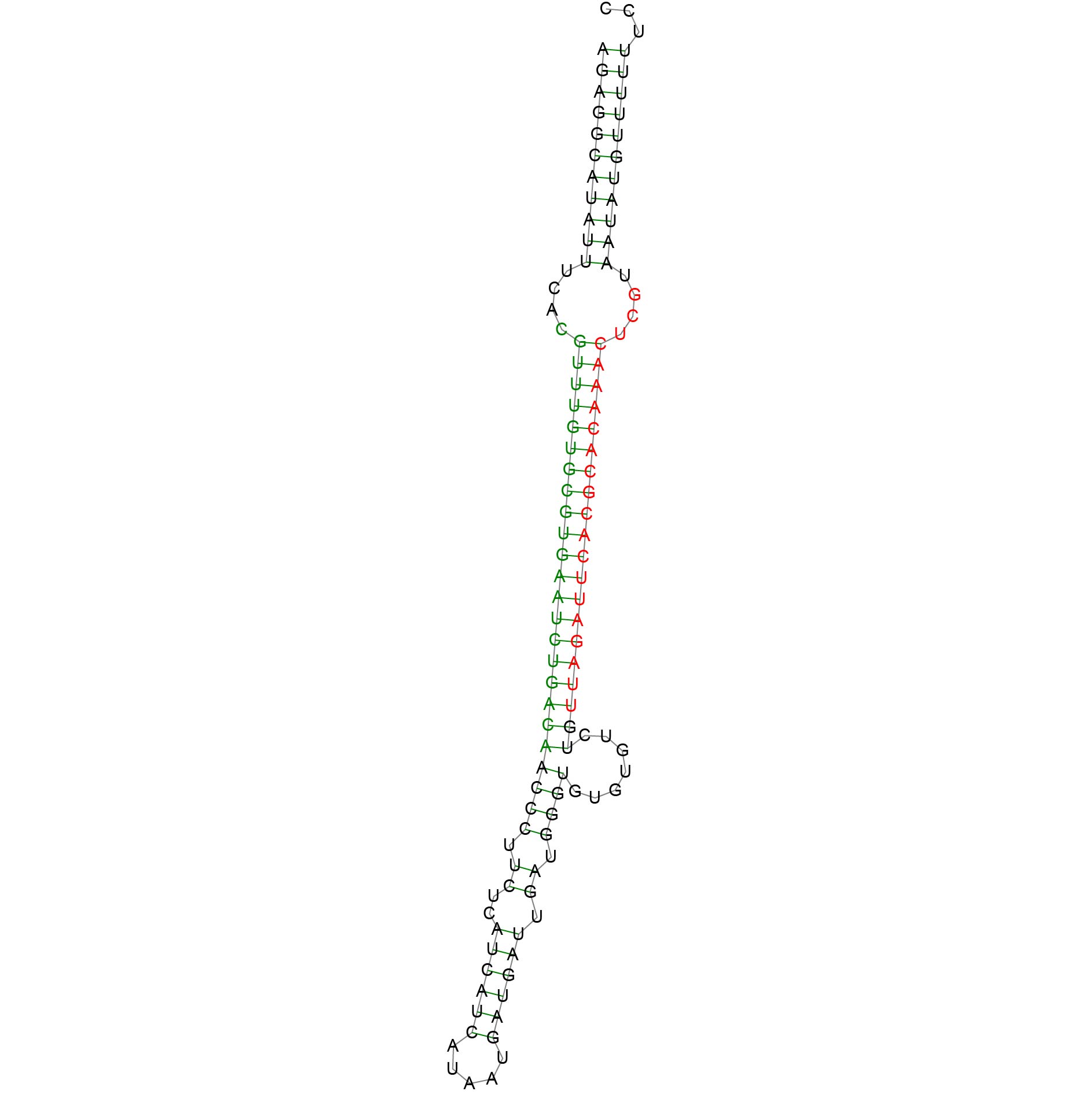

Supplement: Dataset S1 — Full list of hairpin structures in conserved miRNAs. (ZIP) [file pone.0064238.s001.zip › can-miR403.jpg]

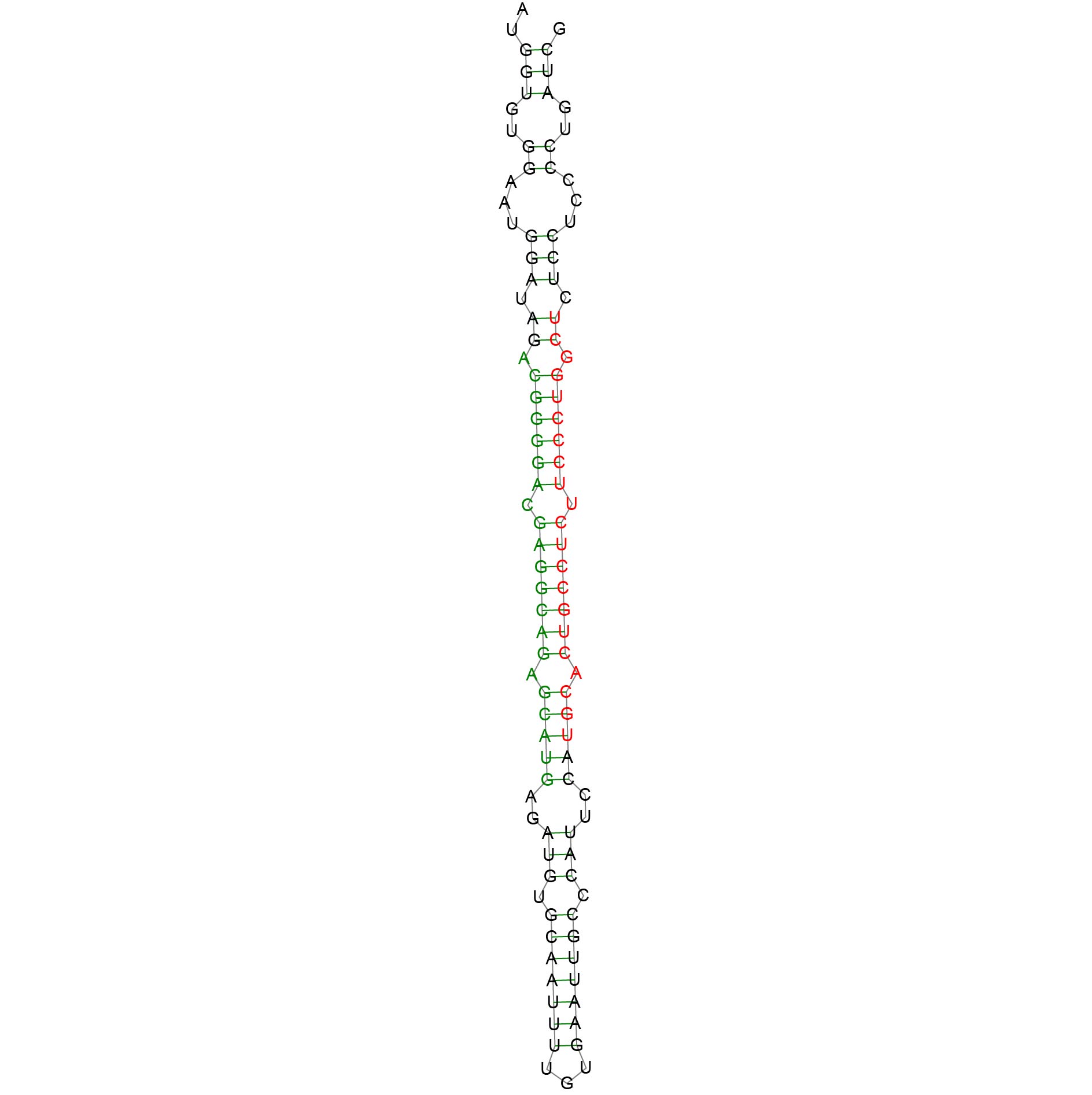

Supplement: Dataset S1 — Full list of hairpin structures in conserved miRNAs. (ZIP) [file pone.0064238.s001.zip › can-miR408a.jpg]

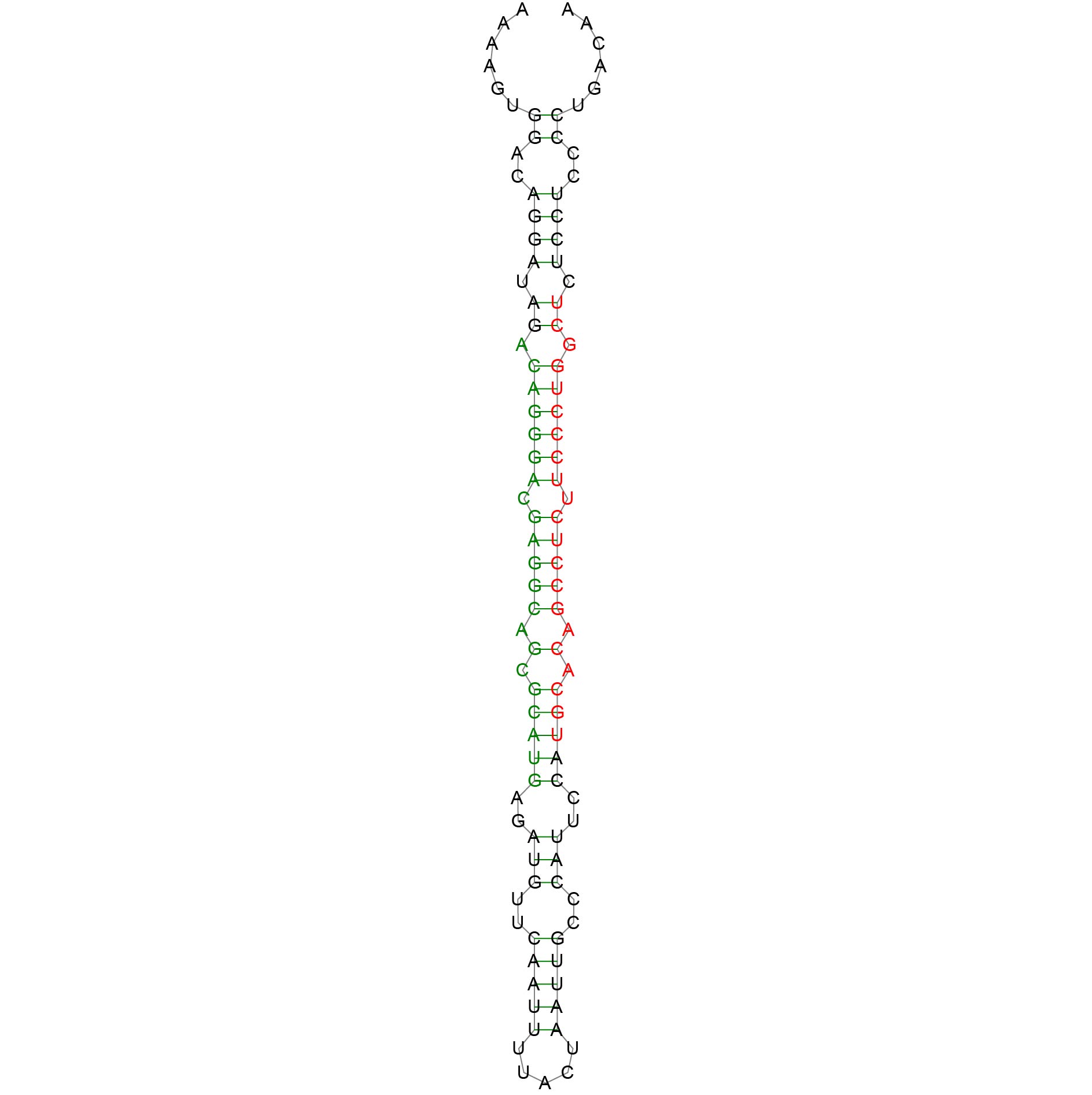

Supplement: Dataset S1 — Full list of hairpin structures in conserved miRNAs. (ZIP) [file pone.0064238.s001.zip › can-miR408b.jpg]

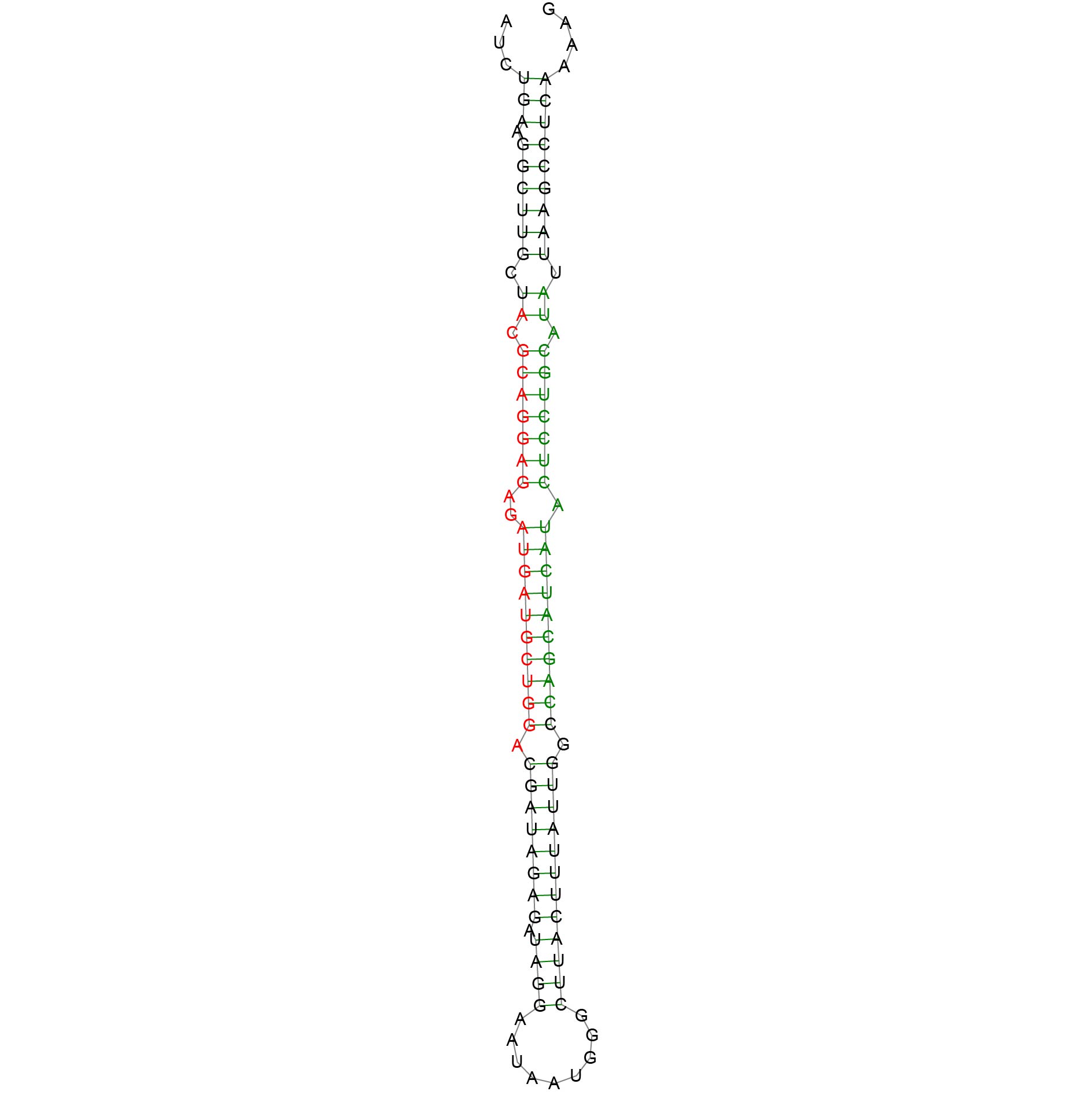

Supplement: Dataset S1 — Full list of hairpin structures in conserved miRNAs. (ZIP) [file pone.0064238.s001.zip › can-miR4376.jpg]

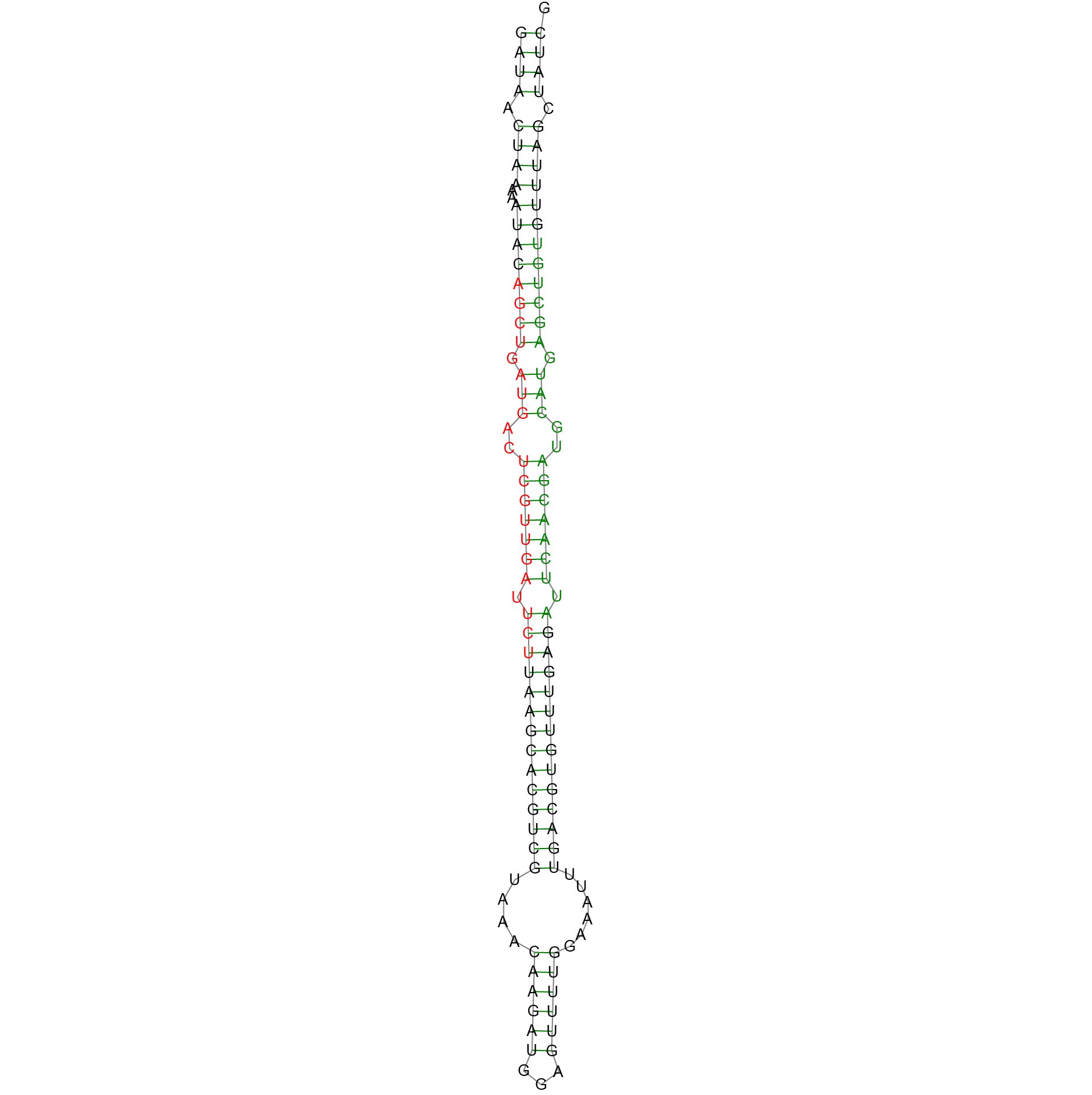

Supplement: Dataset S1 — Full list of hairpin structures in conserved miRNAs. (ZIP) [file pone.0064238.s001.zip › can-miR4414a.jpg]

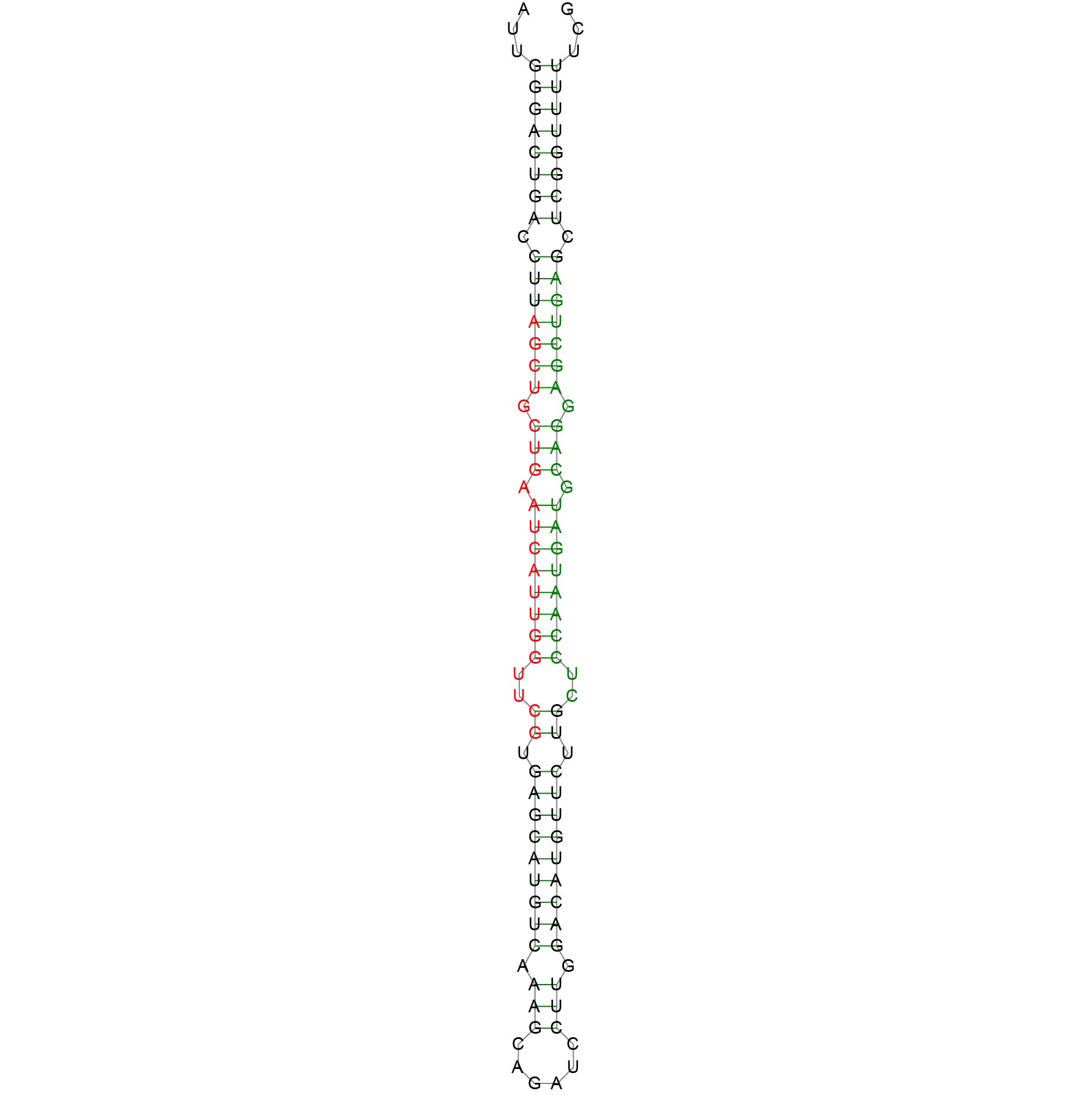

Supplement: Dataset S1 — Full list of hairpin structures in conserved miRNAs. (ZIP) [file pone.0064238.s001.zip › can-miR4414b.jpg]

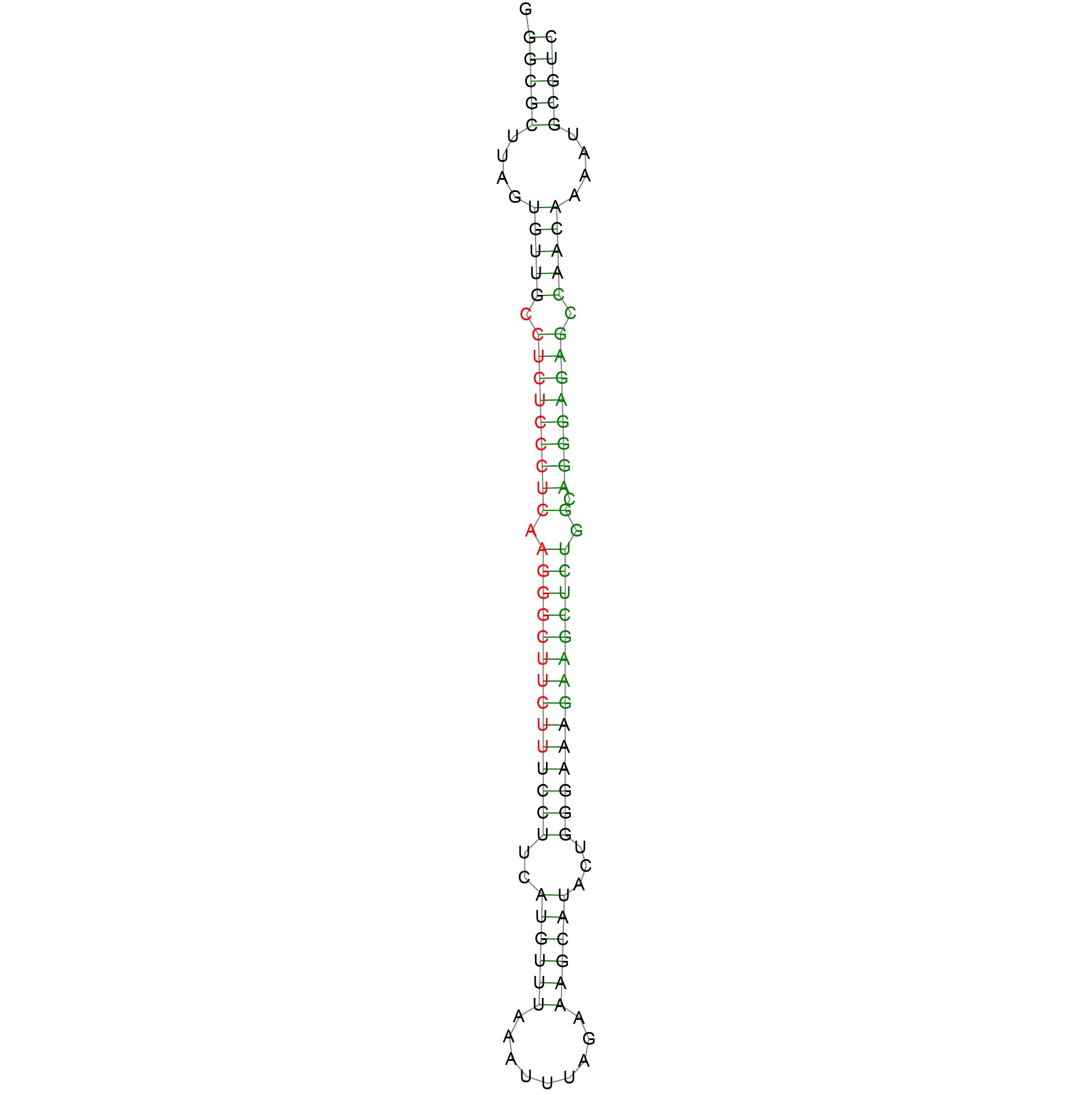

Supplement: Dataset S1 — Full list of hairpin structures in conserved miRNAs. (ZIP) [file pone.0064238.s001.zip › can-miR477.jpg]

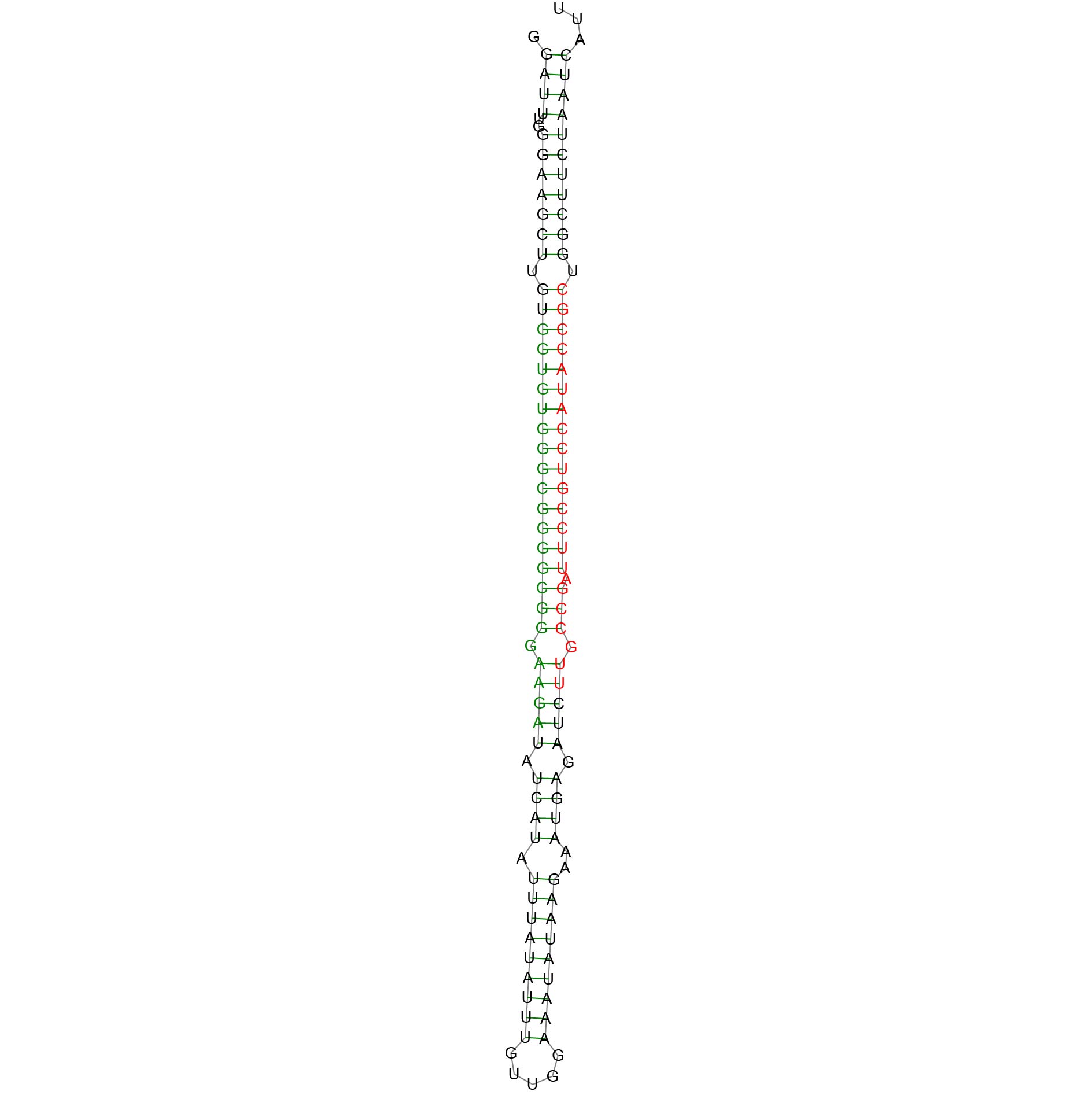

Supplement: Dataset S1 — Full list of hairpin structures in conserved miRNAs. (ZIP) [file pone.0064238.s001.zip › can-miR482a.jpg]

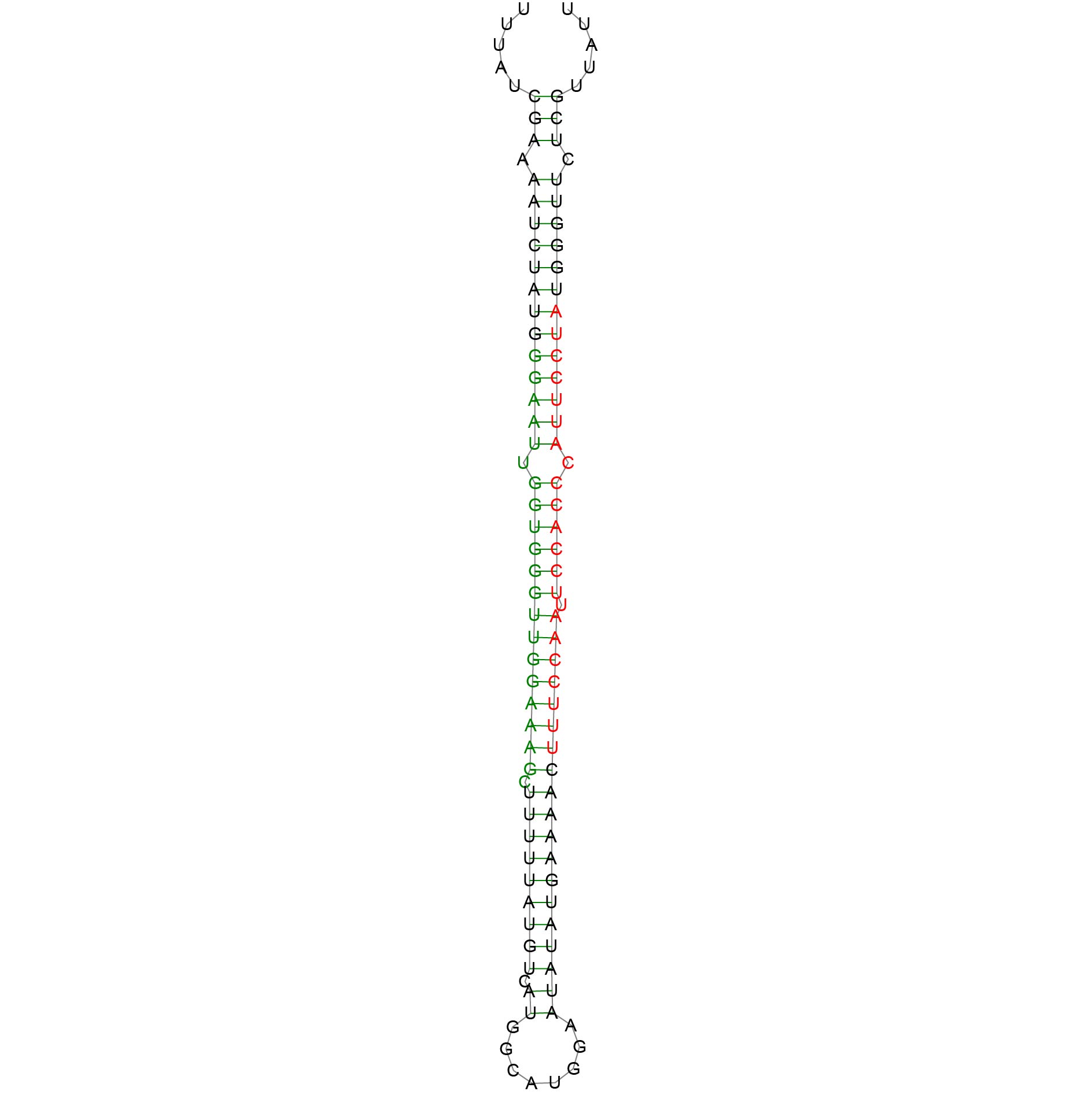

Supplement: Dataset S1 — Full list of hairpin structures in conserved miRNAs. (ZIP) [file pone.0064238.s001.zip › can-miR482b.jpg]

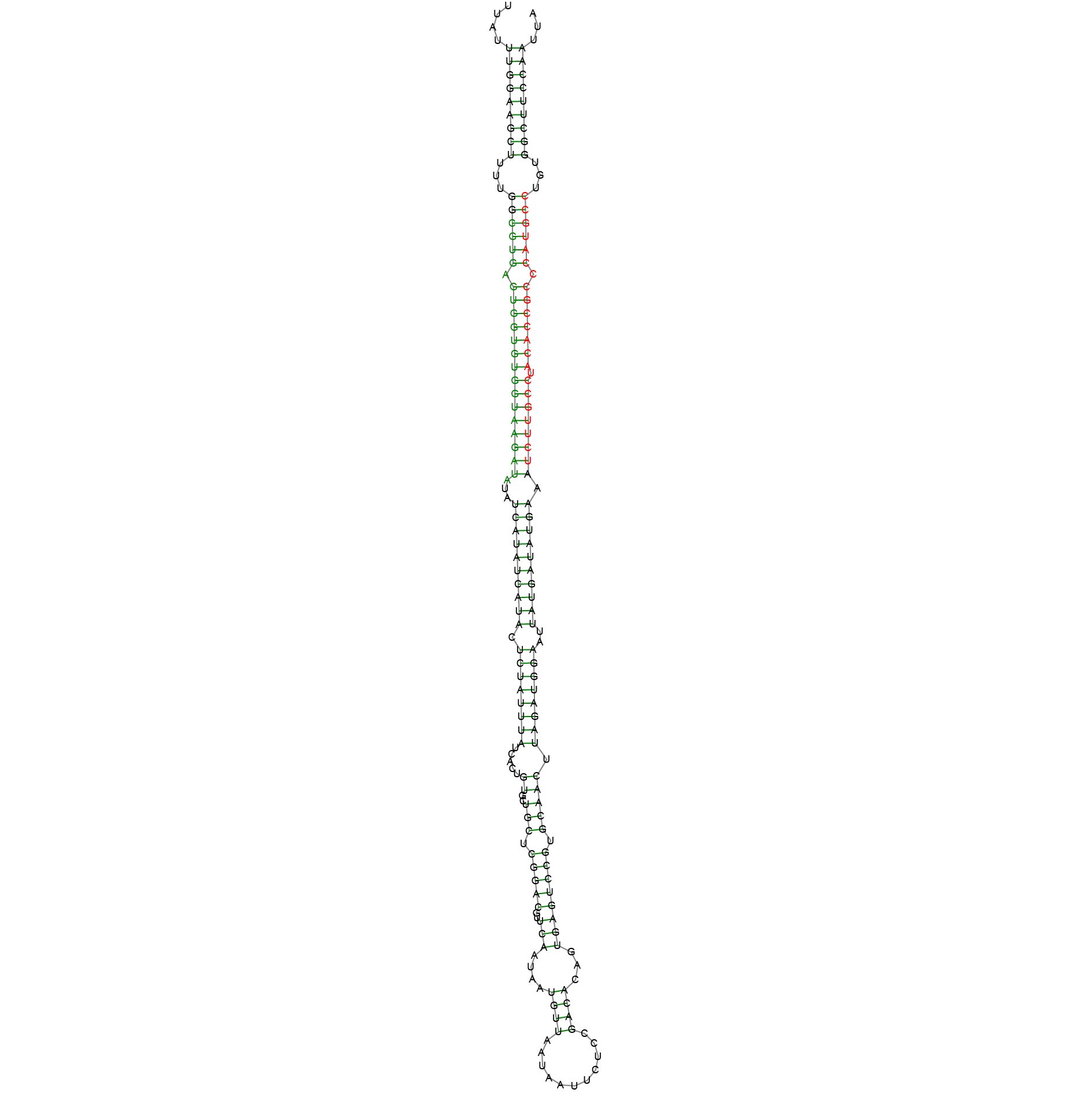

Supplement: Dataset S1 — Full list of hairpin structures in conserved miRNAs. (ZIP) [file pone.0064238.s001.zip › can-miR482c.jpg]

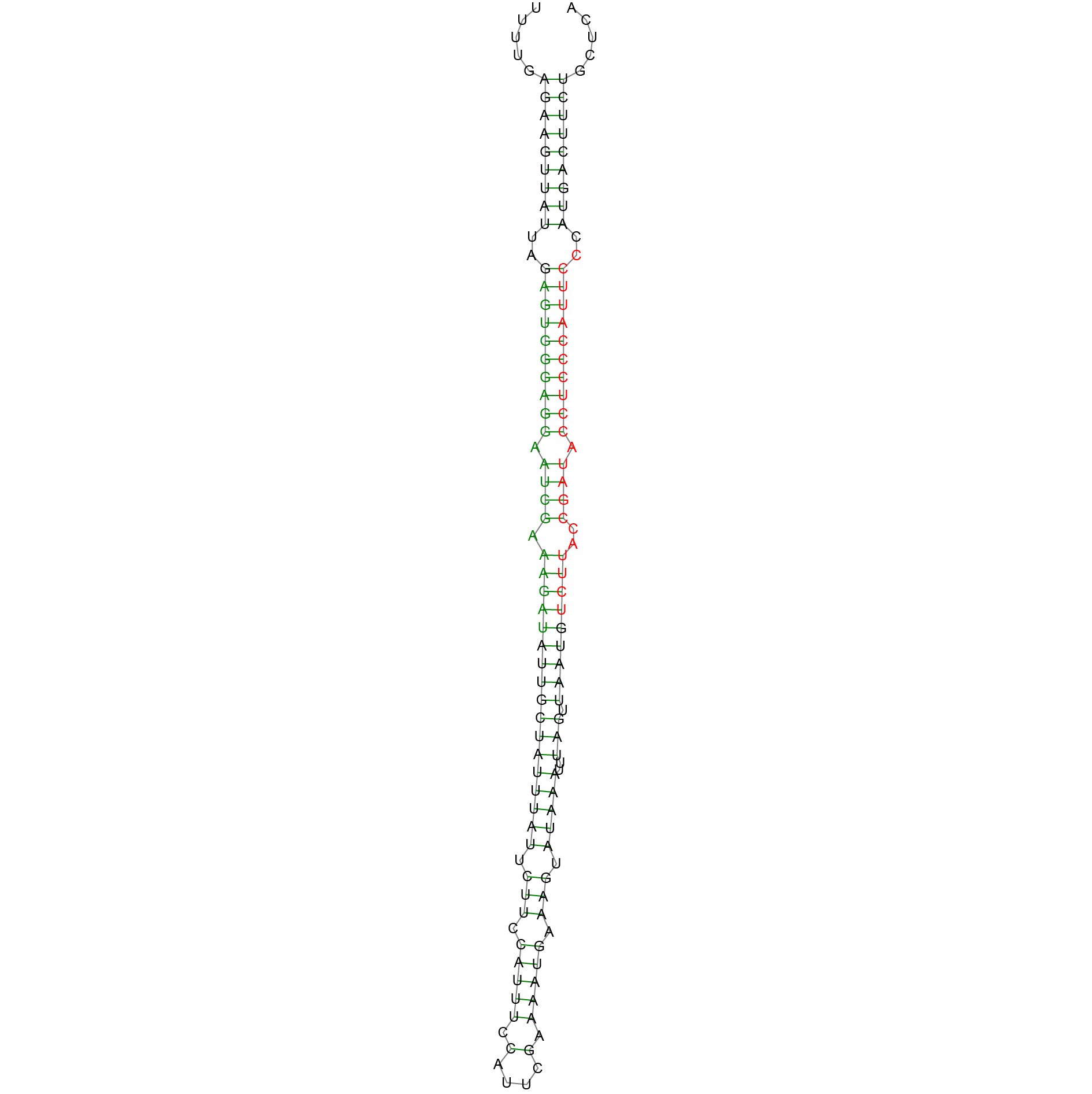

Supplement: Dataset S1 — Full list of hairpin structures in conserved miRNAs. (ZIP) [file pone.0064238.s001.zip › can-miR482d.jpg]

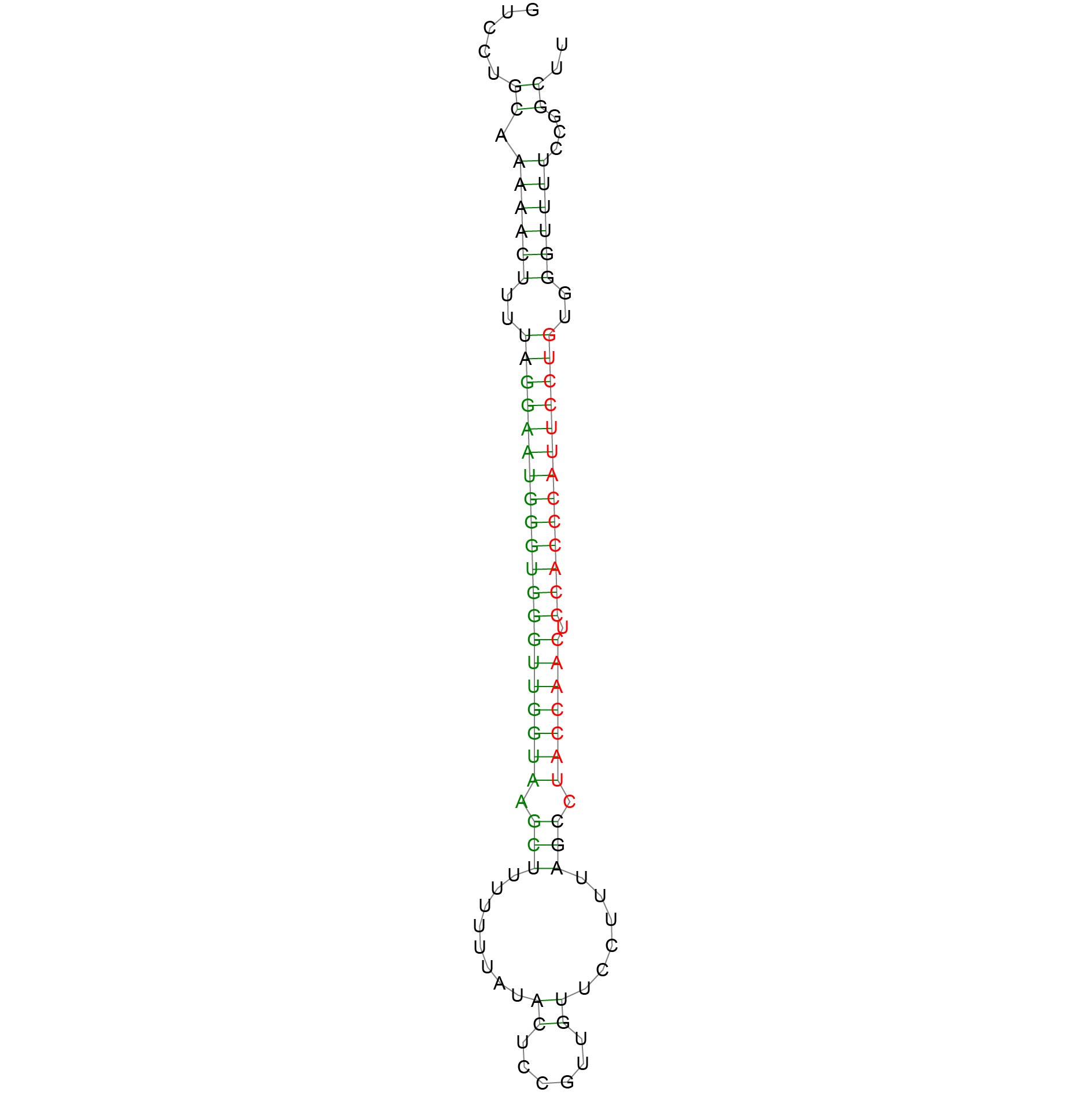

Supplement: Dataset S1 — Full list of hairpin structures in conserved miRNAs. (ZIP) [file pone.0064238.s001.zip › can-miR482e.jpg]

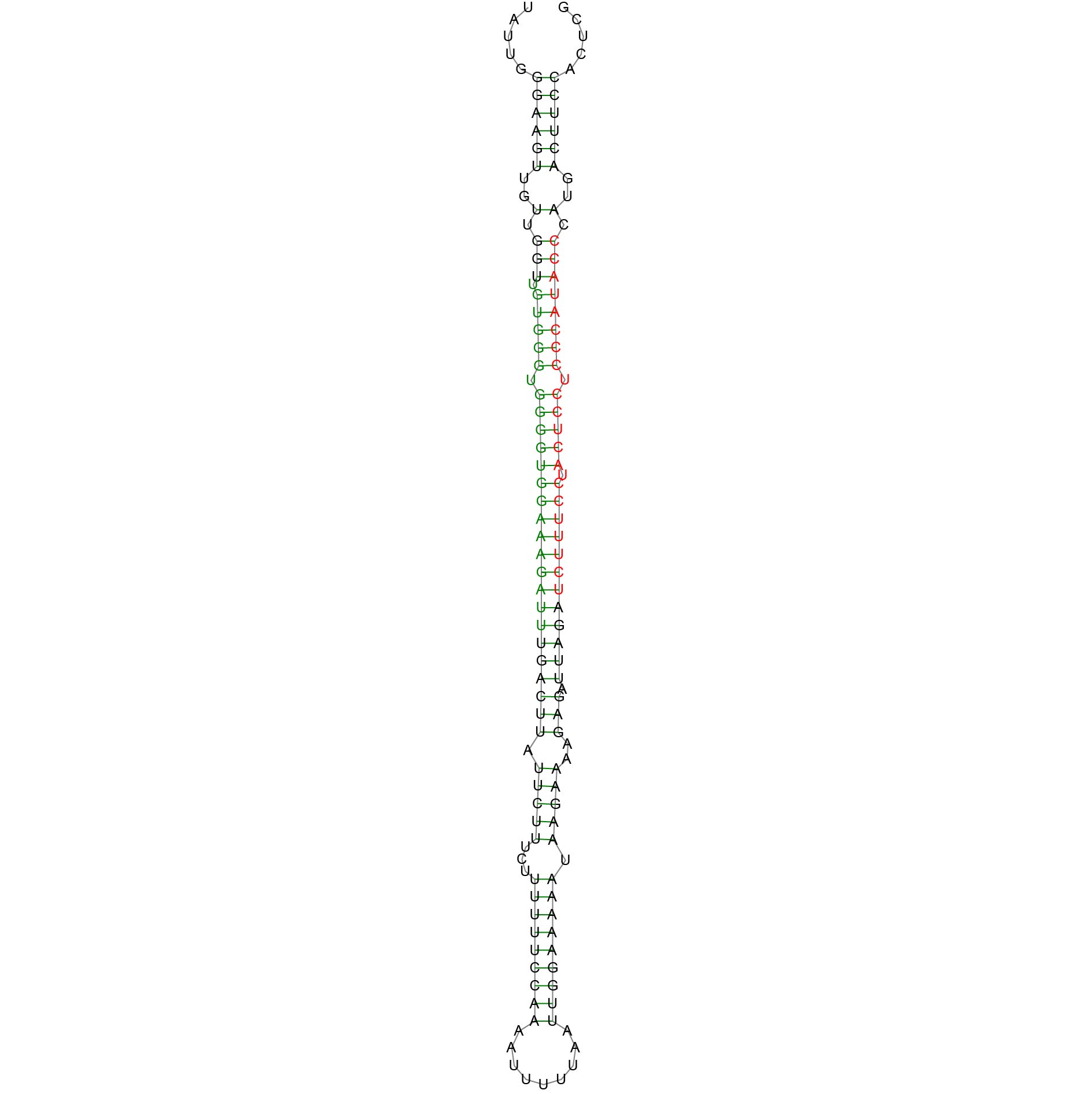

Supplement: Dataset S1 — Full list of hairpin structures in conserved miRNAs. (ZIP) [file pone.0064238.s001.zip › can-miR482f.jpg]

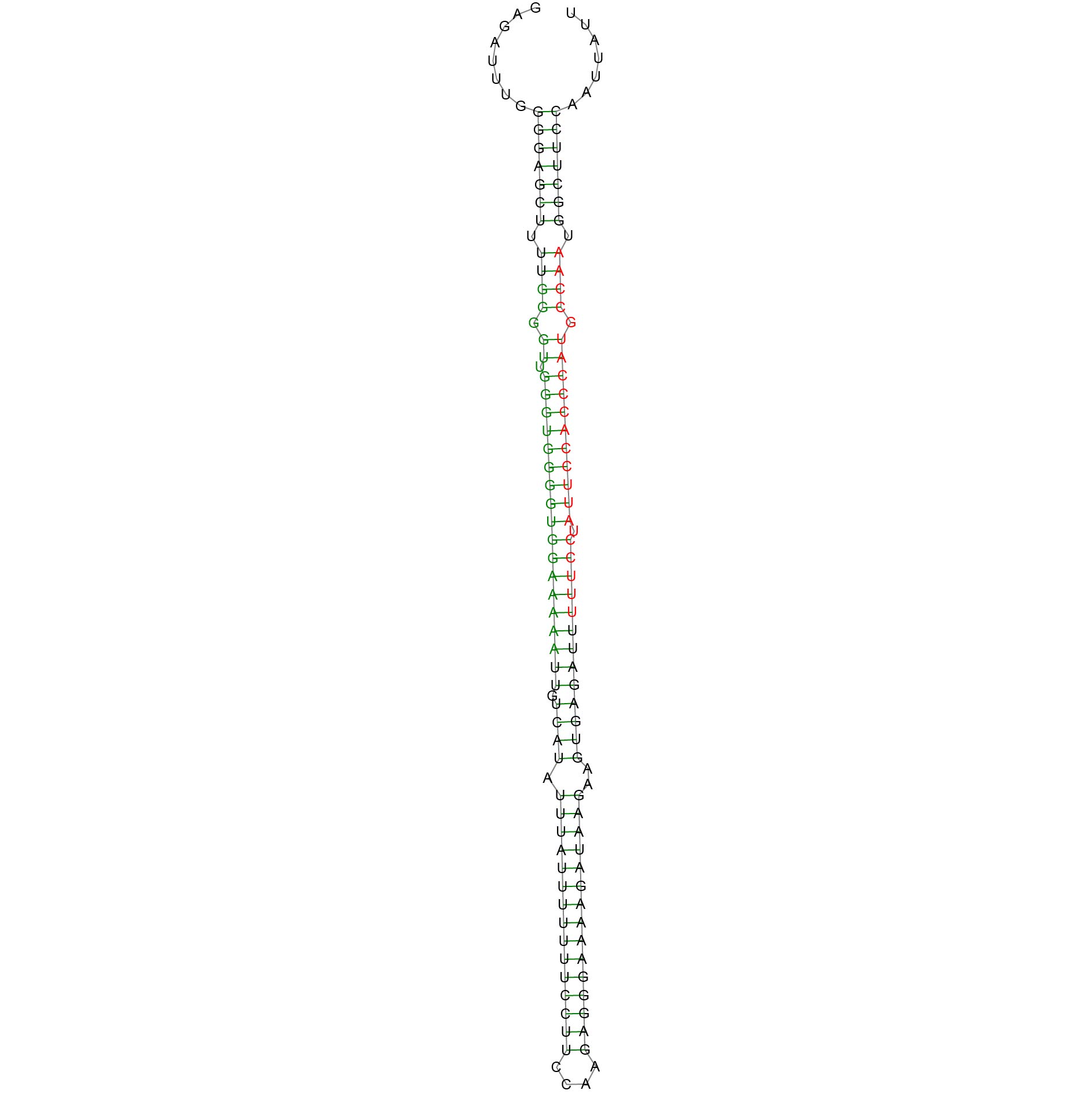

Supplement: Dataset S1 — Full list of hairpin structures in conserved miRNAs. (ZIP) [file pone.0064238.s001.zip › can-miR482g.jpg]

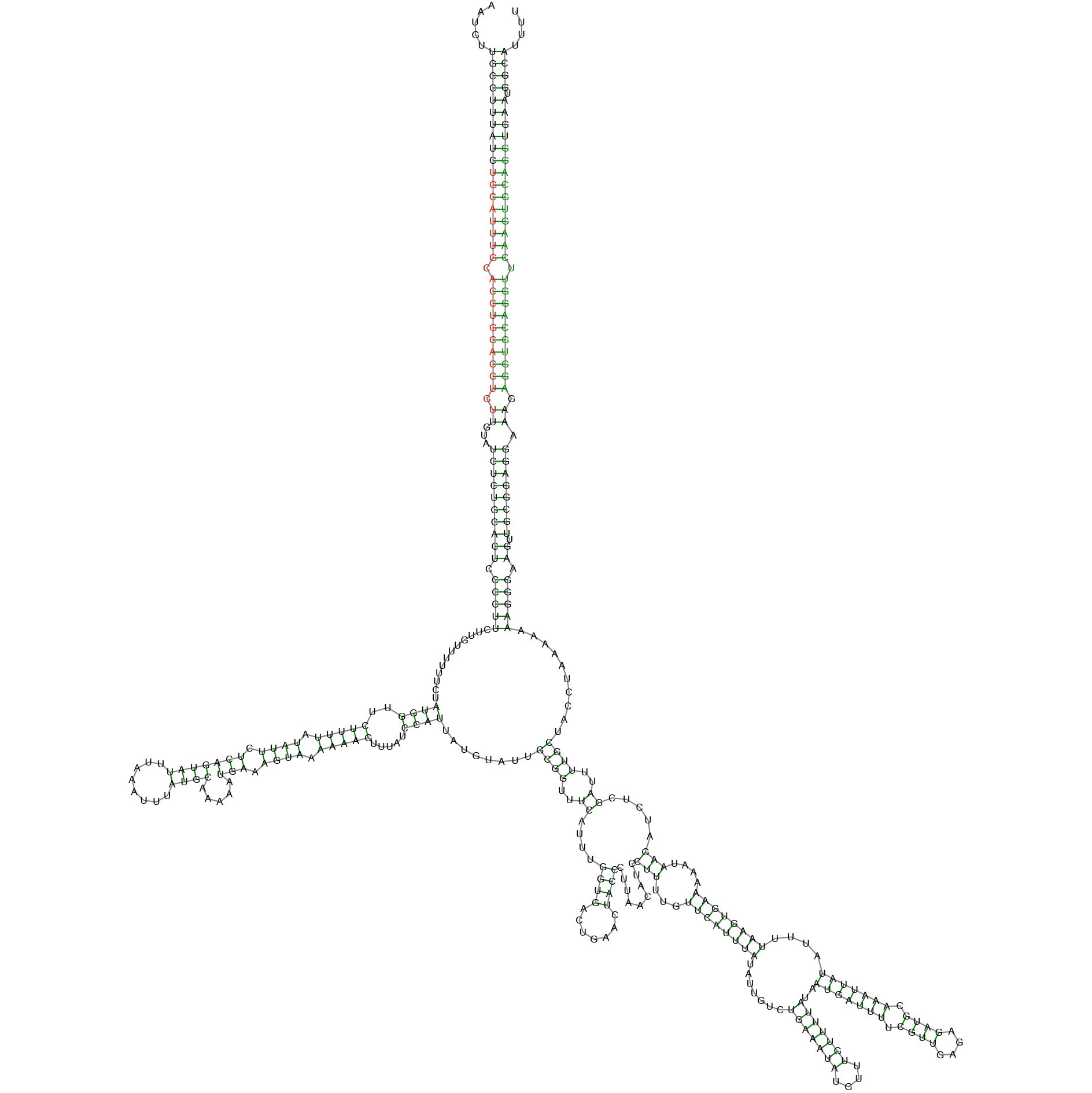

Supplement: Dataset S1 — Full list of hairpin structures in conserved miRNAs. (ZIP) [file pone.0064238.s001.zip › can-miR530.jpg]

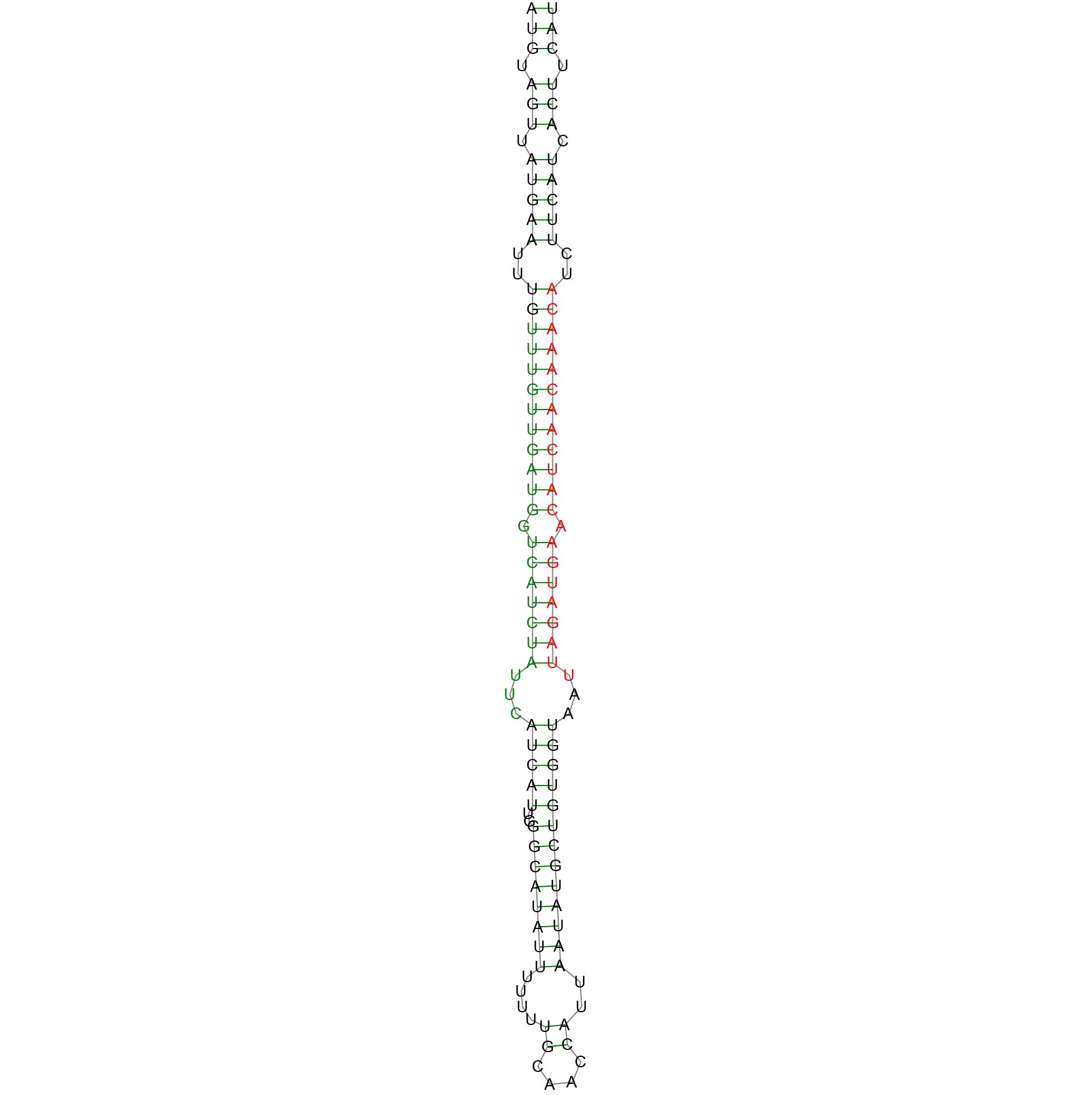

Supplement: Dataset S1 — Full list of hairpin structures in conserved miRNAs. (ZIP) [file pone.0064238.s001.zip › can-miR827.jpg]

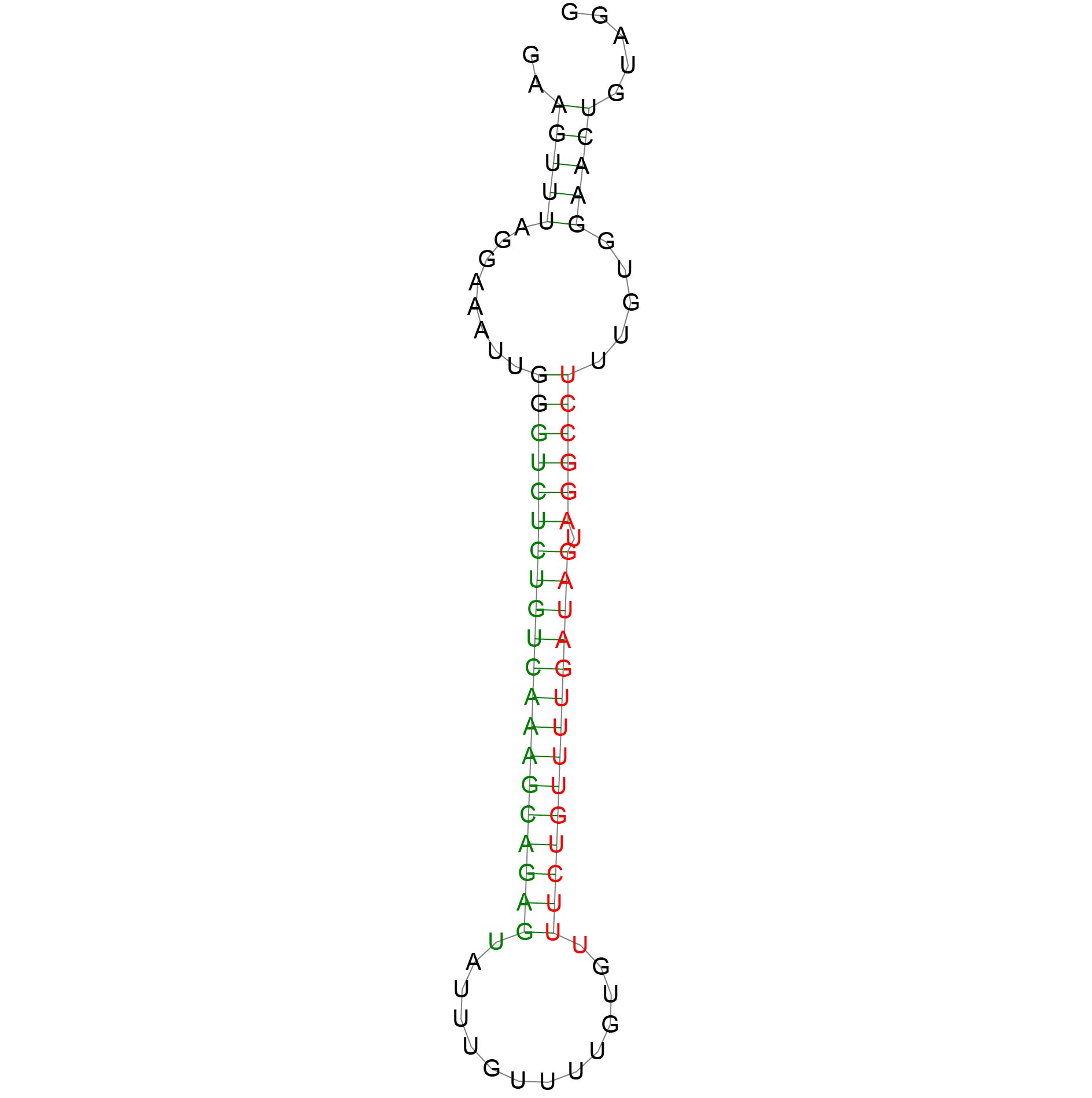

Supplement: Dataset S2 — Full list of hairpin structures in novel miRNAs. (ZIP) [file pone.0064238.s002.zip › can-miR-n001.jpg]

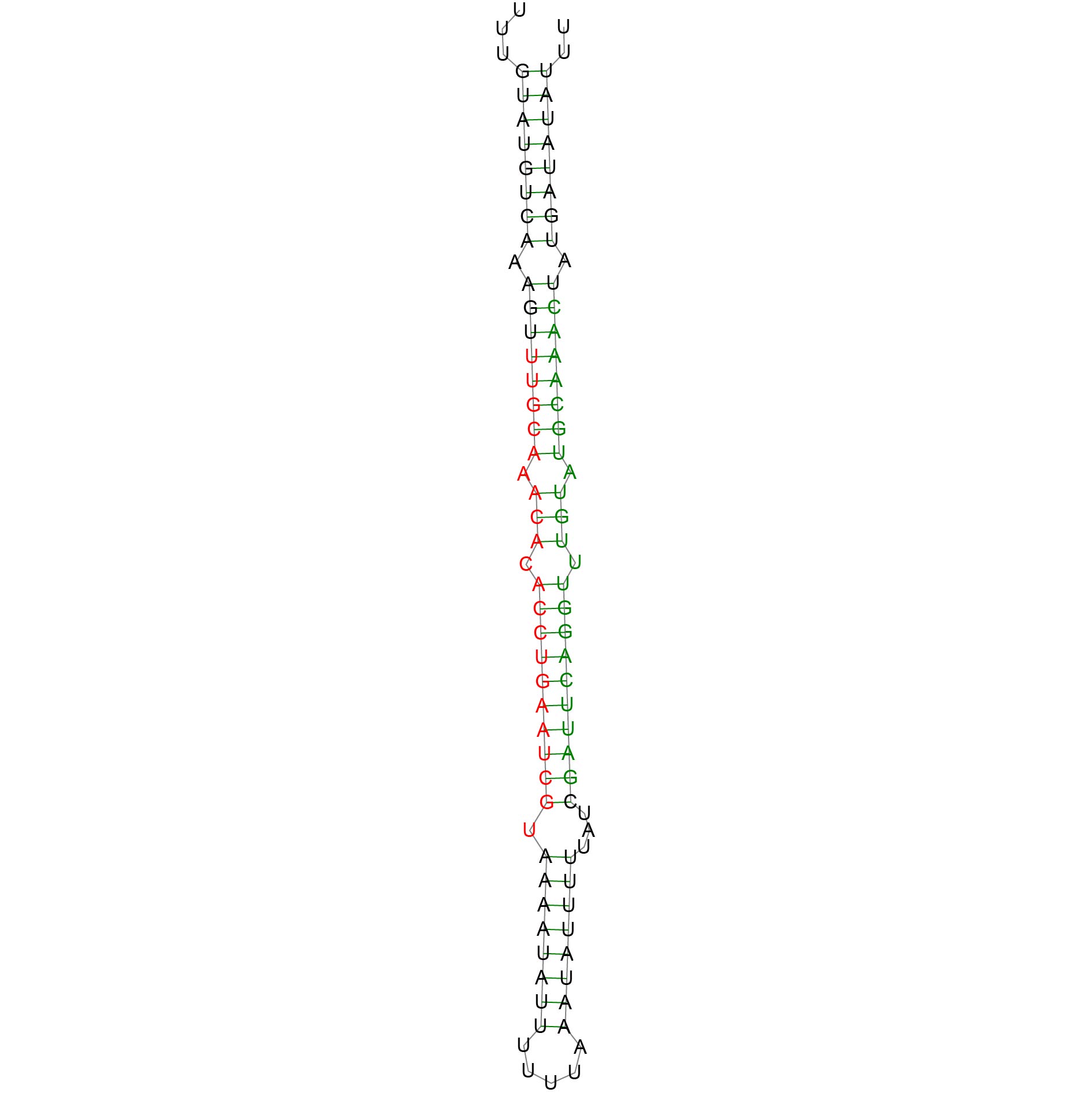

Supplement: Dataset S2 — Full list of hairpin structures in novel miRNAs. (ZIP) [file pone.0064238.s002.zip › can-miR-n002a.jpg]

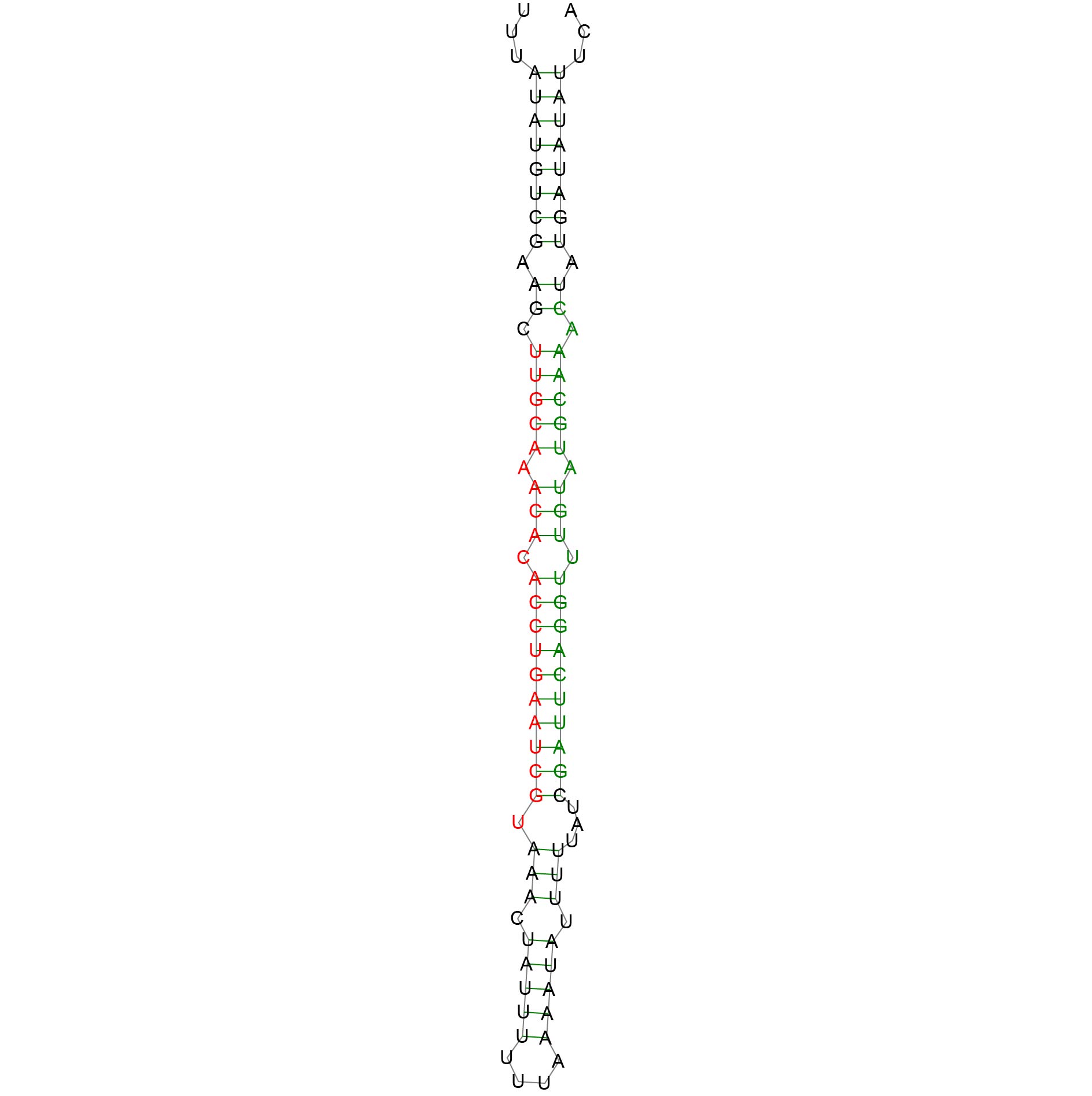

Supplement: Dataset S2 — Full list of hairpin structures in novel miRNAs. (ZIP) [file pone.0064238.s002.zip › can-miR-n002b.jpg]

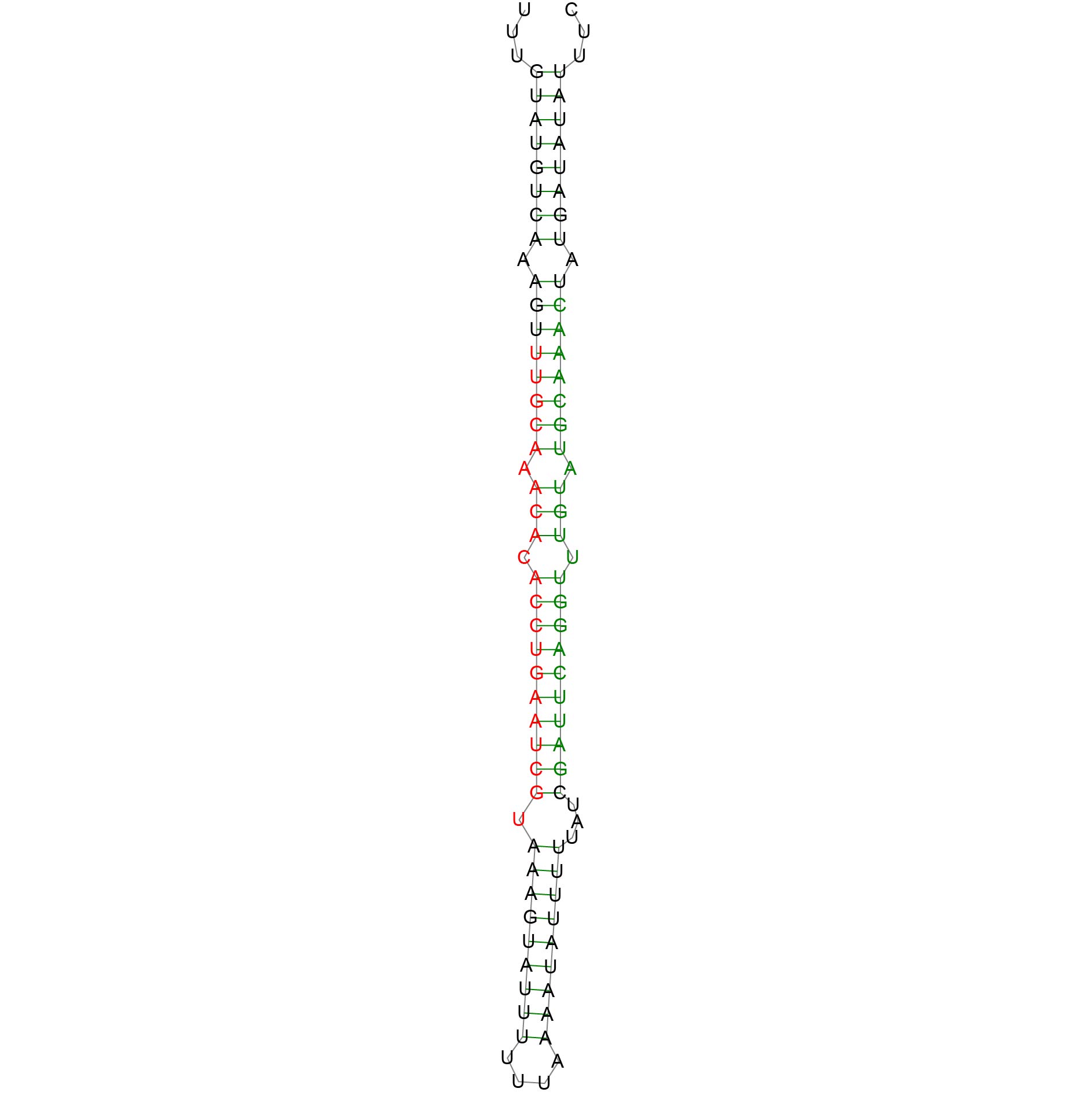

Supplement: Dataset S2 — Full list of hairpin structures in novel miRNAs. (ZIP) [file pone.0064238.s002.zip › can-miR-n002c.jpg]

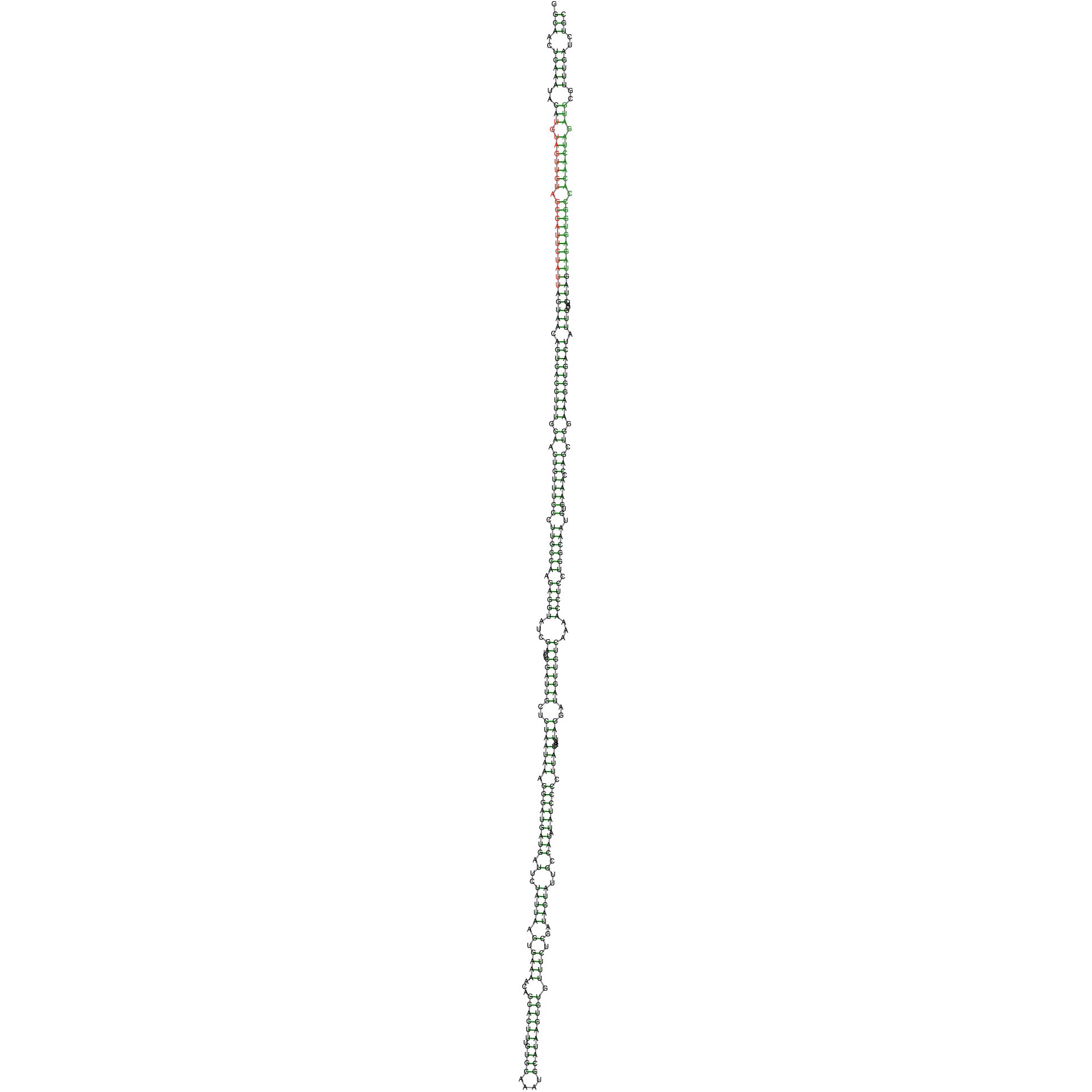

Supplement: Dataset S2 — Full list of hairpin structures in novel miRNAs. (ZIP) [file pone.0064238.s002.zip › can-miR-n003a.jpg]

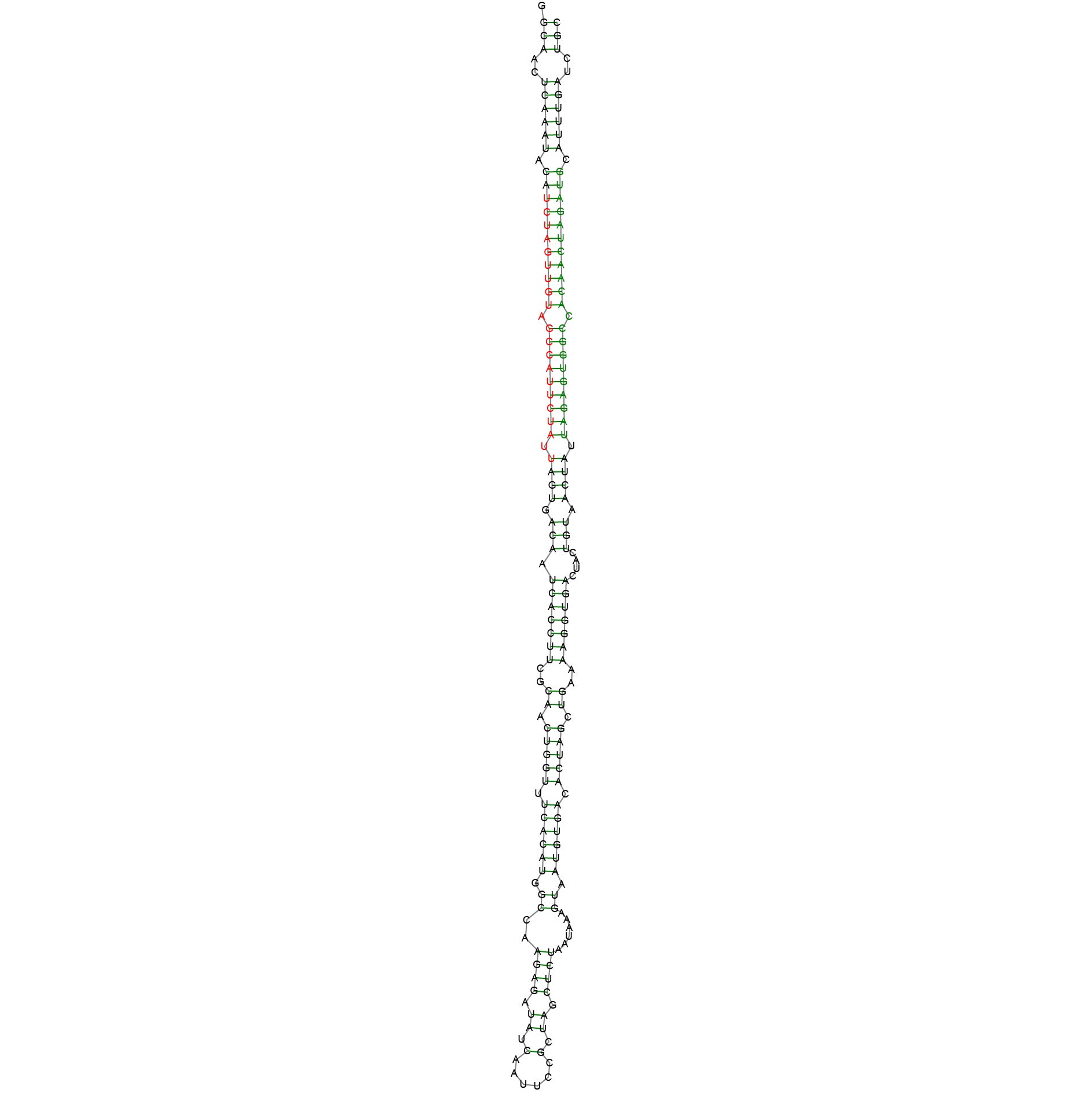

Supplement: Dataset S2 — Full list of hairpin structures in novel miRNAs. (ZIP) [file pone.0064238.s002.zip › can-miR-n003b.jpg]

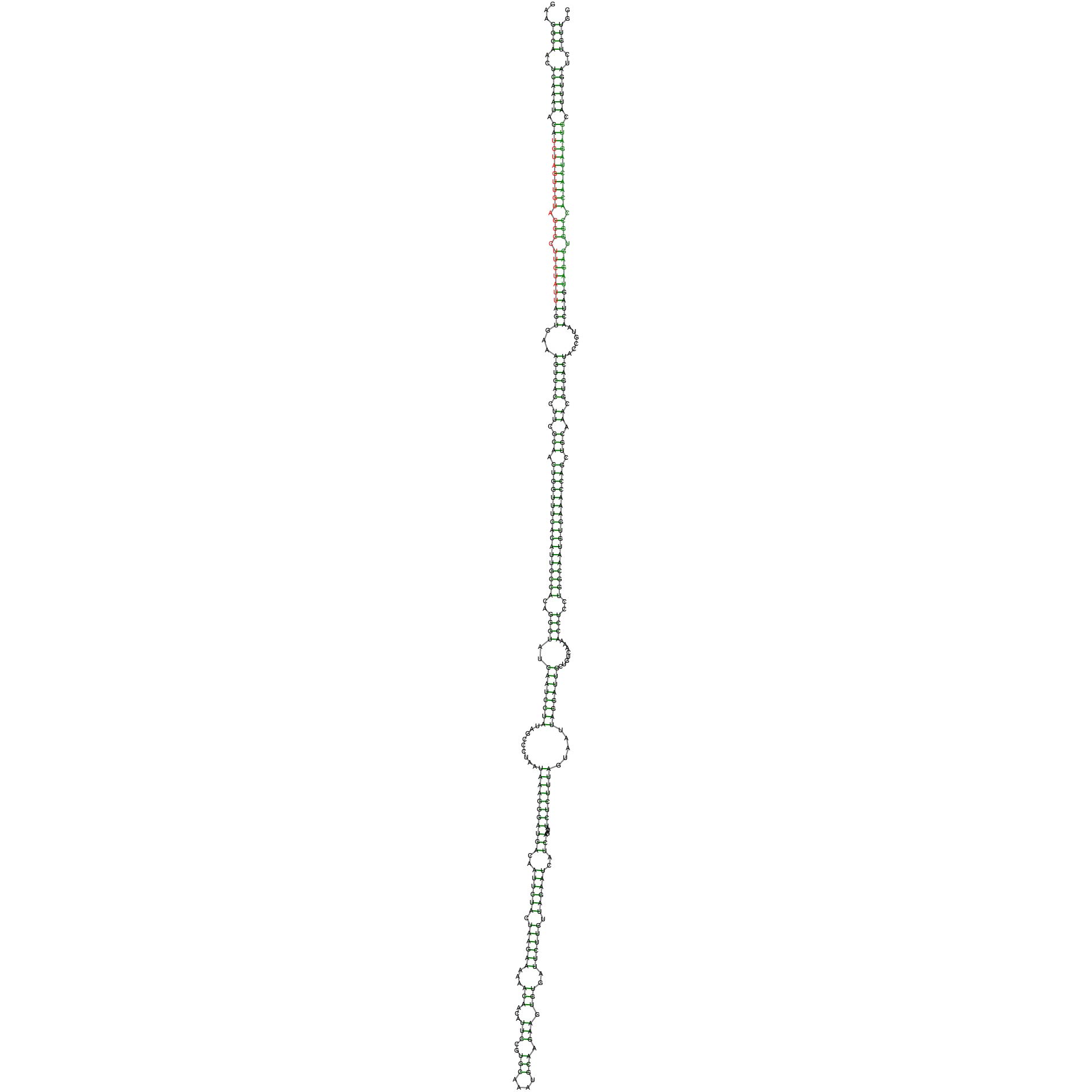

Supplement: Dataset S2 — Full list of hairpin structures in novel miRNAs. (ZIP) [file pone.0064238.s002.zip › can-miR-n003c.jpg]

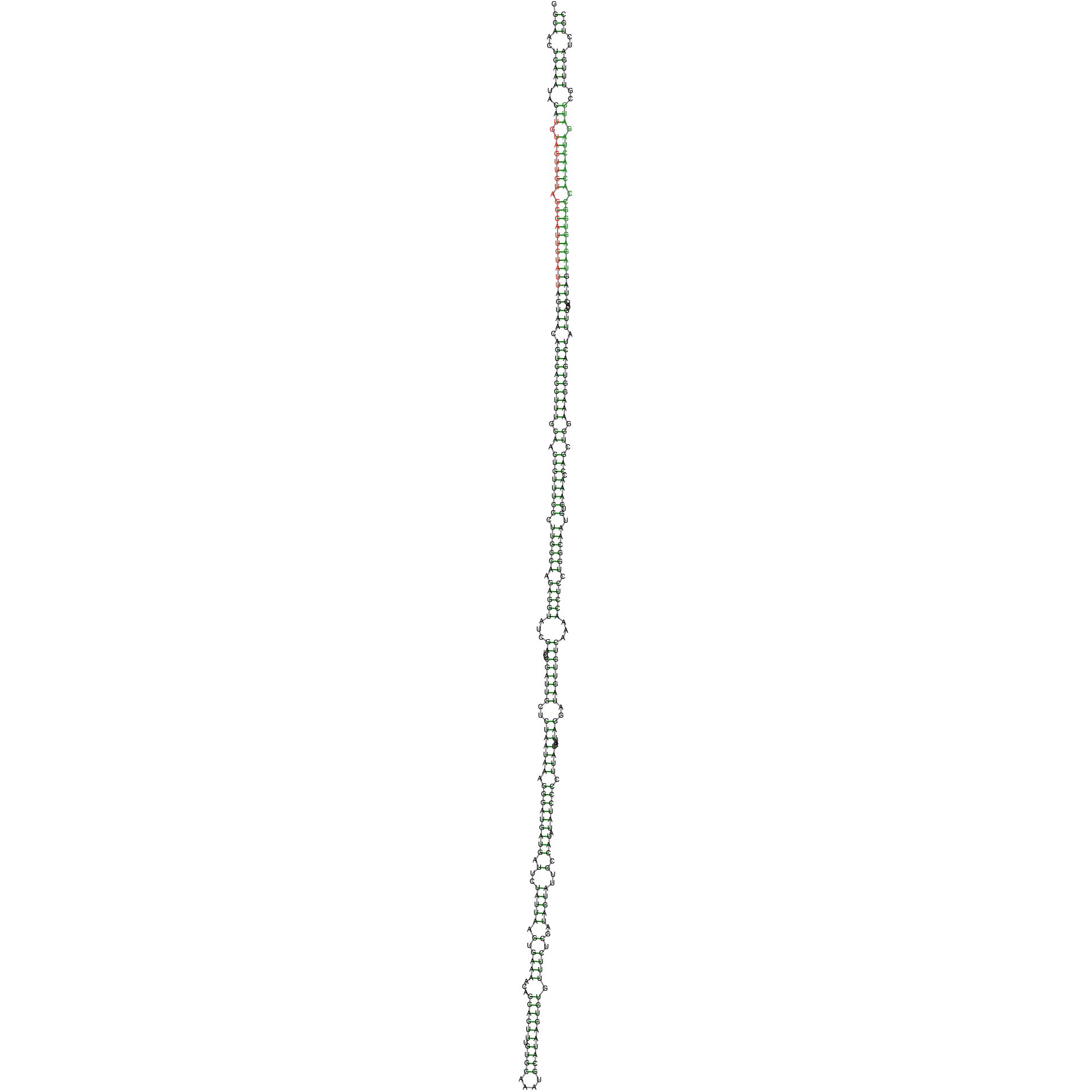

Supplement: Dataset S2 — Full list of hairpin structures in novel miRNAs. (ZIP) [file pone.0064238.s002.zip › can-miR-n003d.jpg]

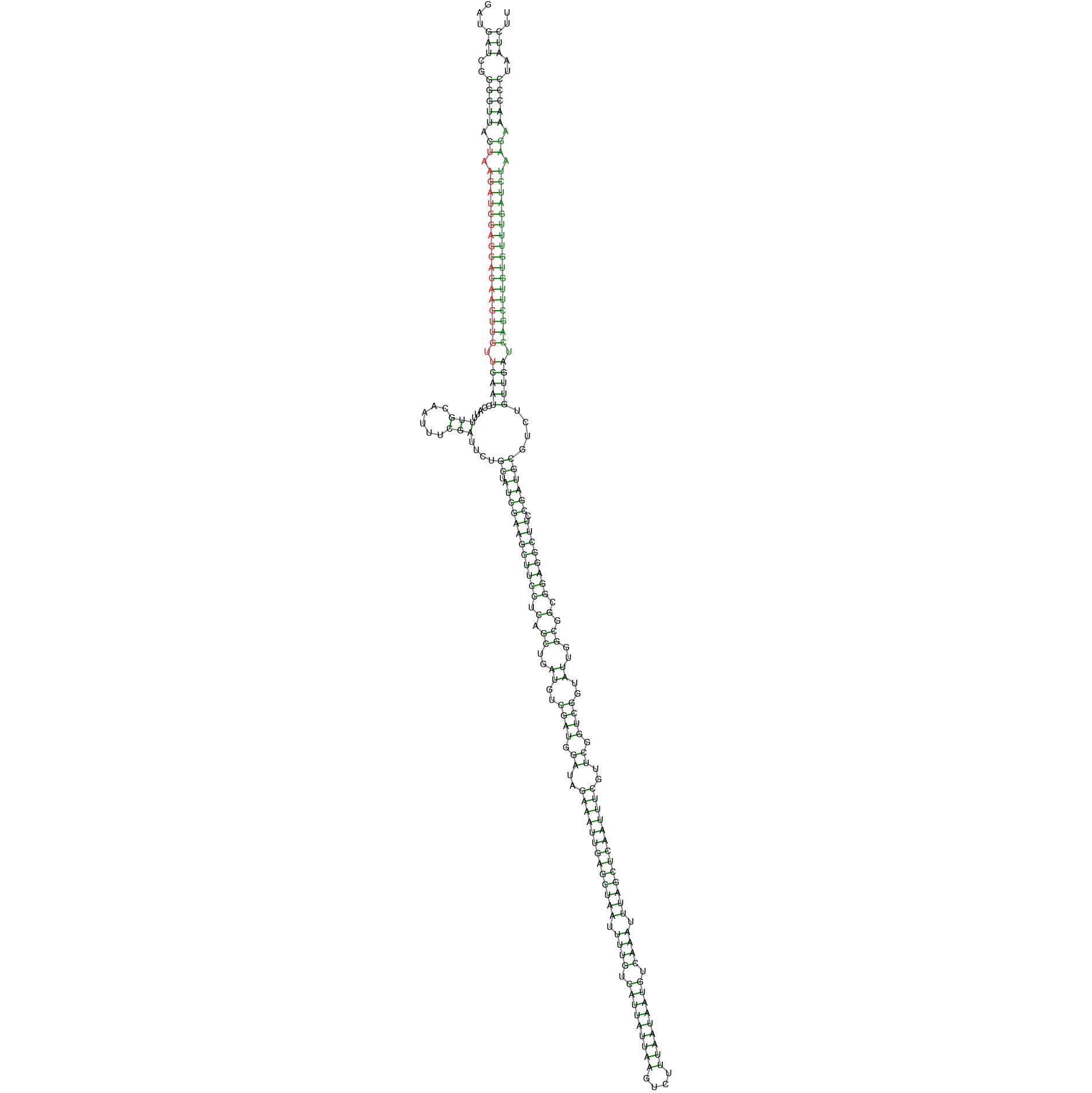

Supplement: Dataset S2 — Full list of hairpin structures in novel miRNAs. (ZIP) [file pone.0064238.s002.zip › can-miR-n004.jpg]

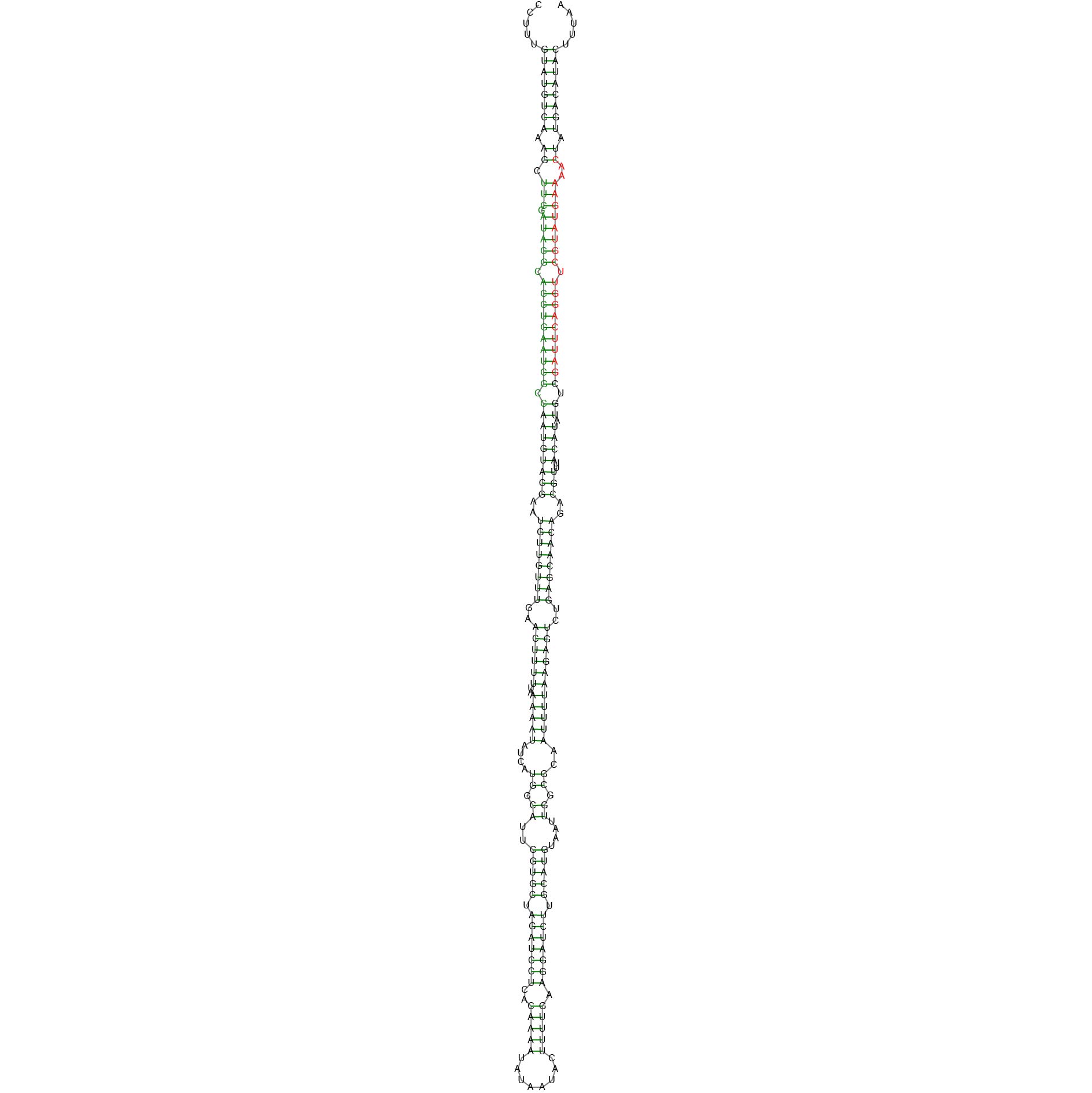

Supplement: Dataset S2 — Full list of hairpin structures in novel miRNAs. (ZIP) [file pone.0064238.s002.zip › can-miR-n005.jpg]

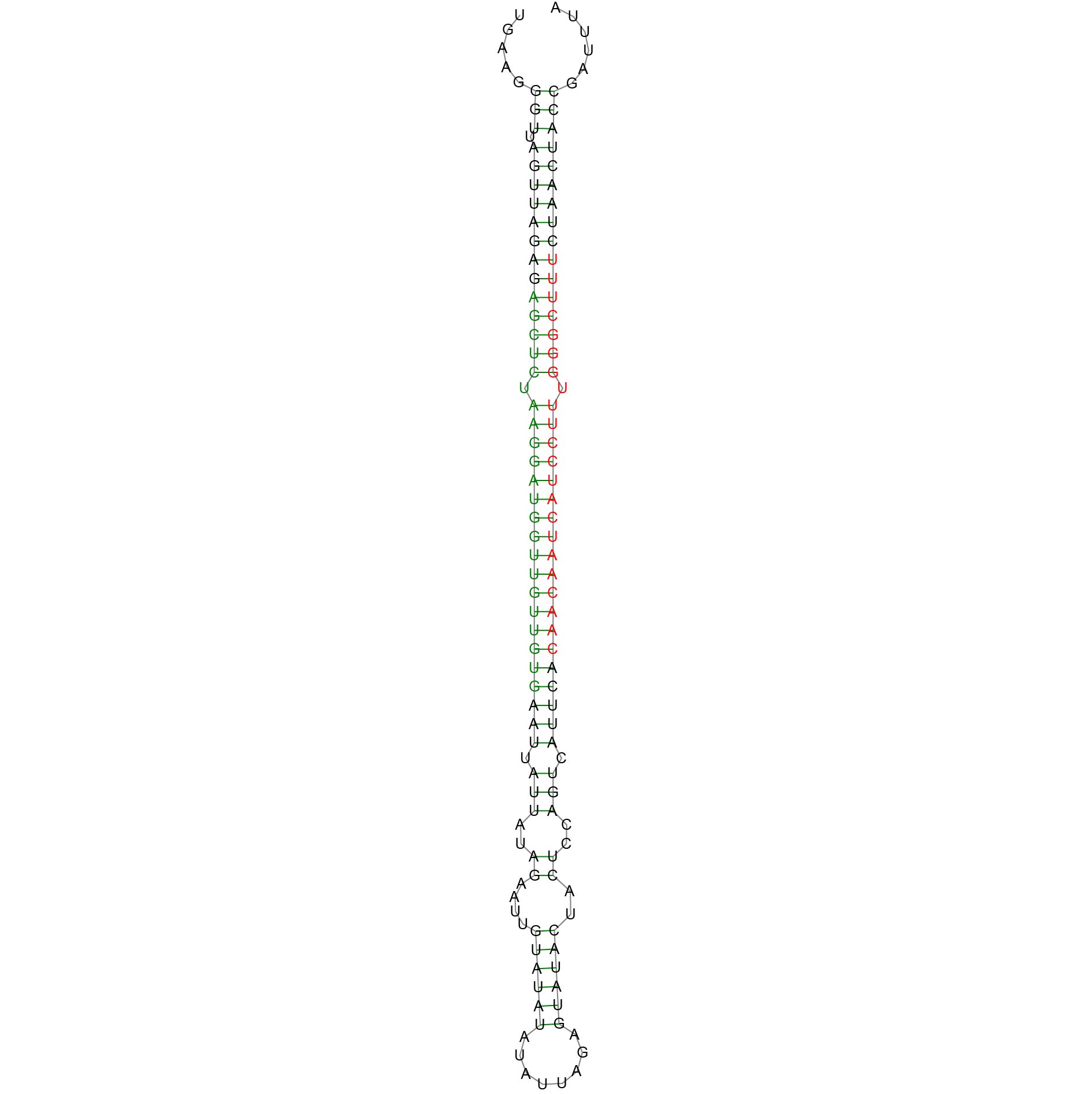

Supplement: Dataset S2 — Full list of hairpin structures in novel miRNAs. (ZIP) [file pone.0064238.s002.zip › can-miR-n006.jpg]

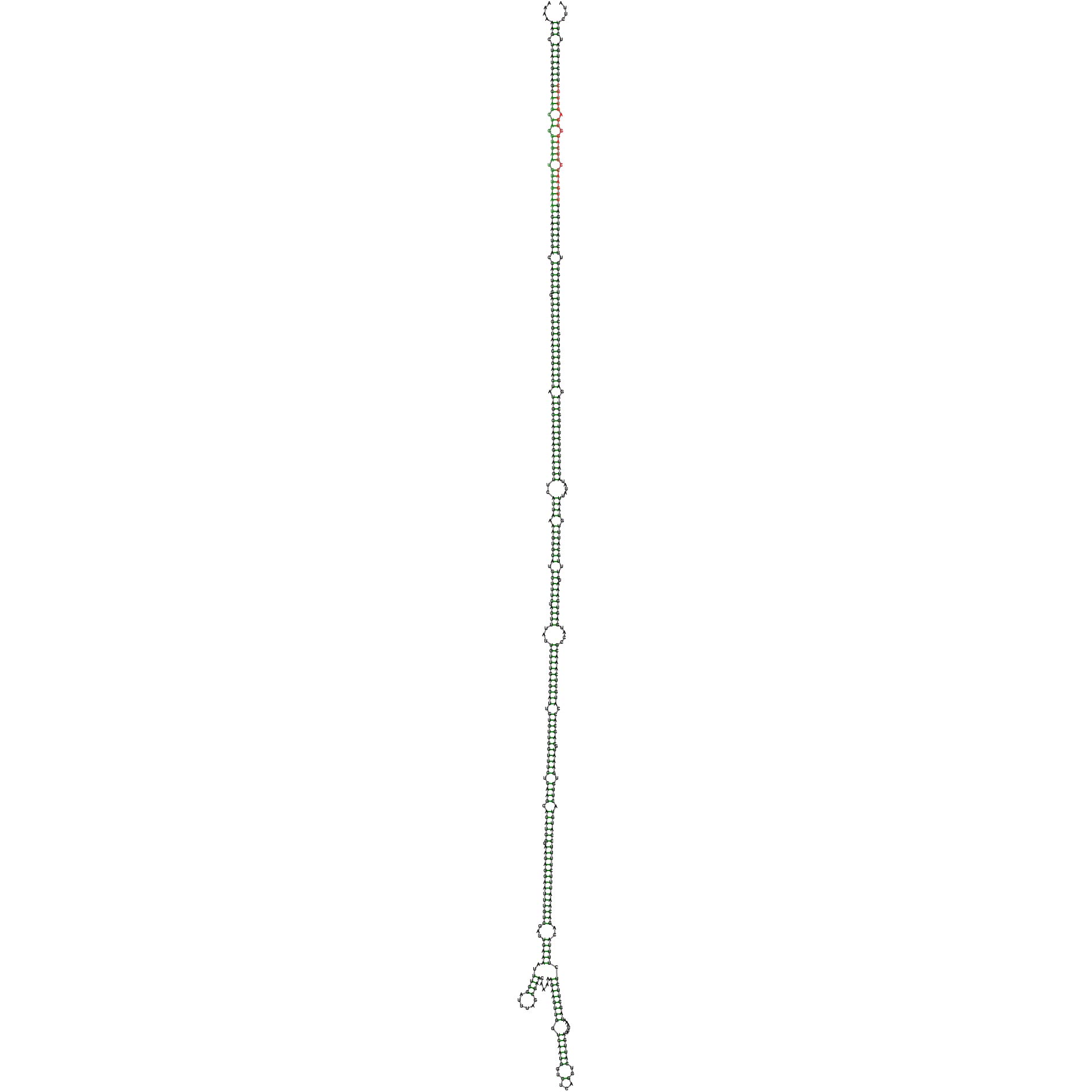

Supplement: Dataset S2 — Full list of hairpin structures in novel miRNAs. (ZIP) [file pone.0064238.s002.zip › can-miR-n007.jpg]

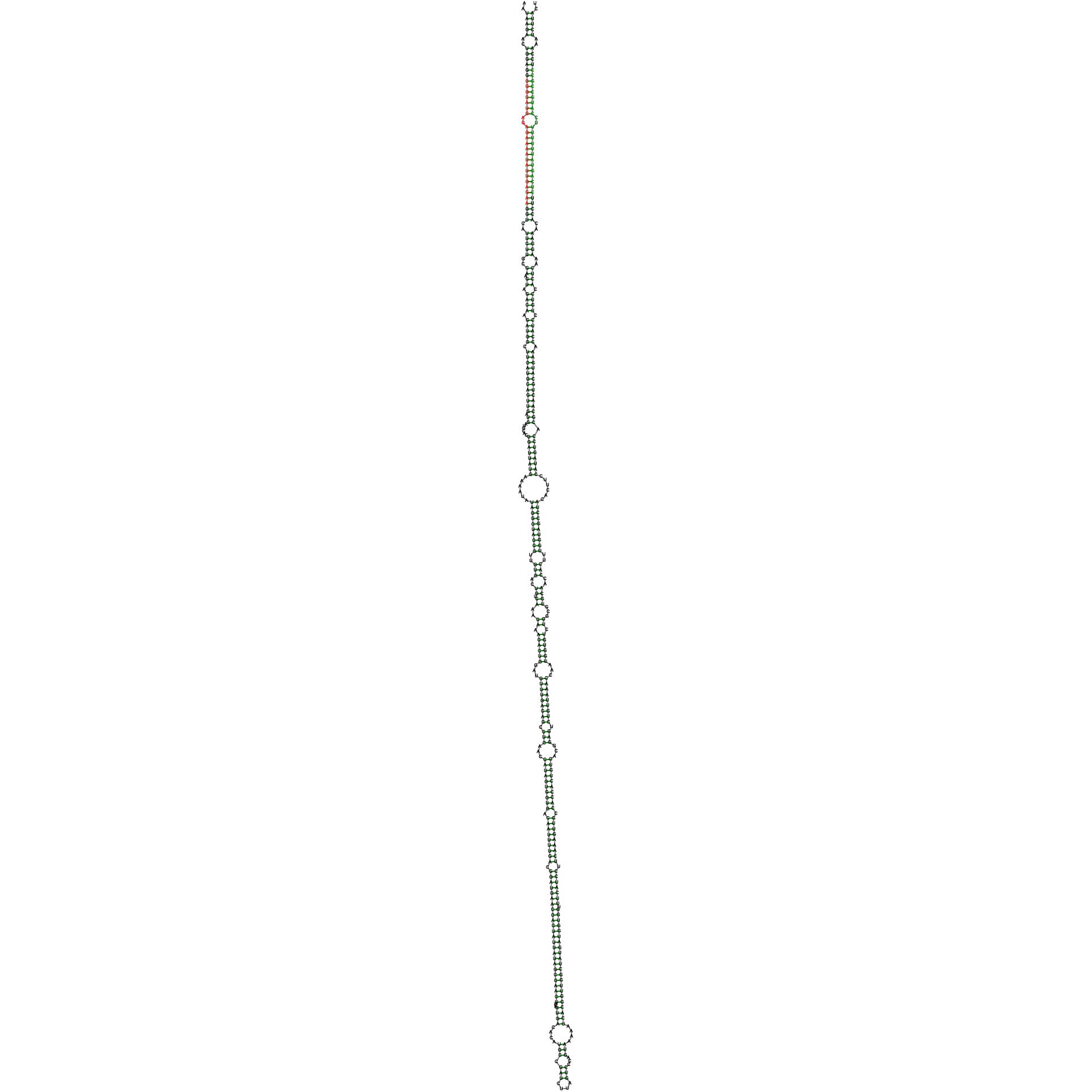

Supplement: Dataset S2 — Full list of hairpin structures in novel miRNAs. (ZIP) [file pone.0064238.s002.zip › can-miR-n008.jpg]

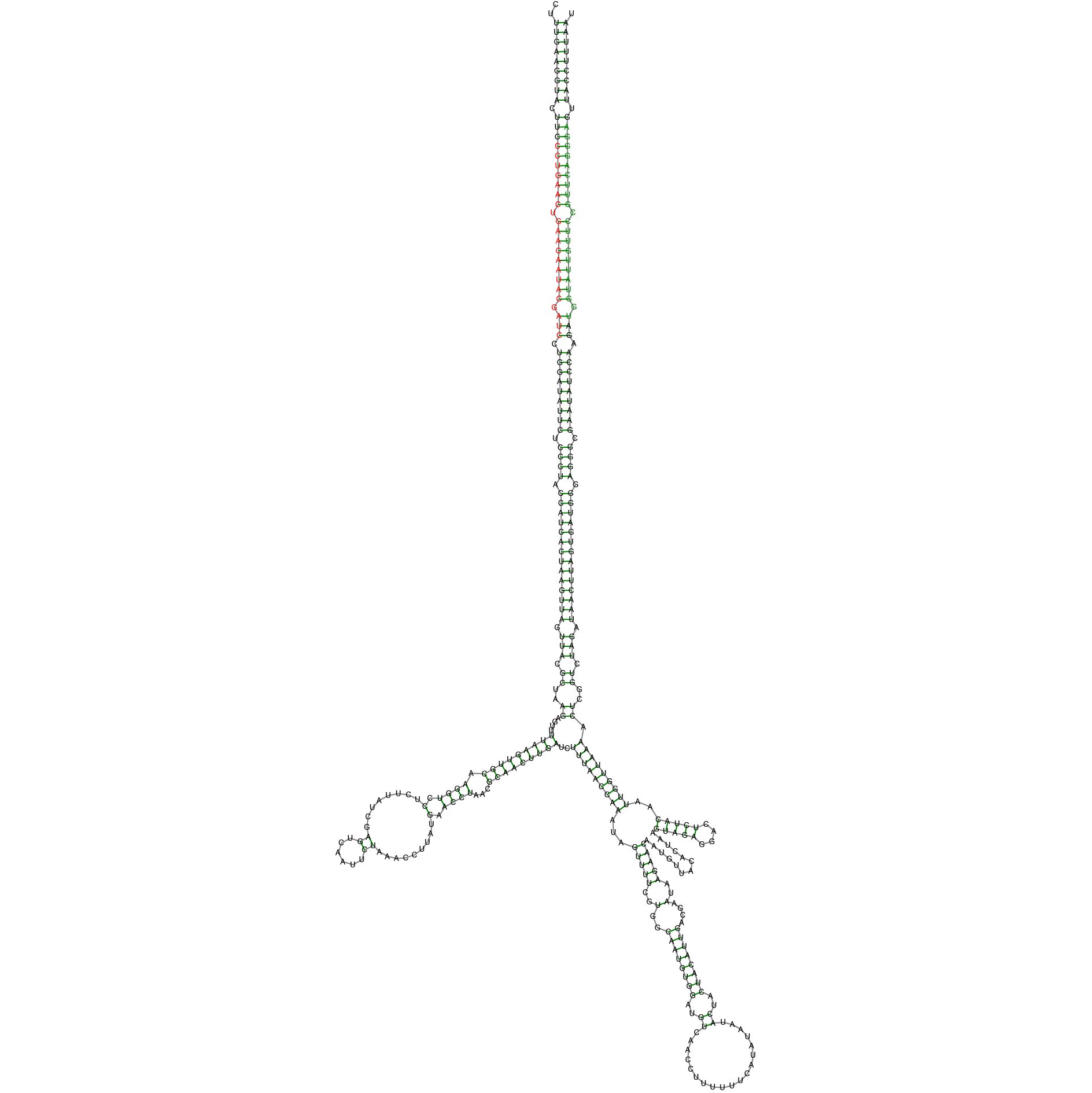

Supplement: Dataset S2 — Full list of hairpin structures in novel miRNAs. (ZIP) [file pone.0064238.s002.zip › can-miR-n009a-5p,3p.jpg]

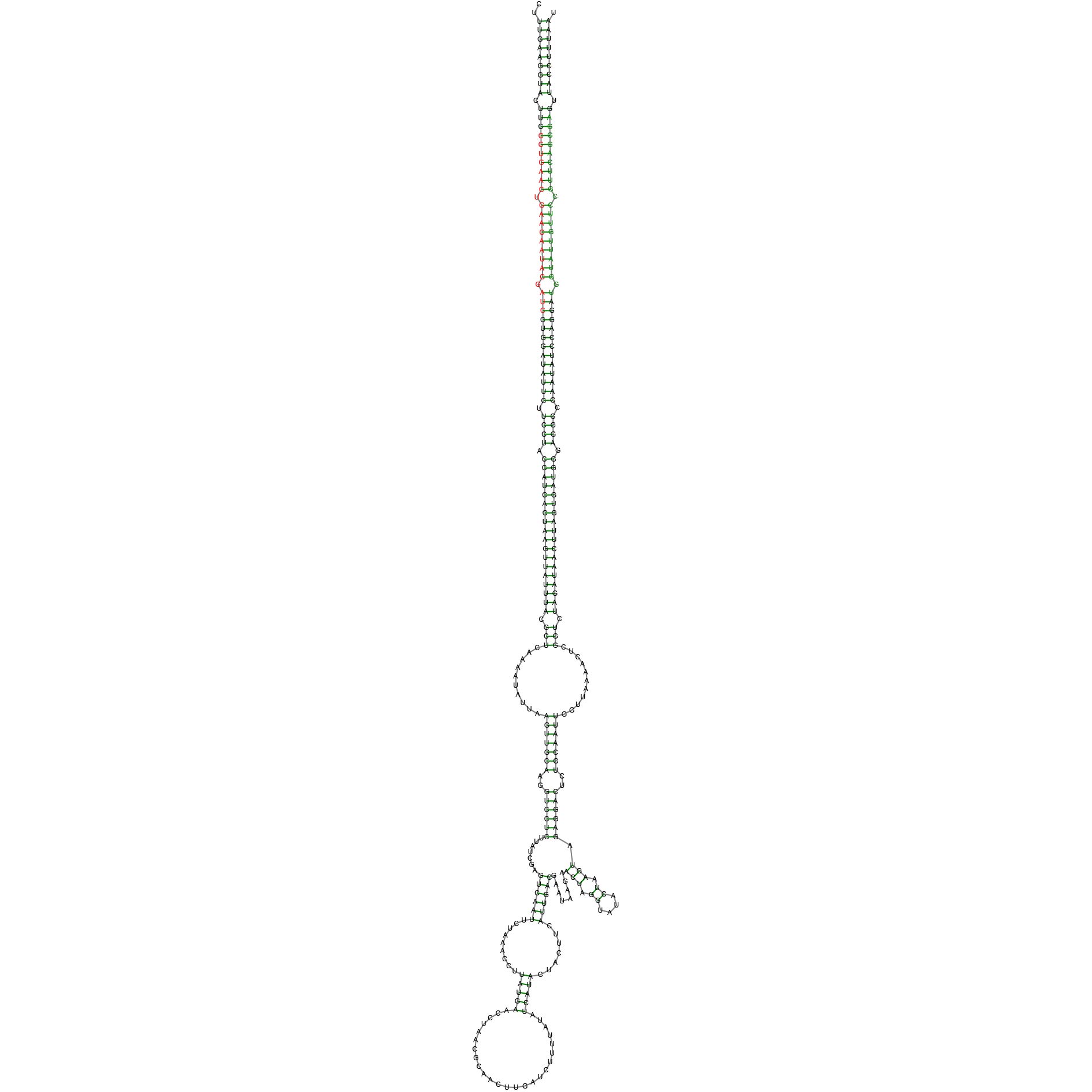

Supplement: Dataset S2 — Full list of hairpin structures in novel miRNAs. (ZIP) [file pone.0064238.s002.zip › can-miR-n009b-5p,3p.jpg]

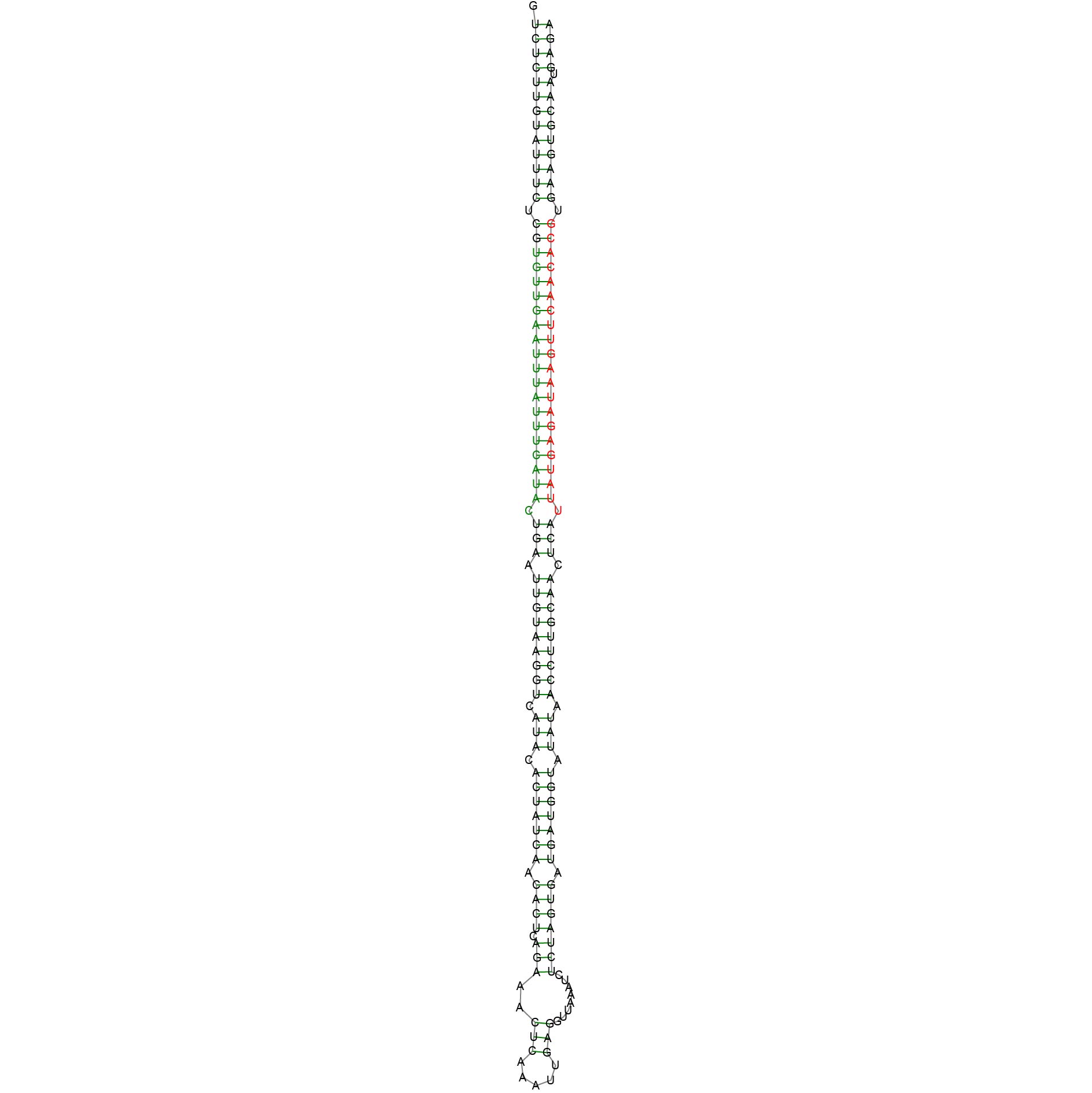

Supplement: Dataset S2 — Full list of hairpin structures in novel miRNAs. (ZIP) [file pone.0064238.s002.zip › can-miR-n010.jpg]

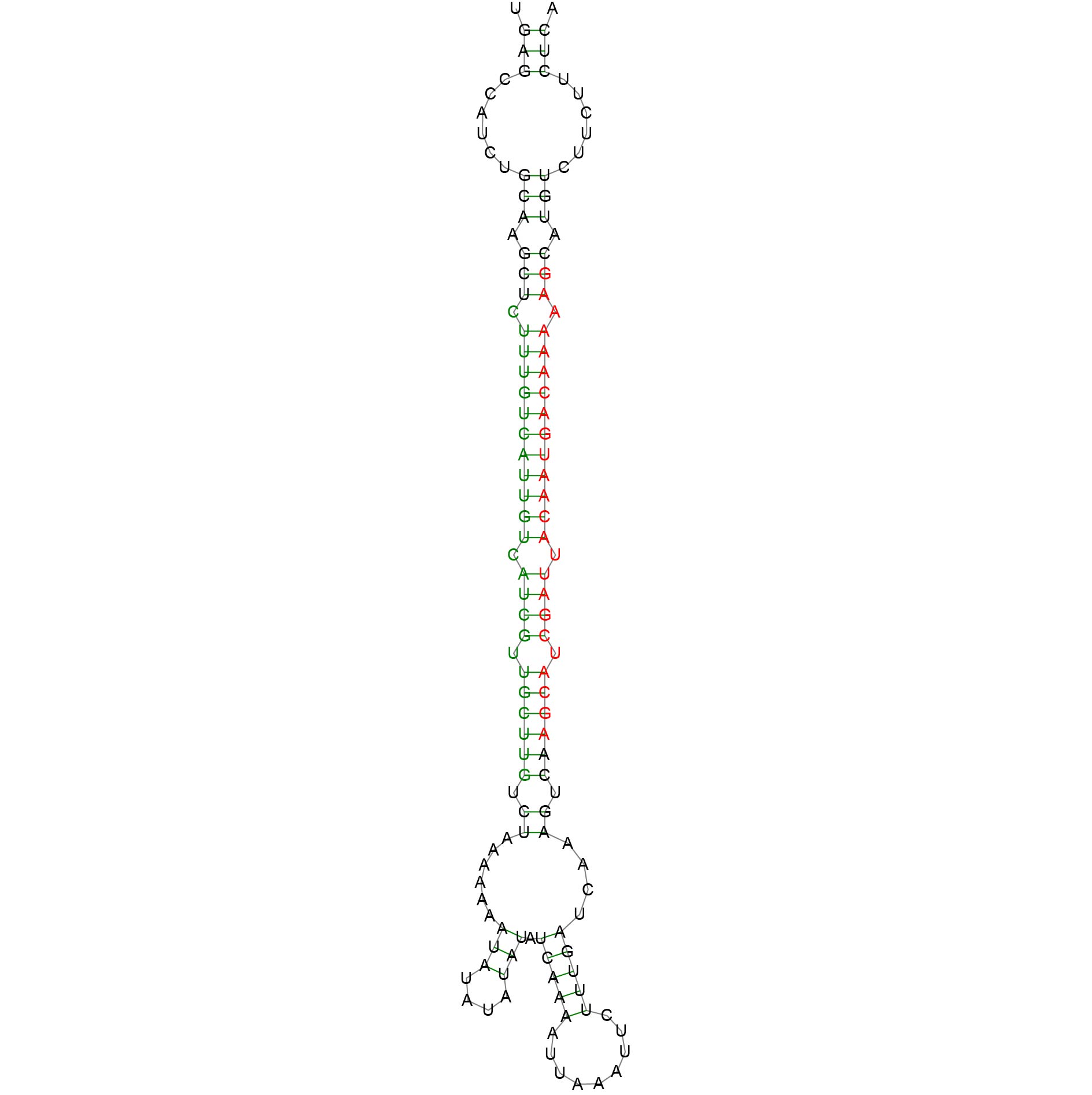

Supplement: Dataset S2 — Full list of hairpin structures in novel miRNAs. (ZIP) [file pone.0064238.s002.zip › can-miR-n011.jpg]

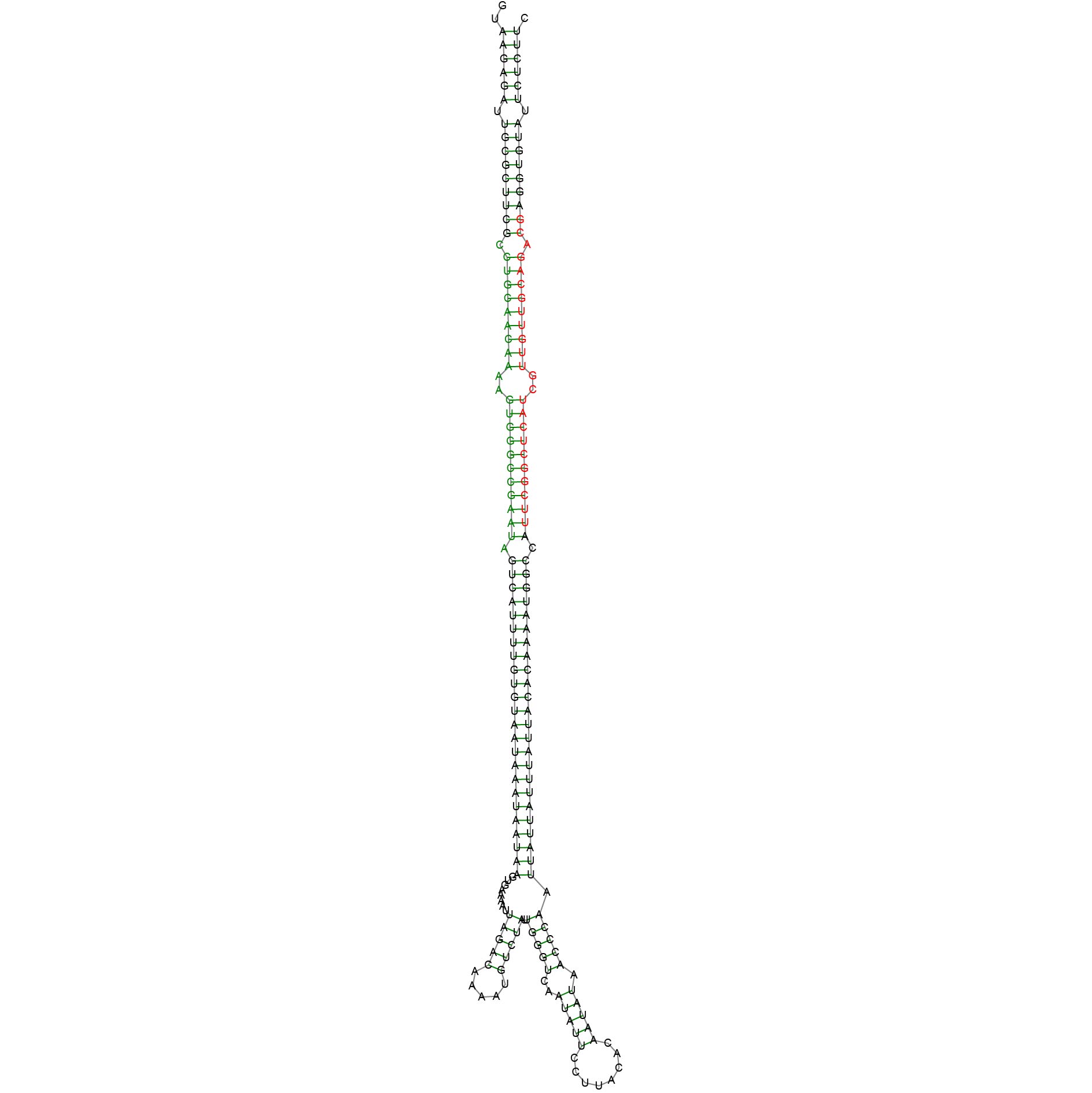

Supplement: Dataset S2 — Full list of hairpin structures in novel miRNAs. (ZIP) [file pone.0064238.s002.zip › can-miR-n012.jpg]

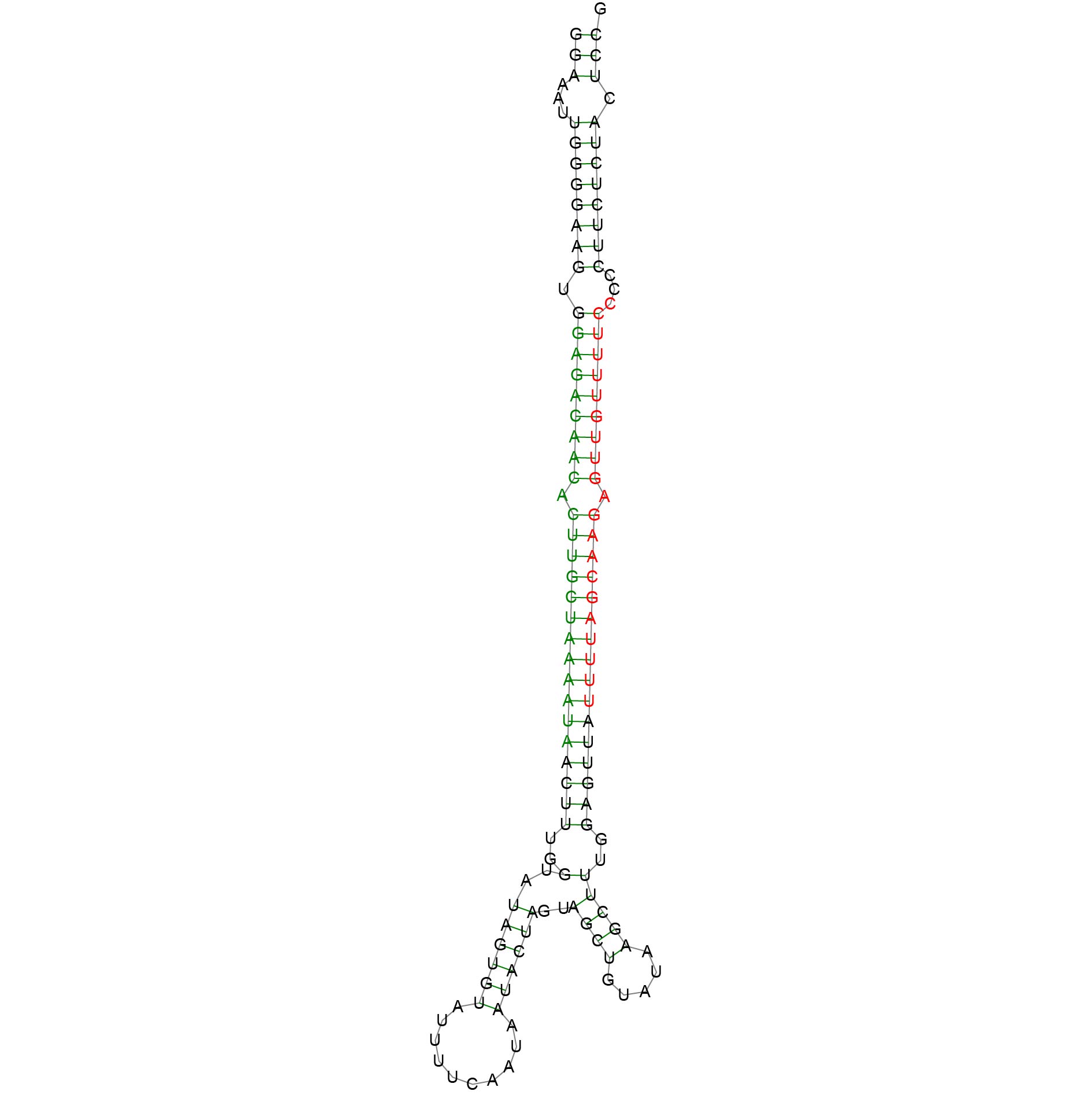

Supplement: Dataset S2 — Full list of hairpin structures in novel miRNAs. (ZIP) [file pone.0064238.s002.zip › can-miR-n013.jpg]

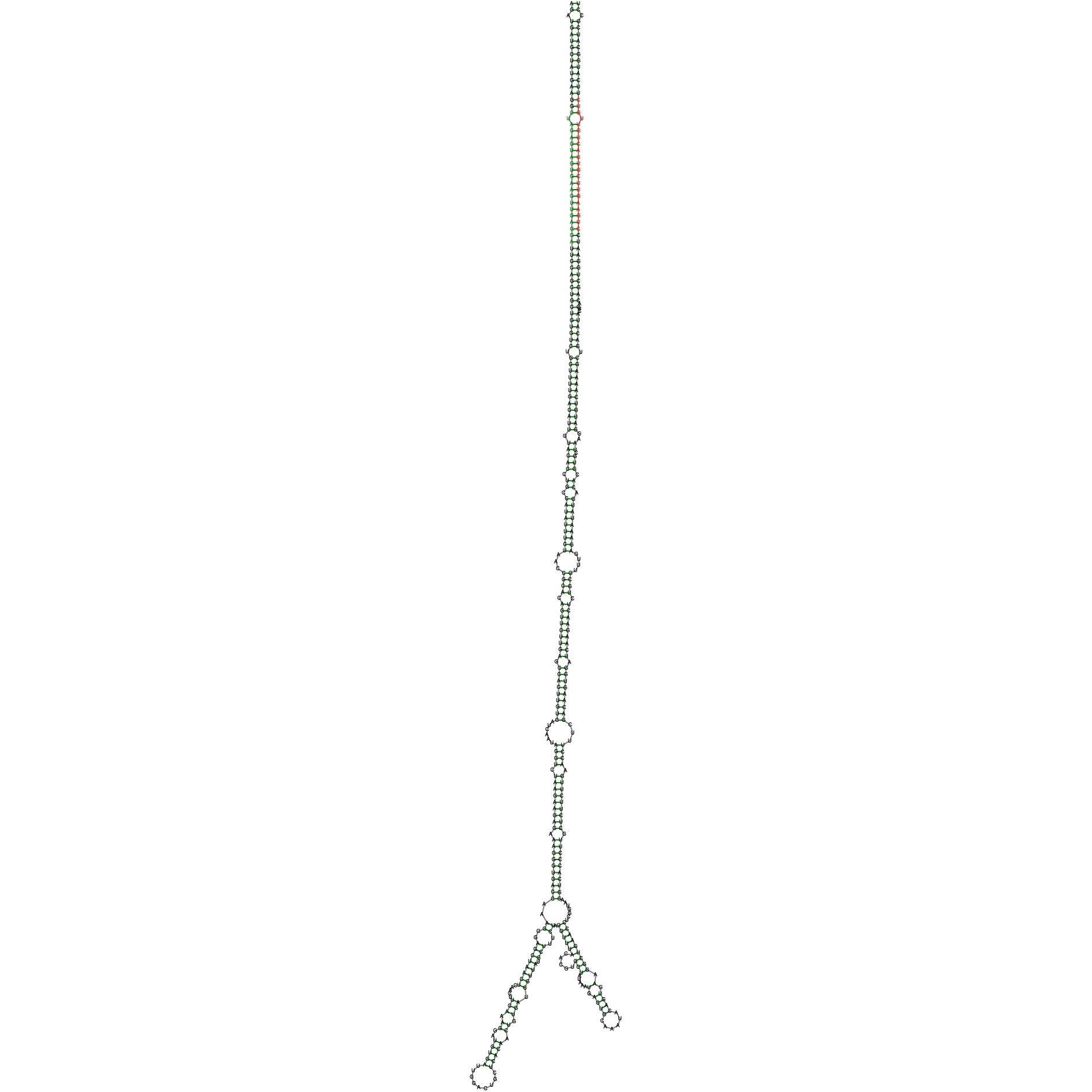

Supplement: Dataset S2 — Full list of hairpin structures in novel miRNAs. (ZIP) [file pone.0064238.s002.zip › can-miR-n014.jpg]

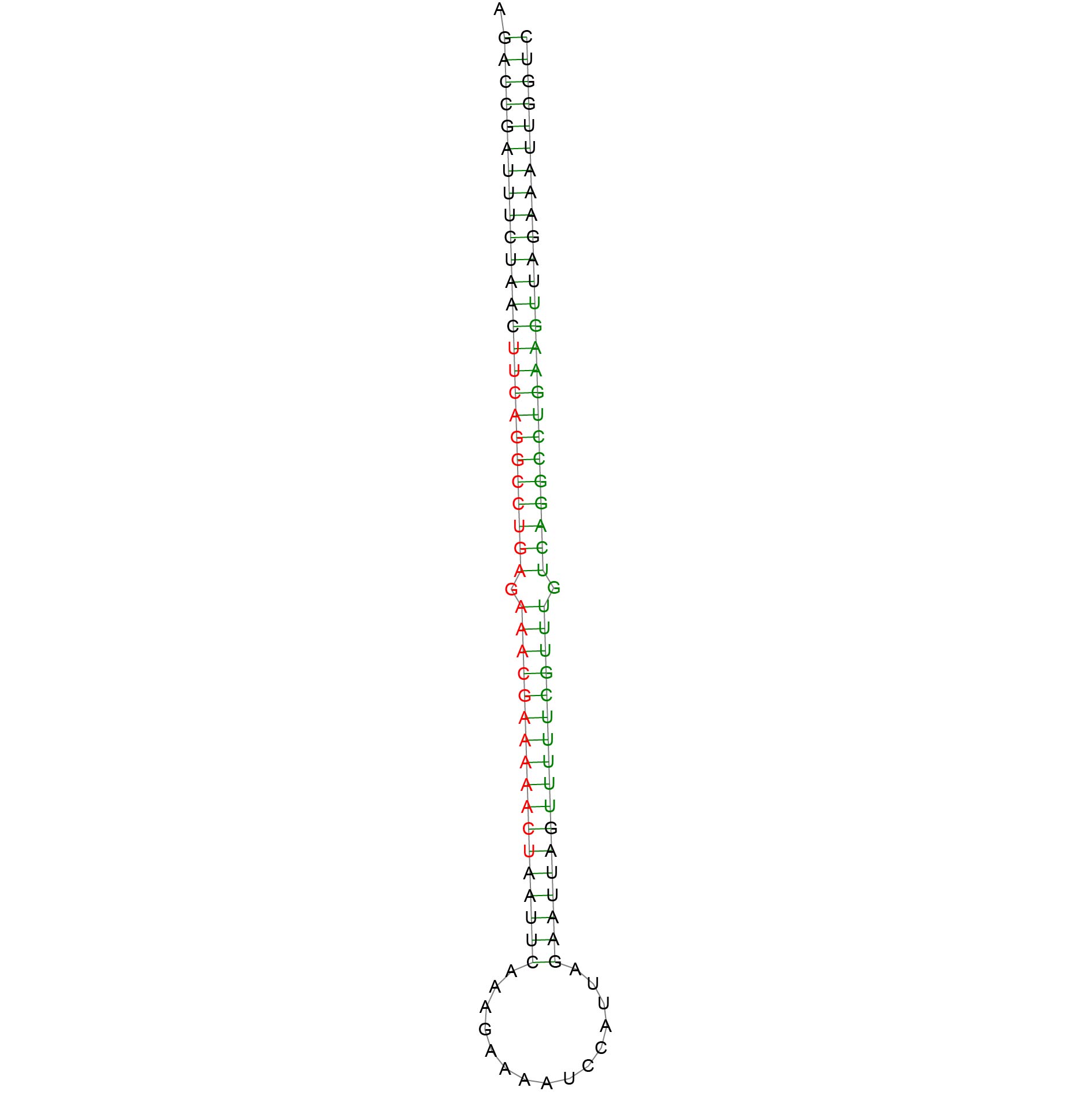

Supplement: Dataset S2 — Full list of hairpin structures in novel miRNAs. (ZIP) [file pone.0064238.s002.zip › can-miR-n015.jpg]

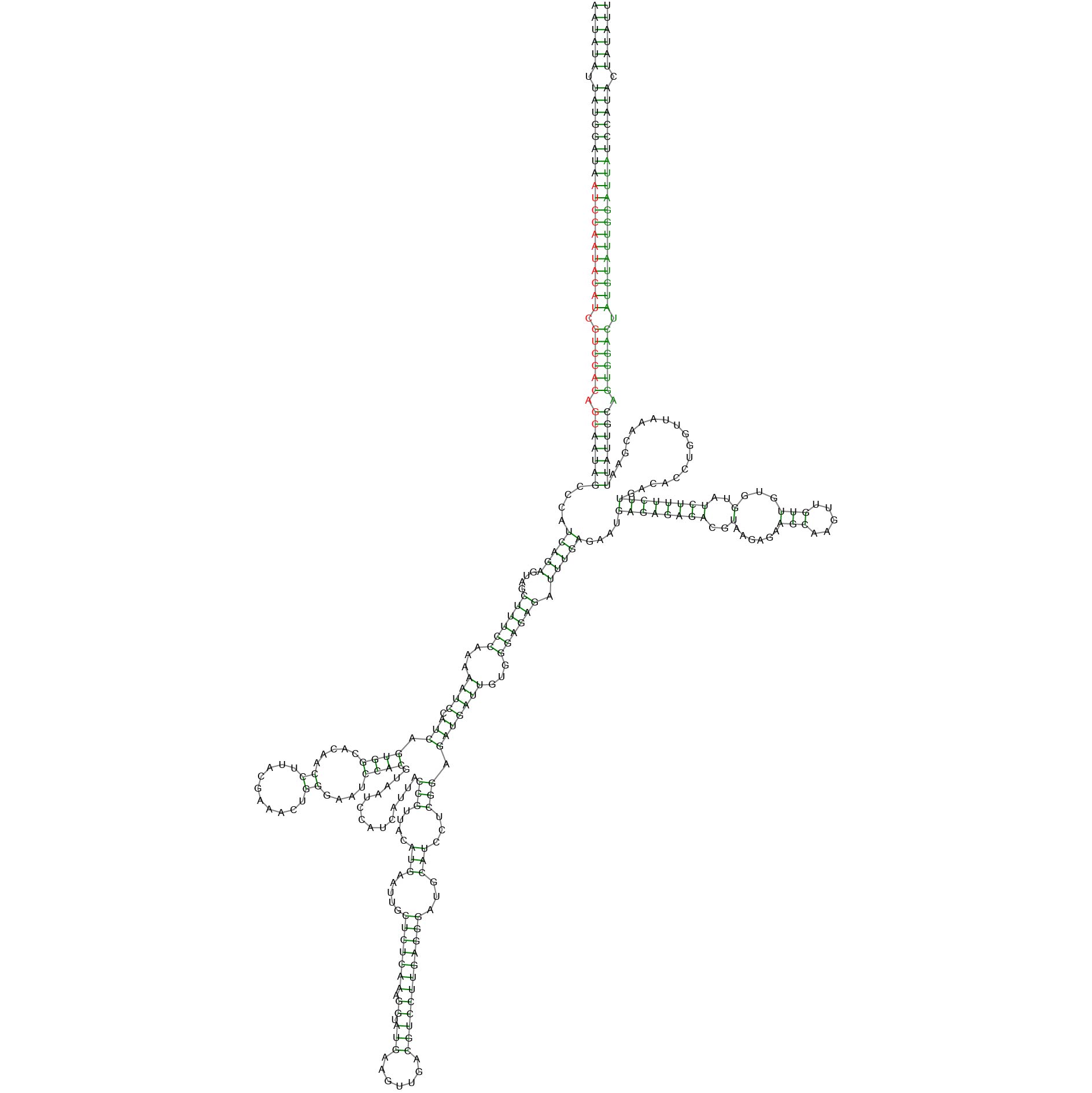

Supplement: Dataset S2 — Full list of hairpin structures in novel miRNAs. (ZIP) [file pone.0064238.s002.zip › can-miR-n016a.jpg]

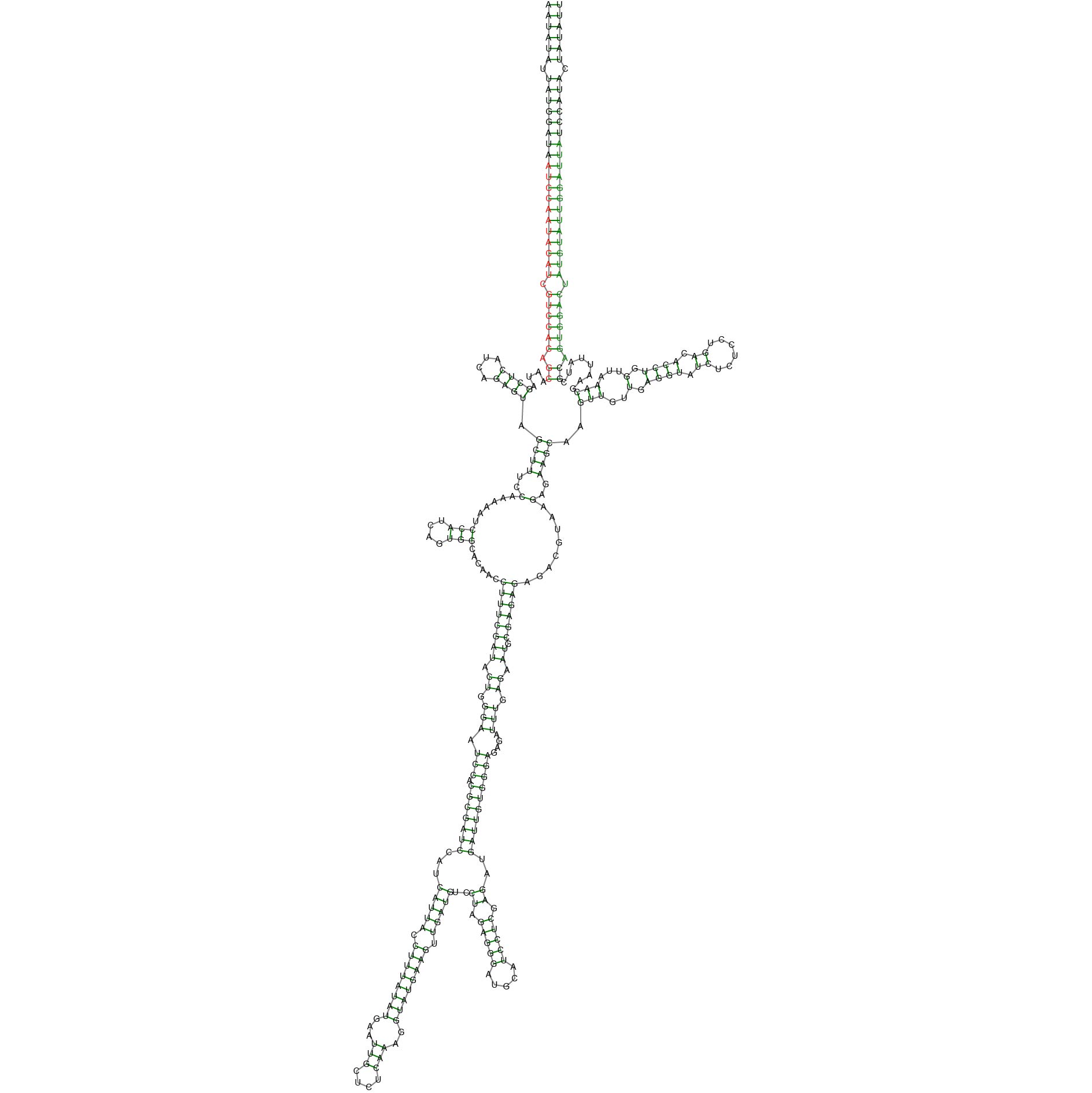

Supplement: Dataset S2 — Full list of hairpin structures in novel miRNAs. (ZIP) [file pone.0064238.s002.zip › can-miR-n016b.jpg]

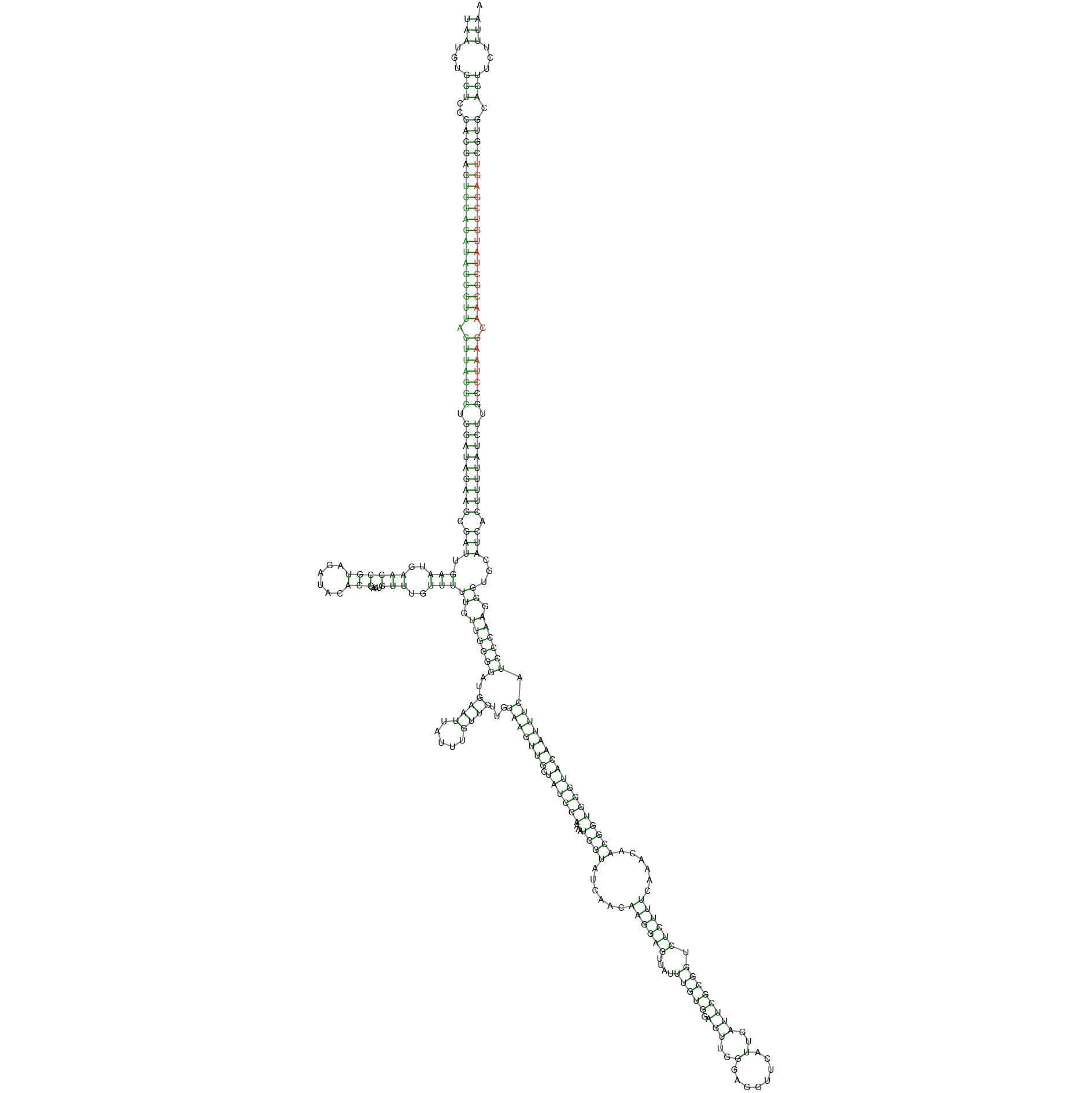

Supplement: Dataset S2 — Full list of hairpin structures in novel miRNAs. (ZIP) [file pone.0064238.s002.zip › can-miR-n017.jpg]

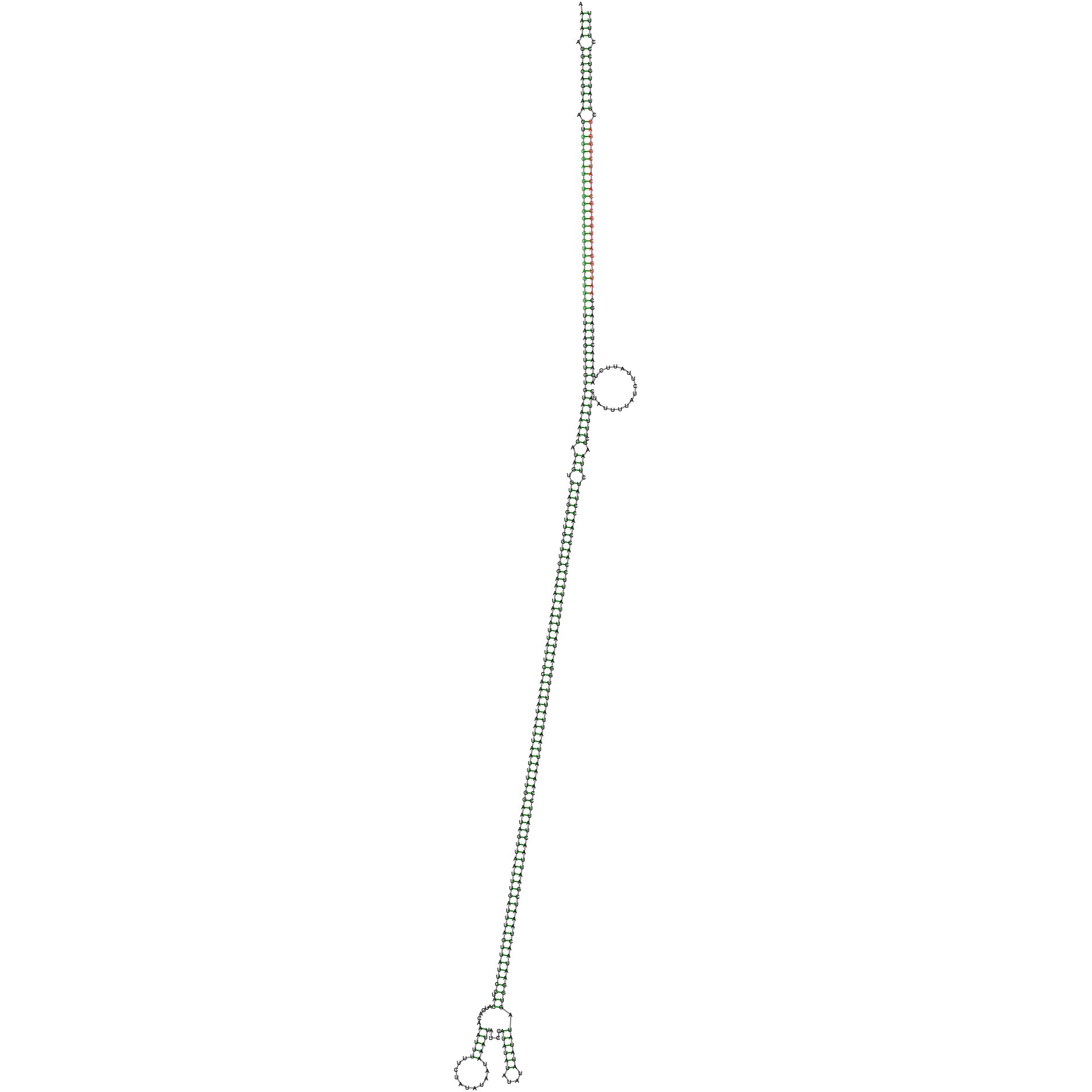

Supplement: Dataset S2 — Full list of hairpin structures in novel miRNAs. (ZIP) [file pone.0064238.s002.zip › can-miR-n018.jpg]

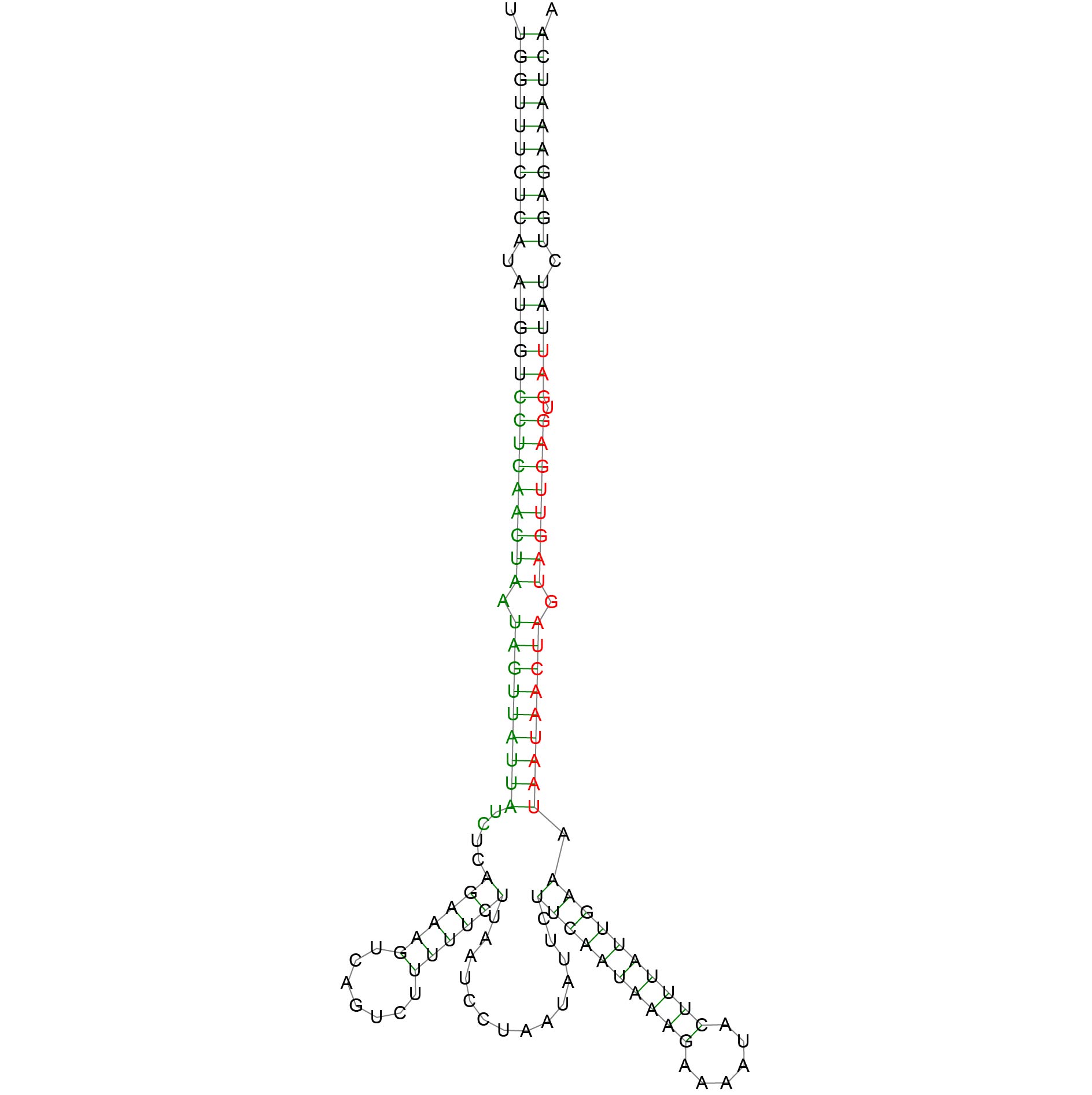

Supplement: Dataset S2 — Full list of hairpin structures in novel miRNAs. (ZIP) [file pone.0064238.s002.zip › can-miR-n019a.jpg]

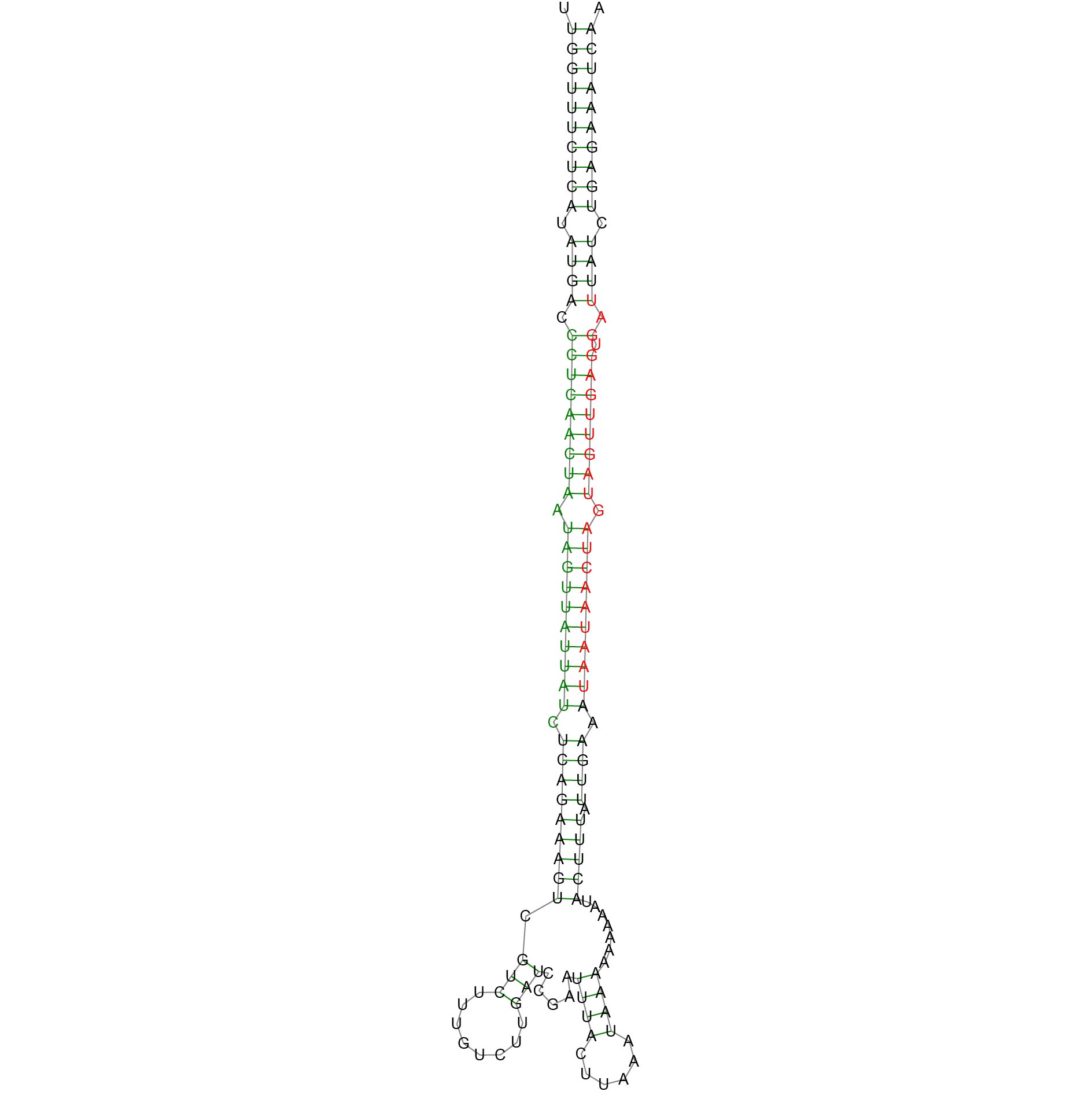

Supplement: Dataset S2 — Full list of hairpin structures in novel miRNAs. (ZIP) [file pone.0064238.s002.zip › can-miR-n019b.jpg]

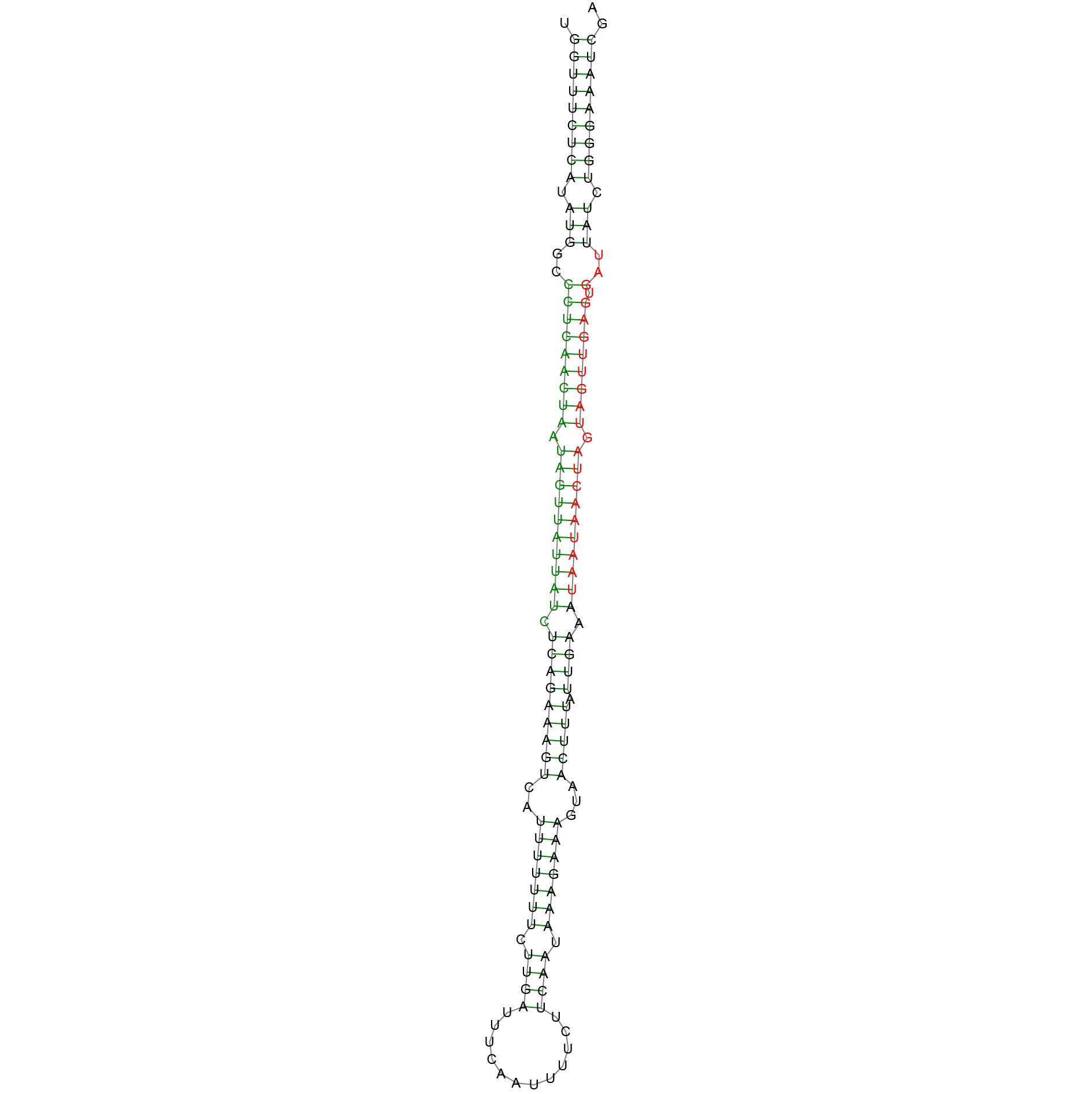

Supplement: Dataset S2 — Full list of hairpin structures in novel miRNAs. (ZIP) [file pone.0064238.s002.zip › can-miR-n019c.jpg]

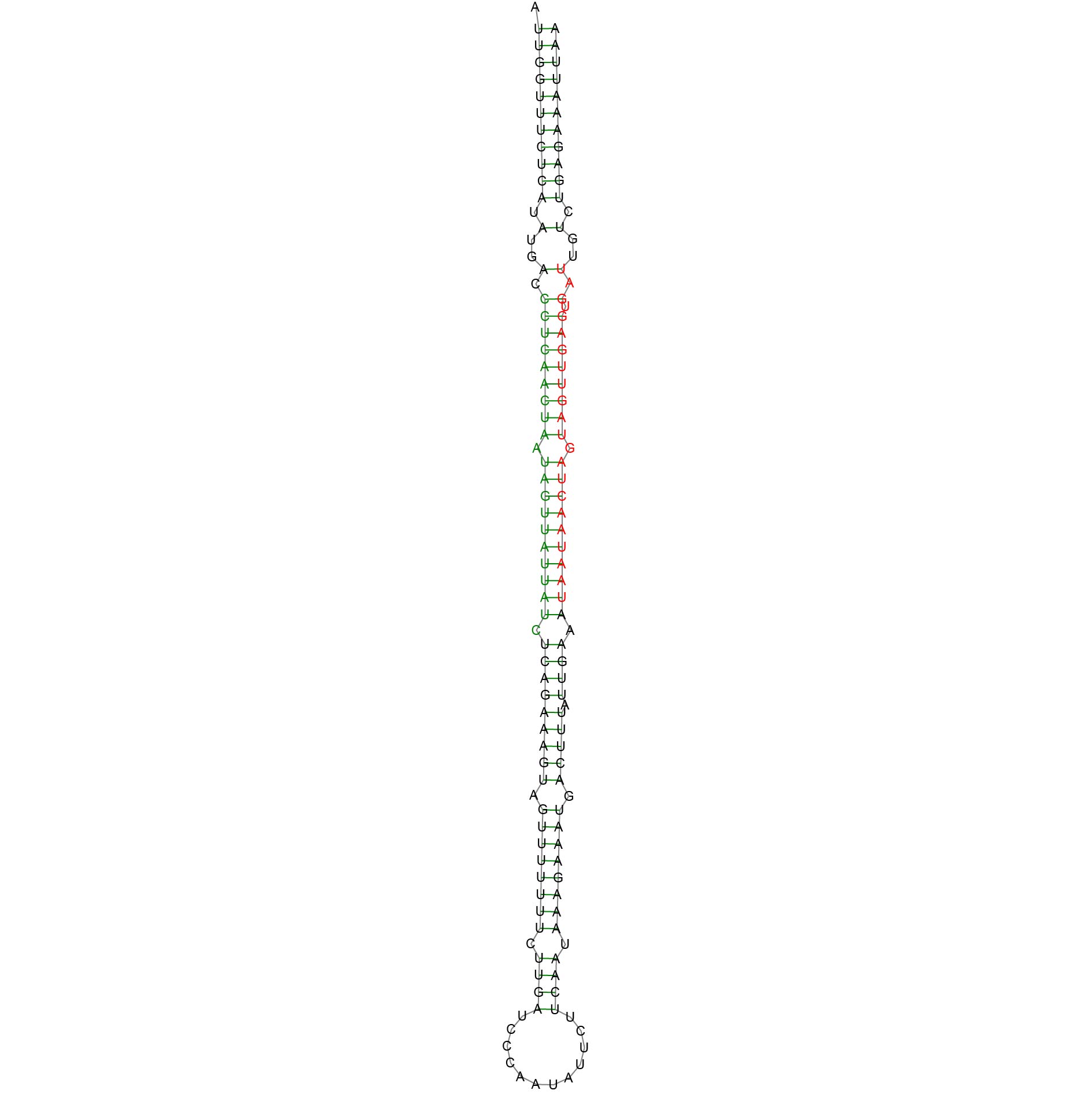

Supplement: Dataset S2 — Full list of hairpin structures in novel miRNAs. (ZIP) [file pone.0064238.s002.zip › can-miR-n019d.jpg]

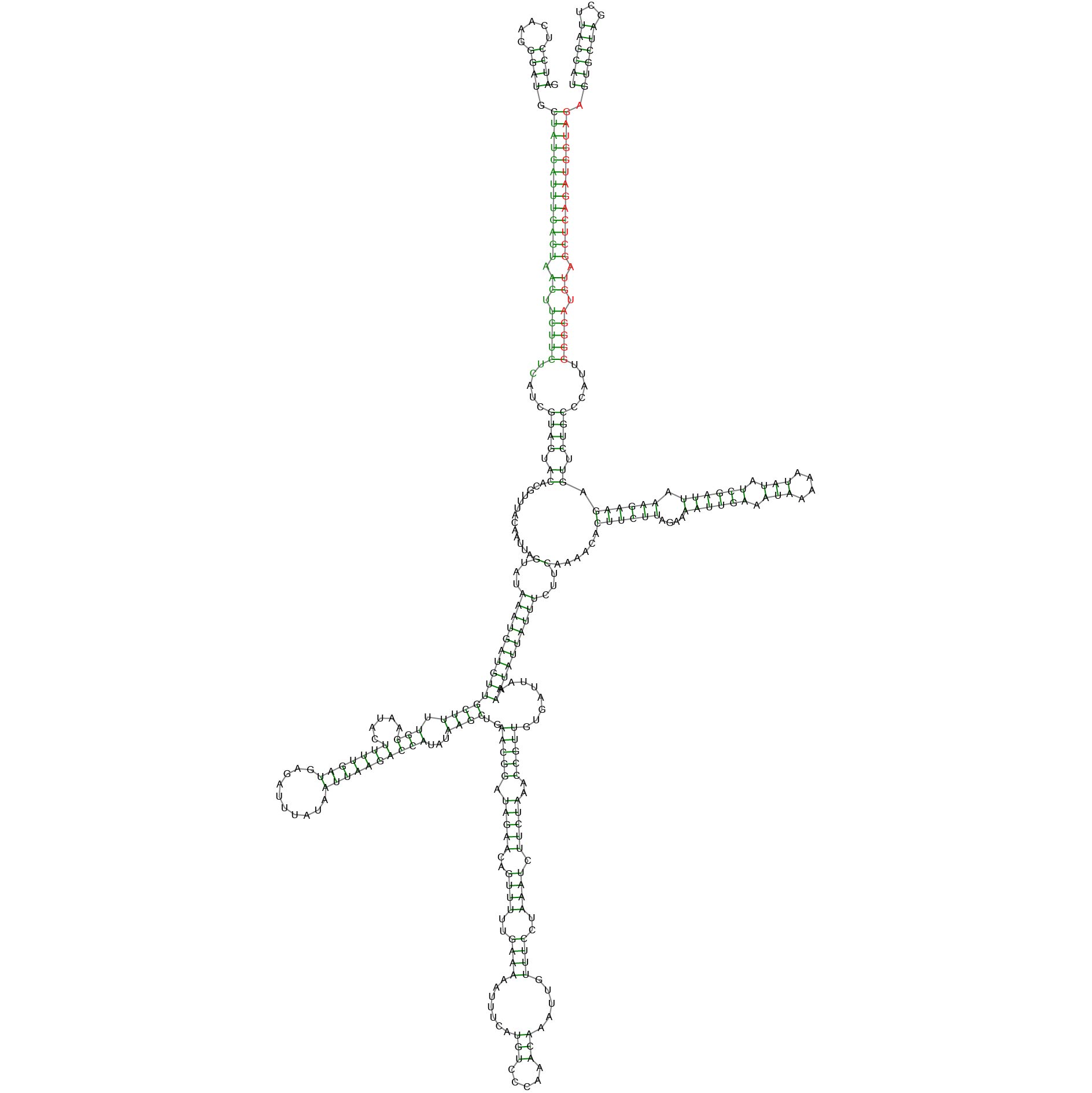

Supplement: Dataset S2 — Full list of hairpin structures in novel miRNAs. (ZIP) [file pone.0064238.s002.zip › can-miR-n020.jpg]

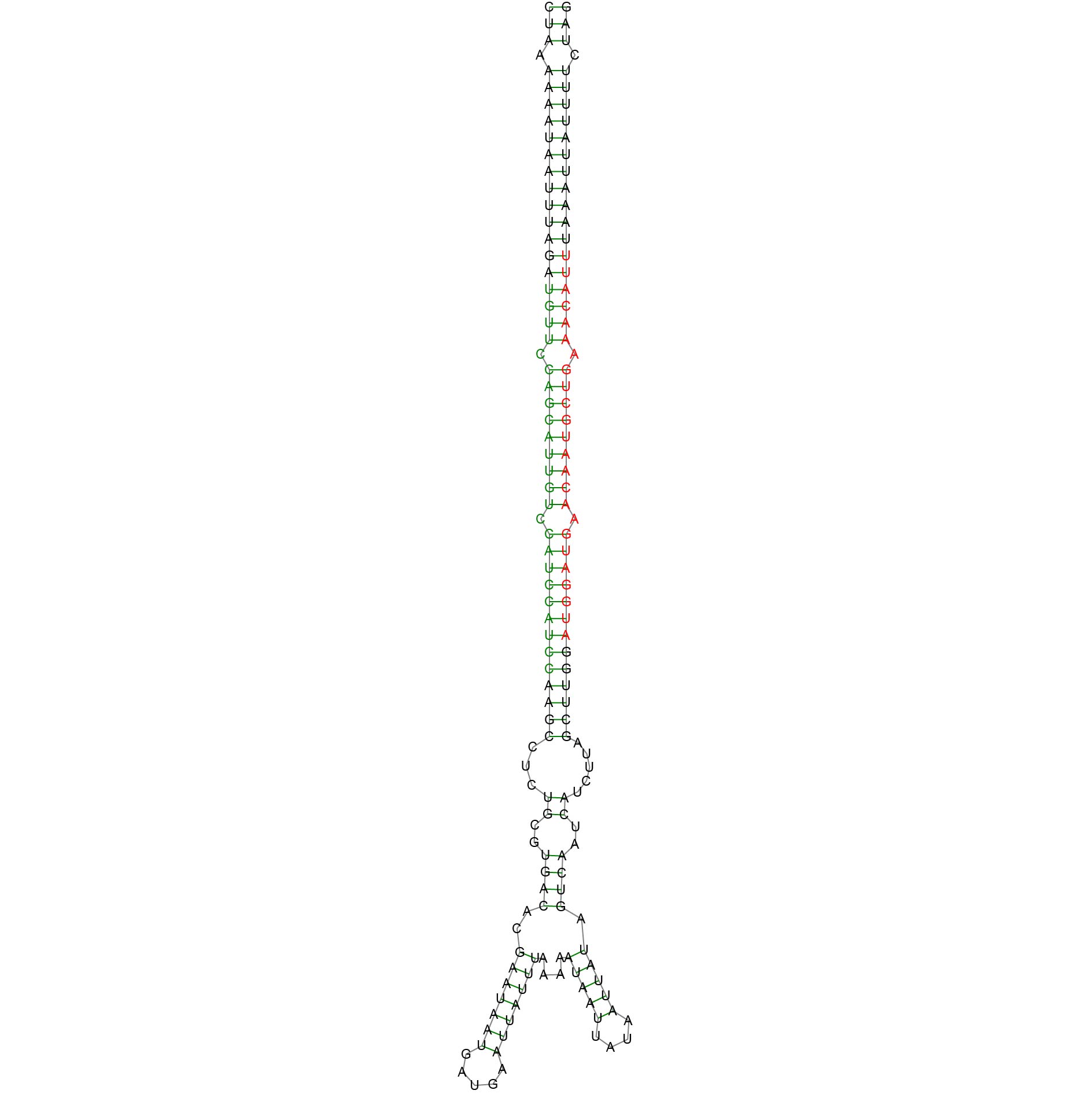

Supplement: Dataset S2 — Full list of hairpin structures in novel miRNAs. (ZIP) [file pone.0064238.s002.zip › can-miR-n021.jpg]

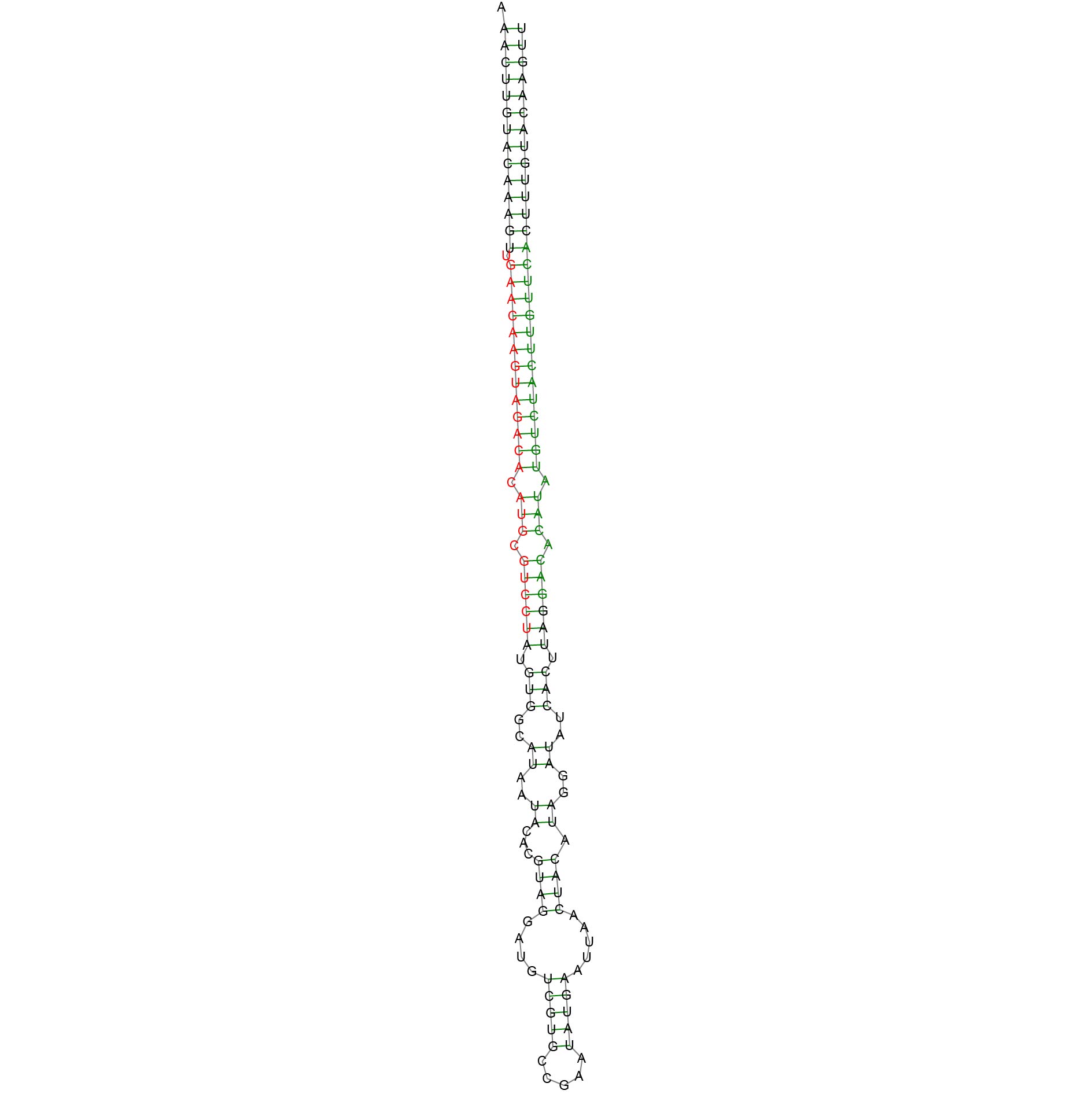

Supplement: Dataset S2 — Full list of hairpin structures in novel miRNAs. (ZIP) [file pone.0064238.s002.zip › can-miR-n022a.jpg]

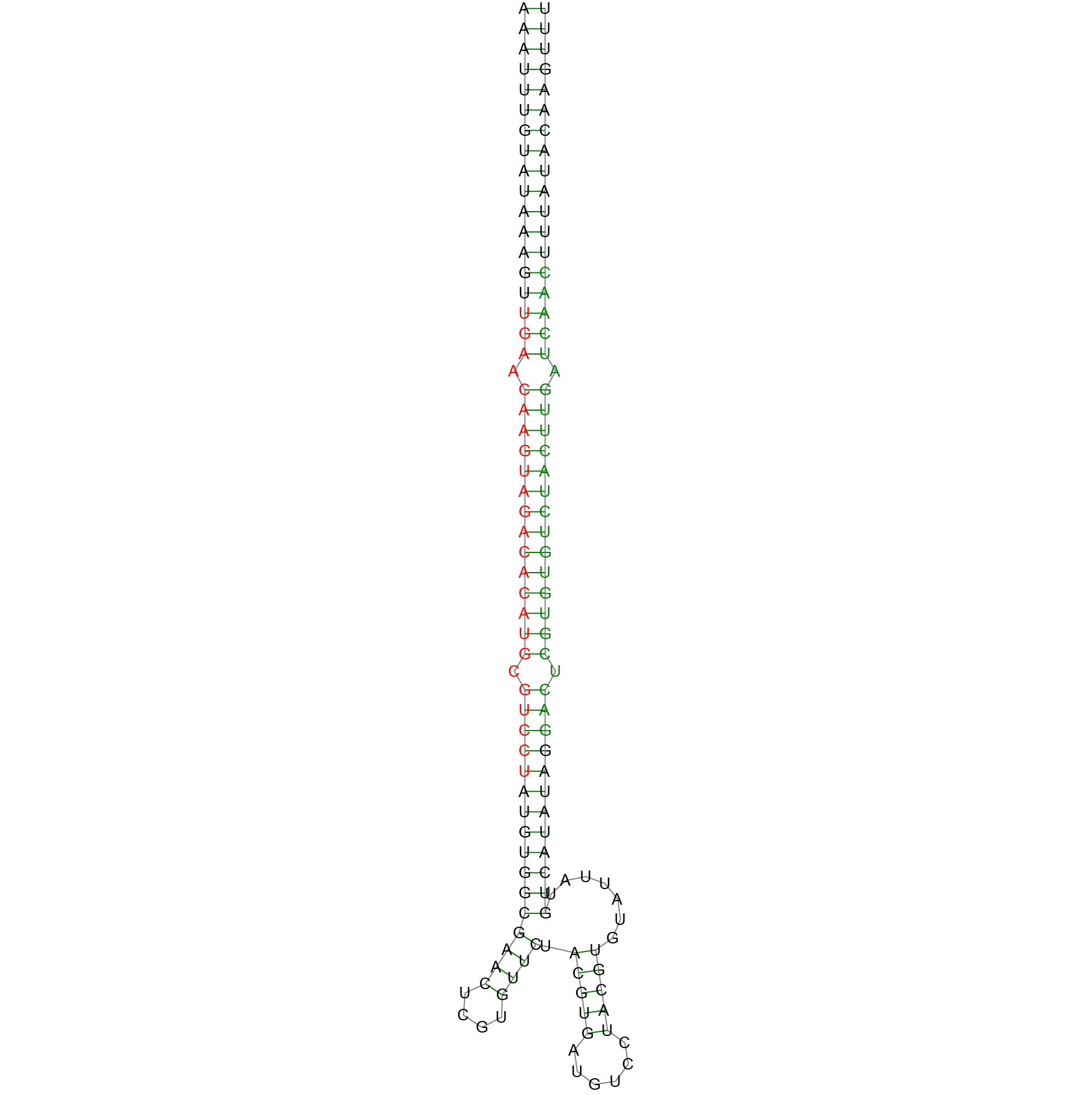

Supplement: Dataset S2 — Full list of hairpin structures in novel miRNAs. (ZIP) [file pone.0064238.s002.zip › can-miR-n022b.jpg]

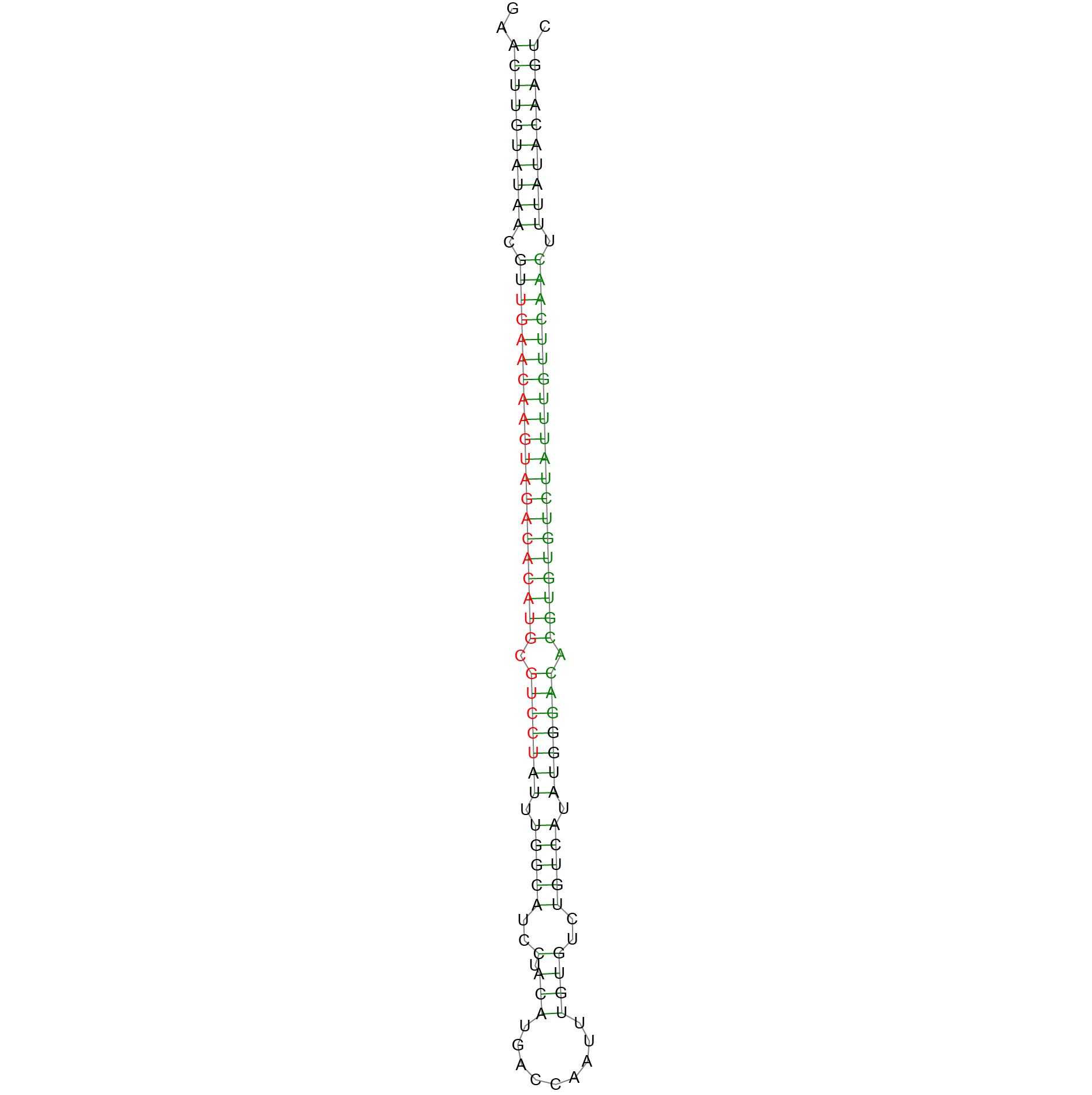

Supplement: Dataset S2 — Full list of hairpin structures in novel miRNAs. (ZIP) [file pone.0064238.s002.zip › can-miR-n022c.jpg]

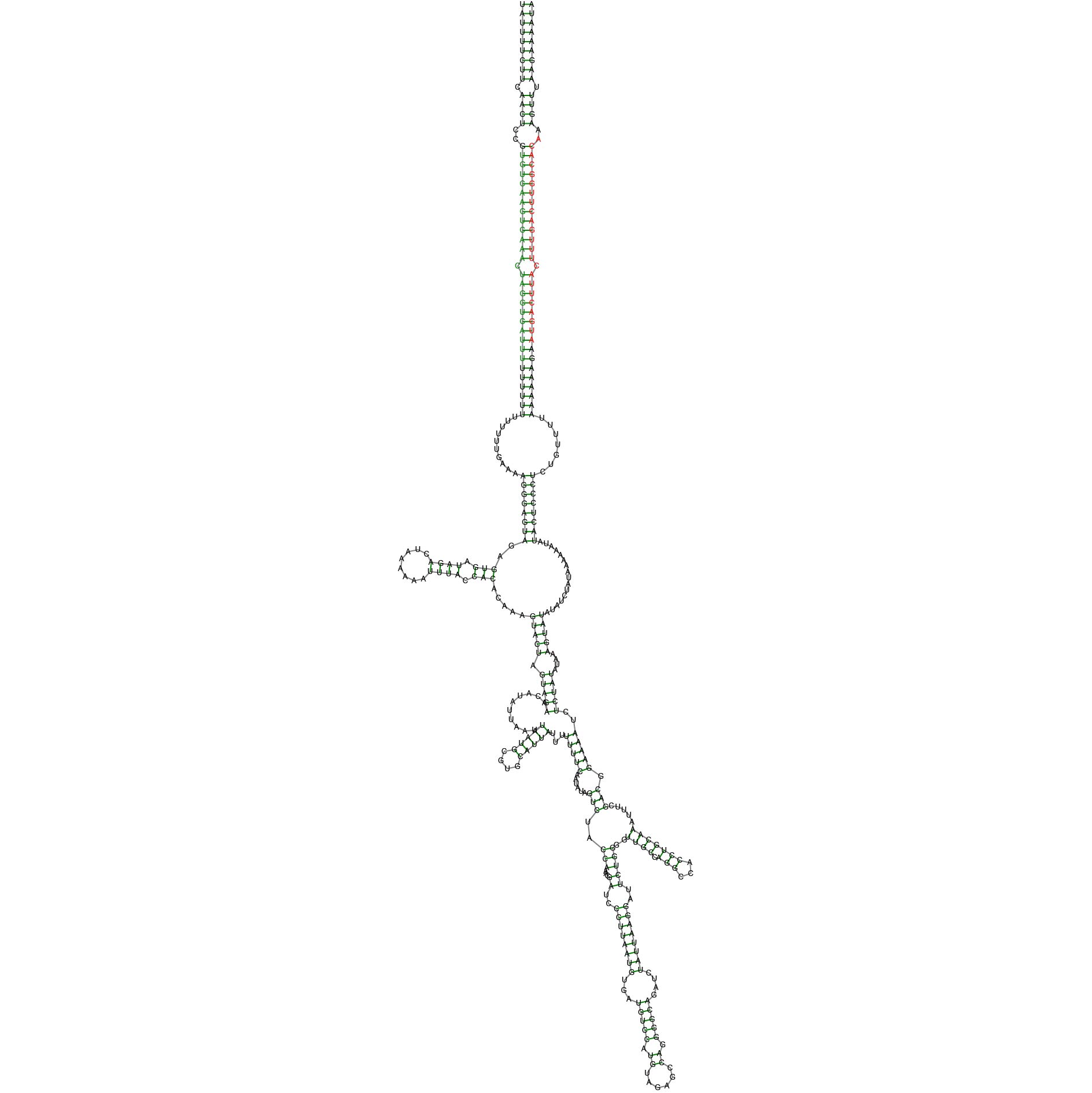

Supplement: Dataset S2 — Full list of hairpin structures in novel miRNAs. (ZIP) [file pone.0064238.s002.zip › can-miR-n023.jpg]

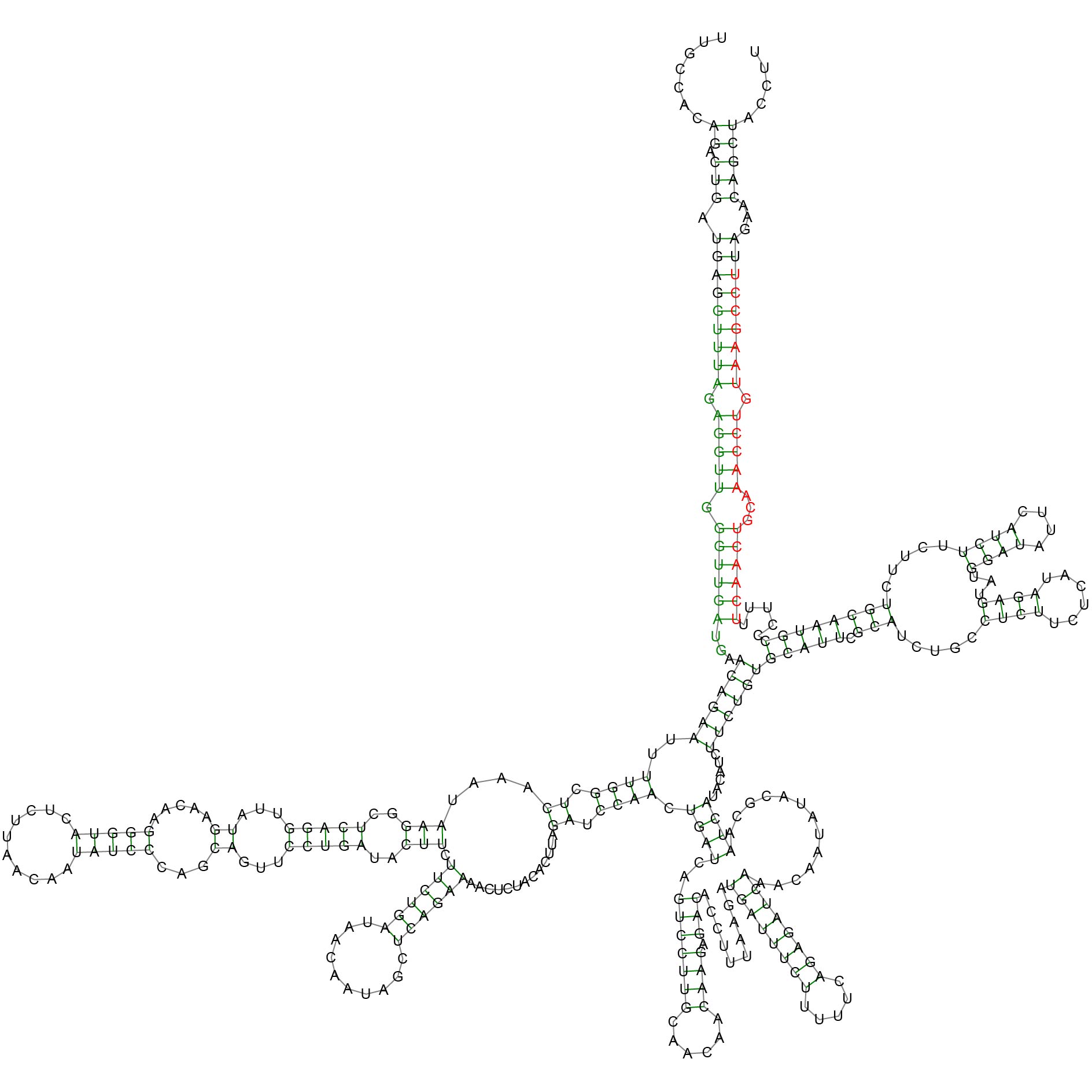

Supplement: Dataset S2 — Full list of hairpin structures in novel miRNAs. (ZIP) [file pone.0064238.s002.zip › can-miR-n024.jpg]

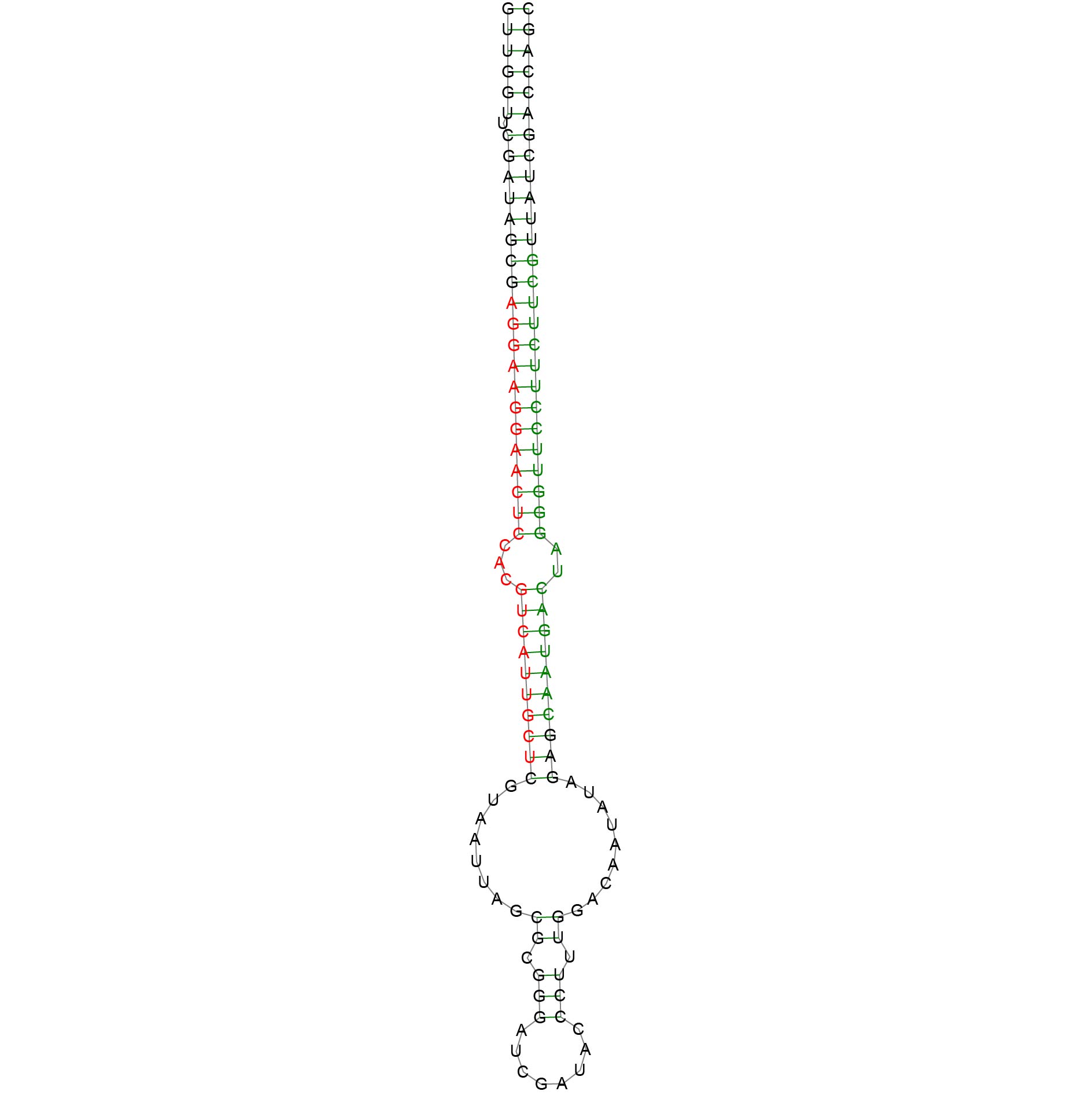

Supplement: Dataset S2 — Full list of hairpin structures in novel miRNAs. (ZIP) [file pone.0064238.s002.zip › can-miR-n025.jpg]

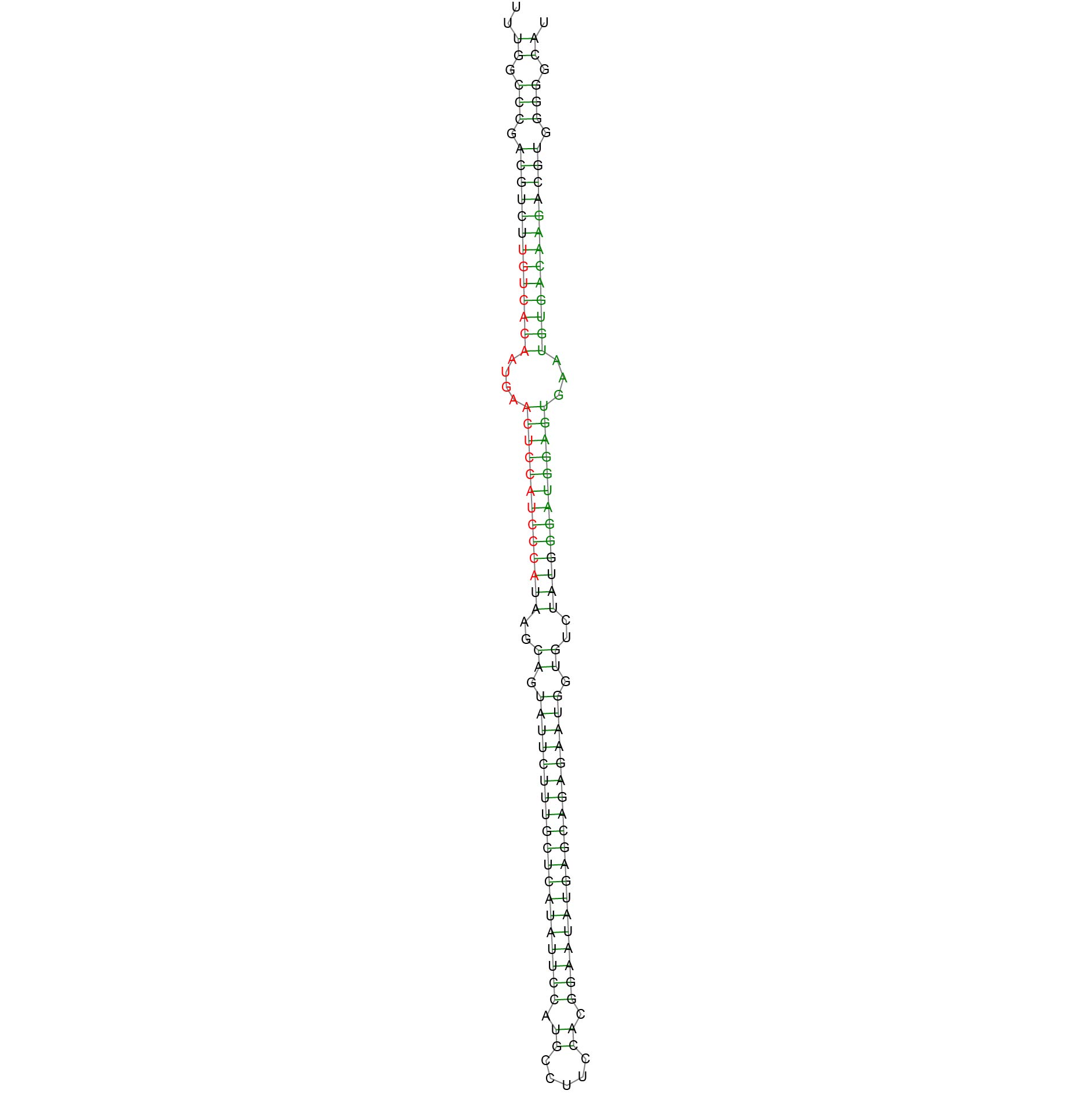

Supplement: Dataset S2 — Full list of hairpin structures in novel miRNAs. (ZIP) [file pone.0064238.s002.zip › can-miR-n026.jpg]

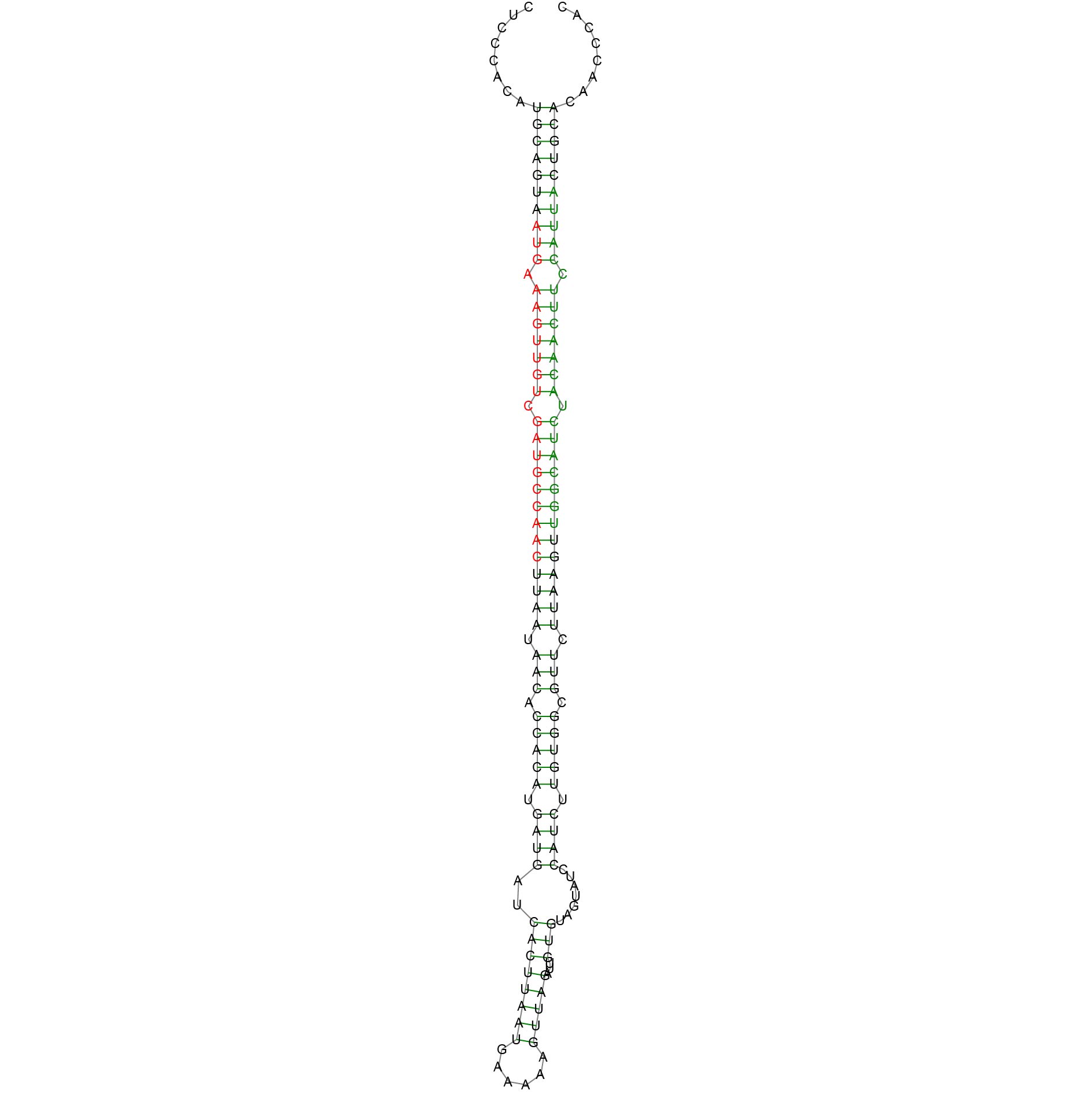

Supplement: Dataset S2 — Full list of hairpin structures in novel miRNAs. (ZIP) [file pone.0064238.s002.zip › can-miR-n027.jpg]

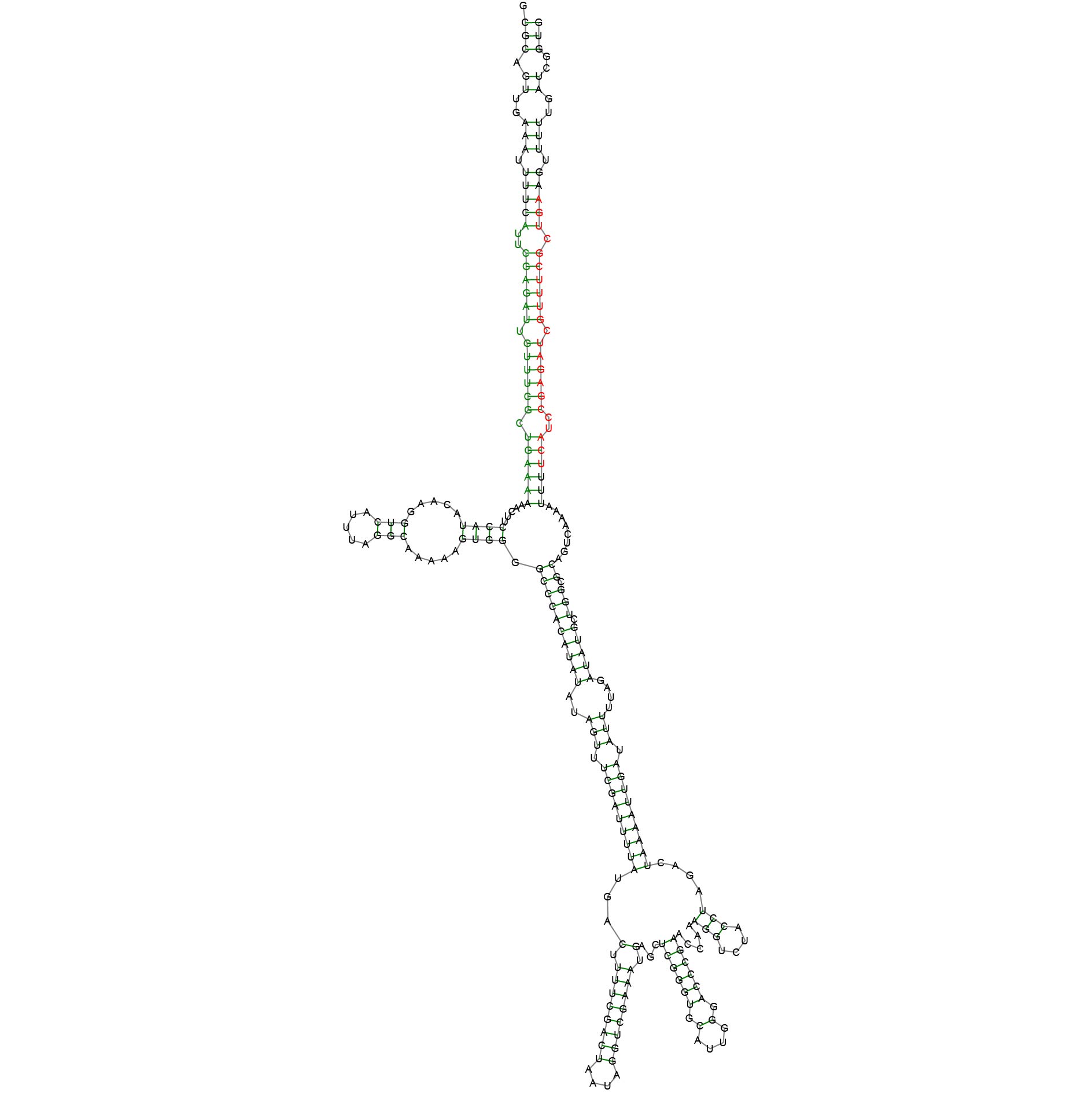

Supplement: Dataset S2 — Full list of hairpin structures in novel miRNAs. (ZIP) [file pone.0064238.s002.zip › can-miR-n028a.jpg]

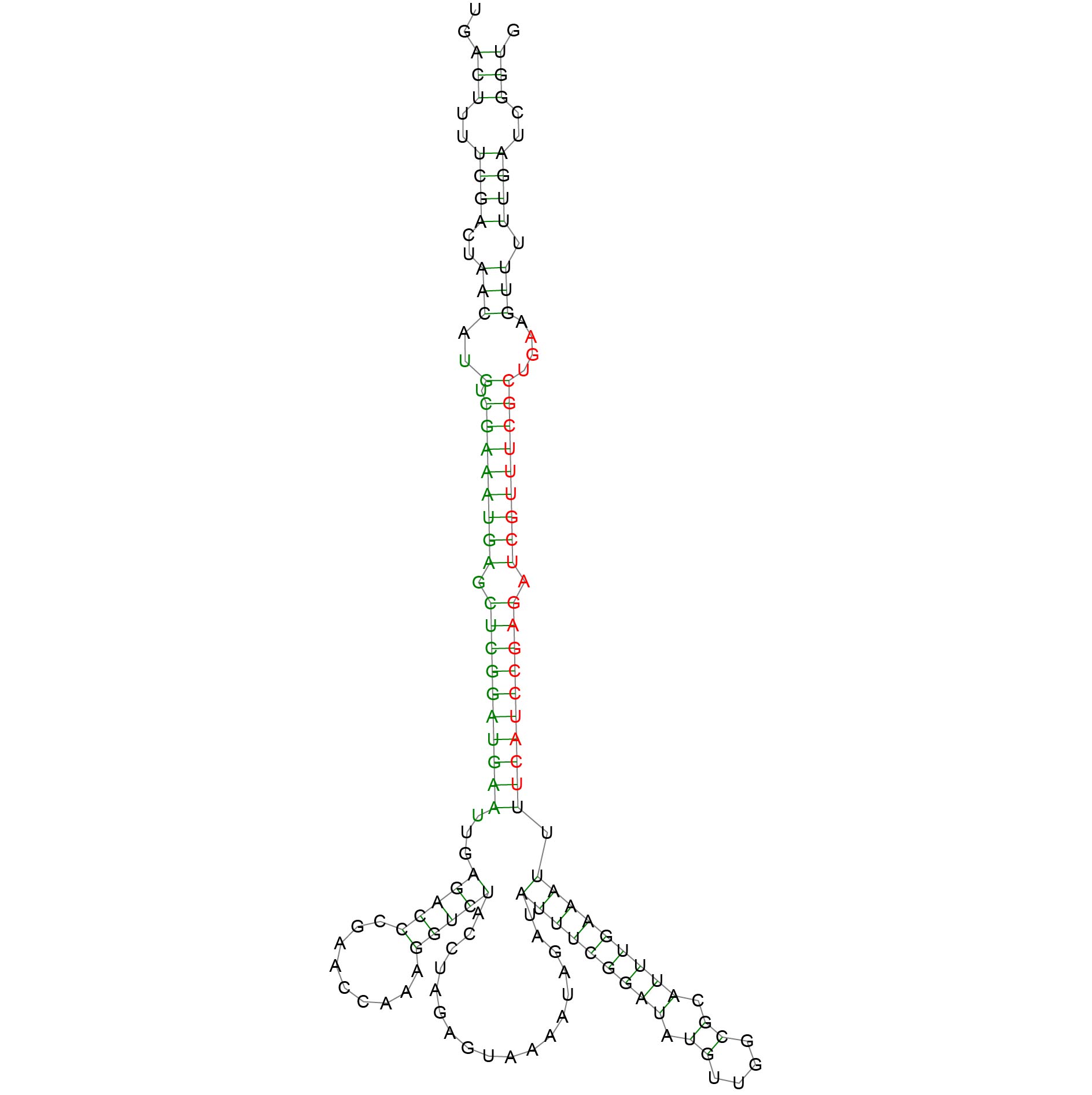

Supplement: Dataset S2 — Full list of hairpin structures in novel miRNAs. (ZIP) [file pone.0064238.s002.zip › can-miR-n028b.jpg]

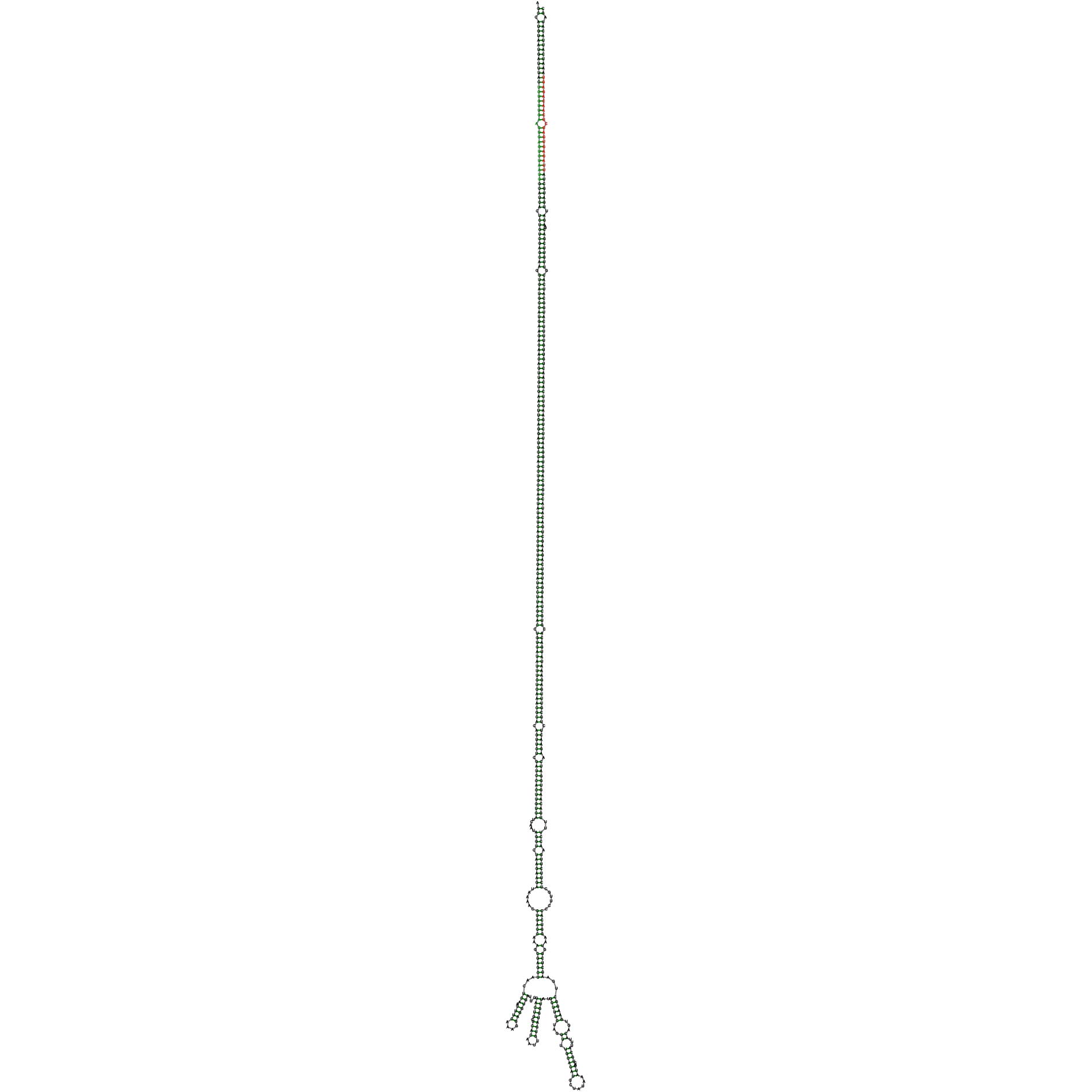

Supplement: Dataset S2 — Full list of hairpin structures in novel miRNAs. (ZIP) [file pone.0064238.s002.zip › can-miR-n029.jpg]

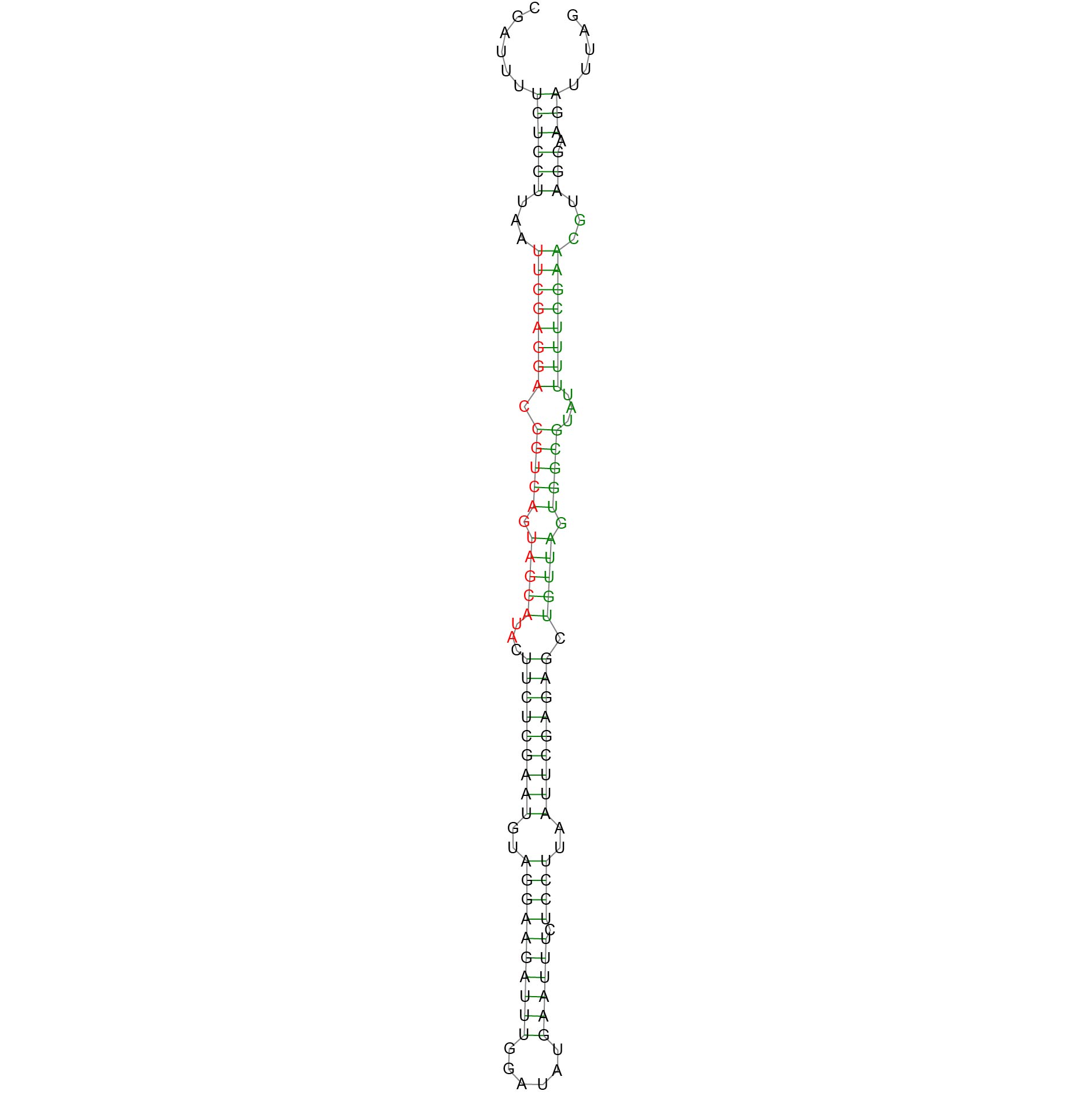

Supplement: Dataset S2 — Full list of hairpin structures in novel miRNAs. (ZIP) [file pone.0064238.s002.zip › can-miR-n030.jpg]

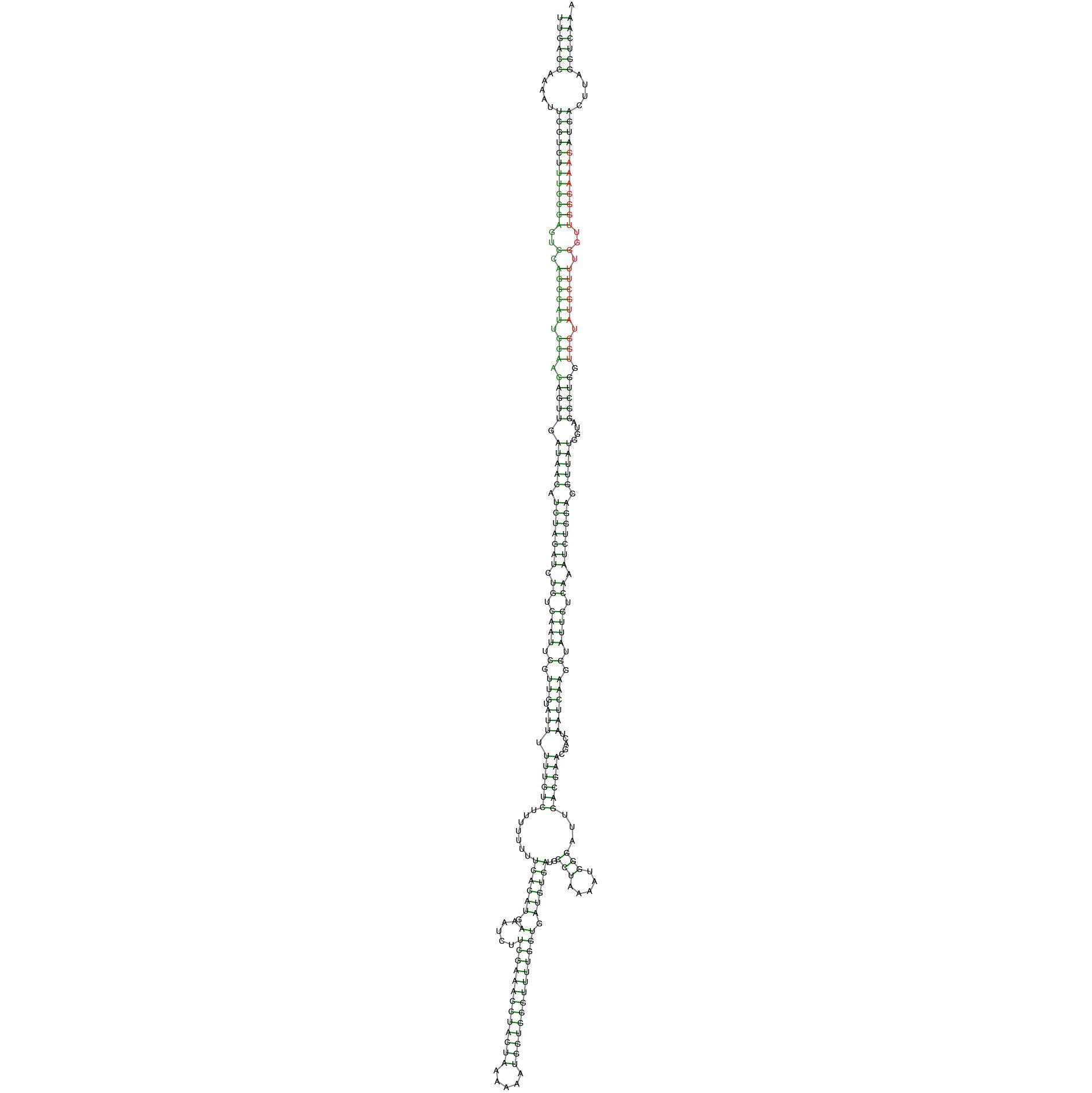

Supplement: Dataset S2 — Full list of hairpin structures in novel miRNAs. (ZIP) [file pone.0064238.s002.zip › can-miR-n031.jpg]

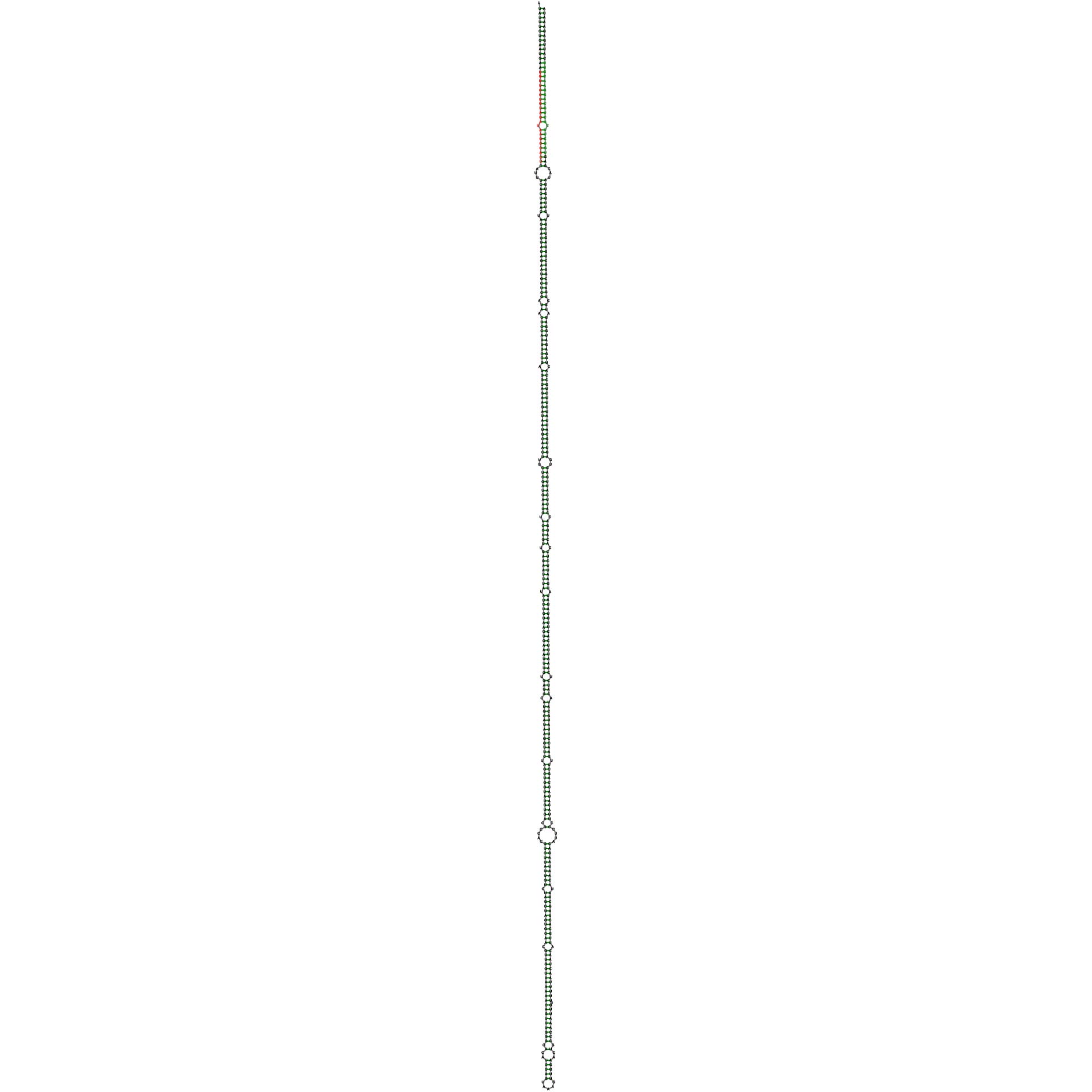

Supplement: Dataset S2 — Full list of hairpin structures in novel miRNAs. (ZIP) [file pone.0064238.s002.zip › can-miR-n032.jpg]

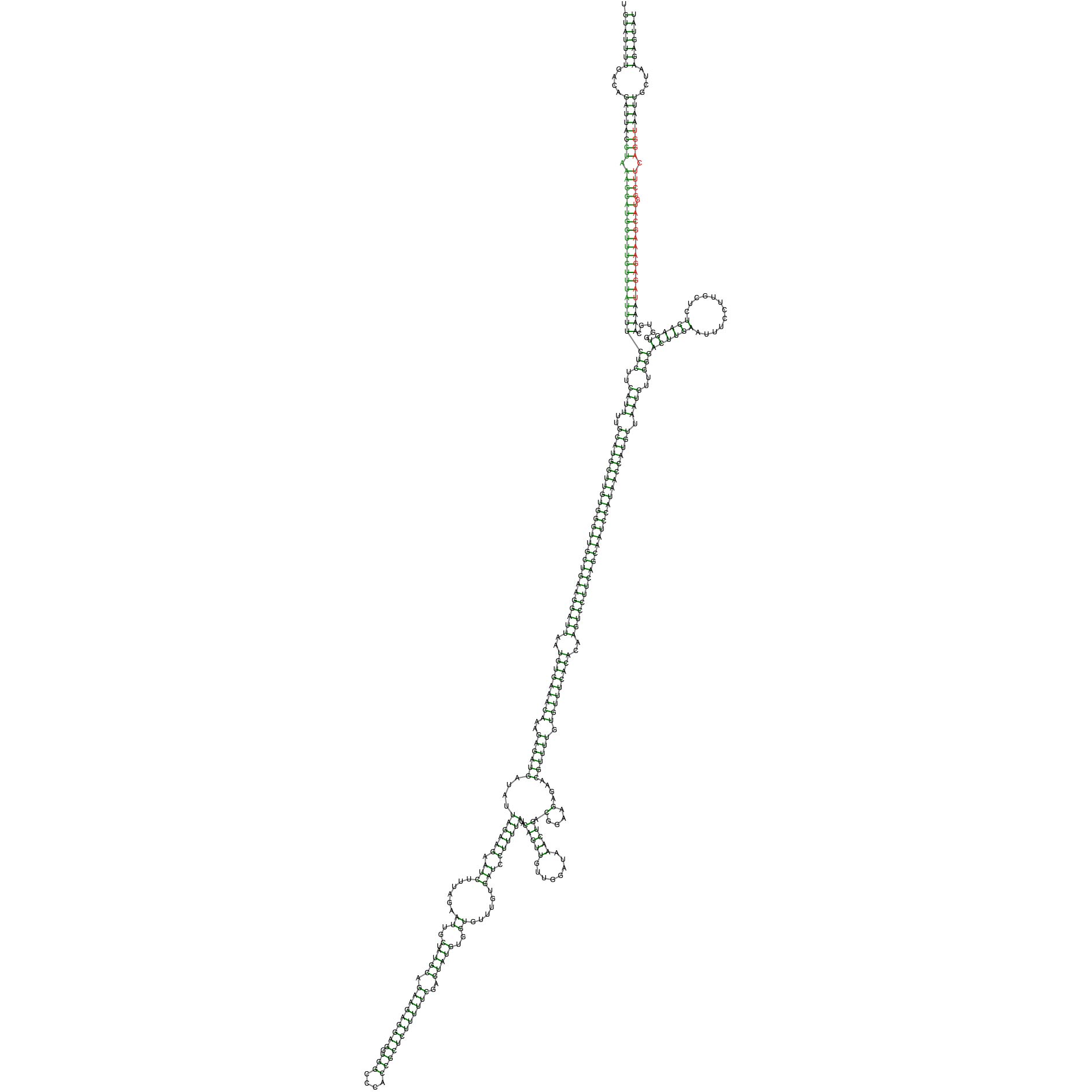

Supplement: Dataset S2 — Full list of hairpin structures in novel miRNAs. (ZIP) [file pone.0064238.s002.zip › can-miR-n033.jpg]

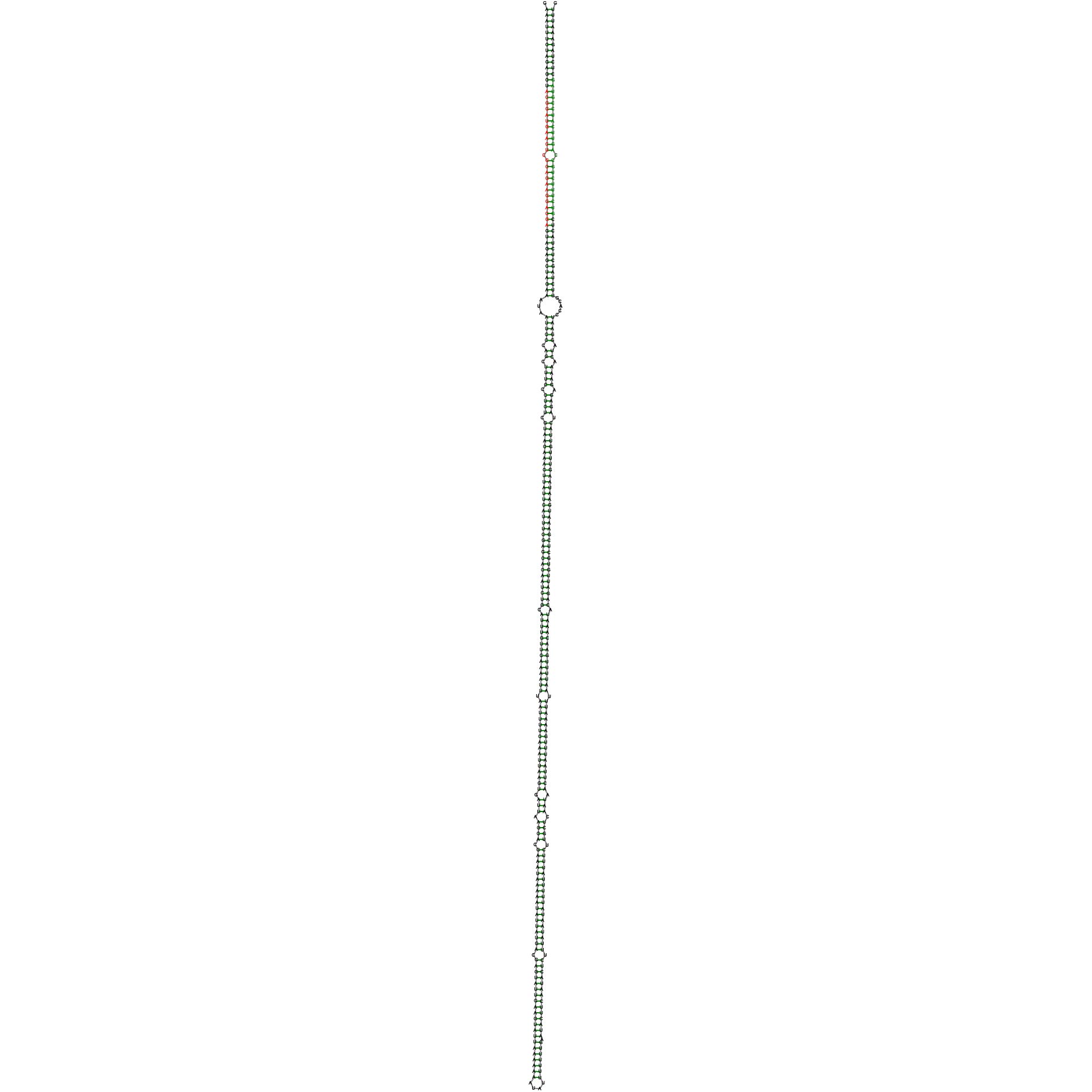

Supplement: Dataset S2 — Full list of hairpin structures in novel miRNAs. (ZIP) [file pone.0064238.s002.zip › can-miR-n034.jpg]

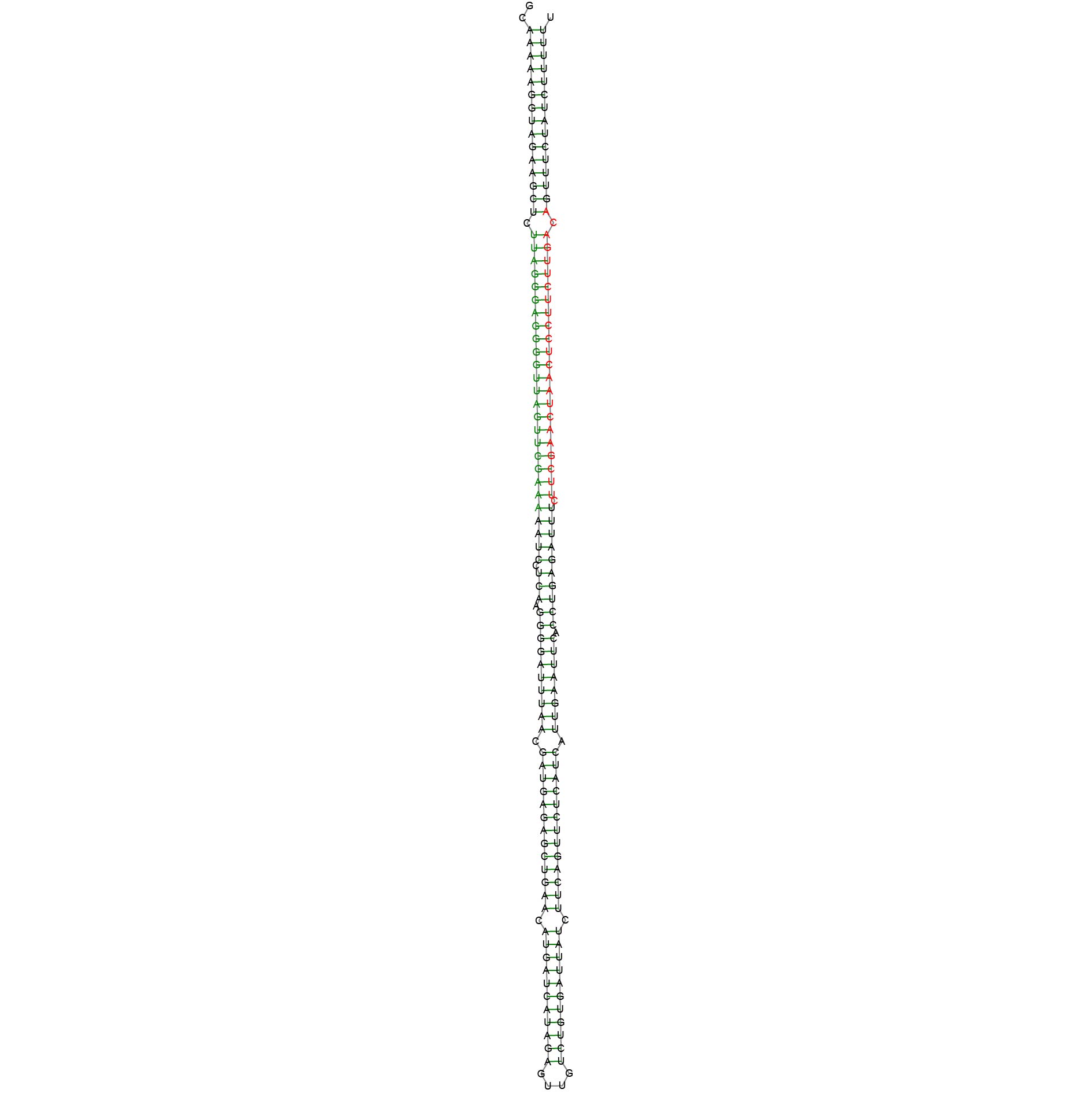

Supplement: Dataset S2 — Full list of hairpin structures in novel miRNAs. (ZIP) [file pone.0064238.s002.zip › can-miR-n035.jpg]

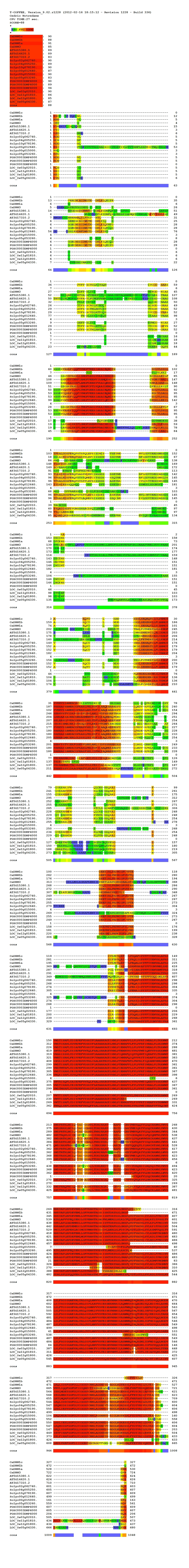

Supplement: Figure S1 — T-coffee alignment of DRM methyltransferase. (JPG) [file pone.0064238.s003.jpg]
