# Supplementary figures and images for: The Hot Pepper (Capsicum annuum) MicroRNA Transcriptome Reveals Novel and Conserved Targets: A Foundation for Understanding MicroRNA Functional Roles in Hot Pepper (part 1 of 2)
Source: PLoS One. 2013 May 30;8(5):e64238. doi: 10.1371/journal.pone.0064238 (PMC3667847; doi:10.1371/journal.pone.0064238)

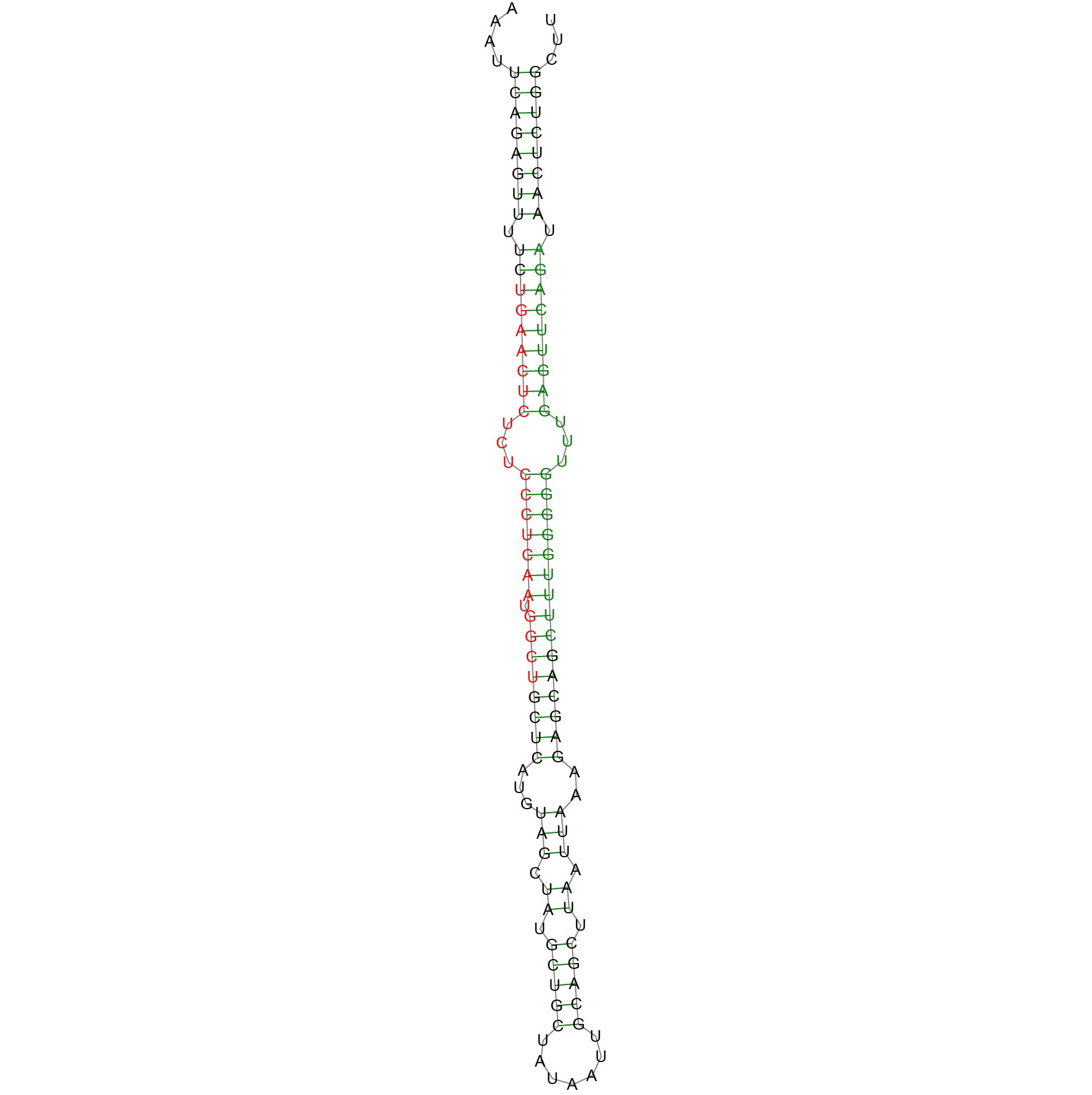

Supplement: Dataset S1 — Full list of hairpin structures in conserved miRNAs. (ZIP) [file pone.0064238.s001.zip › can-miR1446a.jpg]

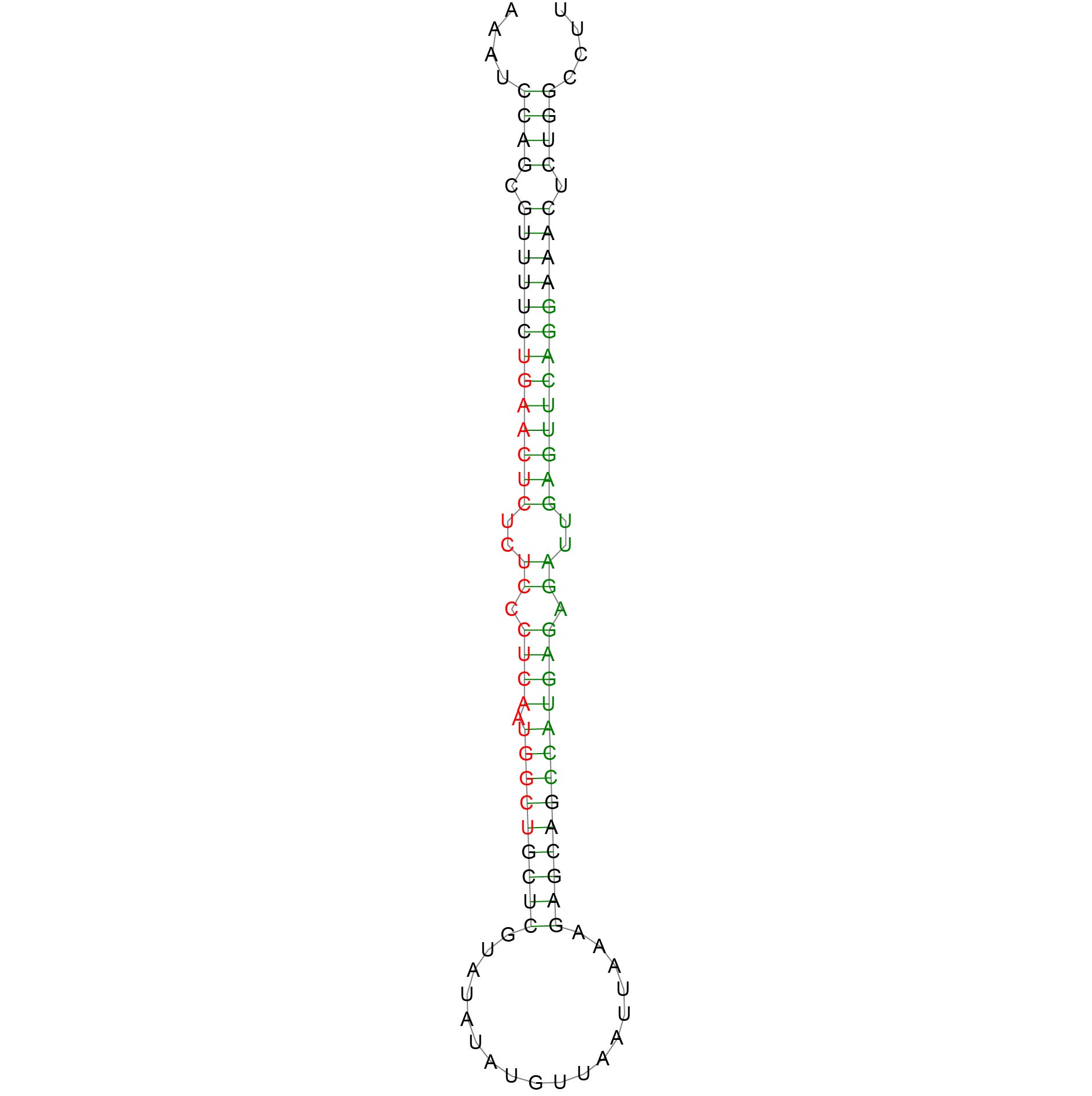

Supplement: Dataset S1 — Full list of hairpin structures in conserved miRNAs. (ZIP) [file pone.0064238.s001.zip › can-miR1446b.jpg]

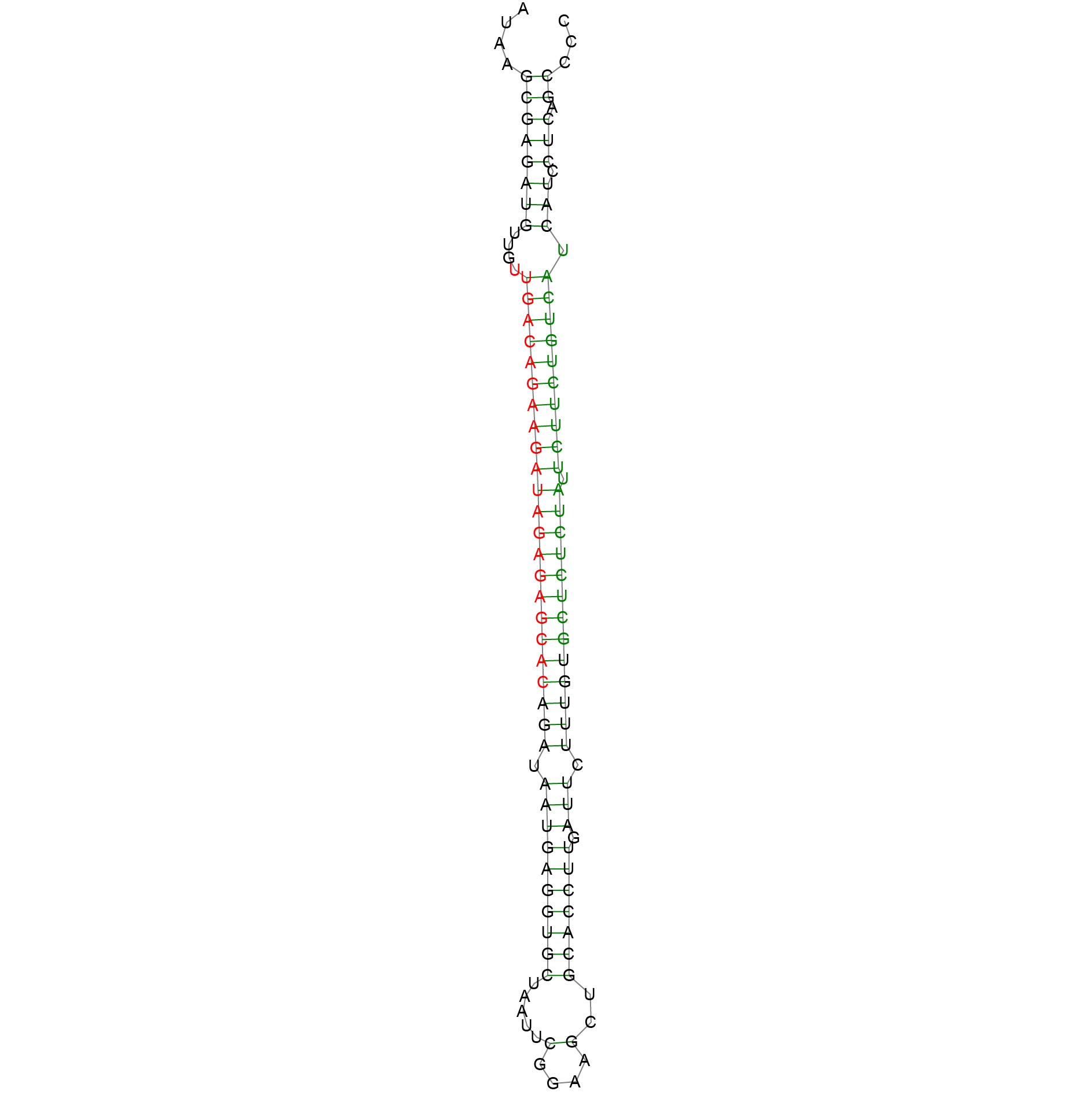

Supplement: Dataset S1 — Full list of hairpin structures in conserved miRNAs. (ZIP) [file pone.0064238.s001.zip › can-miR156a.jpg]

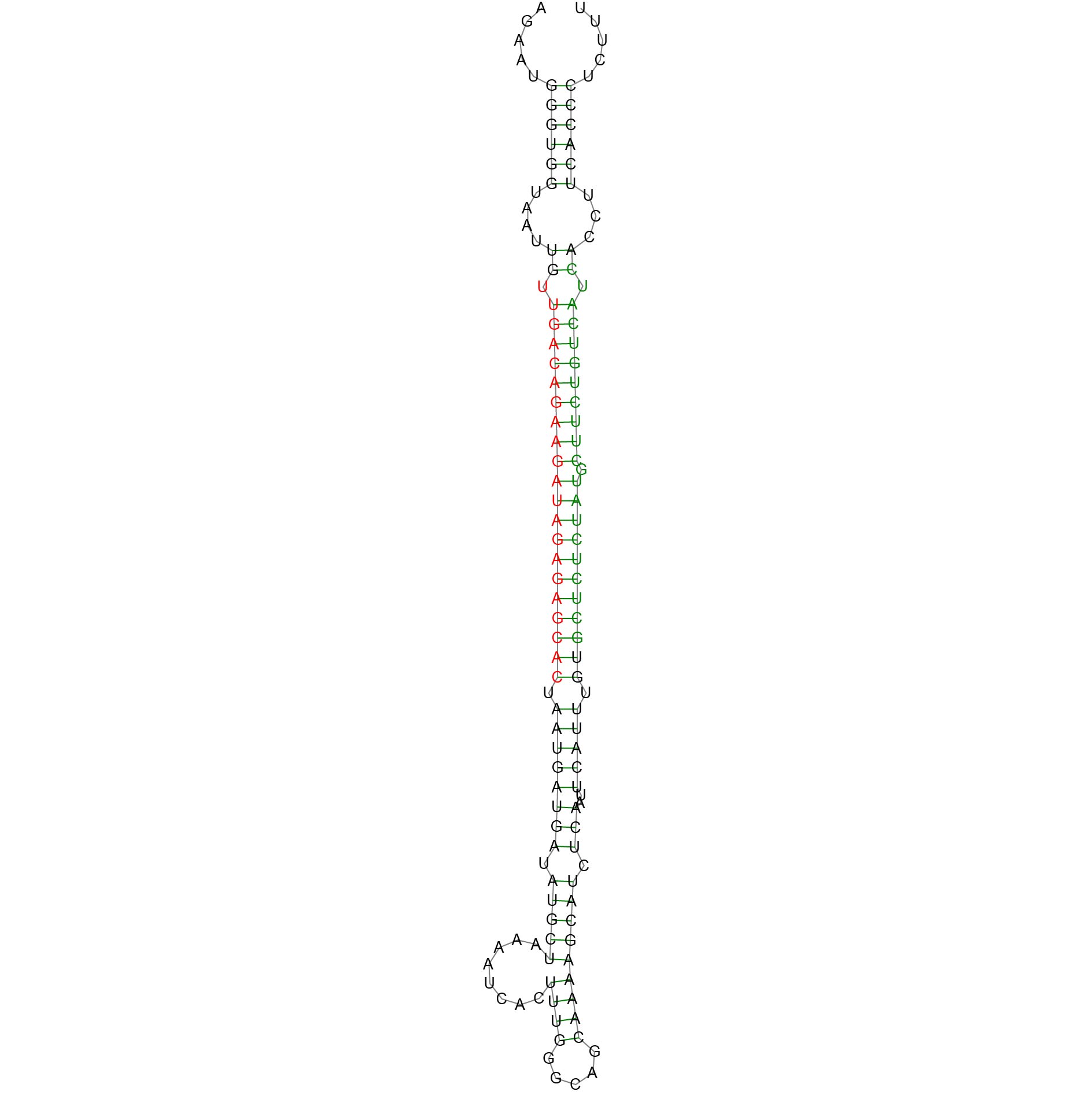

Supplement: Dataset S1 — Full list of hairpin structures in conserved miRNAs. (ZIP) [file pone.0064238.s001.zip › can-miR156b.jpg]

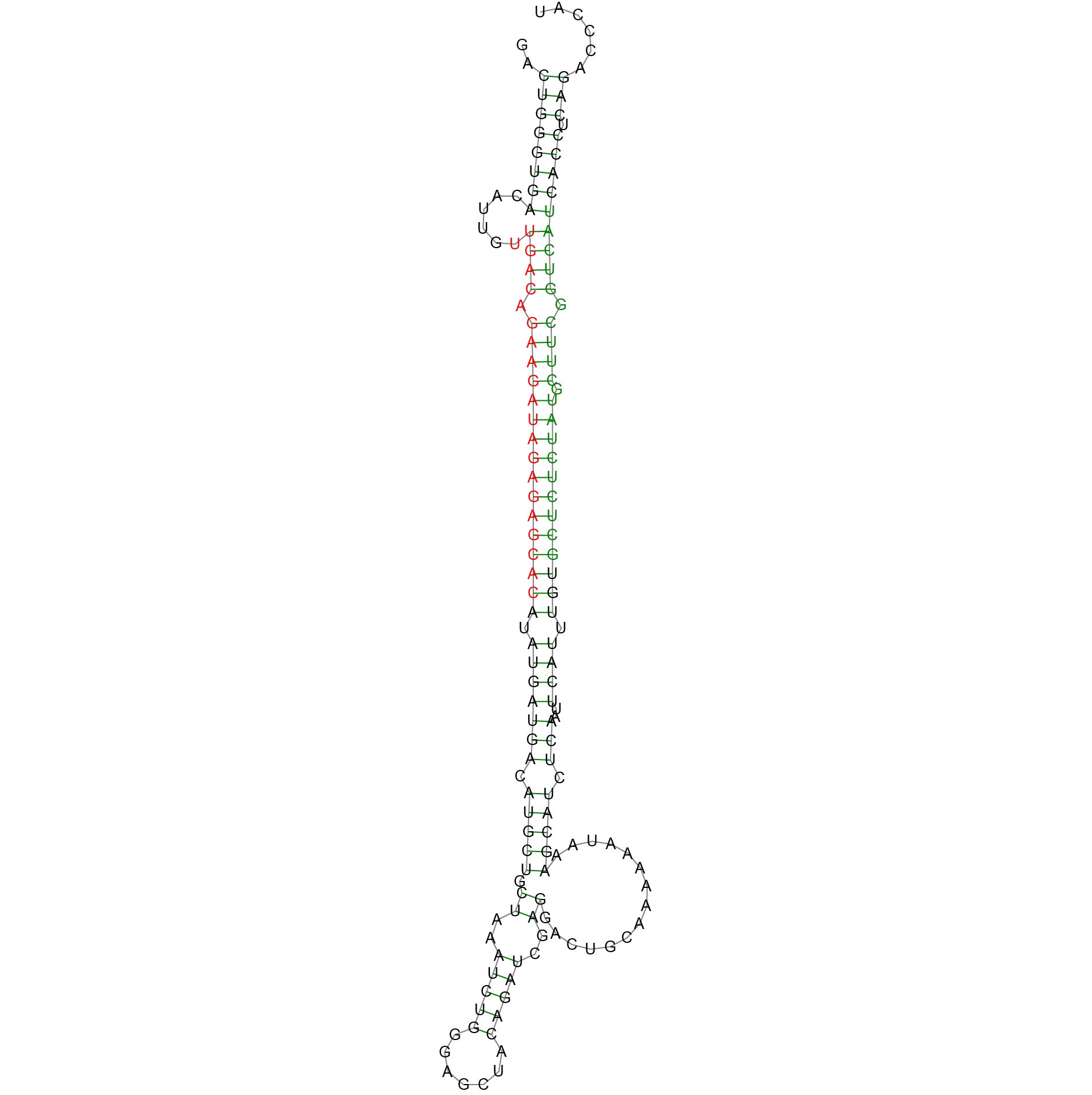

Supplement: Dataset S1 — Full list of hairpin structures in conserved miRNAs. (ZIP) [file pone.0064238.s001.zip › can-miR156c.jpg]

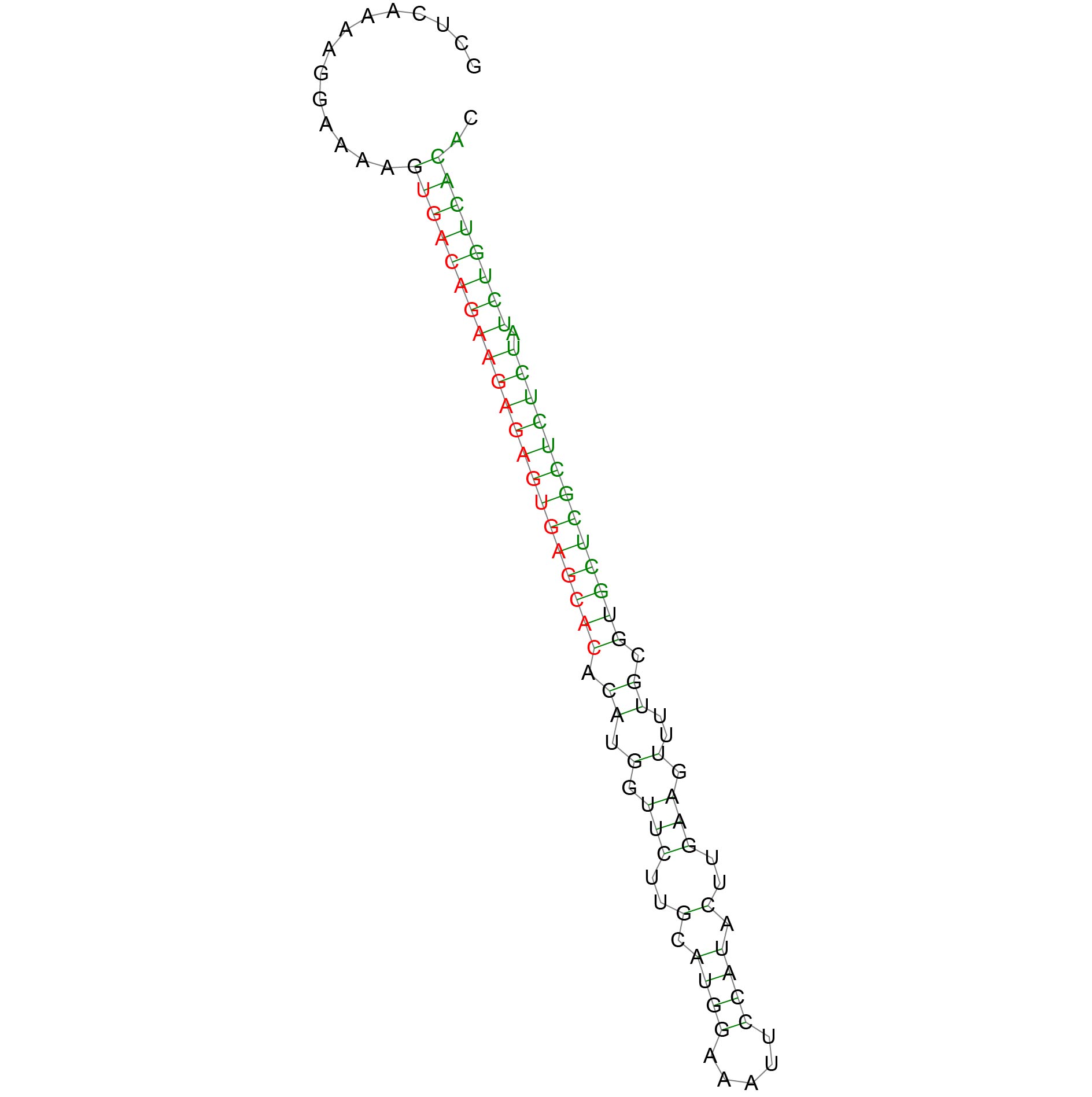

Supplement: Dataset S1 — Full list of hairpin structures in conserved miRNAs. (ZIP) [file pone.0064238.s001.zip › can-miR156d.jpg]

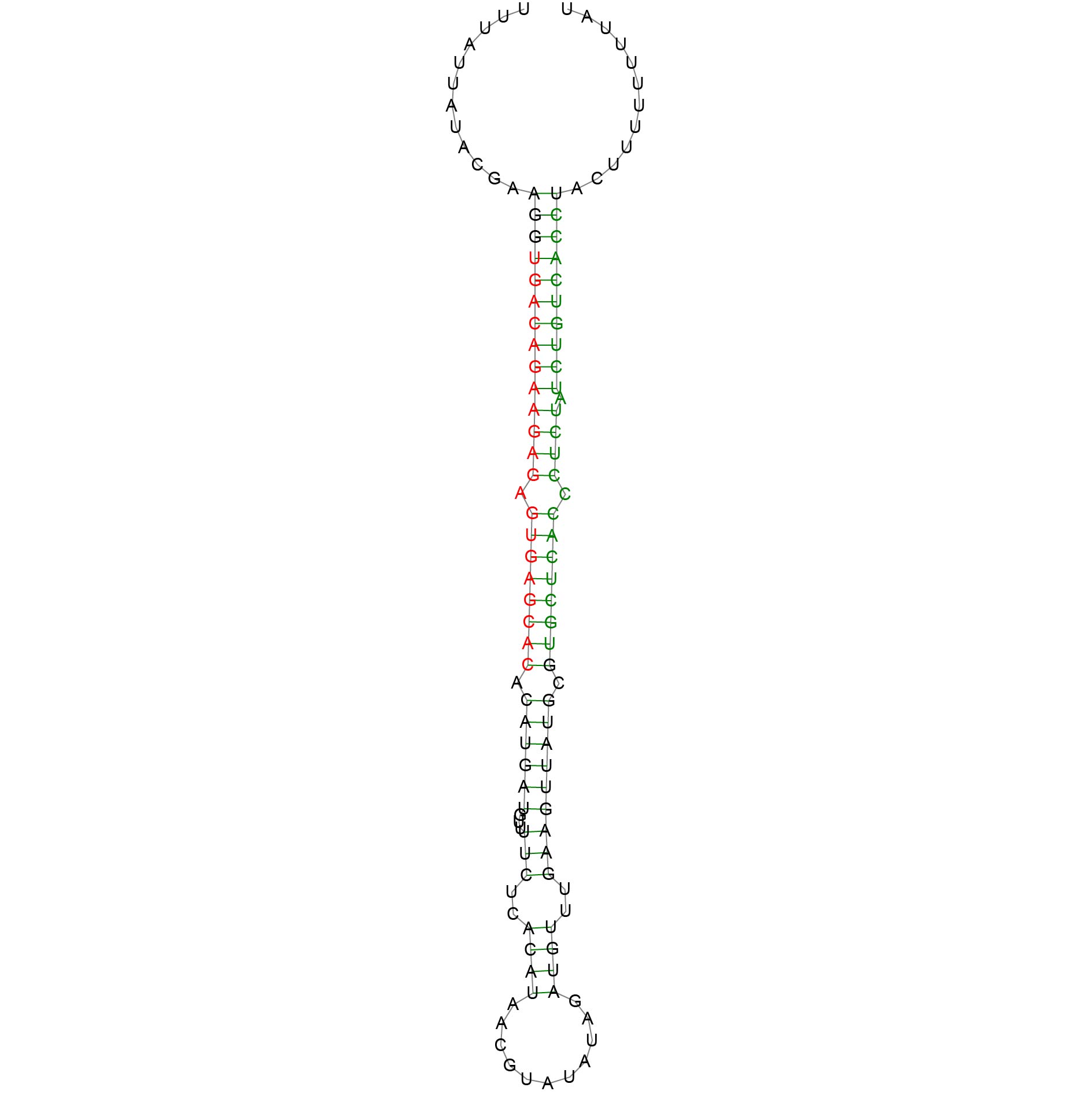

Supplement: Dataset S1 — Full list of hairpin structures in conserved miRNAs. (ZIP) [file pone.0064238.s001.zip › can-miR156e.jpg]

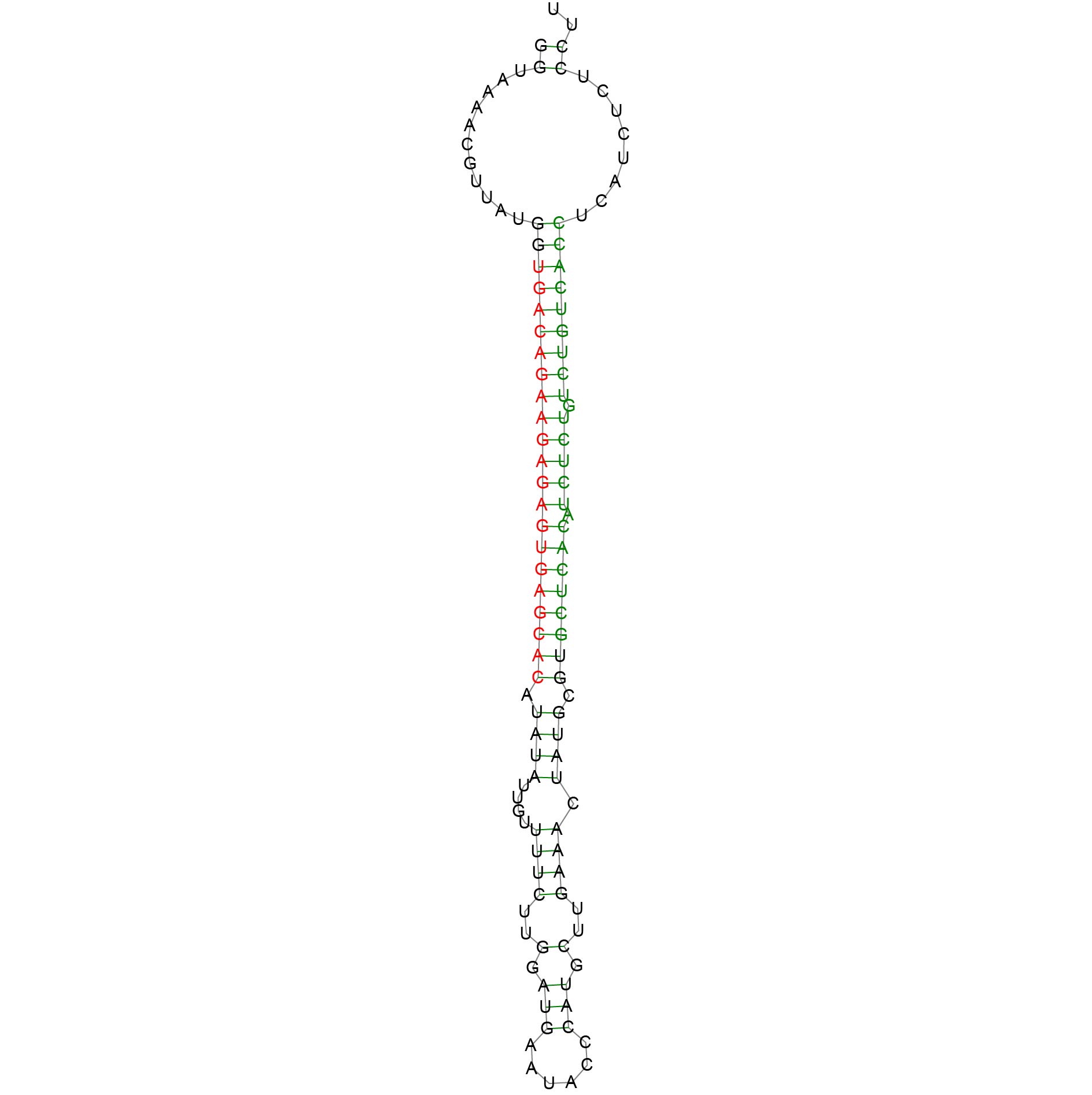

Supplement: Dataset S1 — Full list of hairpin structures in conserved miRNAs. (ZIP) [file pone.0064238.s001.zip › can-miR156f.jpg]

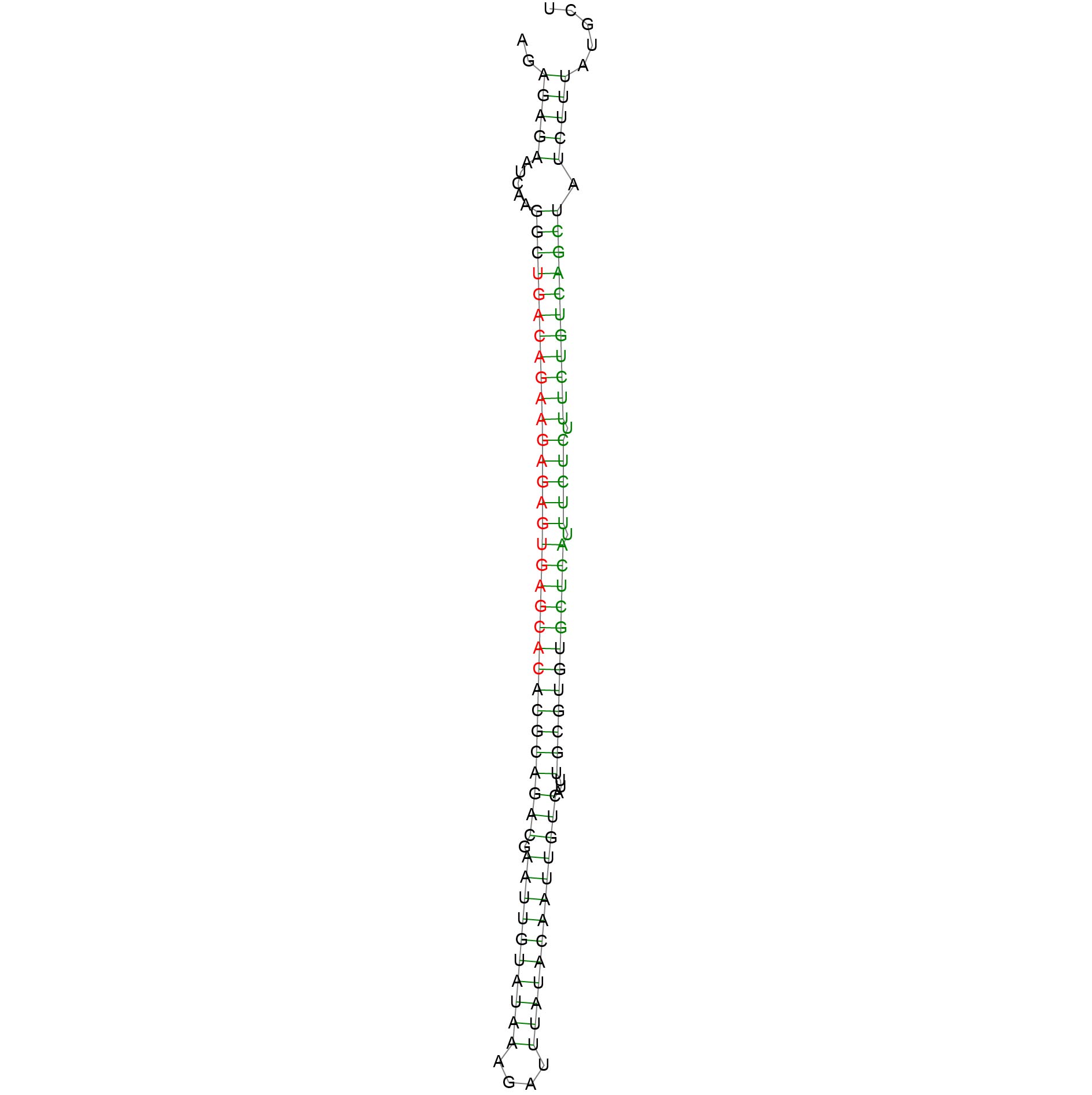

Supplement: Dataset S1 — Full list of hairpin structures in conserved miRNAs. (ZIP) [file pone.0064238.s001.zip › can-miR156g.jpg]

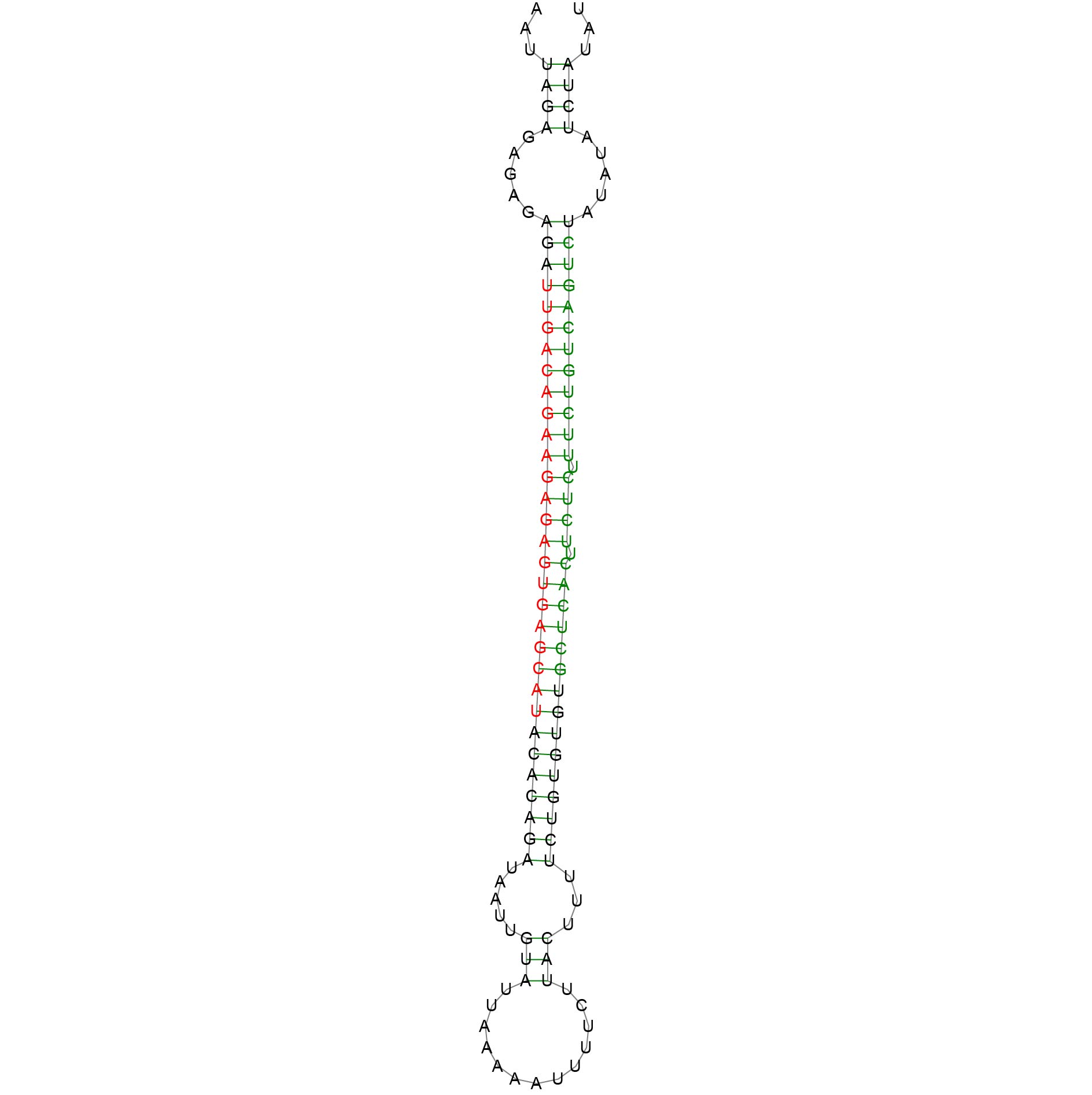

Supplement: Dataset S1 — Full list of hairpin structures in conserved miRNAs. (ZIP) [file pone.0064238.s001.zip › can-miR156h.jpg]

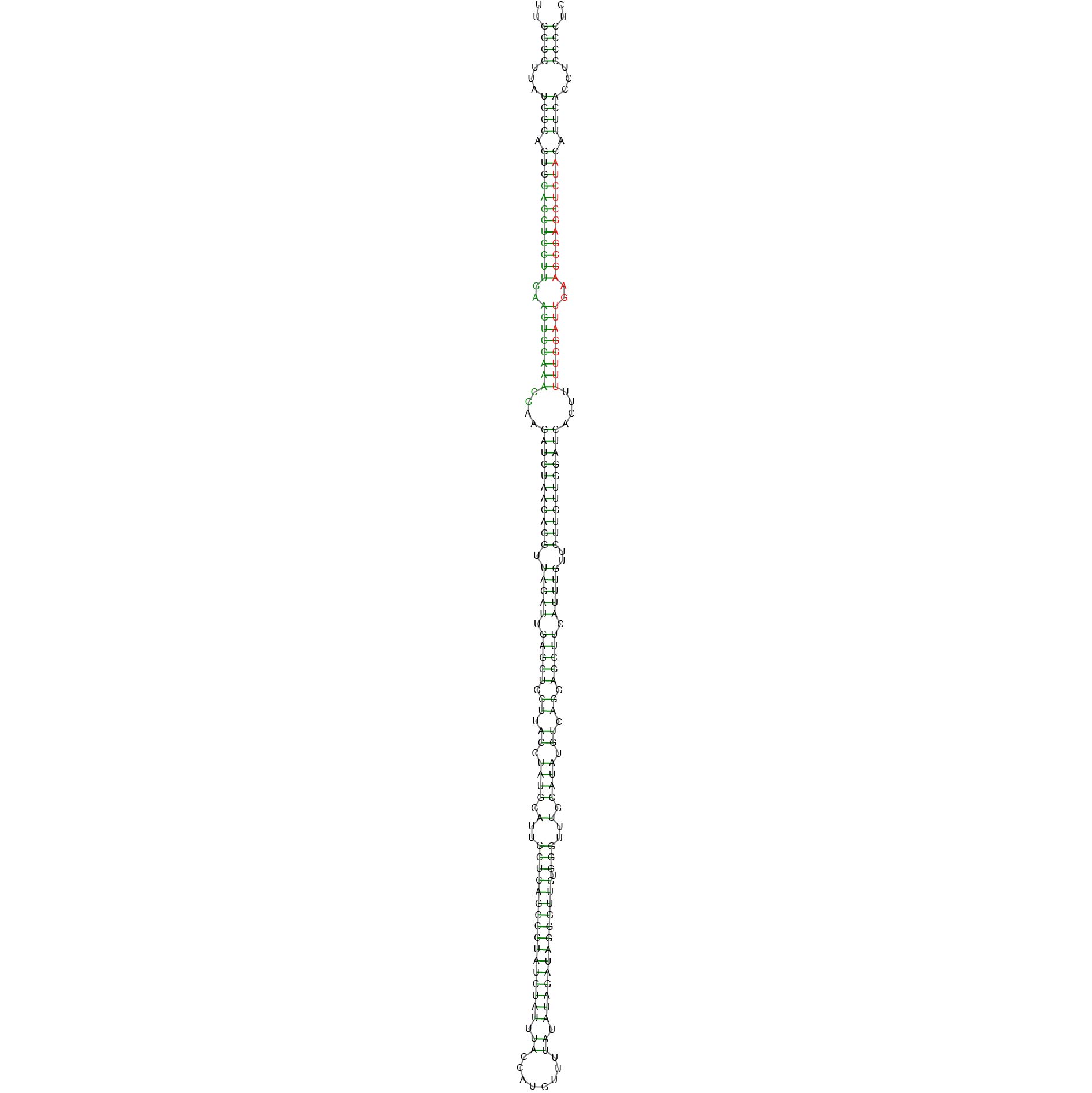

Supplement: Dataset S1 — Full list of hairpin structures in conserved miRNAs. (ZIP) [file pone.0064238.s001.zip › can-miR159a.jpg]

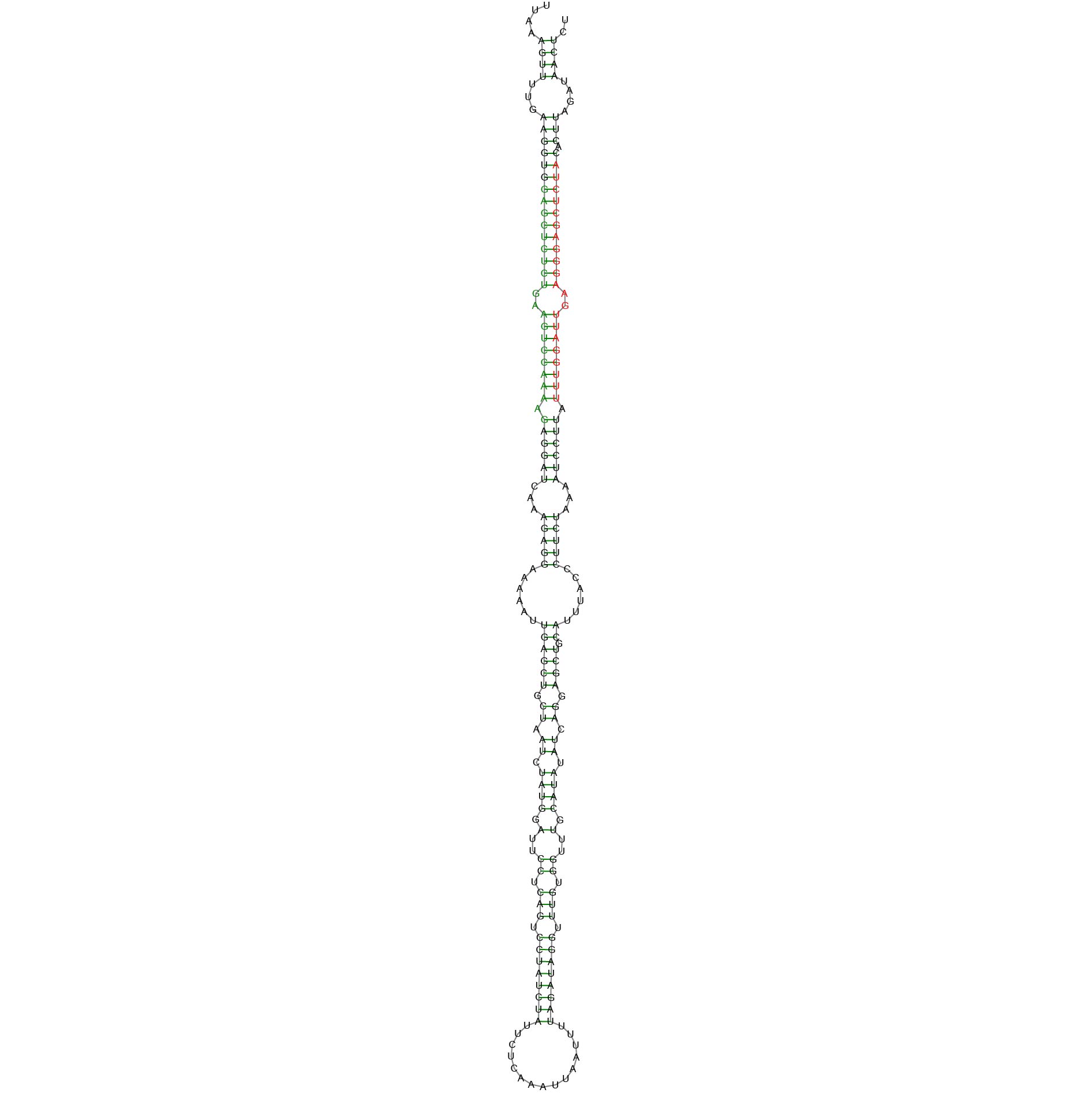

Supplement: Dataset S1 — Full list of hairpin structures in conserved miRNAs. (ZIP) [file pone.0064238.s001.zip › can-miR159b.jpg]

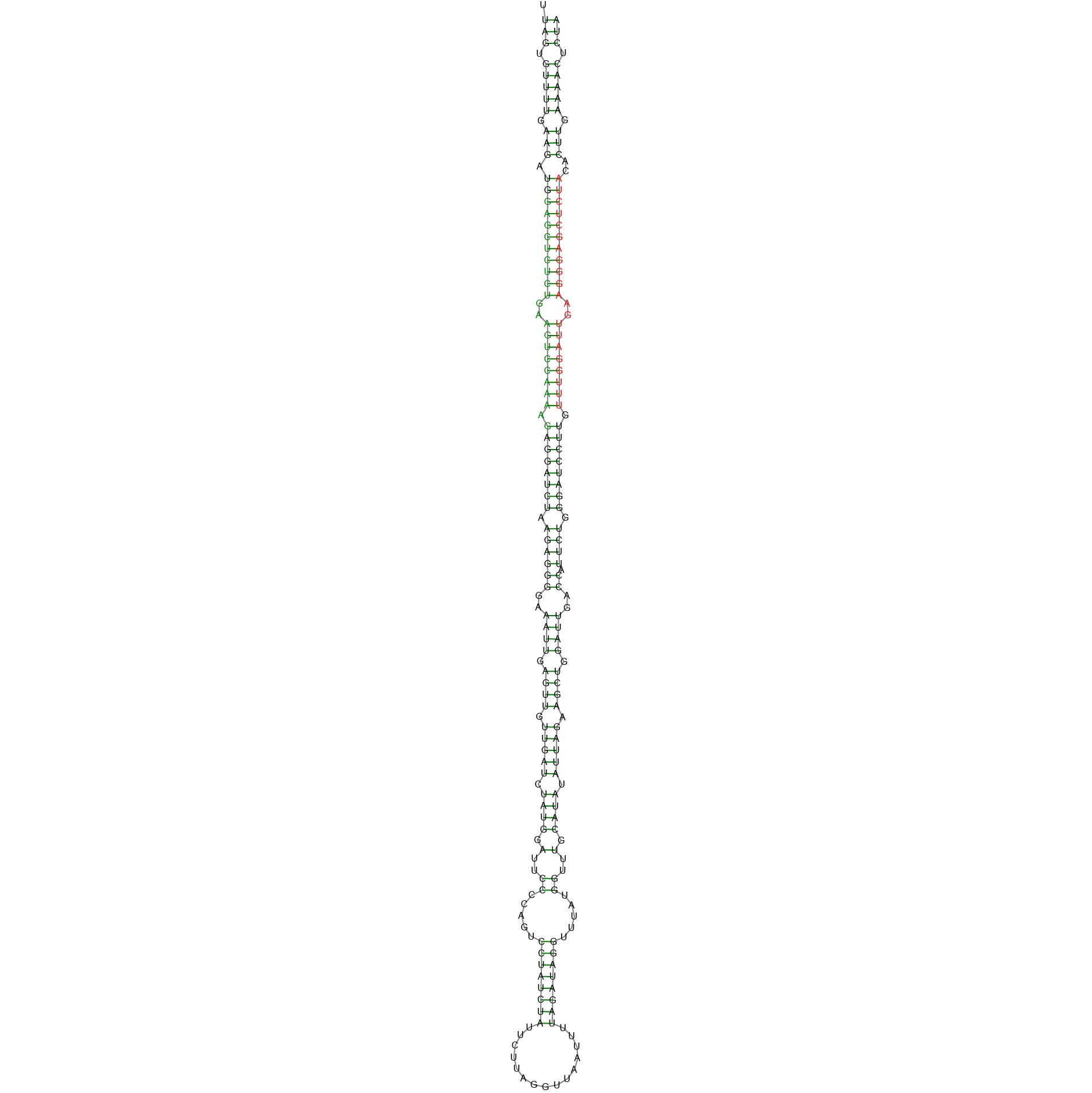

Supplement: Dataset S1 — Full list of hairpin structures in conserved miRNAs. (ZIP) [file pone.0064238.s001.zip › can-miR159c.jpg]

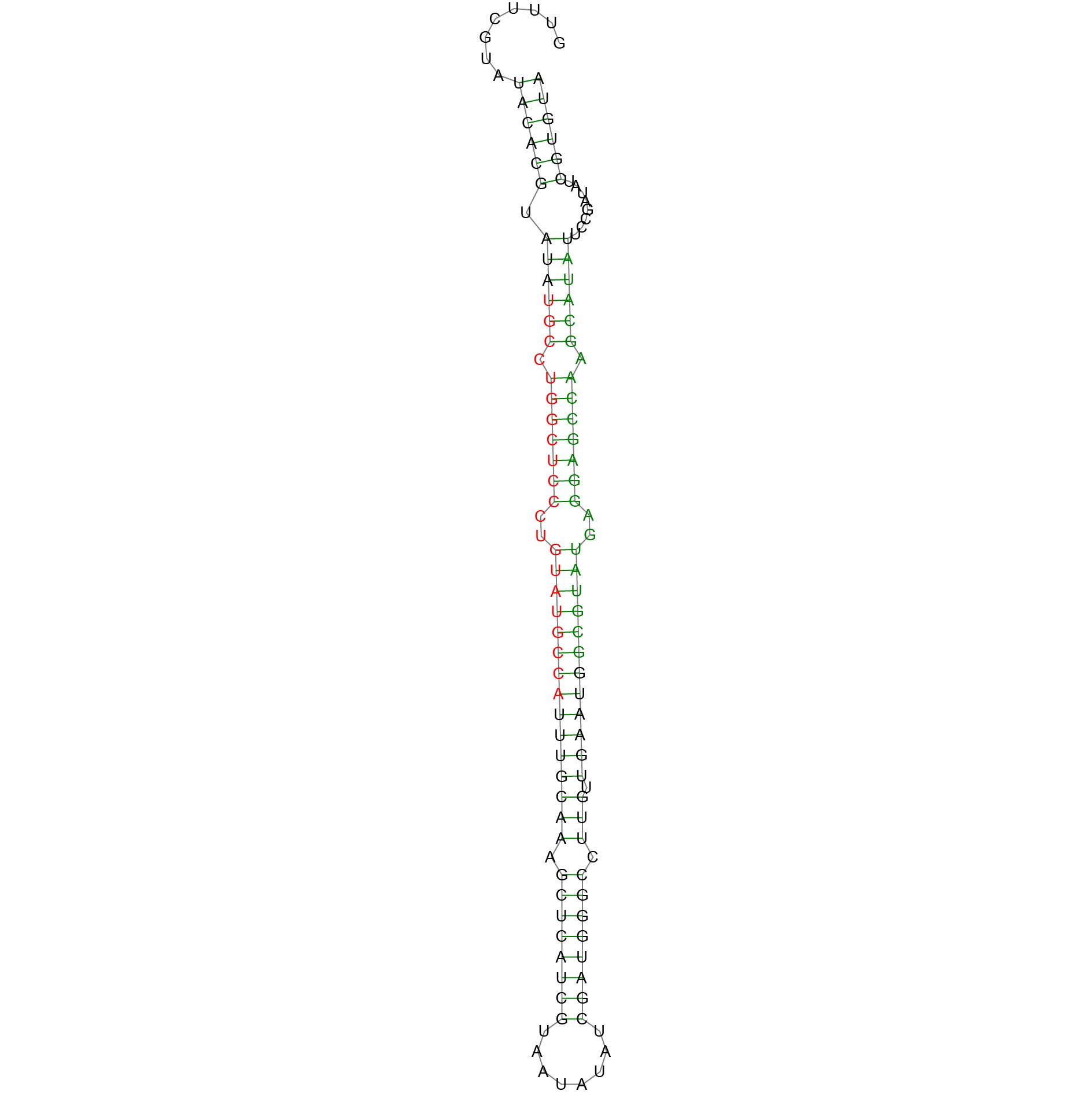

Supplement: Dataset S1 — Full list of hairpin structures in conserved miRNAs. (ZIP) [file pone.0064238.s001.zip › can-miR160.jpg]

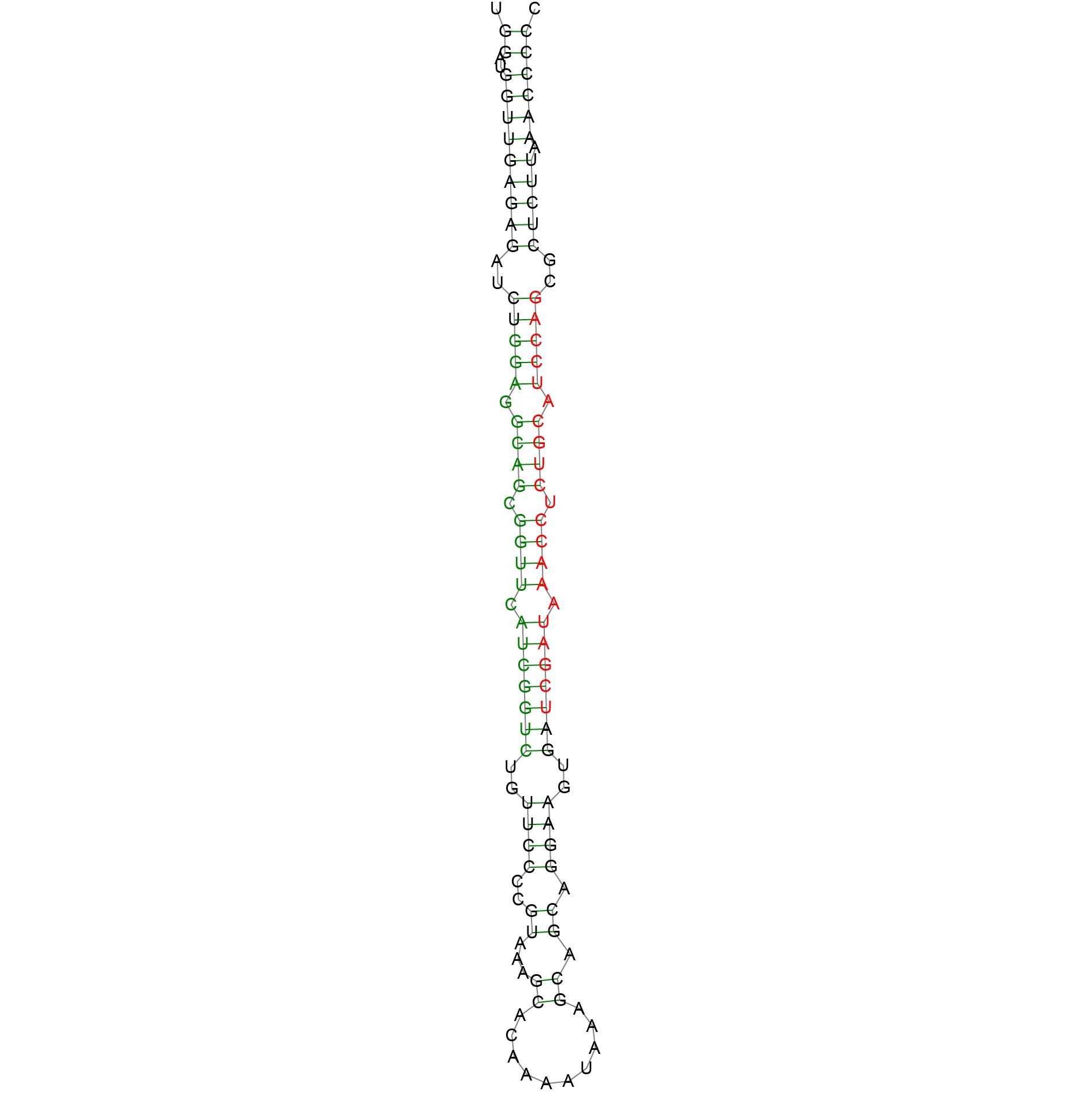

Supplement: Dataset S1 — Full list of hairpin structures in conserved miRNAs. (ZIP) [file pone.0064238.s001.zip › can-miR162a.jpg]

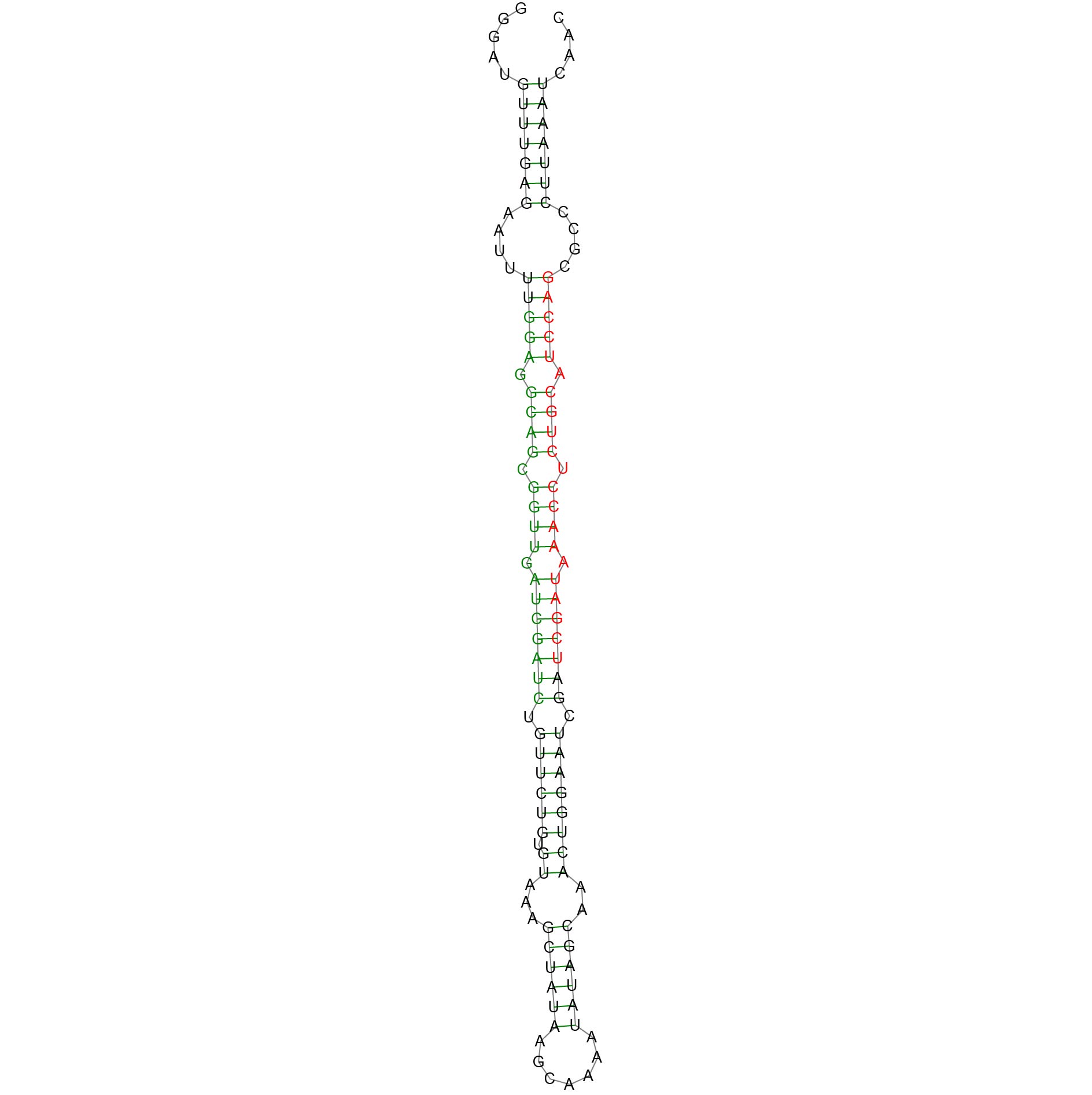

Supplement: Dataset S1 — Full list of hairpin structures in conserved miRNAs. (ZIP) [file pone.0064238.s001.zip › can-miR162b.jpg]

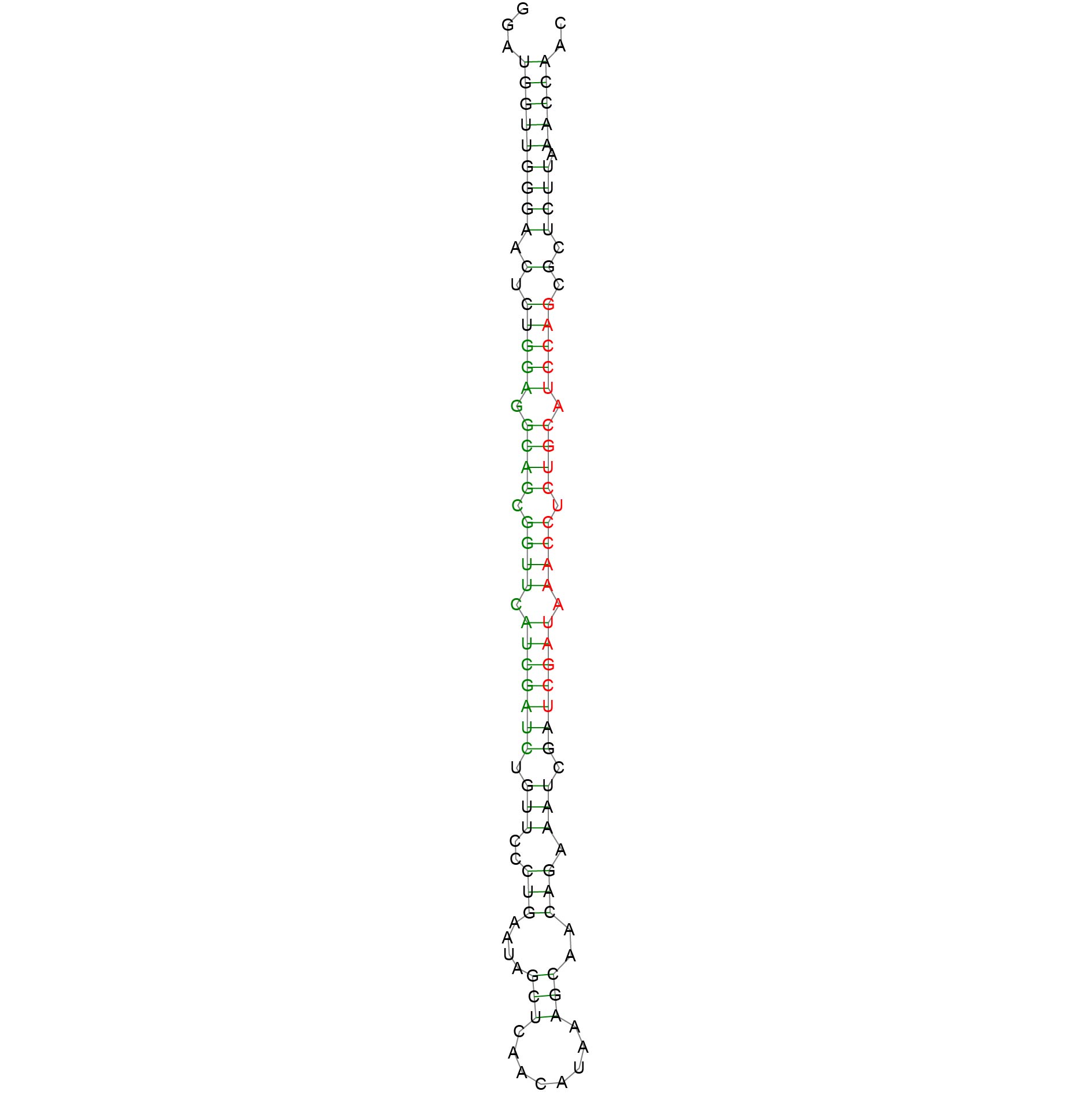

Supplement: Dataset S1 — Full list of hairpin structures in conserved miRNAs. (ZIP) [file pone.0064238.s001.zip › can-miR162c.jpg]

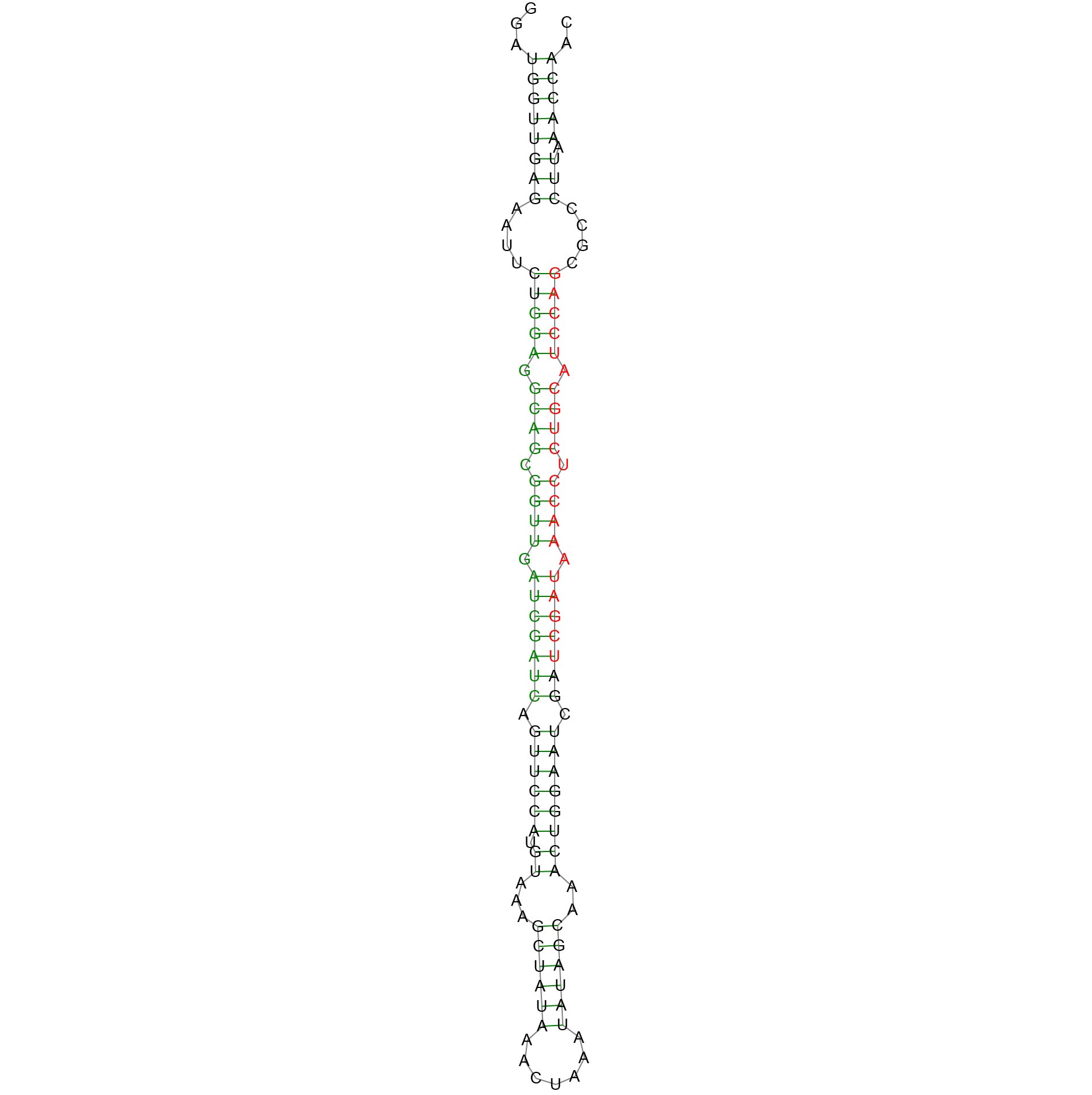

Supplement: Dataset S1 — Full list of hairpin structures in conserved miRNAs. (ZIP) [file pone.0064238.s001.zip › can-miR162d.jpg]

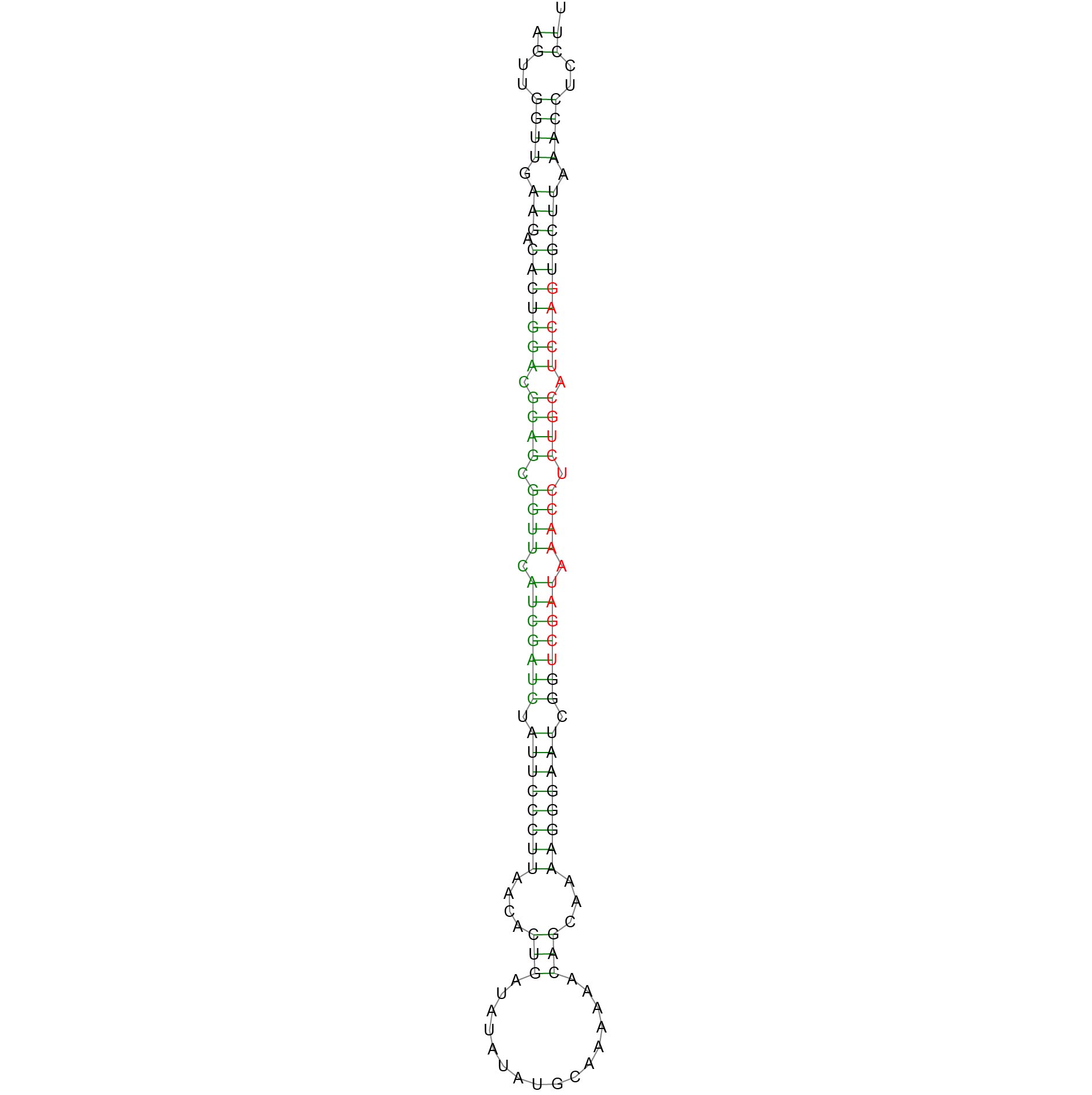

Supplement: Dataset S1 — Full list of hairpin structures in conserved miRNAs. (ZIP) [file pone.0064238.s001.zip › can-miR162e.jpg]

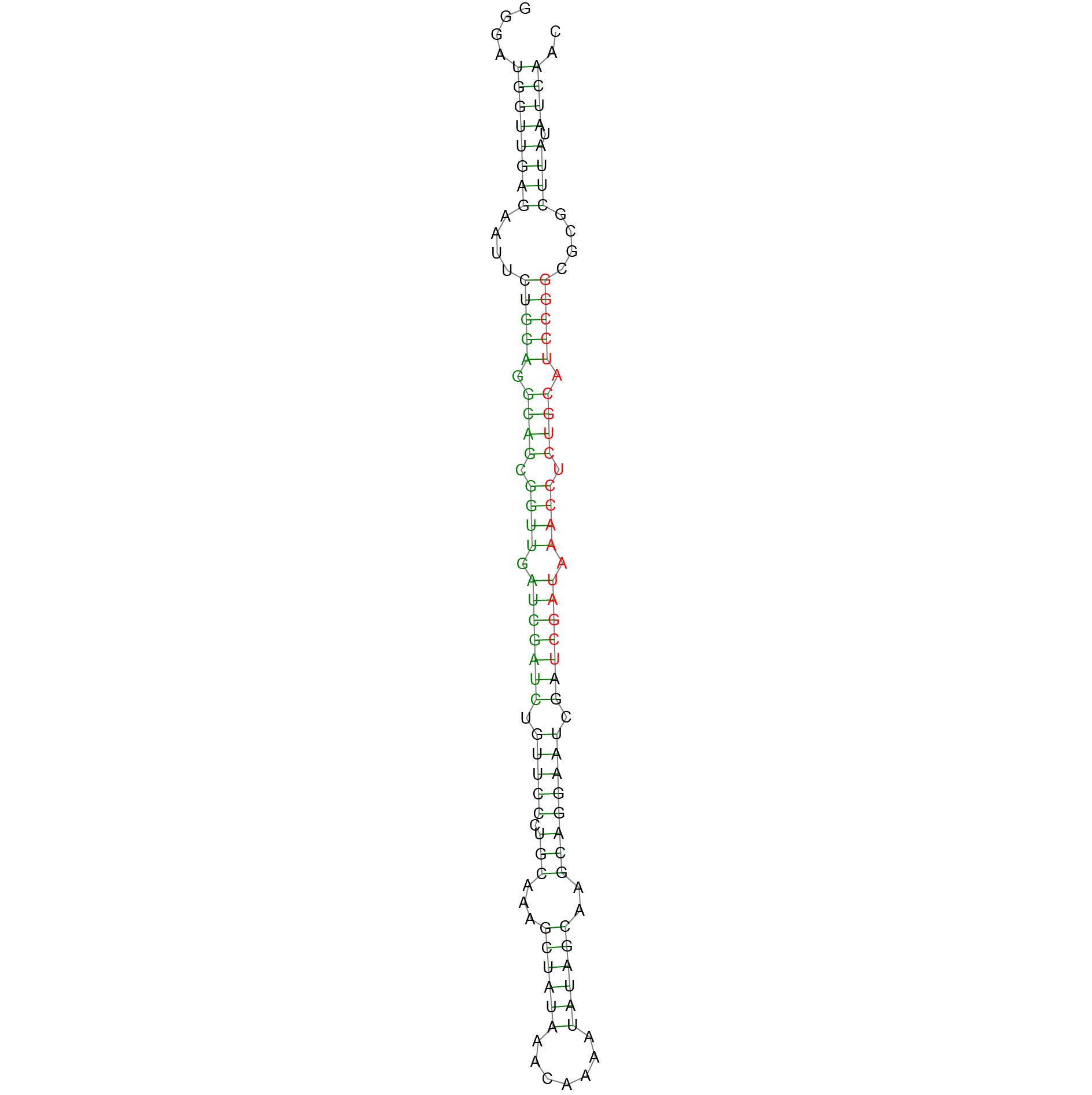

Supplement: Dataset S1 — Full list of hairpin structures in conserved miRNAs. (ZIP) [file pone.0064238.s001.zip › can-miR162f.jpg]

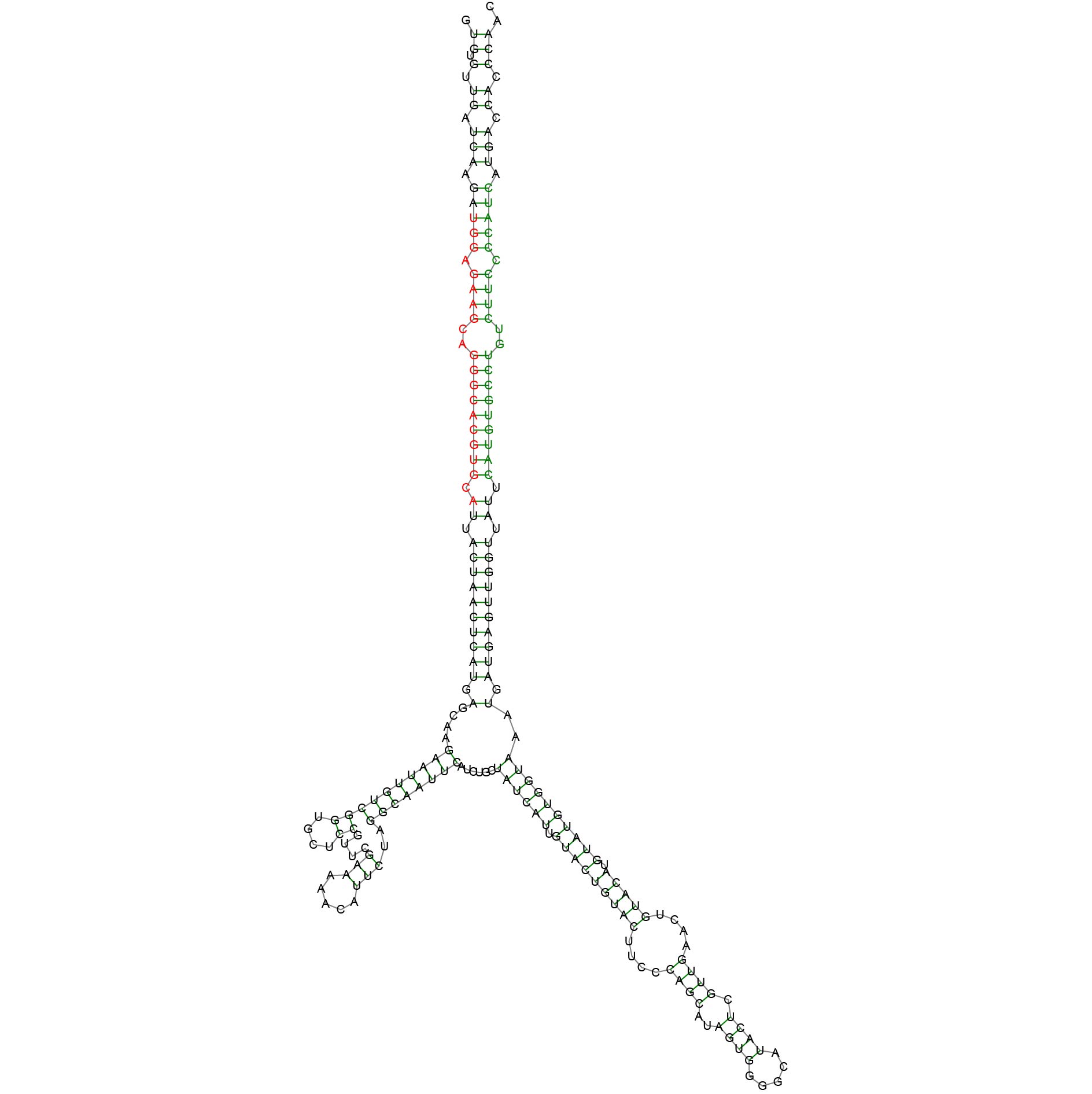

Supplement: Dataset S1 — Full list of hairpin structures in conserved miRNAs. (ZIP) [file pone.0064238.s001.zip › can-miR164a.jpg]

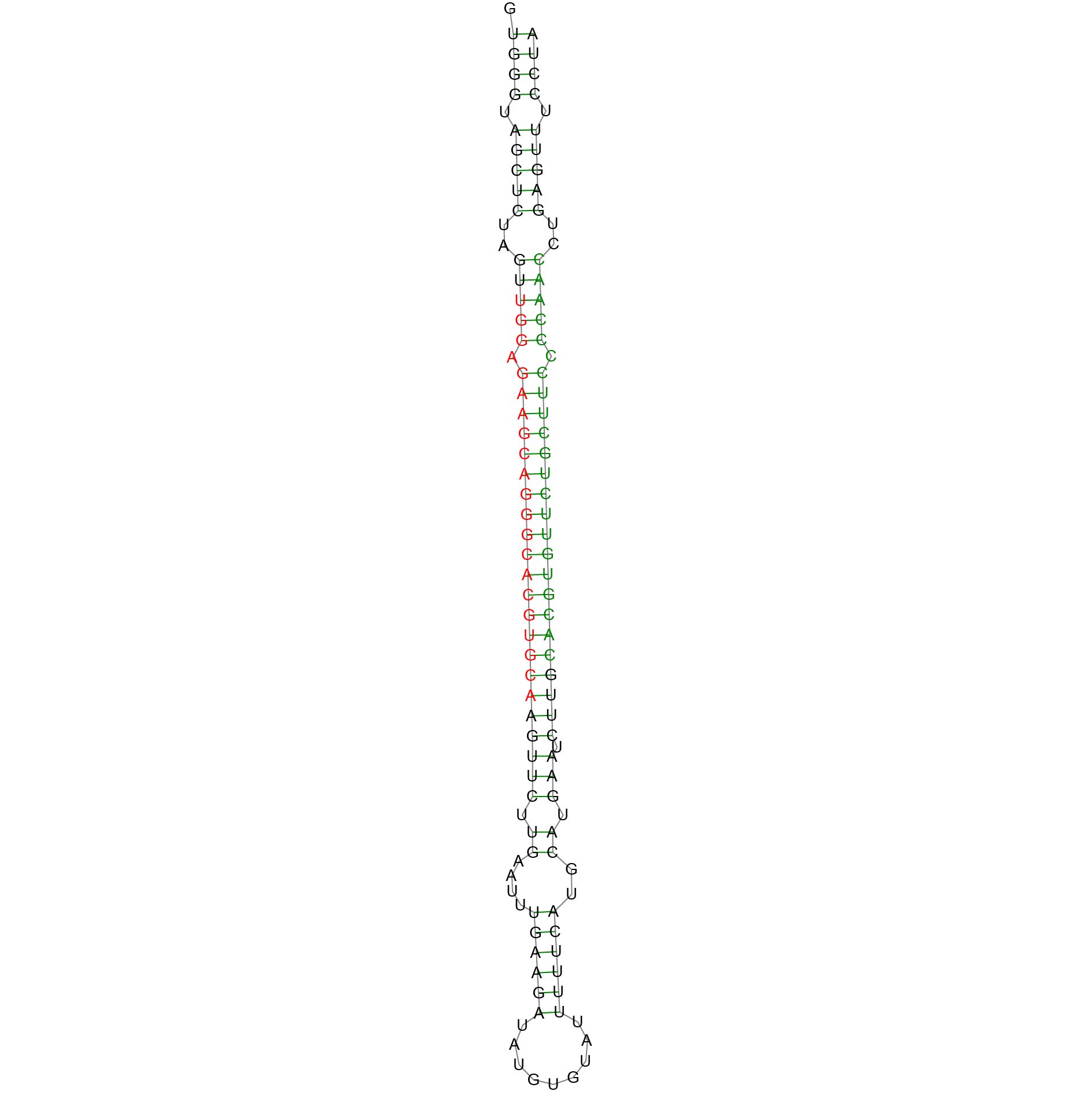

Supplement: Dataset S1 — Full list of hairpin structures in conserved miRNAs. (ZIP) [file pone.0064238.s001.zip › can-miR164b.jpg]

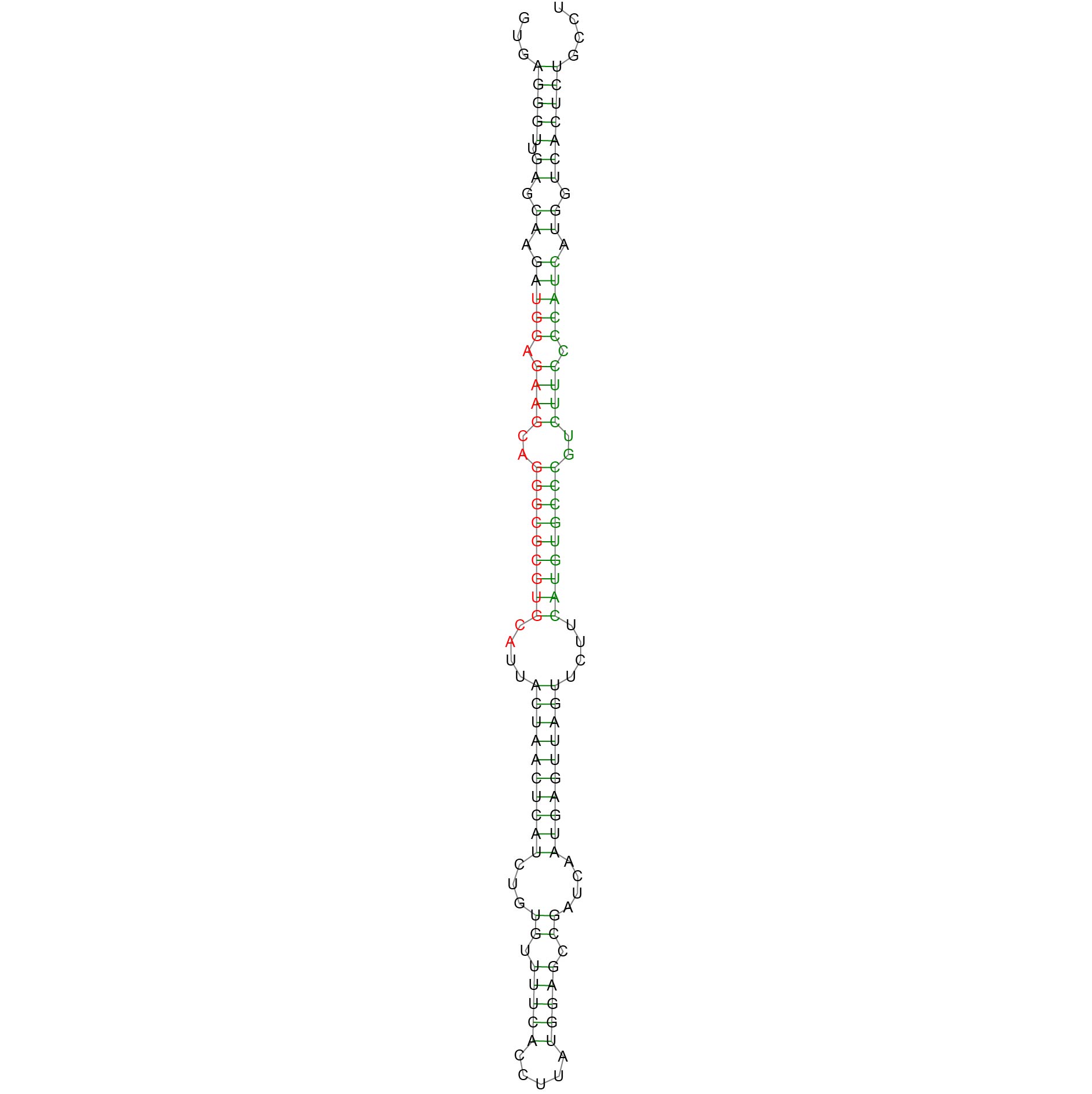

Supplement: Dataset S1 — Full list of hairpin structures in conserved miRNAs. (ZIP) [file pone.0064238.s001.zip › can-miR164c.jpg]

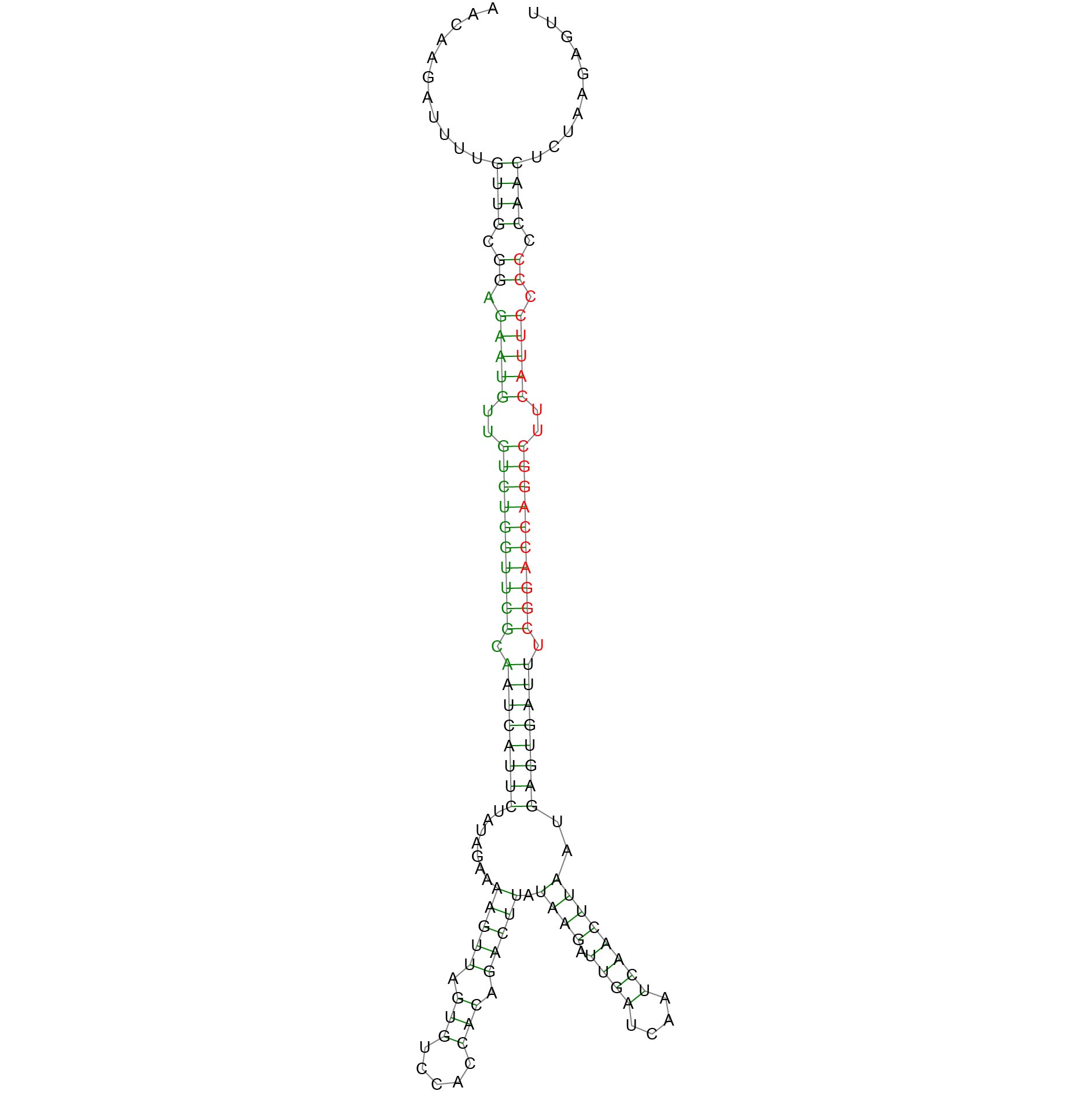

Supplement: Dataset S1 — Full list of hairpin structures in conserved miRNAs. (ZIP) [file pone.0064238.s001.zip › can-miR166a.jpg]

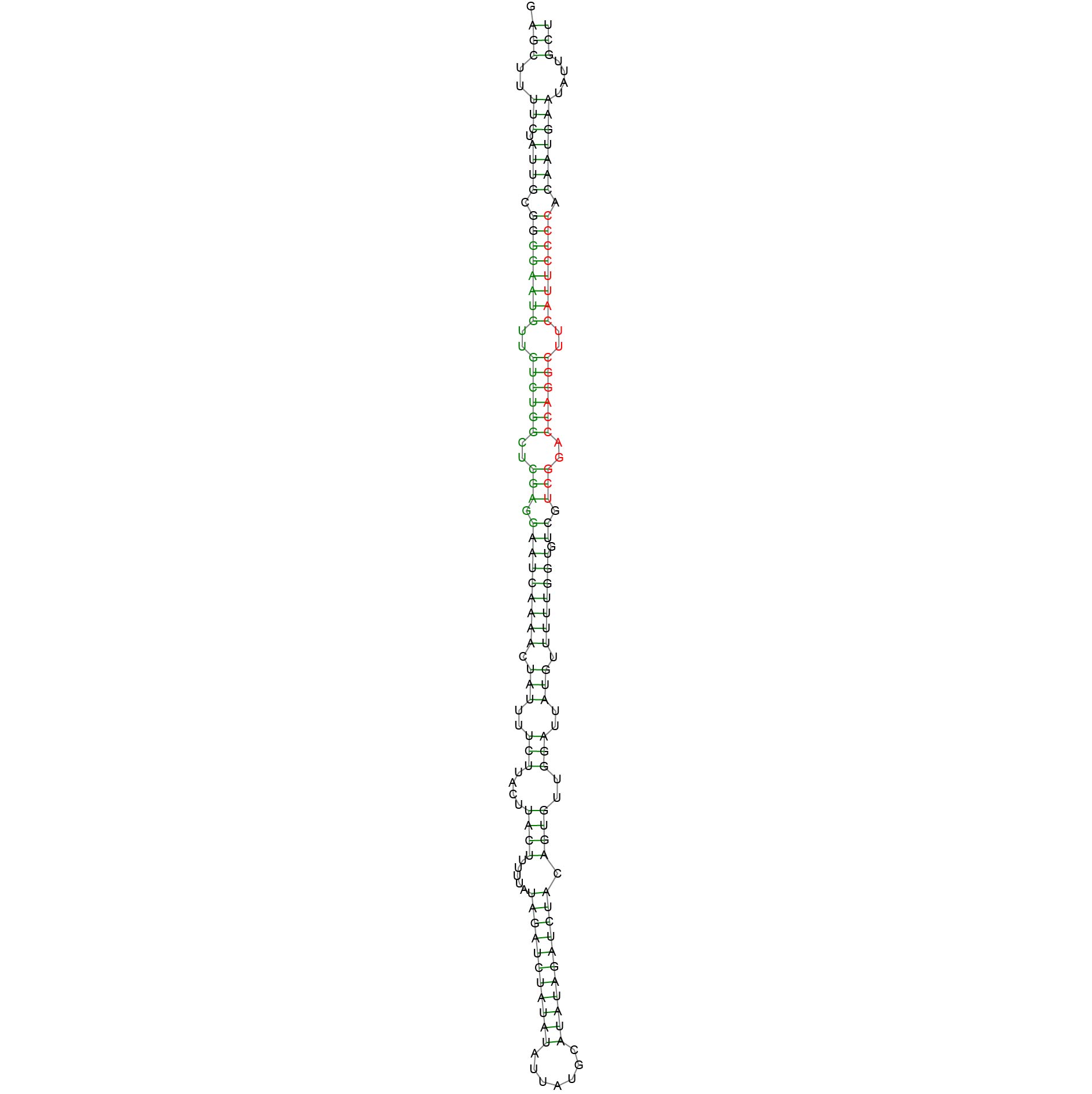

Supplement: Dataset S1 — Full list of hairpin structures in conserved miRNAs. (ZIP) [file pone.0064238.s001.zip › can-miR166b.jpg]

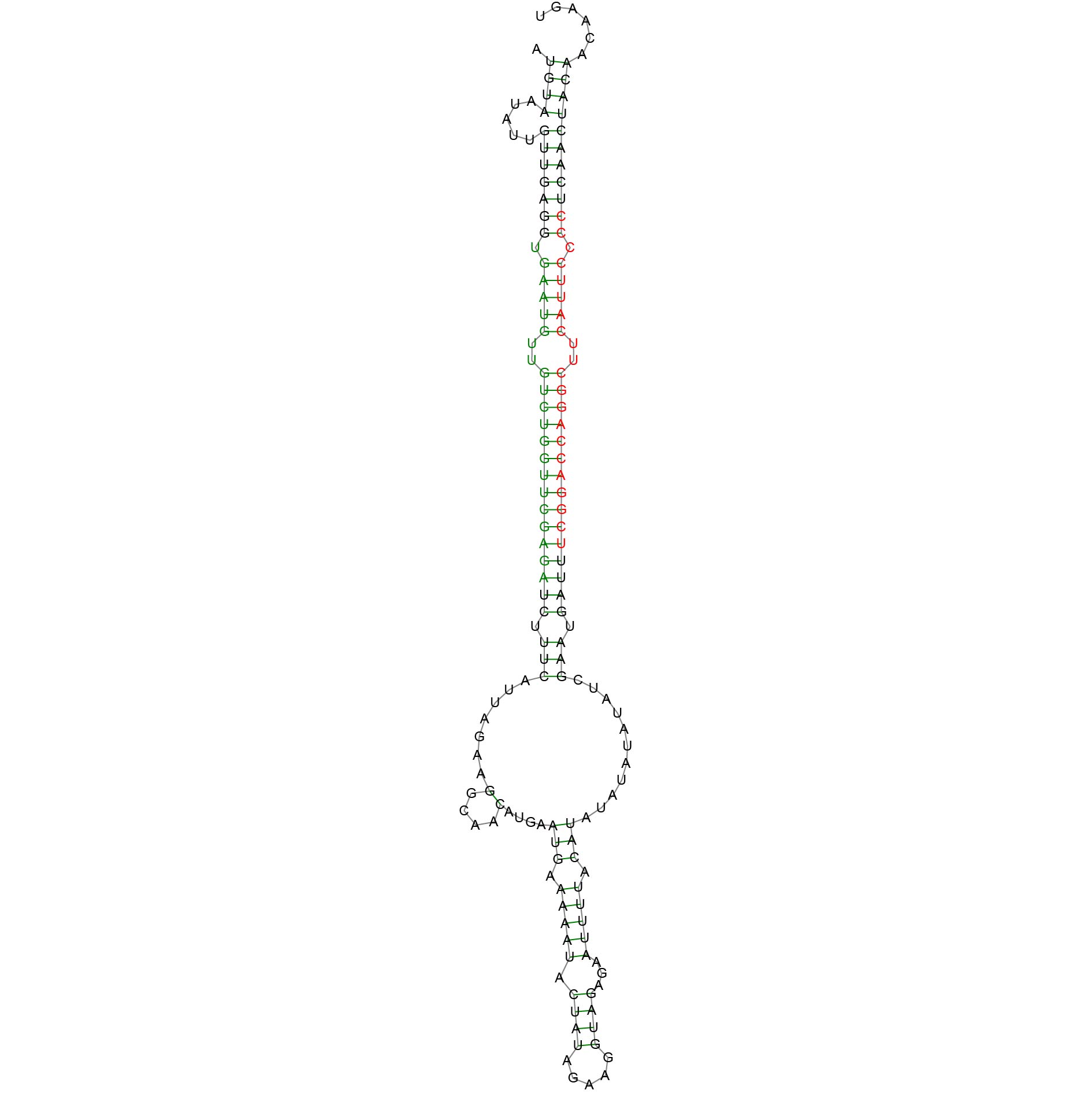

Supplement: Dataset S1 — Full list of hairpin structures in conserved miRNAs. (ZIP) [file pone.0064238.s001.zip › can-miR166c.jpg]

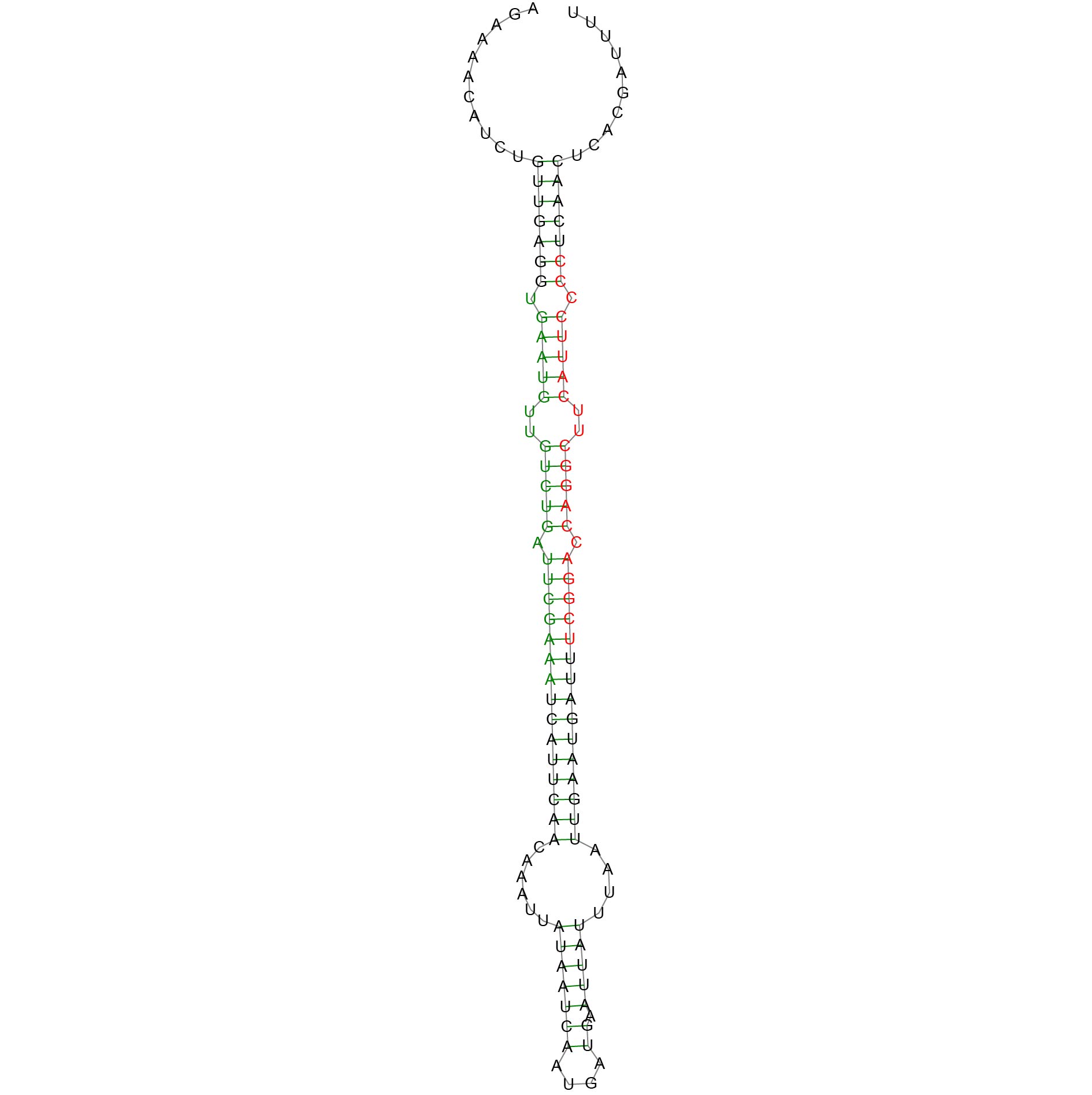

Supplement: Dataset S1 — Full list of hairpin structures in conserved miRNAs. (ZIP) [file pone.0064238.s001.zip › can-miR166d.jpg]

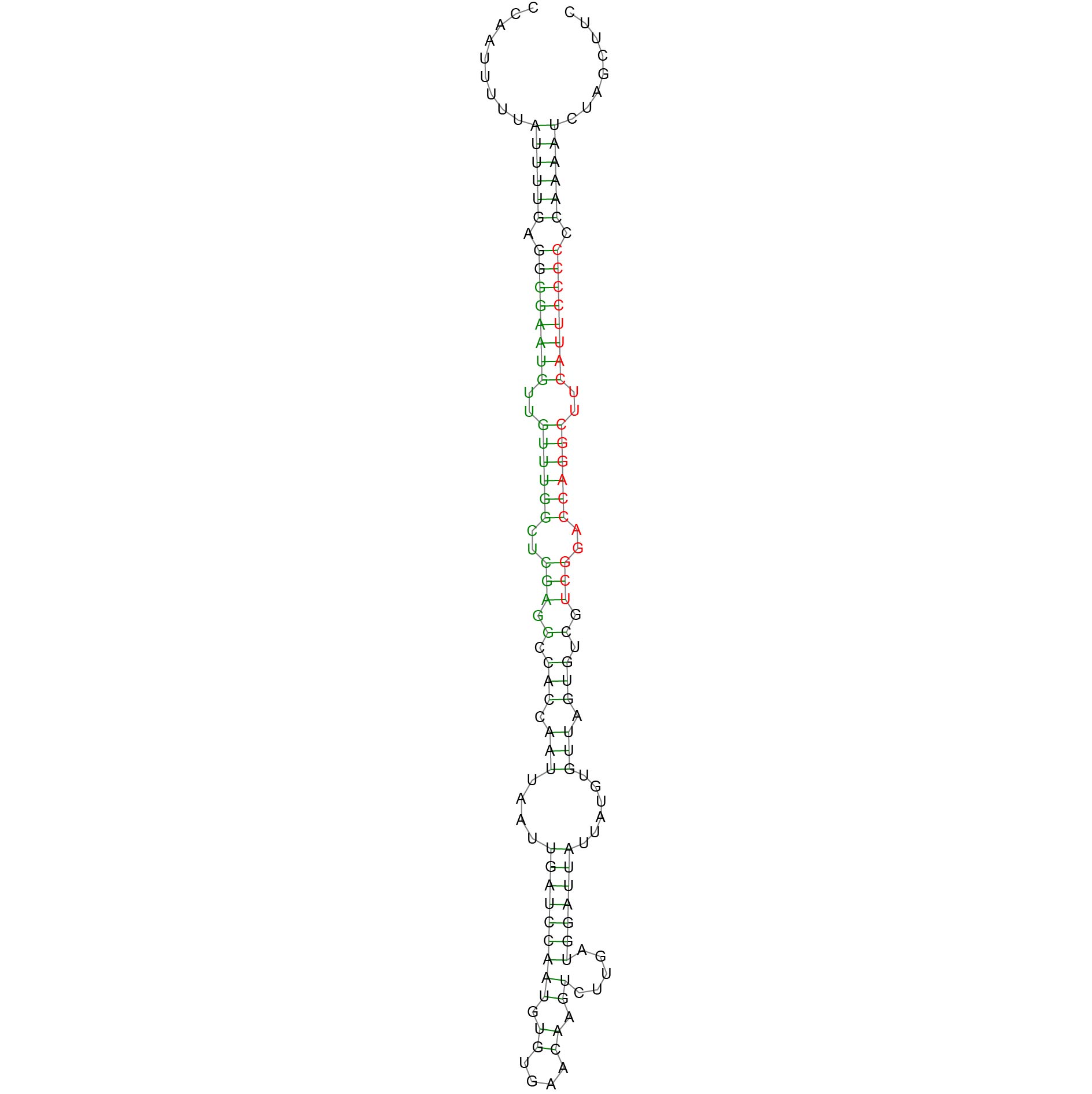

Supplement: Dataset S1 — Full list of hairpin structures in conserved miRNAs. (ZIP) [file pone.0064238.s001.zip › can-miR166e.jpg]

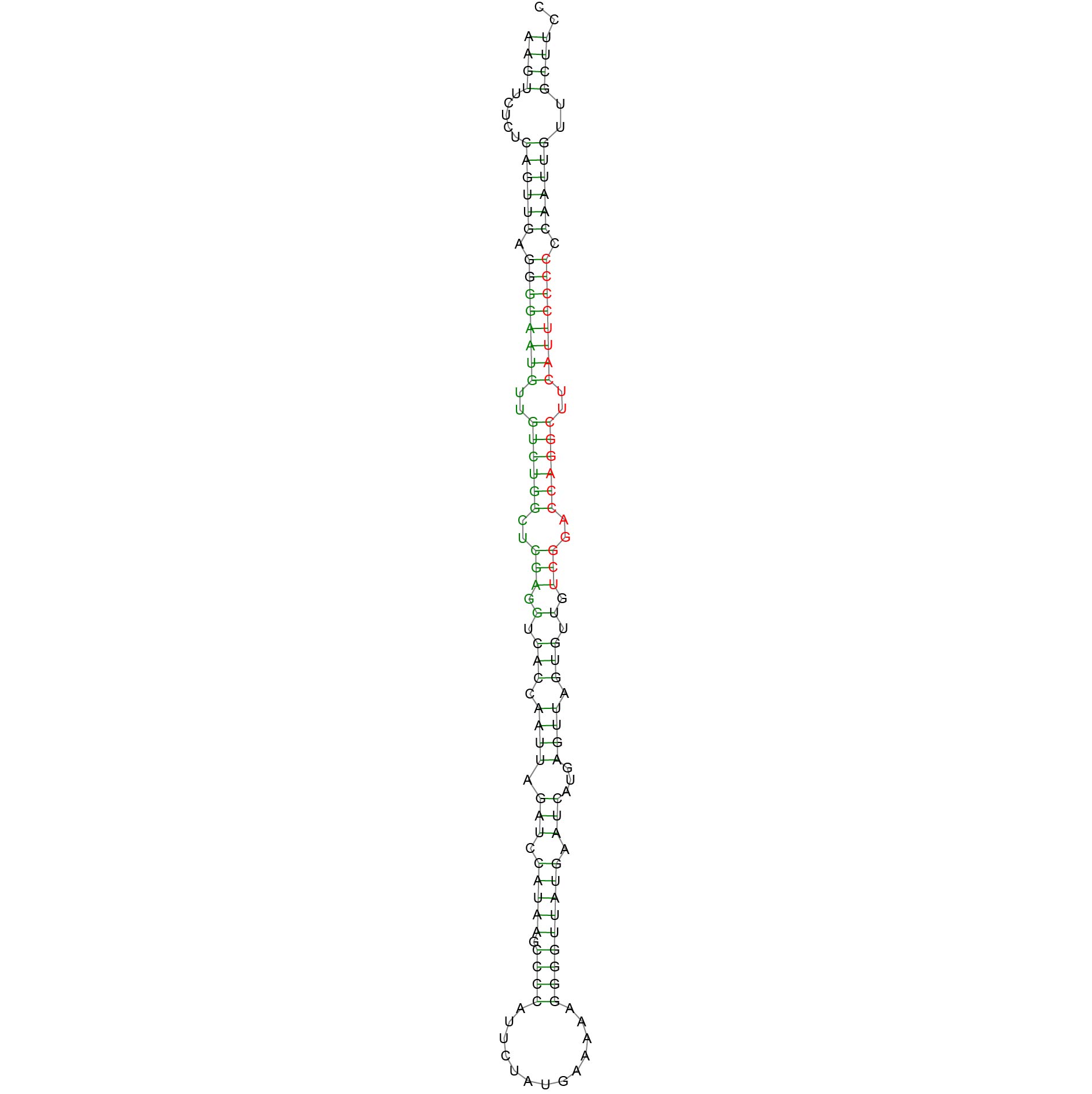

Supplement: Dataset S1 — Full list of hairpin structures in conserved miRNAs. (ZIP) [file pone.0064238.s001.zip › can-miR166f.jpg]

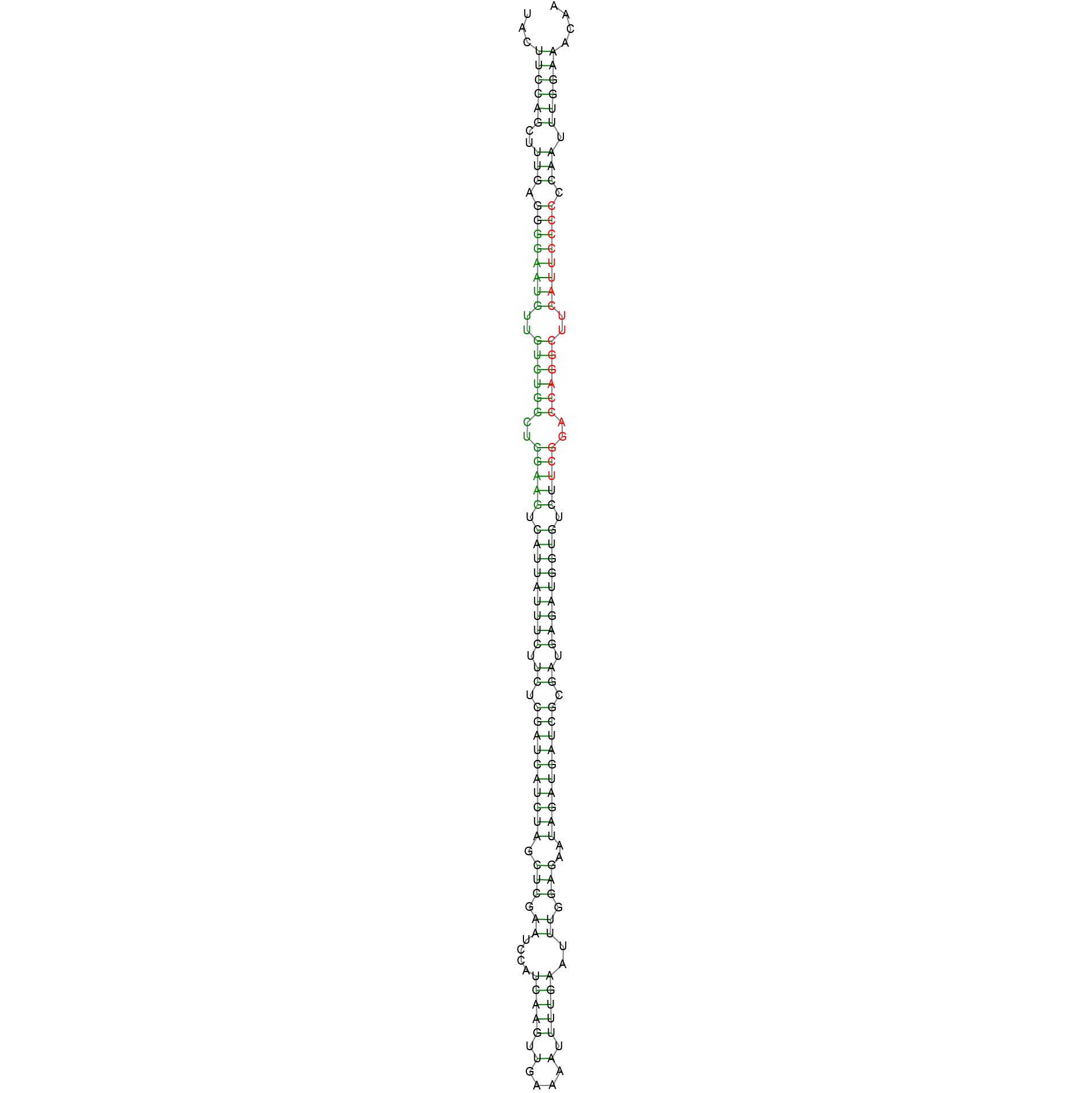

Supplement: Dataset S1 — Full list of hairpin structures in conserved miRNAs. (ZIP) [file pone.0064238.s001.zip › can-miR166g.jpg]

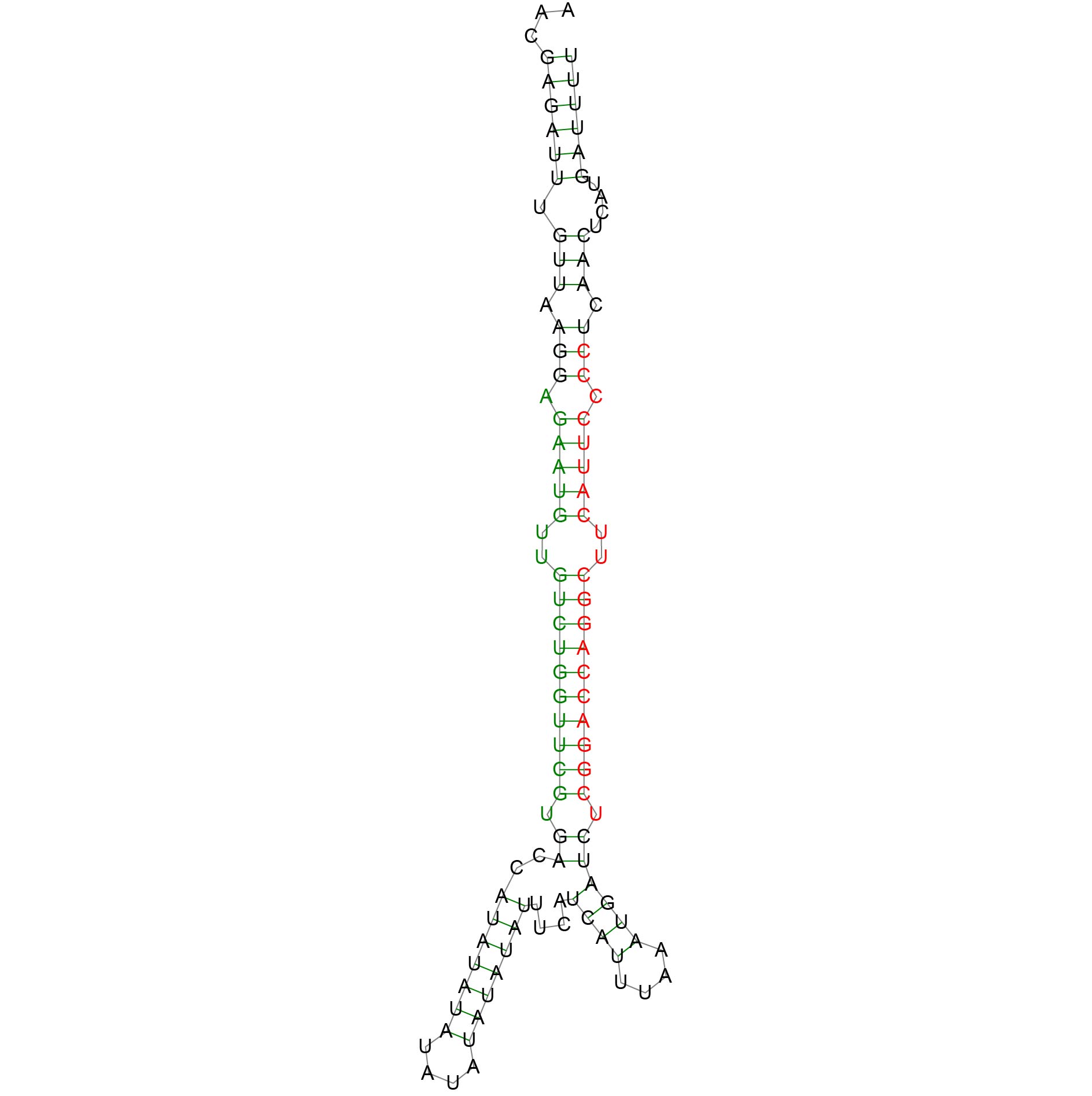

Supplement: Dataset S1 — Full list of hairpin structures in conserved miRNAs. (ZIP) [file pone.0064238.s001.zip › can-miR166h.jpg]

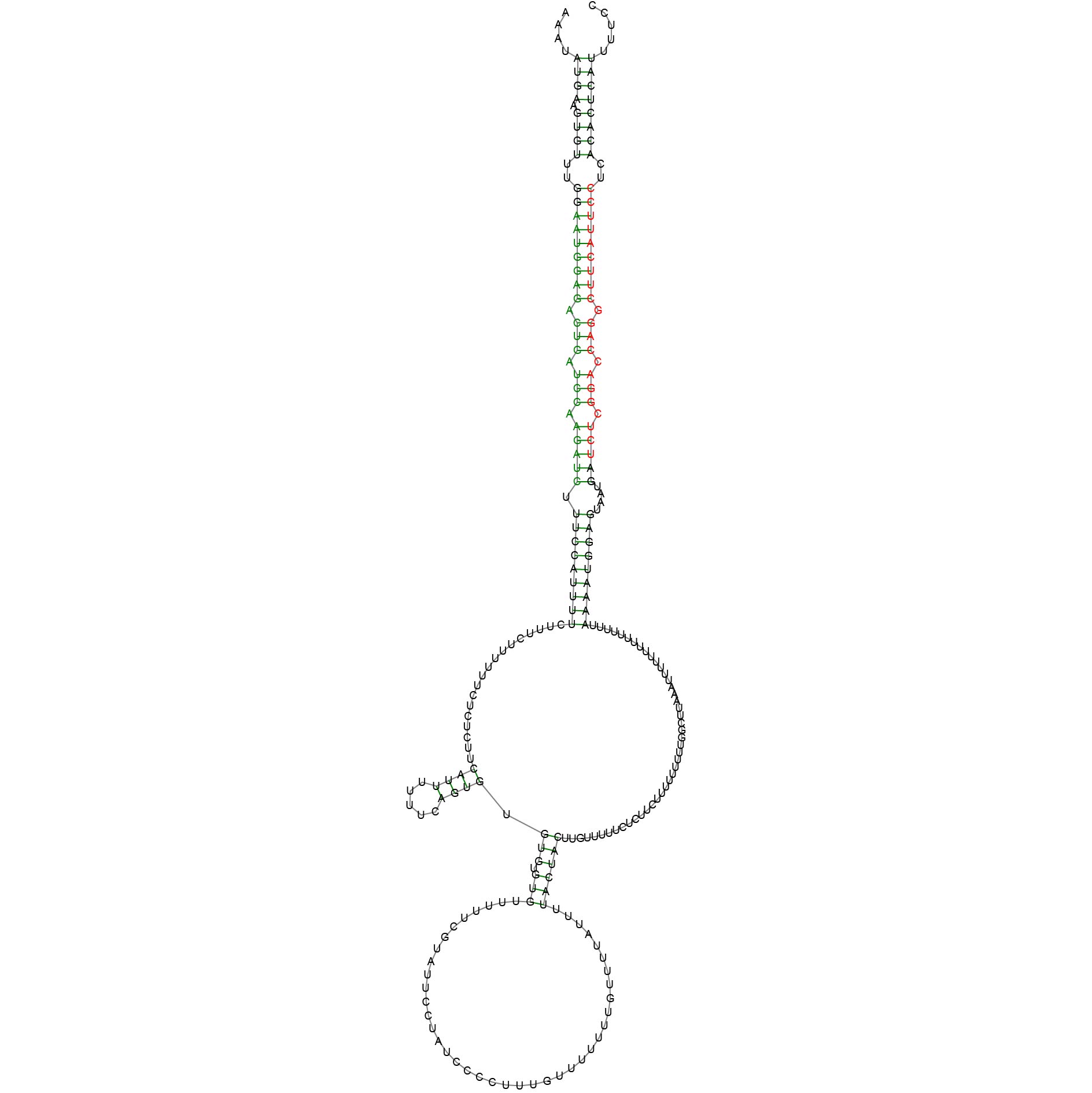

Supplement: Dataset S1 — Full list of hairpin structures in conserved miRNAs. (ZIP) [file pone.0064238.s001.zip › can-miR166i.jpg]

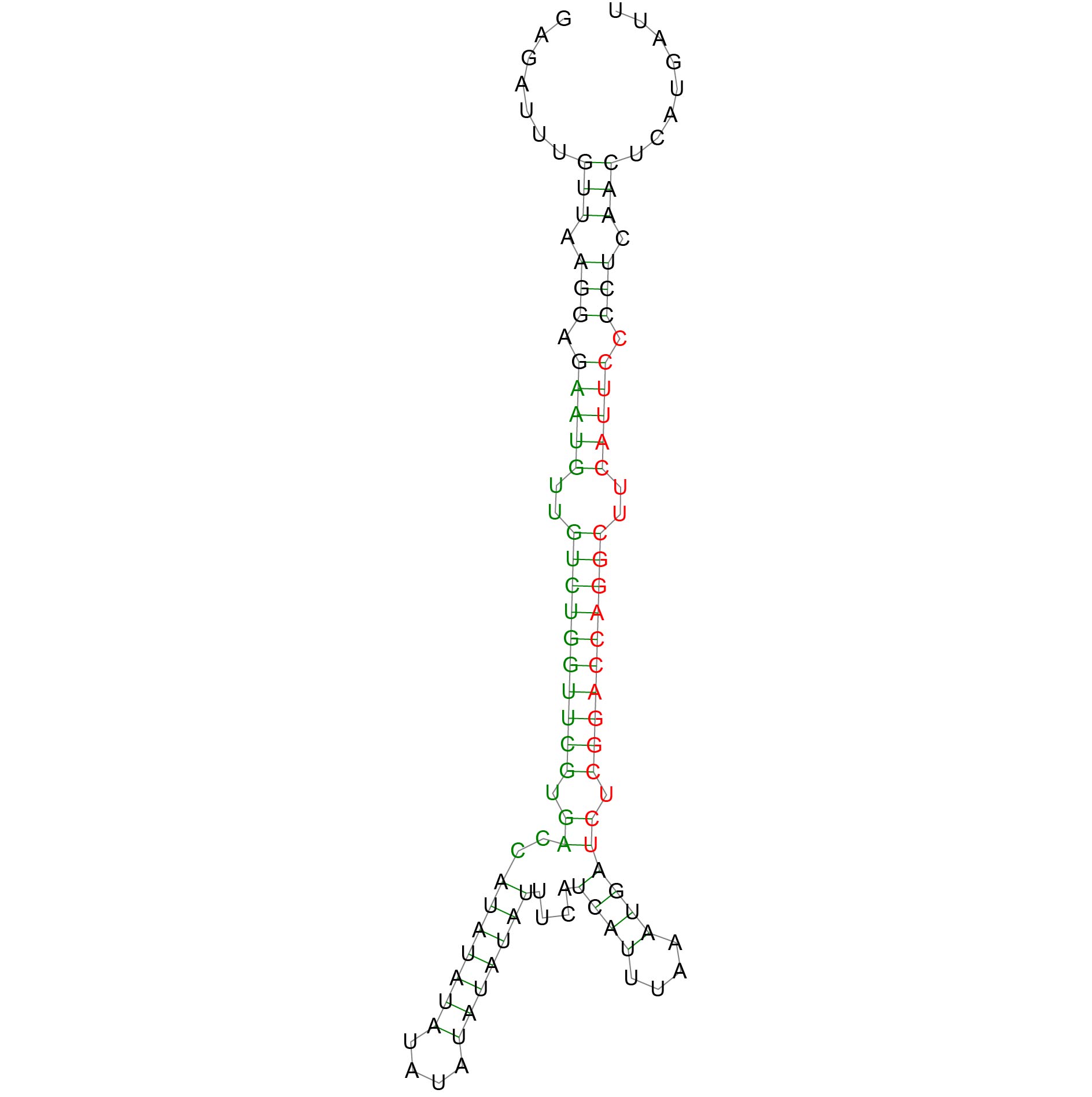

Supplement: Dataset S1 — Full list of hairpin structures in conserved miRNAs. (ZIP) [file pone.0064238.s001.zip › can-miR166j.jpg]

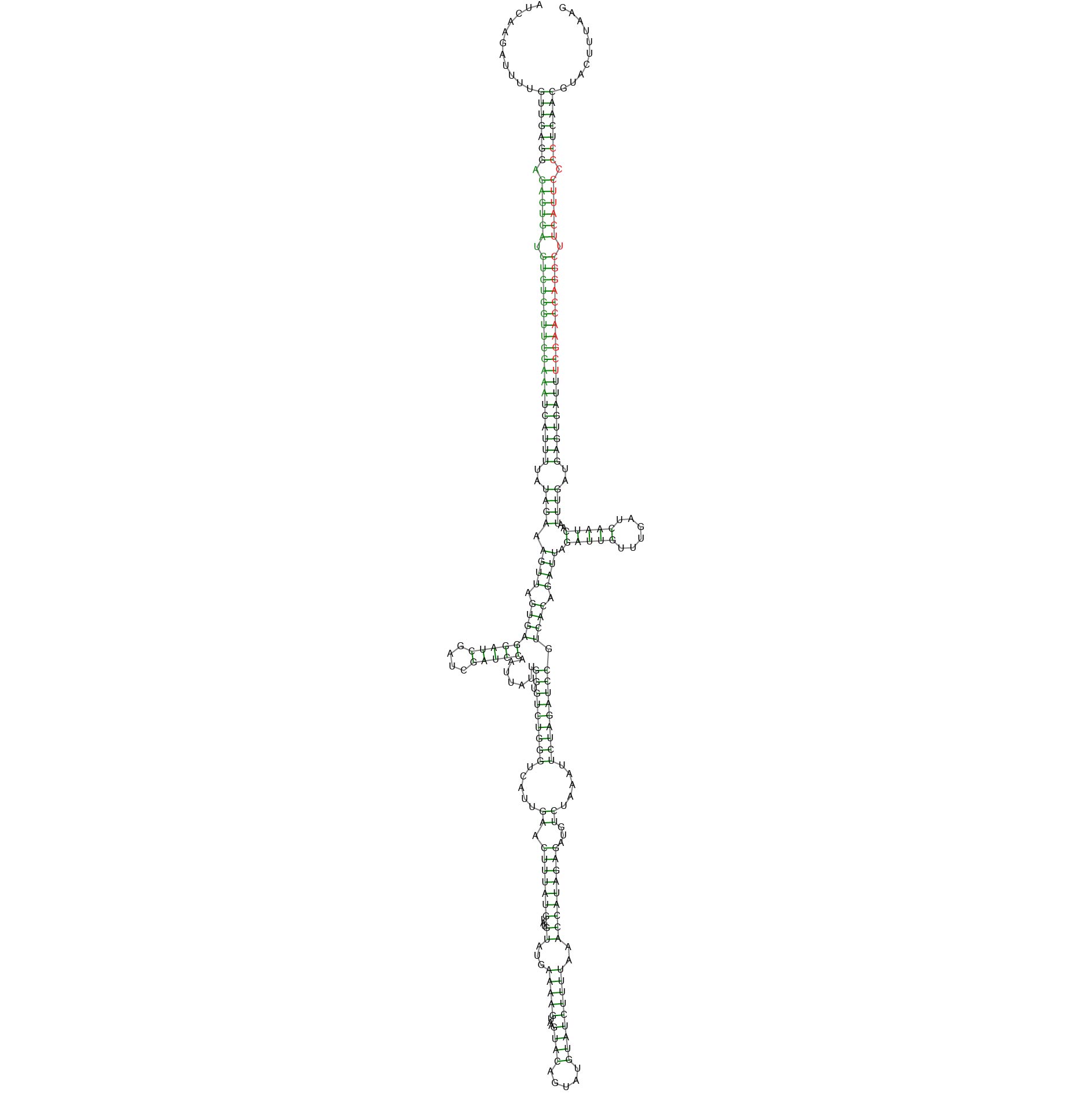

Supplement: Dataset S1 — Full list of hairpin structures in conserved miRNAs. (ZIP) [file pone.0064238.s001.zip › can-miR166k.jpg]

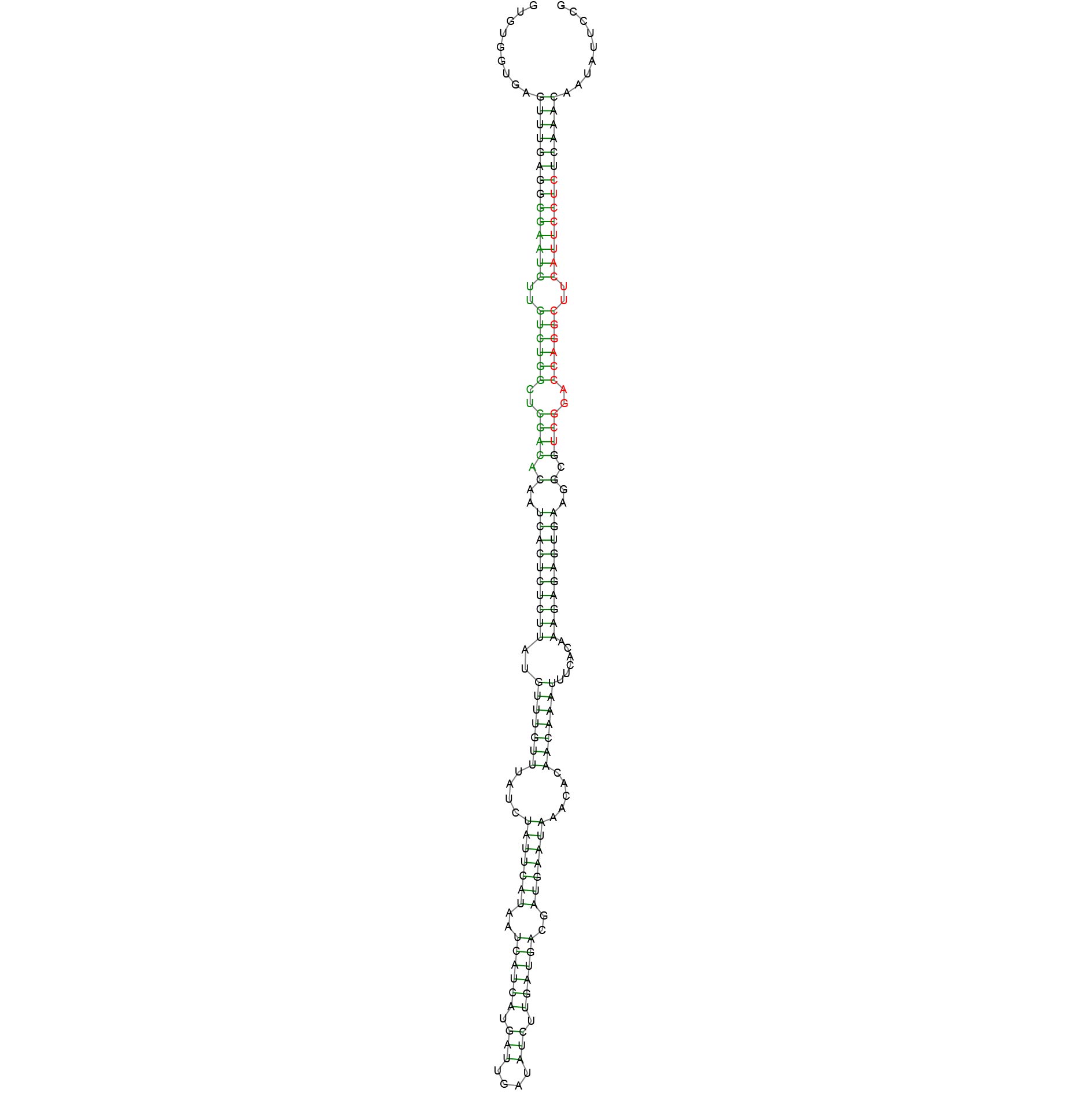

Supplement: Dataset S1 — Full list of hairpin structures in conserved miRNAs. (ZIP) [file pone.0064238.s001.zip › can-miR166l.jpg]

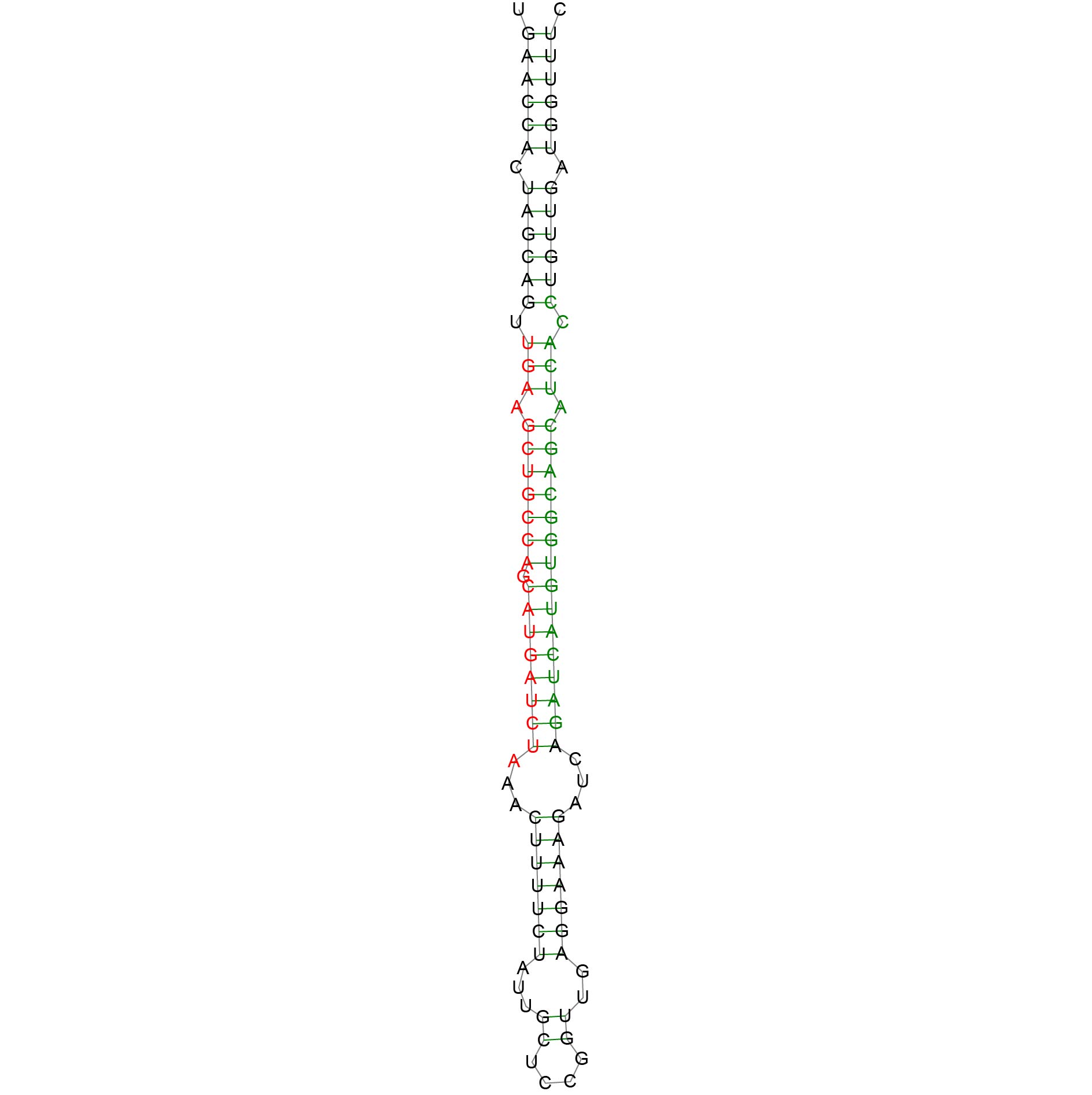

Supplement: Dataset S1 — Full list of hairpin structures in conserved miRNAs. (ZIP) [file pone.0064238.s001.zip › can-miR167a.jpg]

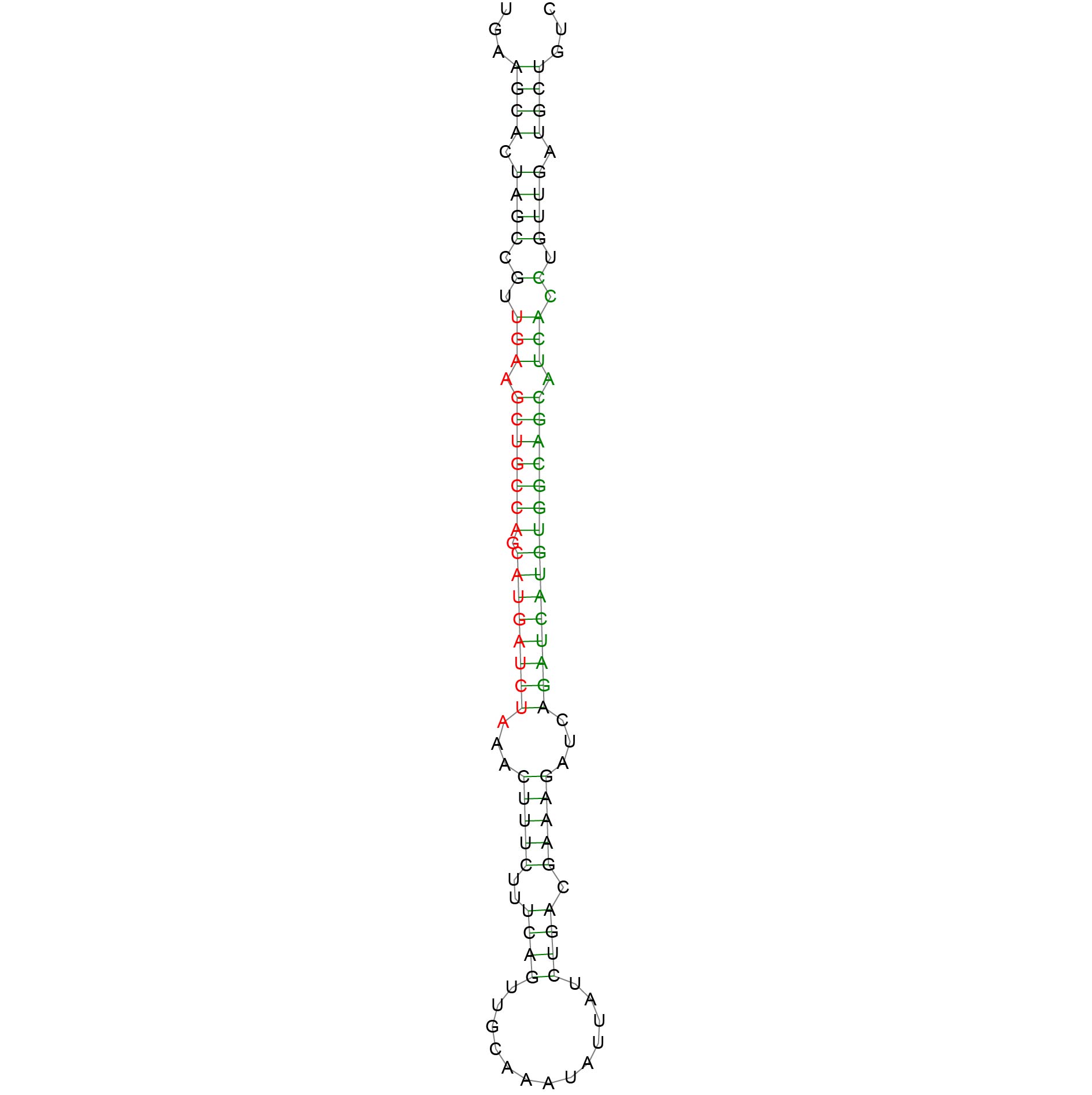

Supplement: Dataset S1 — Full list of hairpin structures in conserved miRNAs. (ZIP) [file pone.0064238.s001.zip › can-miR167b.jpg]

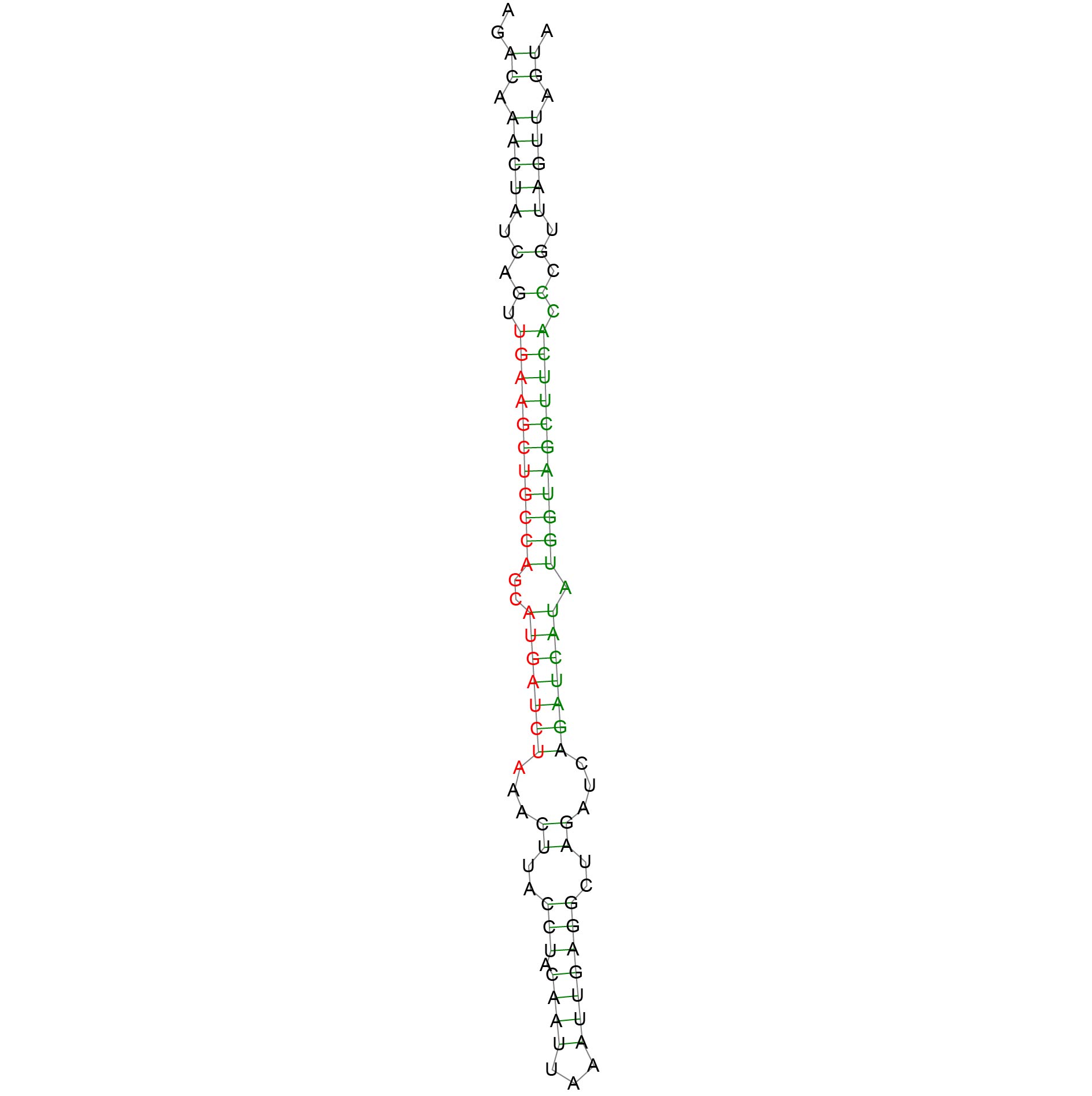

Supplement: Dataset S1 — Full list of hairpin structures in conserved miRNAs. (ZIP) [file pone.0064238.s001.zip › can-miR167c.jpg]

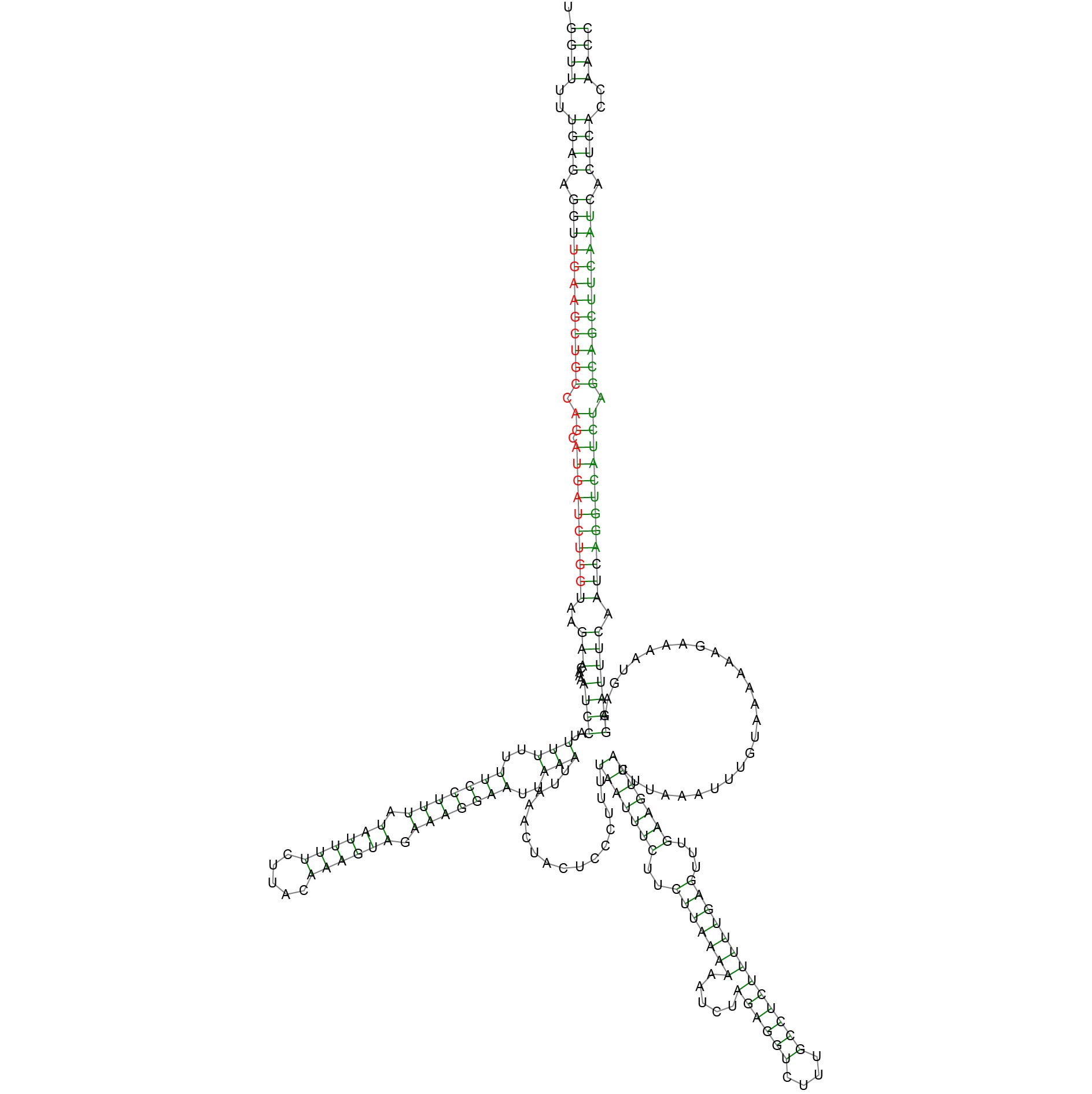

Supplement: Dataset S1 — Full list of hairpin structures in conserved miRNAs. (ZIP) [file pone.0064238.s001.zip › can-miR167d.jpg]

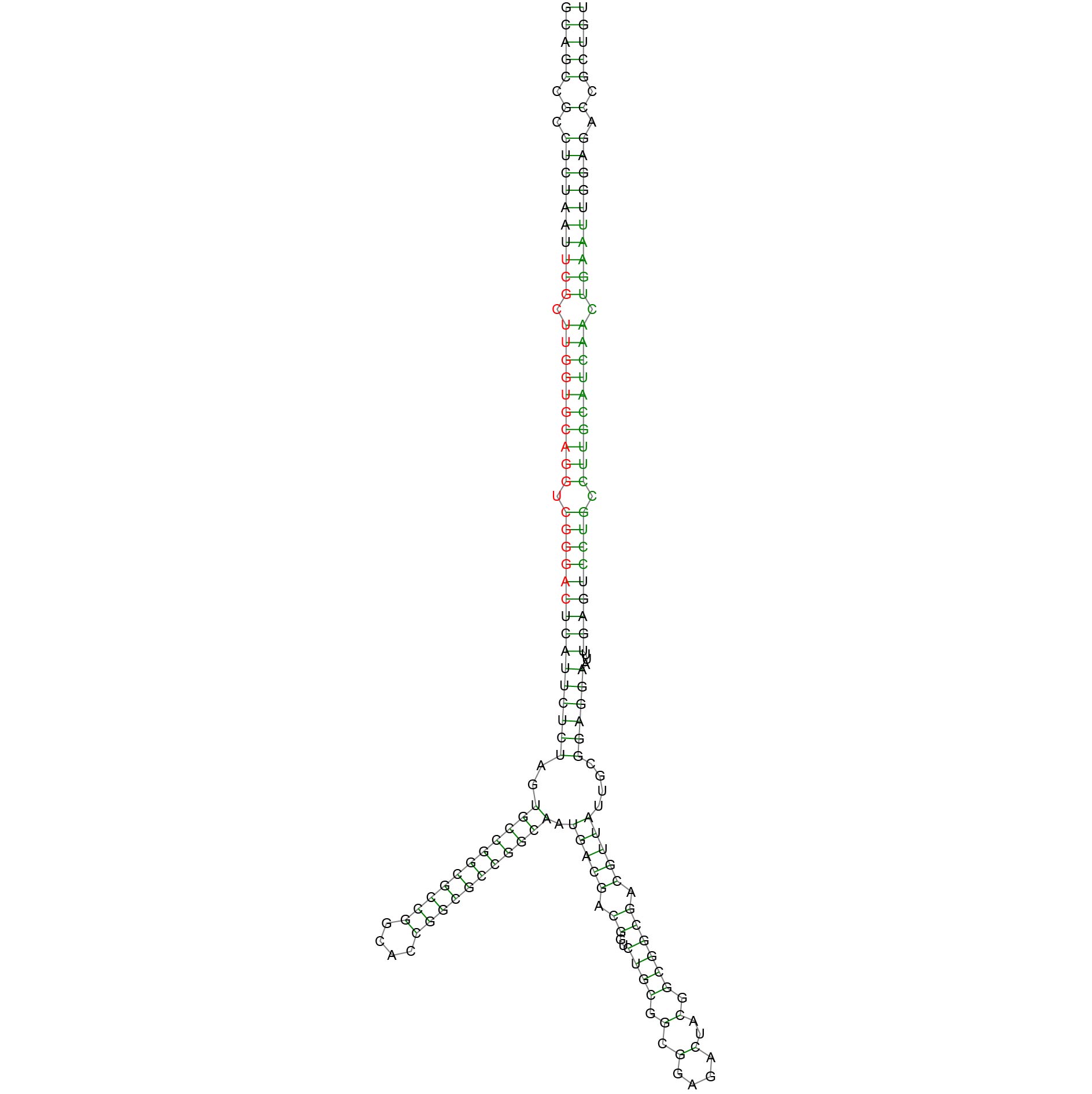

Supplement: Dataset S1 — Full list of hairpin structures in conserved miRNAs. (ZIP) [file pone.0064238.s001.zip › can-miR168a.jpg]

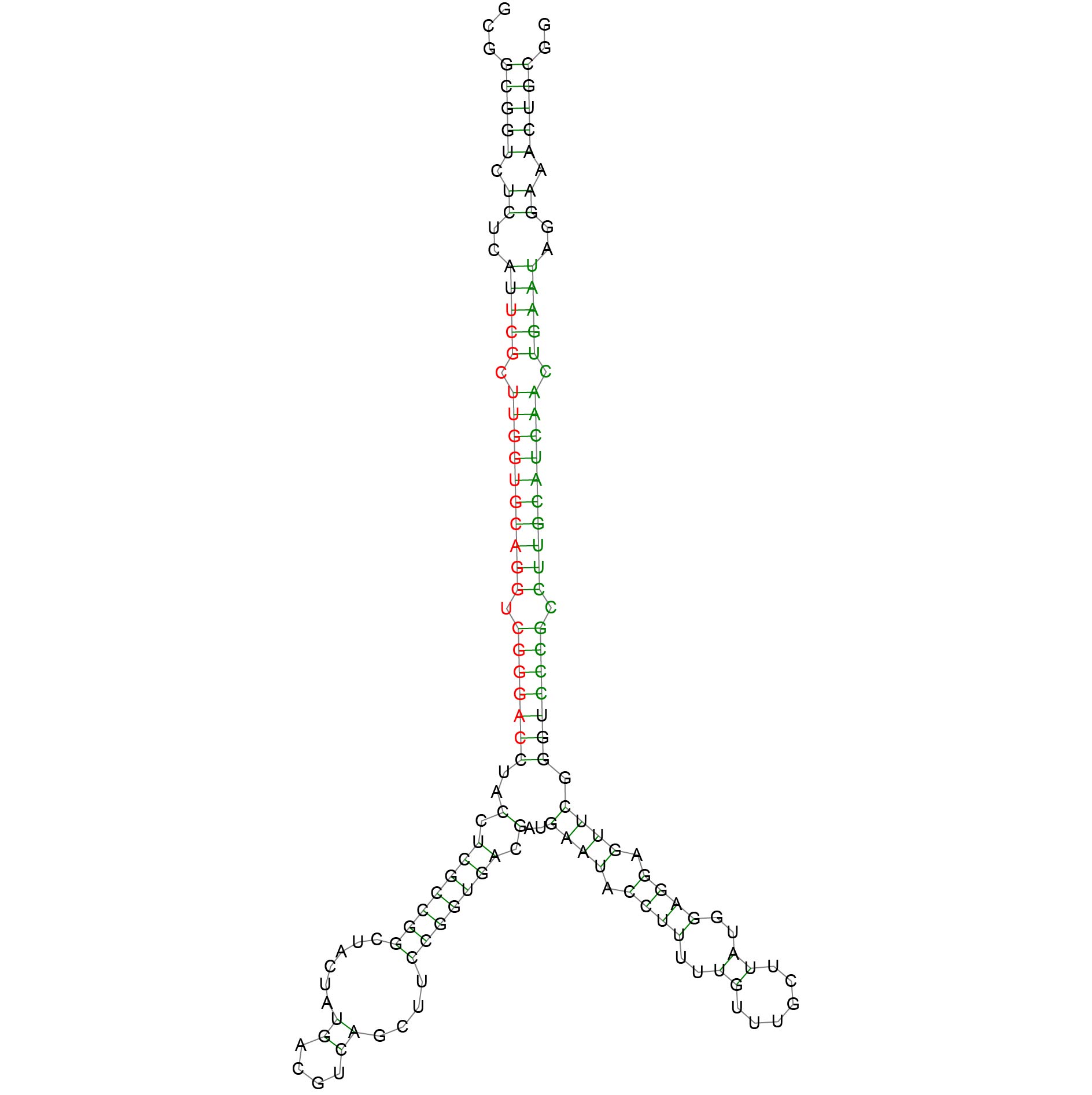

Supplement: Dataset S1 — Full list of hairpin structures in conserved miRNAs. (ZIP) [file pone.0064238.s001.zip › can-miR168b.jpg]

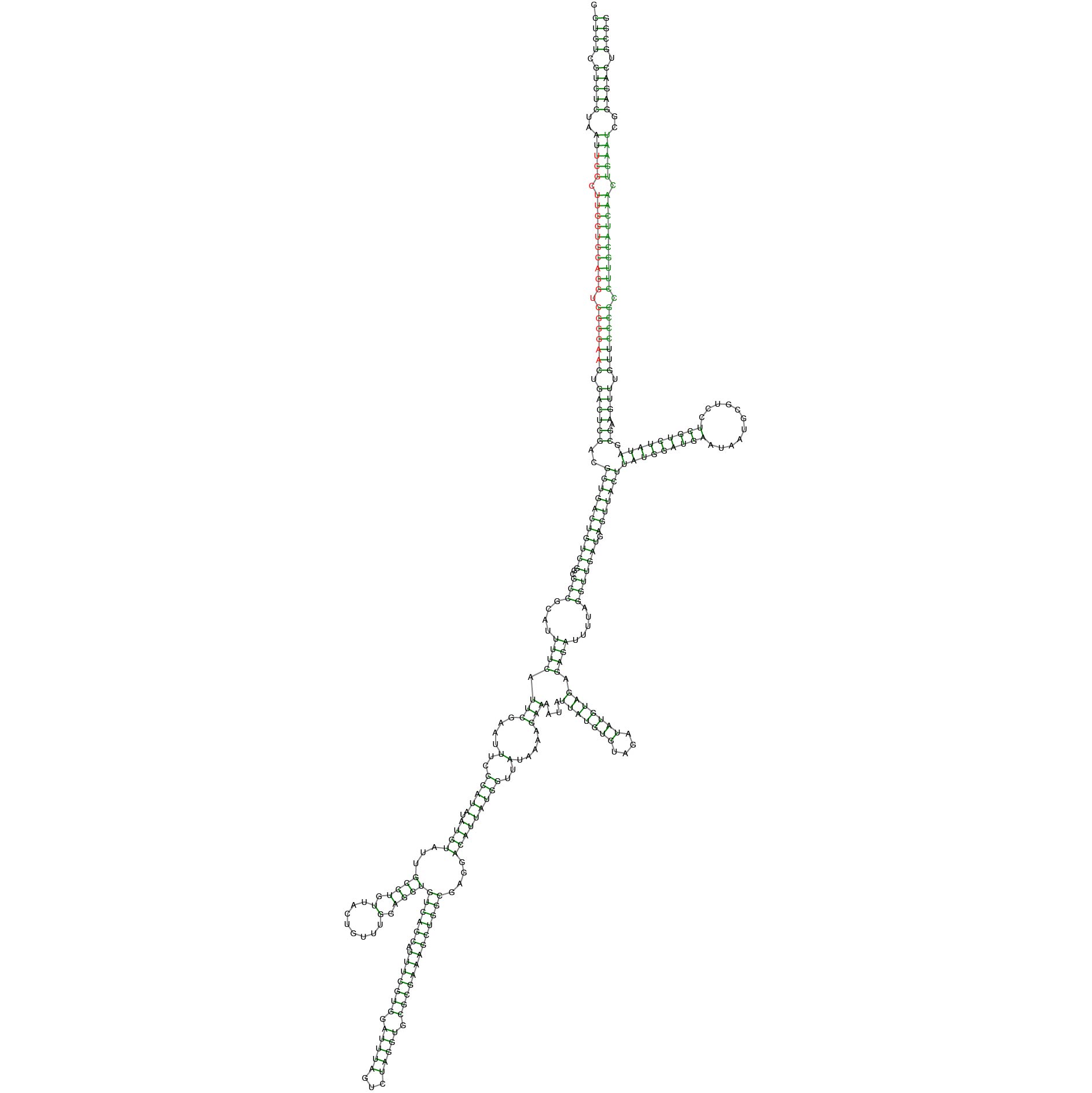

Supplement: Dataset S1 — Full list of hairpin structures in conserved miRNAs. (ZIP) [file pone.0064238.s001.zip › can-miR168c.jpg]

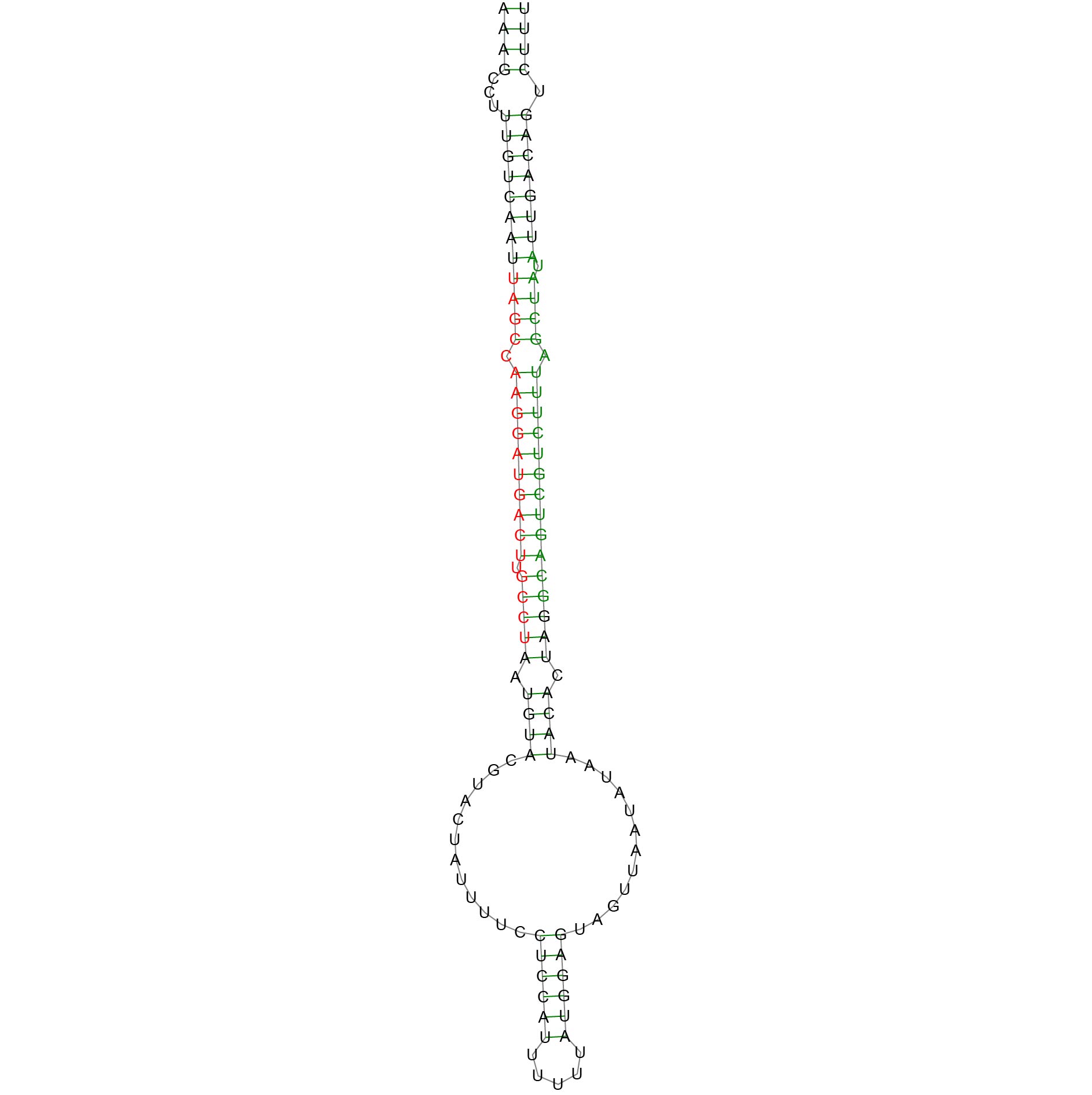

Supplement: Dataset S1 — Full list of hairpin structures in conserved miRNAs. (ZIP) [file pone.0064238.s001.zip › can-miR169a.jpg]

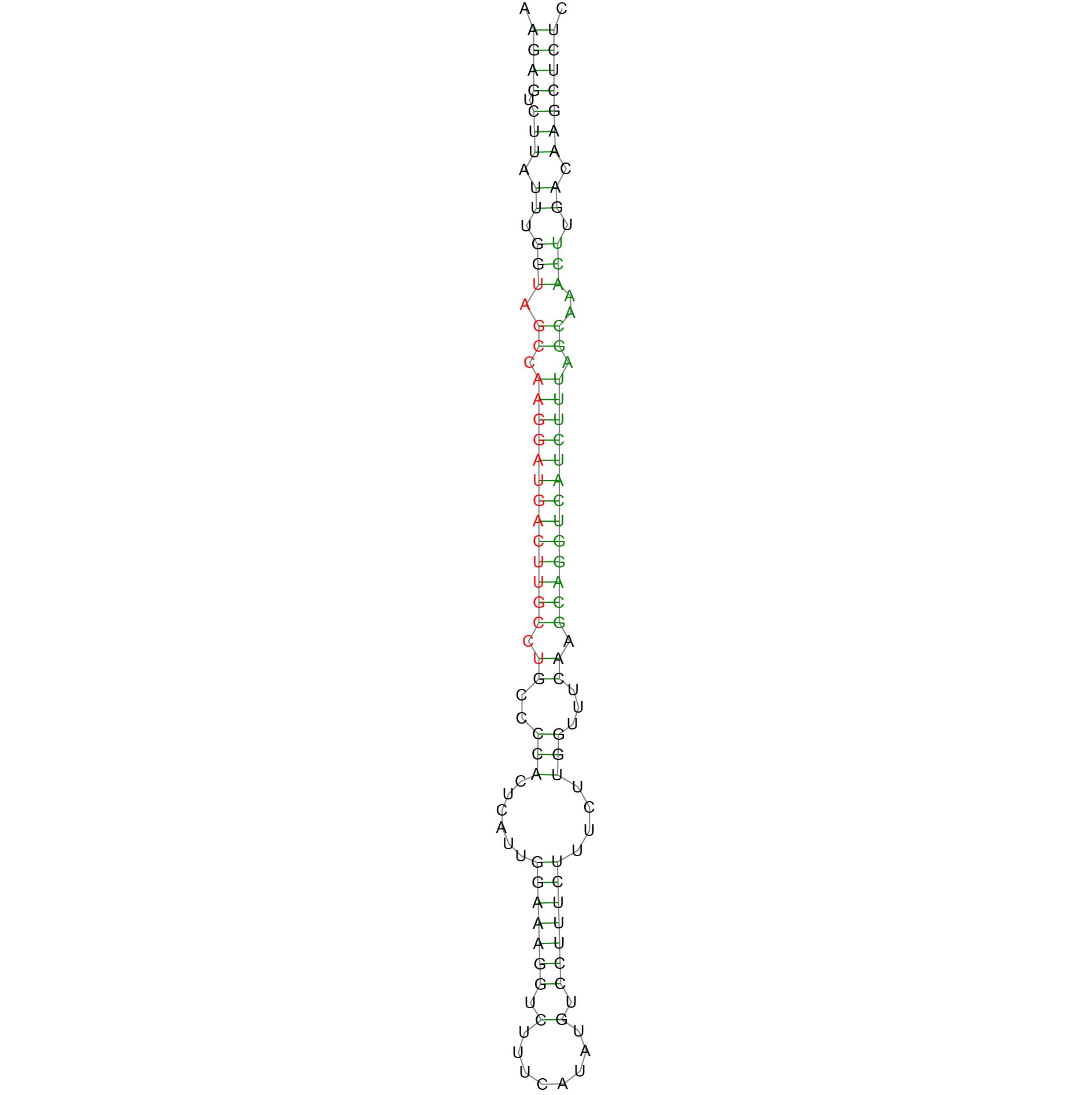

Supplement: Dataset S1 — Full list of hairpin structures in conserved miRNAs. (ZIP) [file pone.0064238.s001.zip › can-miR169b.jpg]

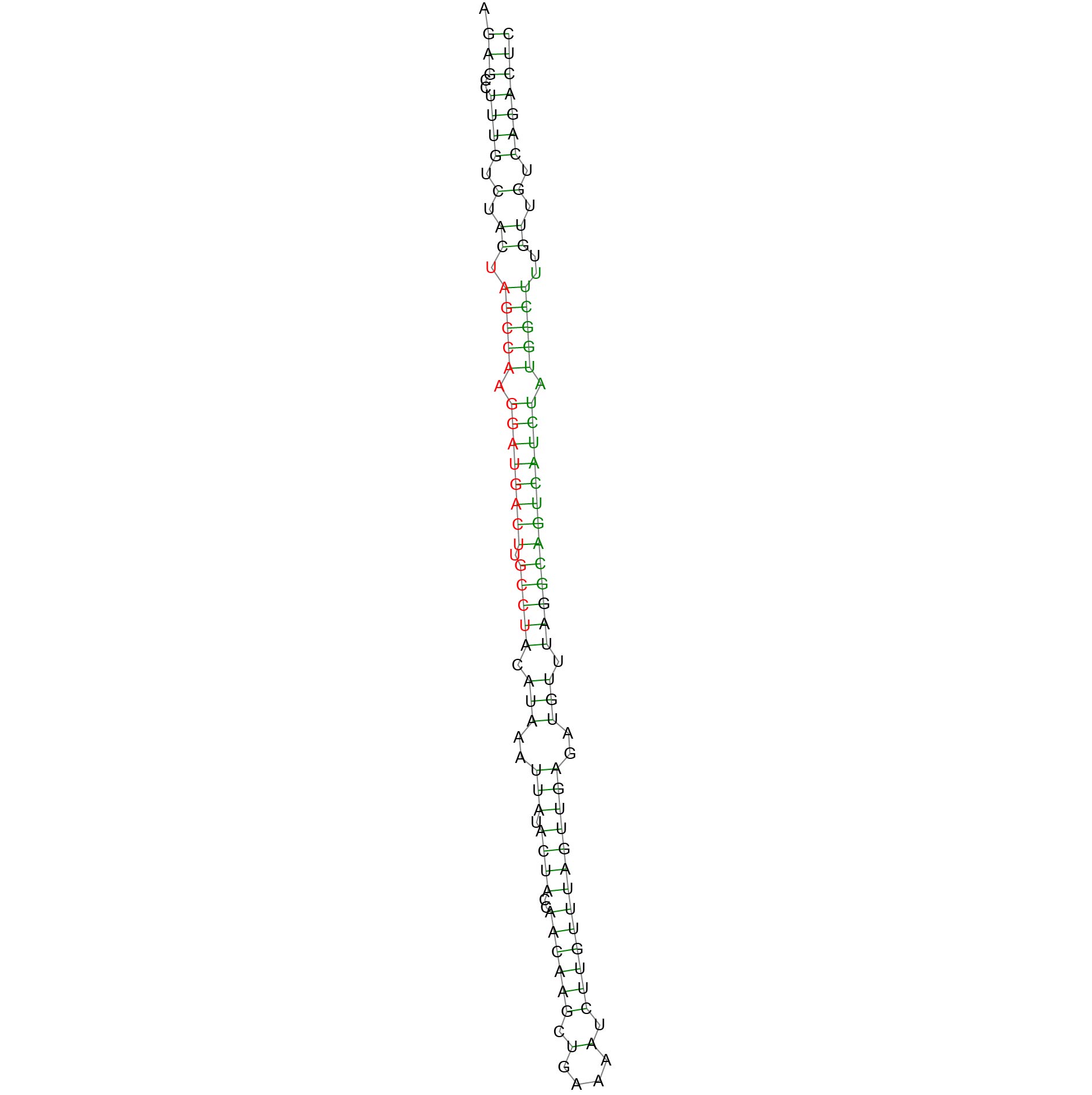

Supplement: Dataset S1 — Full list of hairpin structures in conserved miRNAs. (ZIP) [file pone.0064238.s001.zip › can-miR169c.jpg]

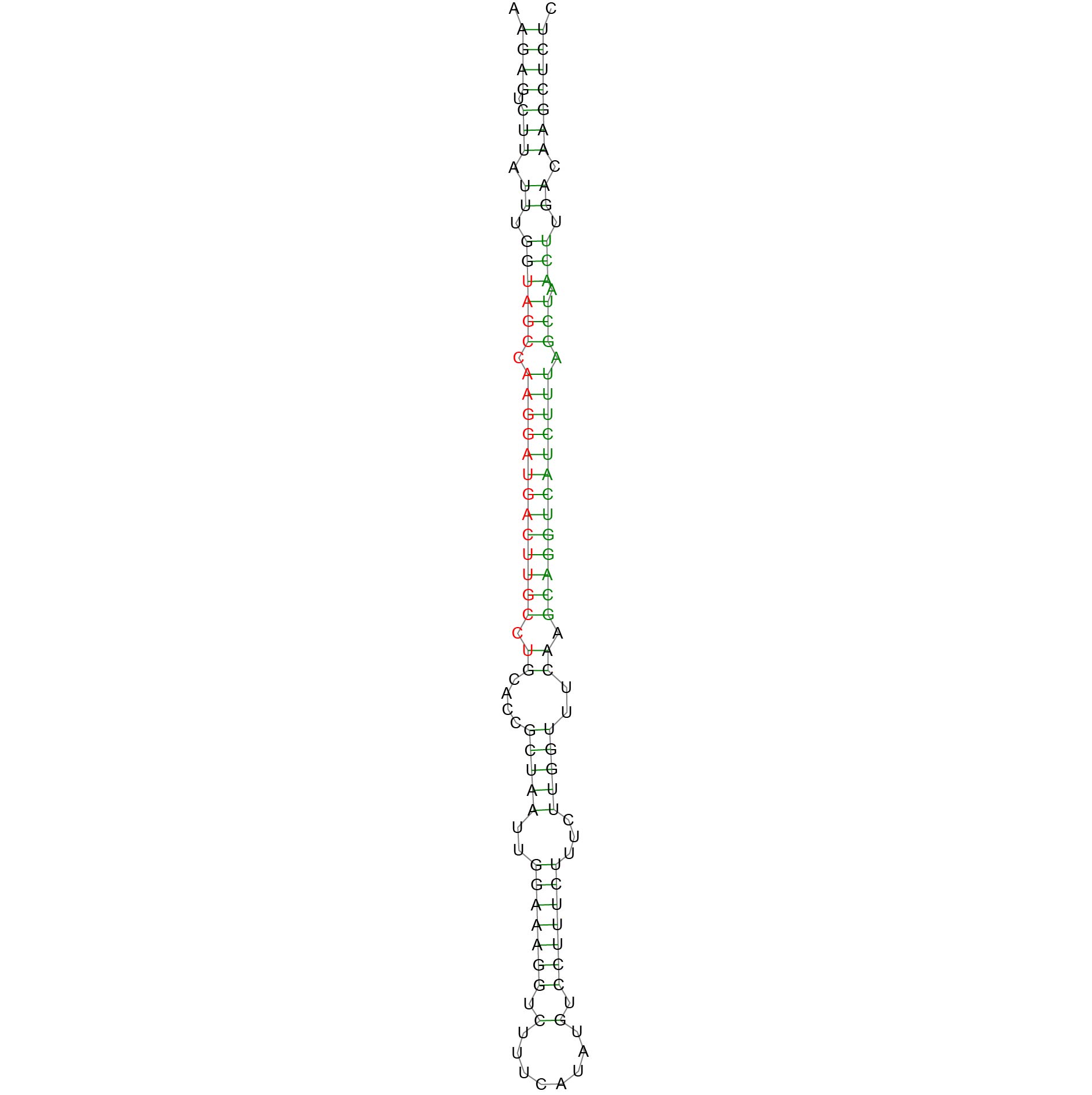

Supplement: Dataset S1 — Full list of hairpin structures in conserved miRNAs. (ZIP) [file pone.0064238.s001.zip › can-miR169d.jpg]

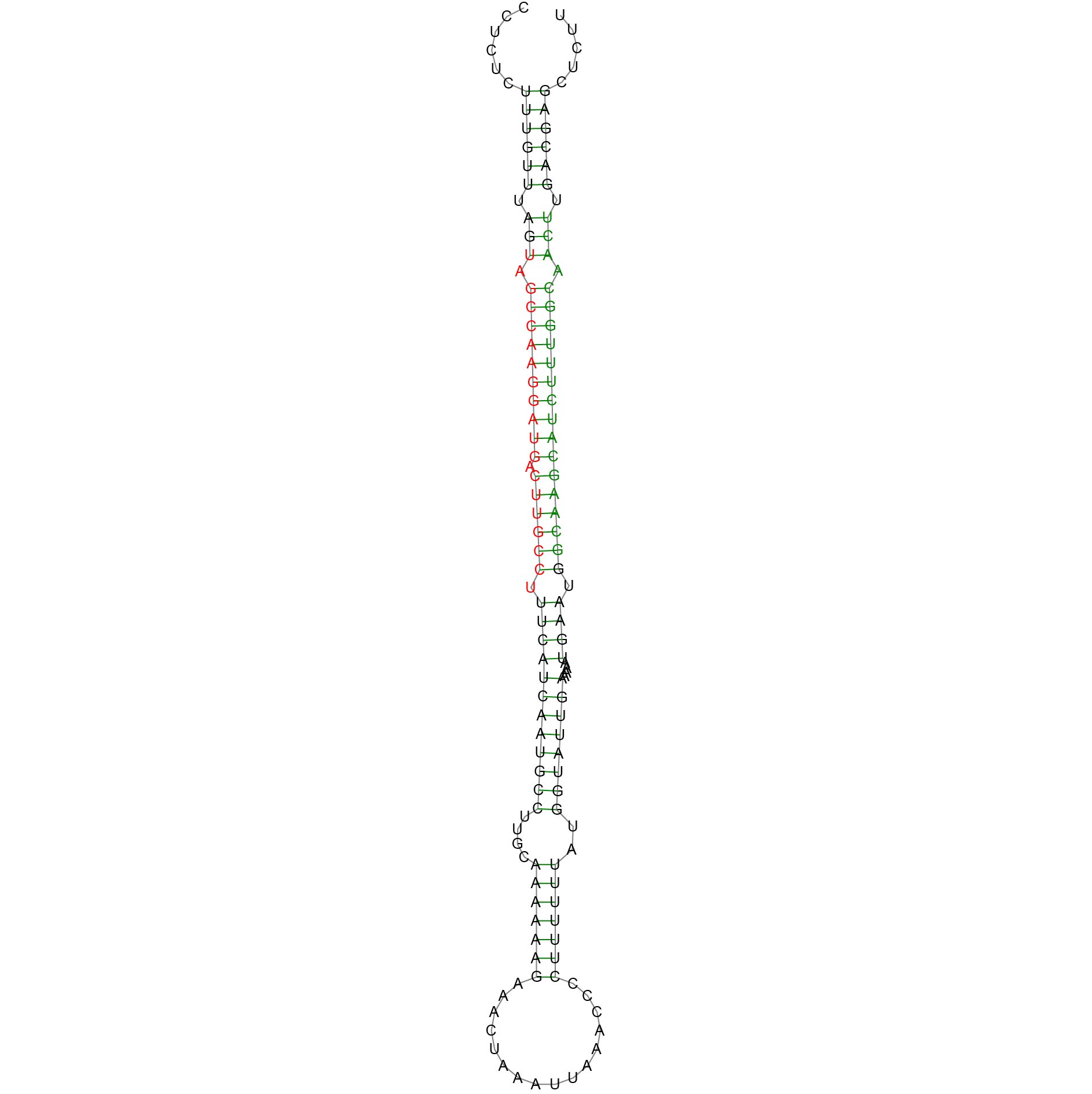

Supplement: Dataset S1 — Full list of hairpin structures in conserved miRNAs. (ZIP) [file pone.0064238.s001.zip › can-miR169e.jpg]

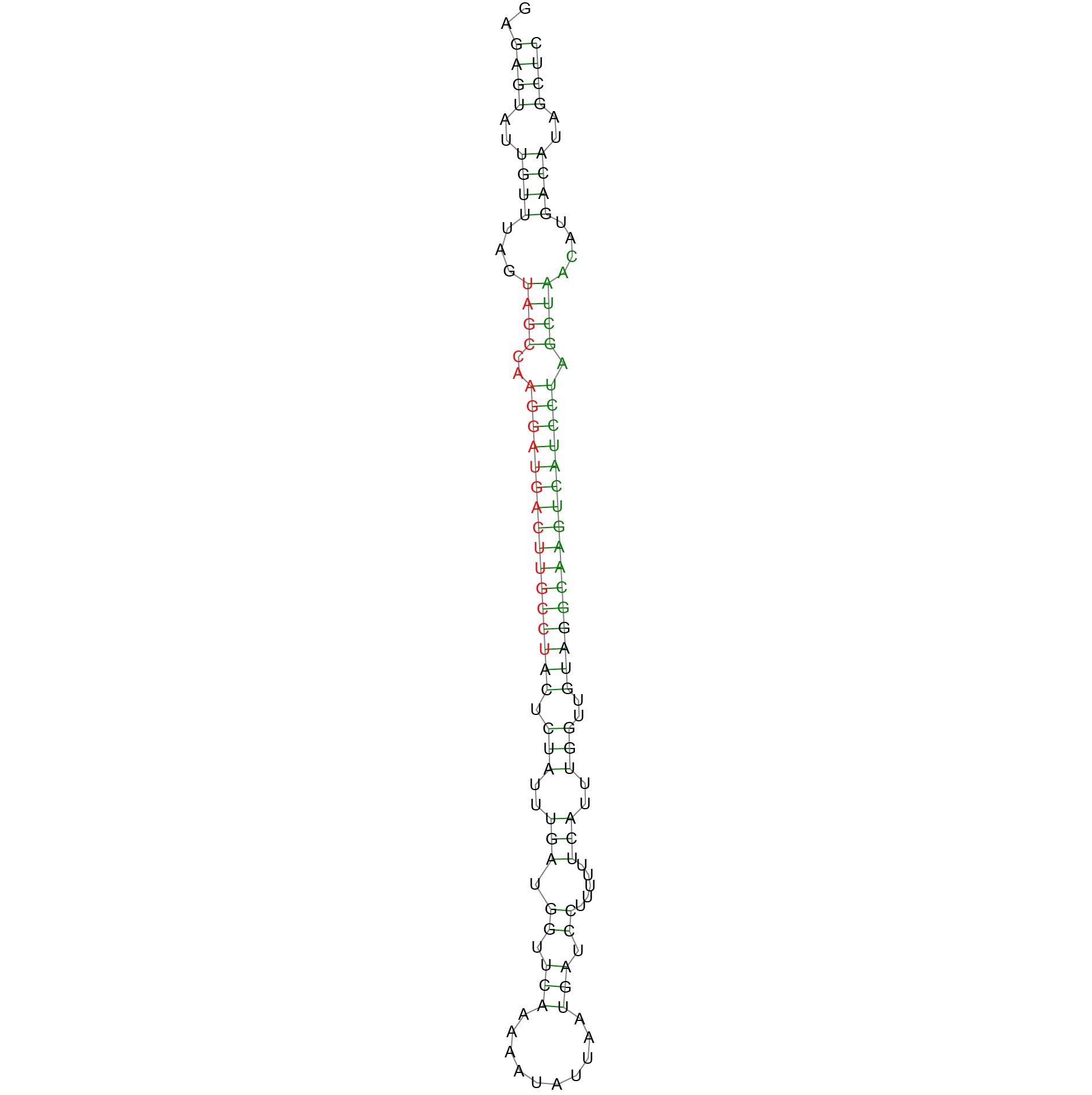

Supplement: Dataset S1 — Full list of hairpin structures in conserved miRNAs. (ZIP) [file pone.0064238.s001.zip › can-miR169f.jpg]

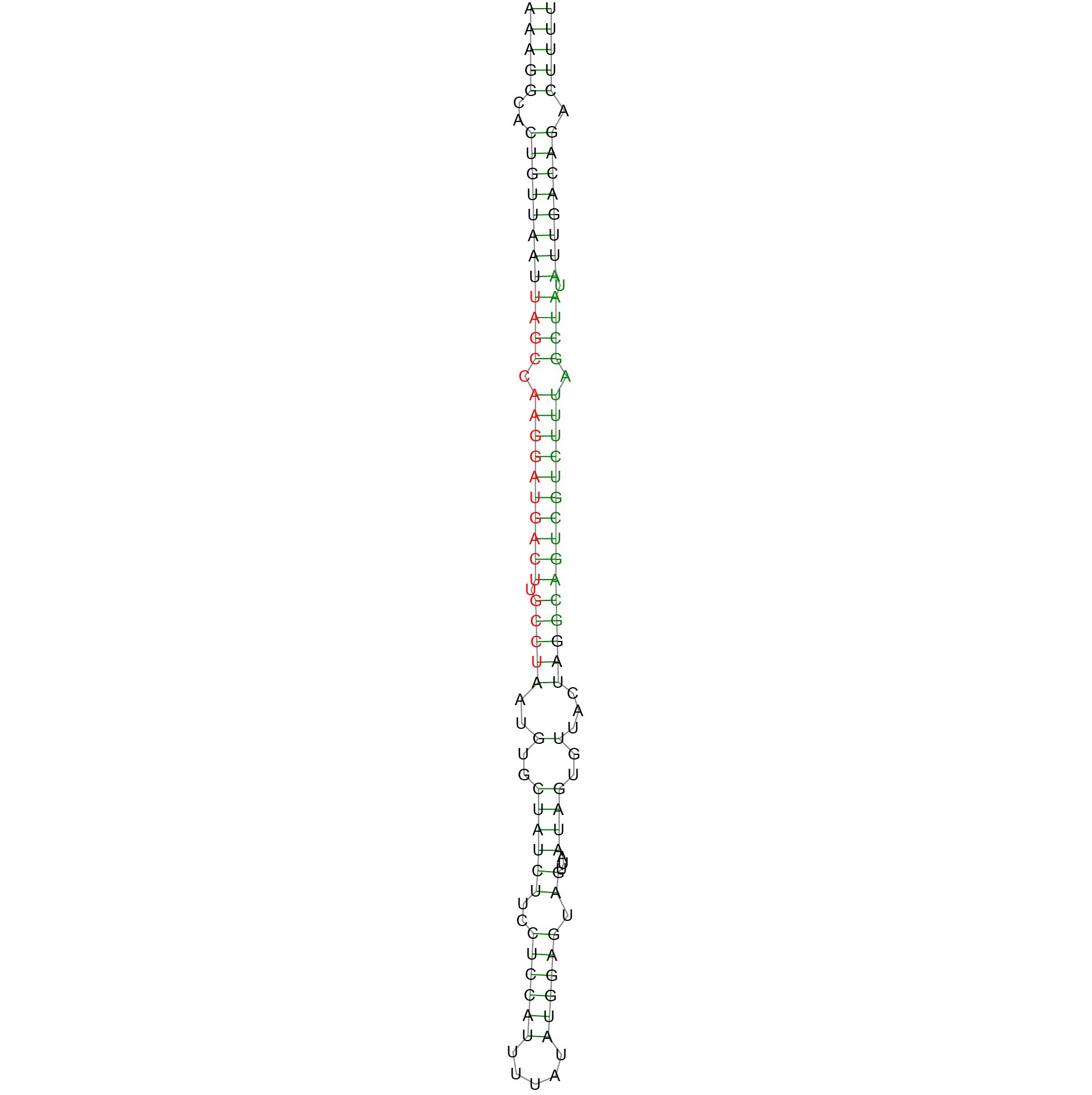

Supplement: Dataset S1 — Full list of hairpin structures in conserved miRNAs. (ZIP) [file pone.0064238.s001.zip › can-miR169g.jpg]

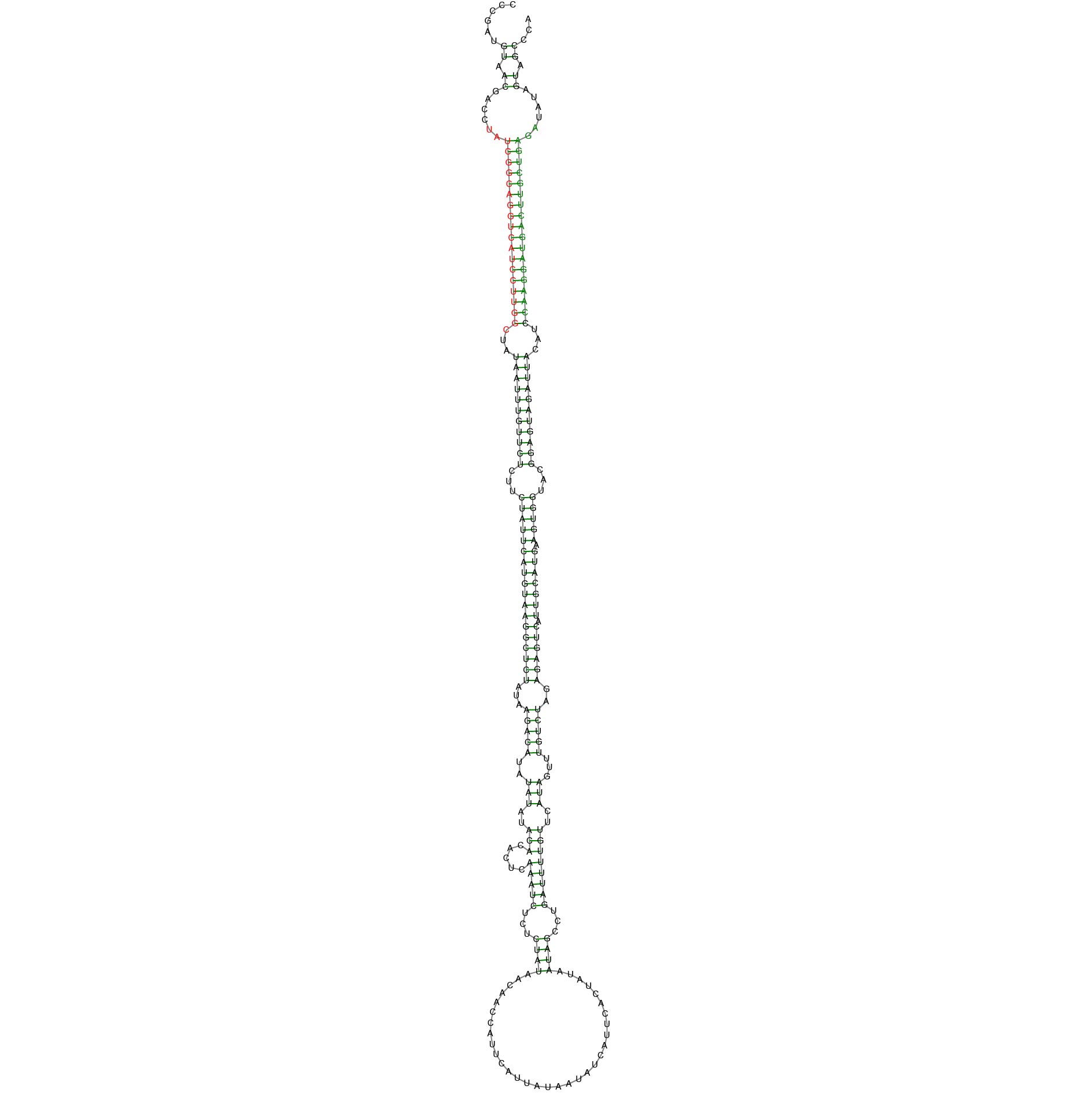

Supplement: Dataset S1 — Full list of hairpin structures in conserved miRNAs. (ZIP) [file pone.0064238.s001.zip › can-miR169h.jpg]

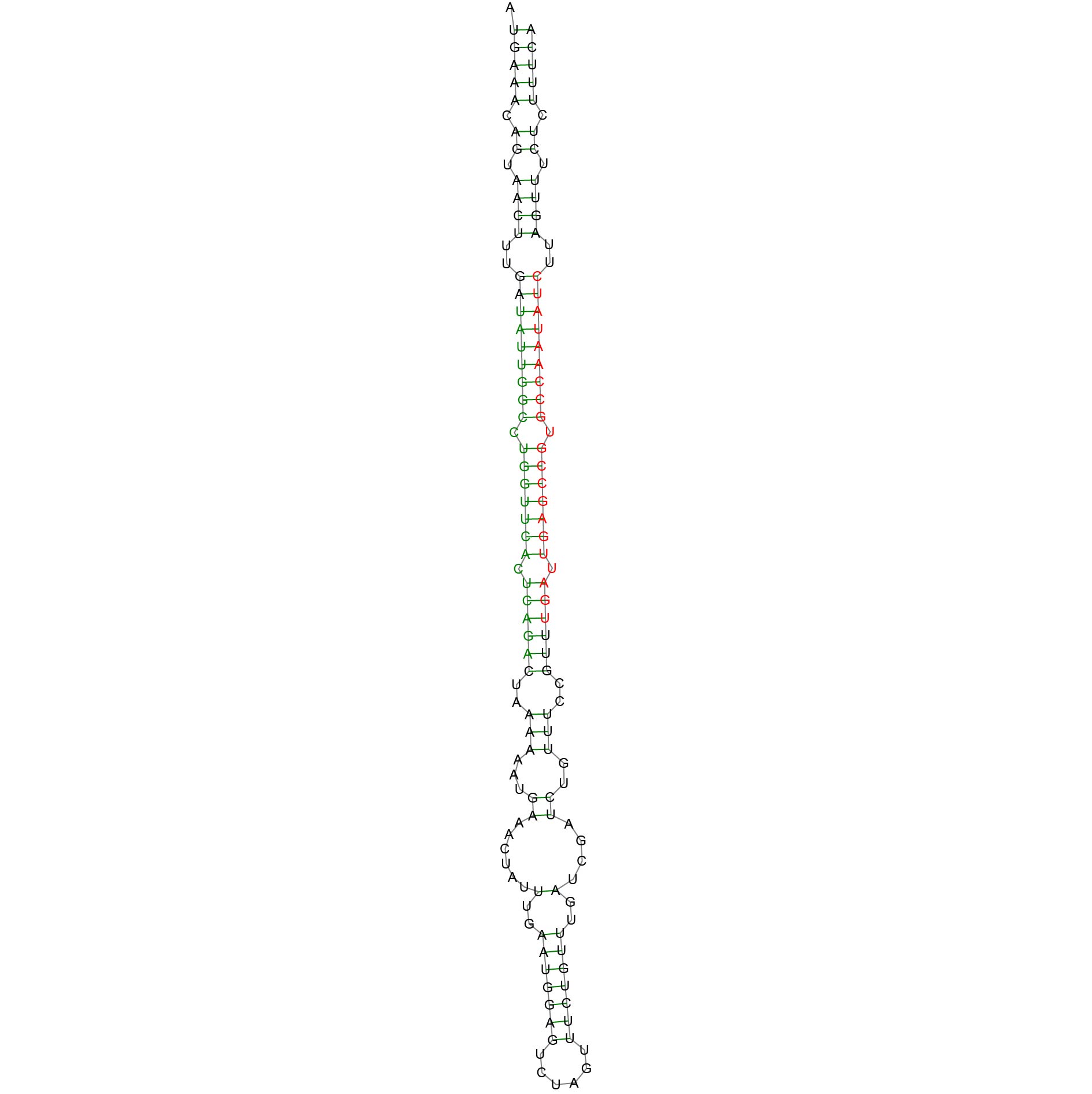

Supplement: Dataset S1 — Full list of hairpin structures in conserved miRNAs. (ZIP) [file pone.0064238.s001.zip › can-miR171a.jpg]

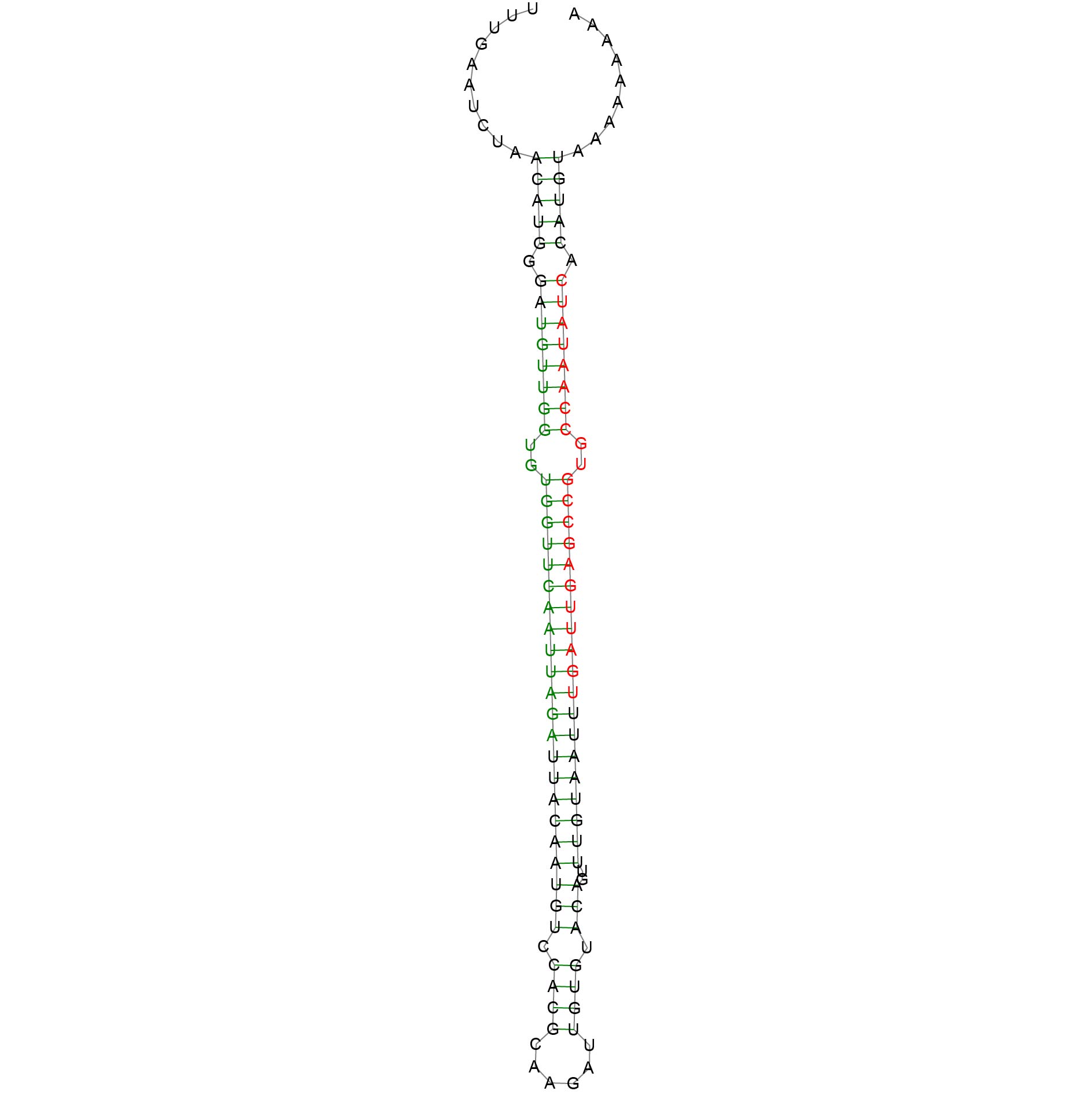

Supplement: Dataset S1 — Full list of hairpin structures in conserved miRNAs. (ZIP) [file pone.0064238.s001.zip › can-miR171b.jpg]

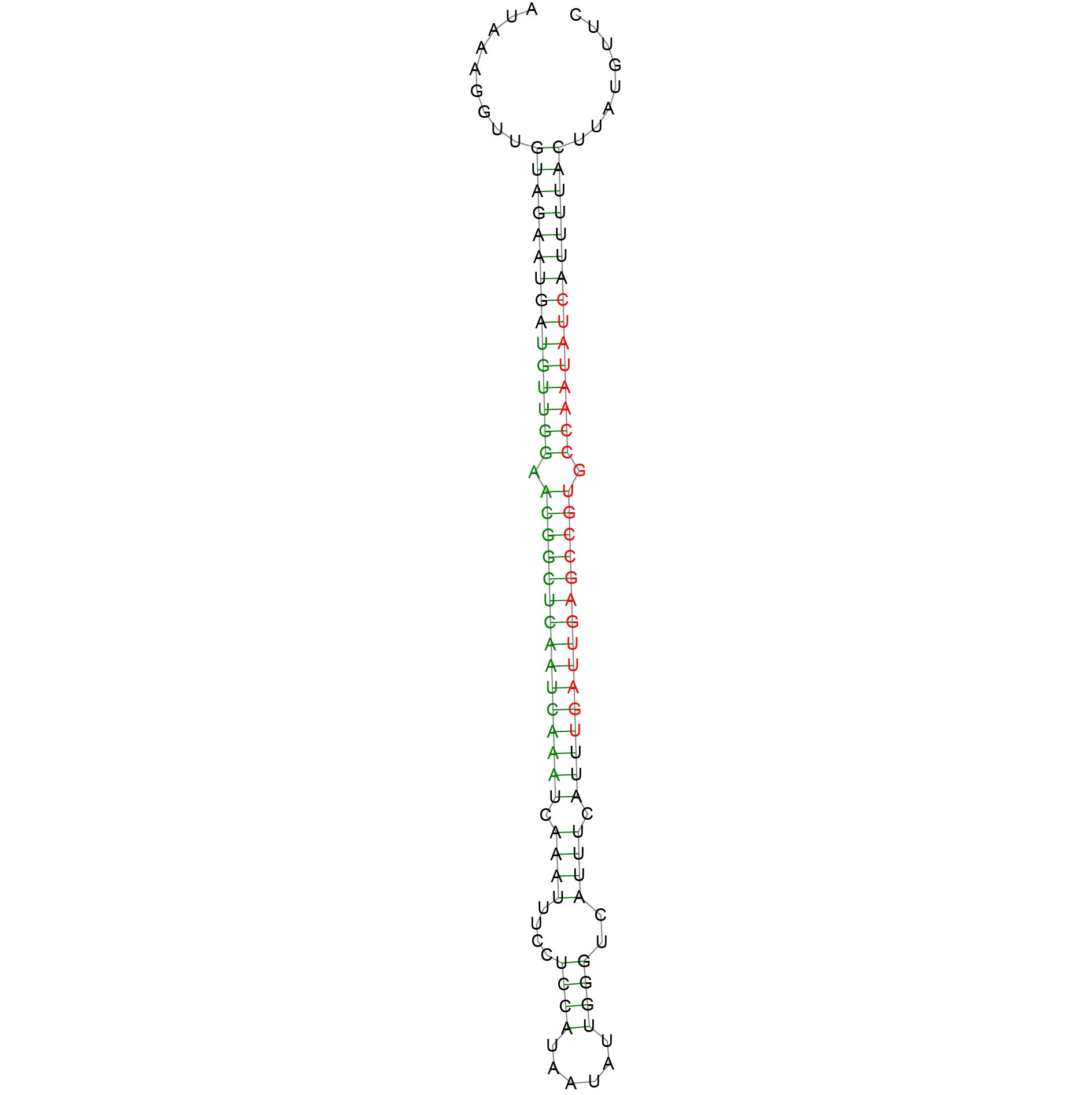

Supplement: Dataset S1 — Full list of hairpin structures in conserved miRNAs. (ZIP) [file pone.0064238.s001.zip › can-miR171c.jpg]

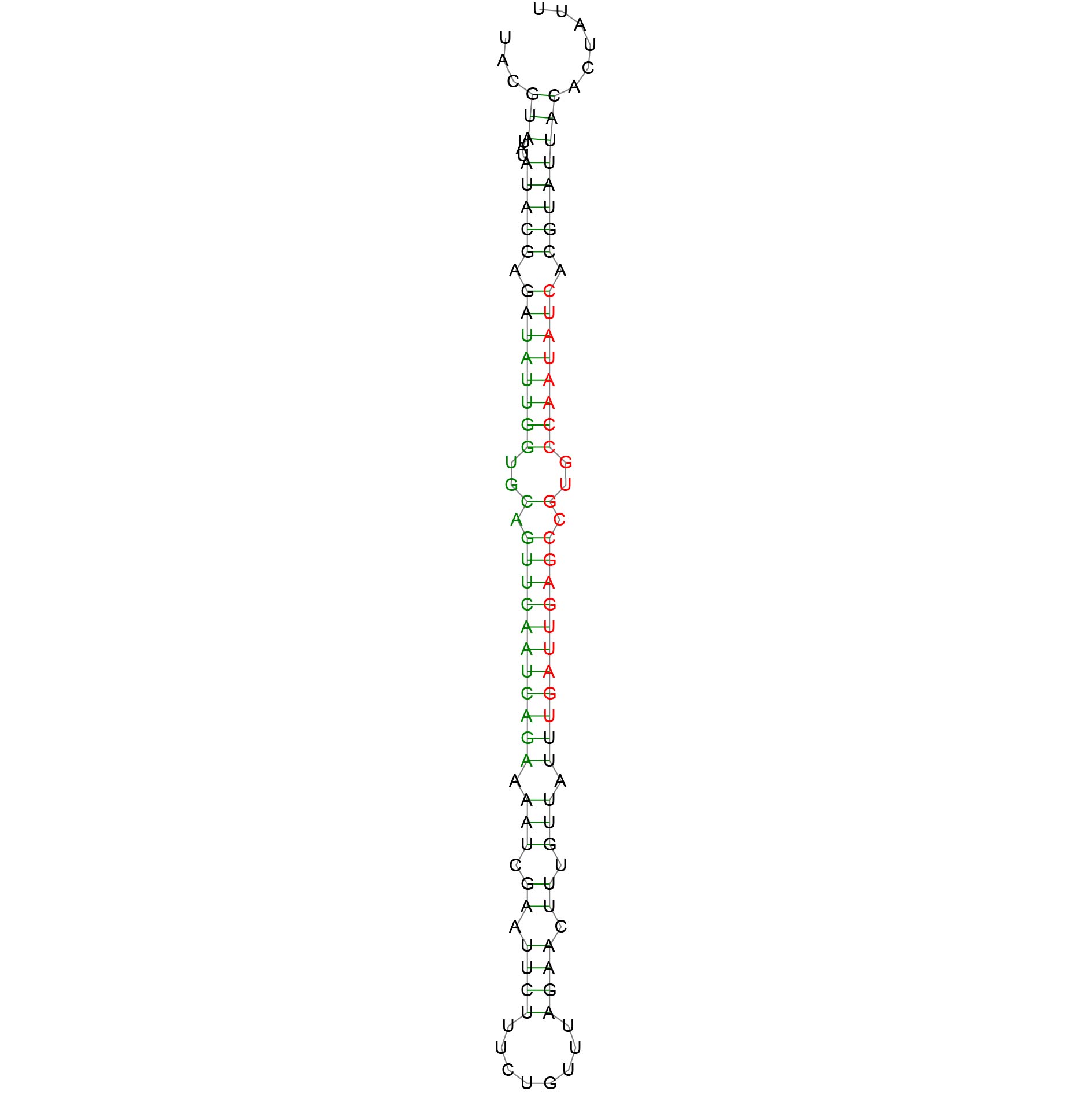

Supplement: Dataset S1 — Full list of hairpin structures in conserved miRNAs. (ZIP) [file pone.0064238.s001.zip › can-miR171d.jpg]

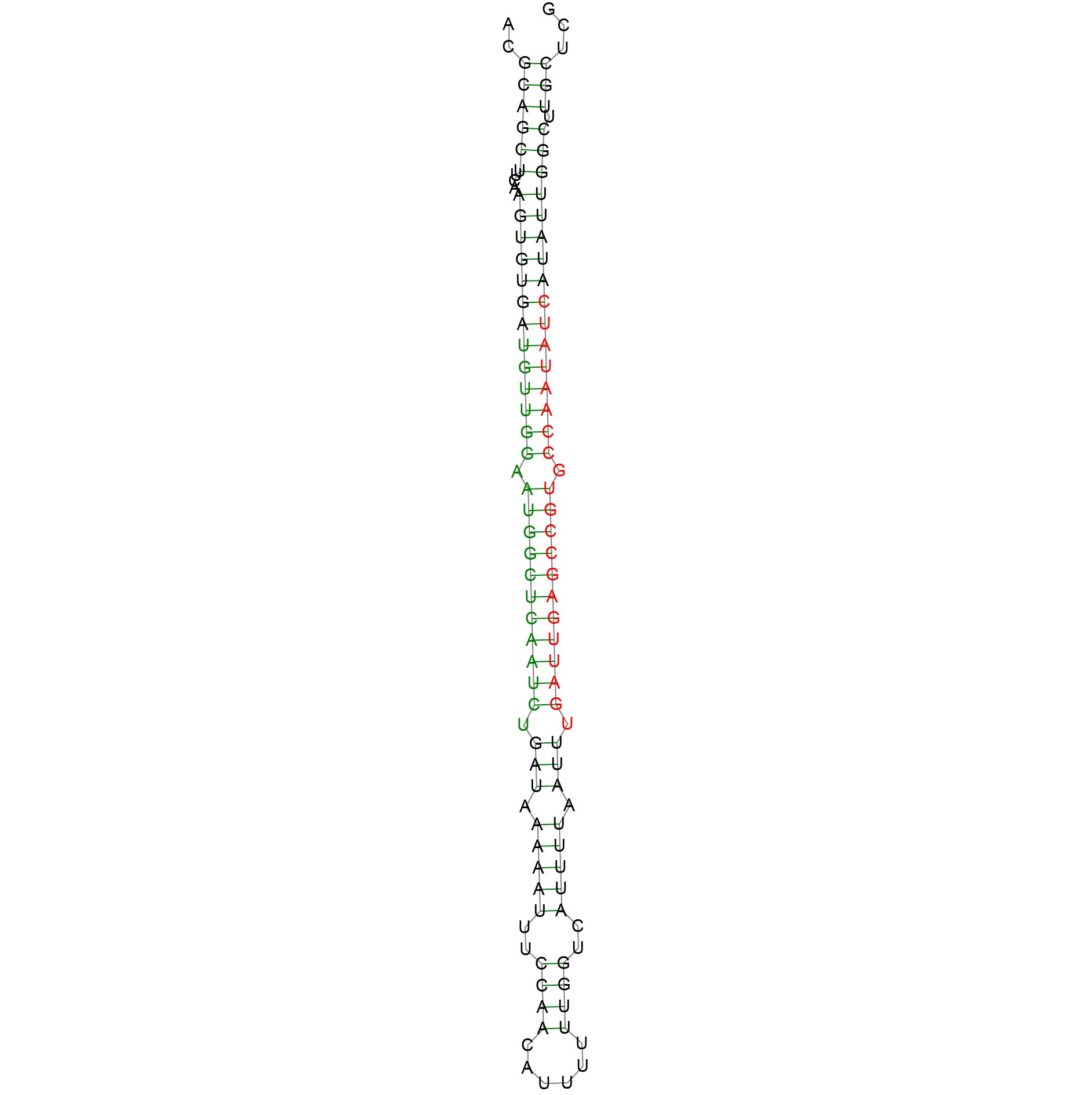

Supplement: Dataset S1 — Full list of hairpin structures in conserved miRNAs. (ZIP) [file pone.0064238.s001.zip › can-miR171e.jpg]

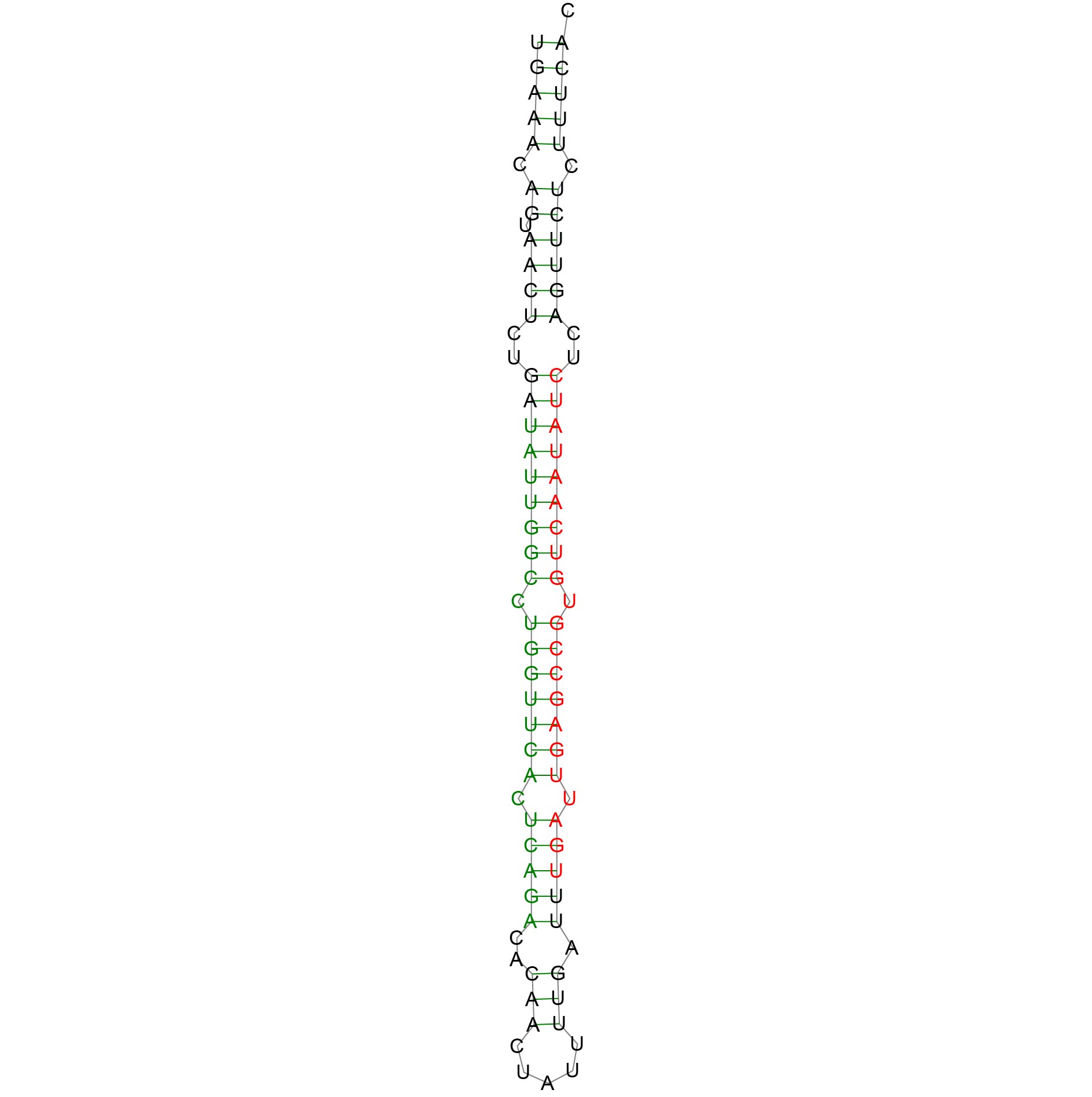

Supplement: Dataset S1 — Full list of hairpin structures in conserved miRNAs. (ZIP) [file pone.0064238.s001.zip › can-miR171f.jpg]

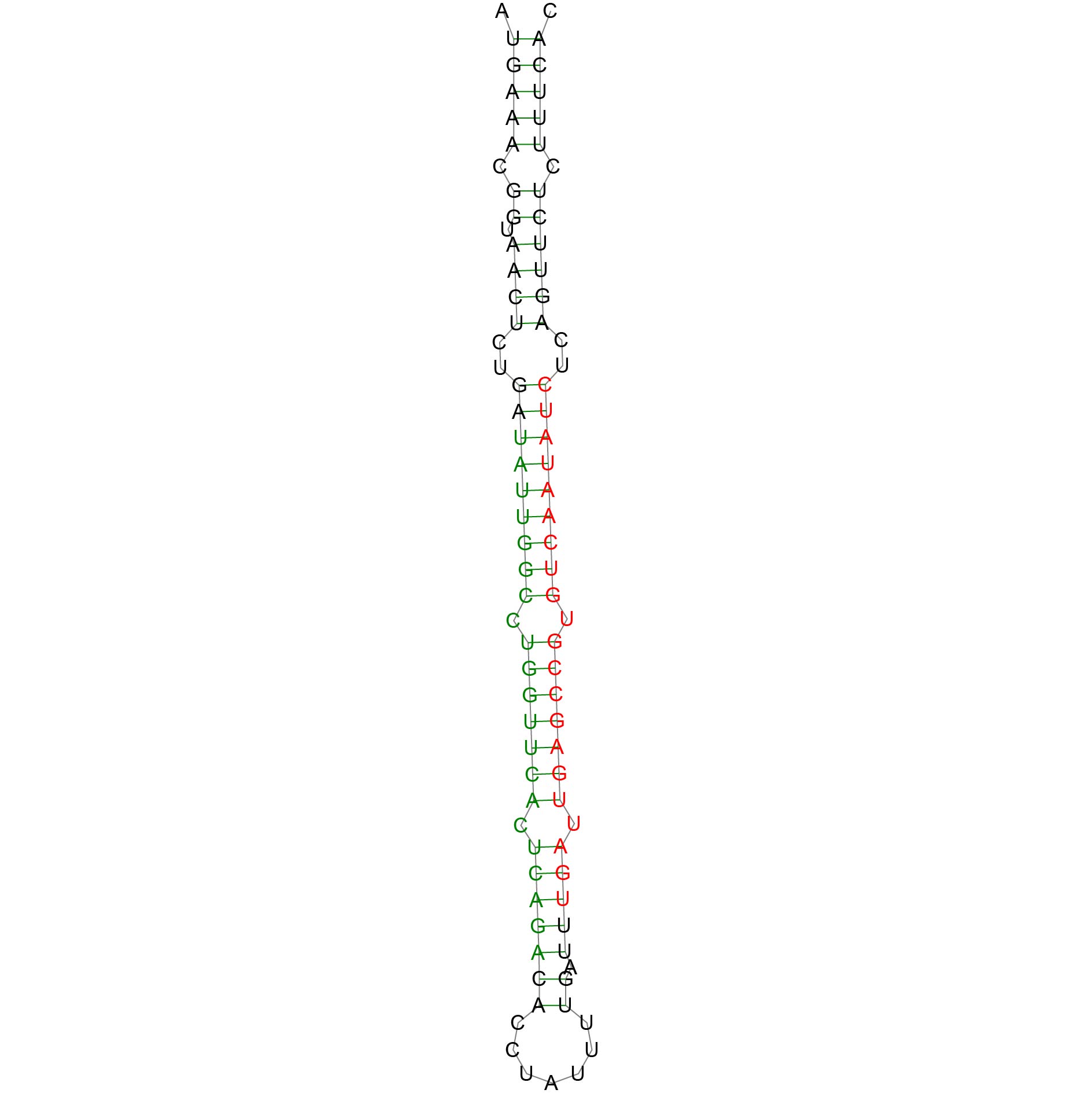

Supplement: Dataset S1 — Full list of hairpin structures in conserved miRNAs. (ZIP) [file pone.0064238.s001.zip › can-miR171g.jpg]

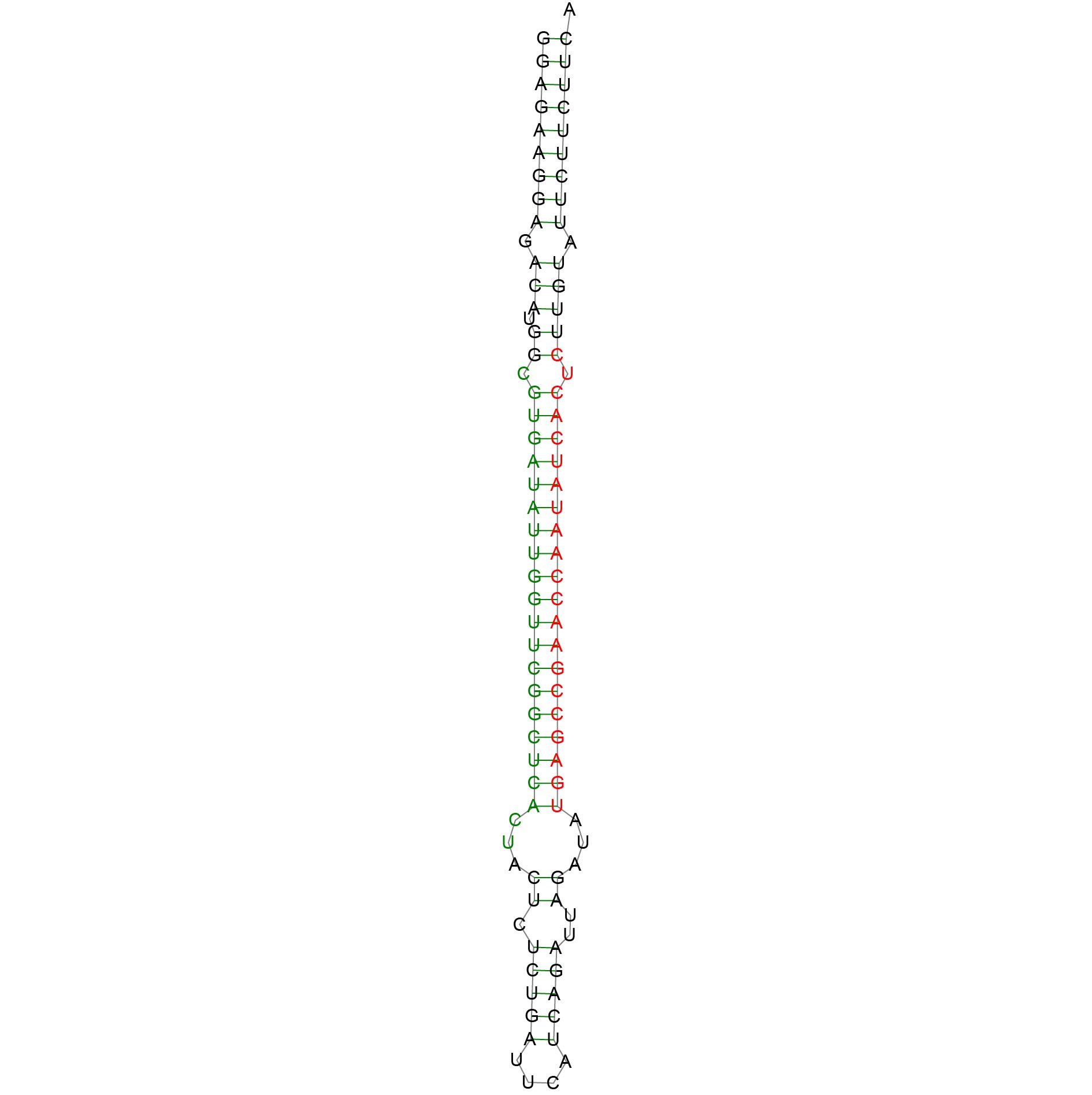

Supplement: Dataset S1 — Full list of hairpin structures in conserved miRNAs. (ZIP) [file pone.0064238.s001.zip › can-miR171h.jpg]

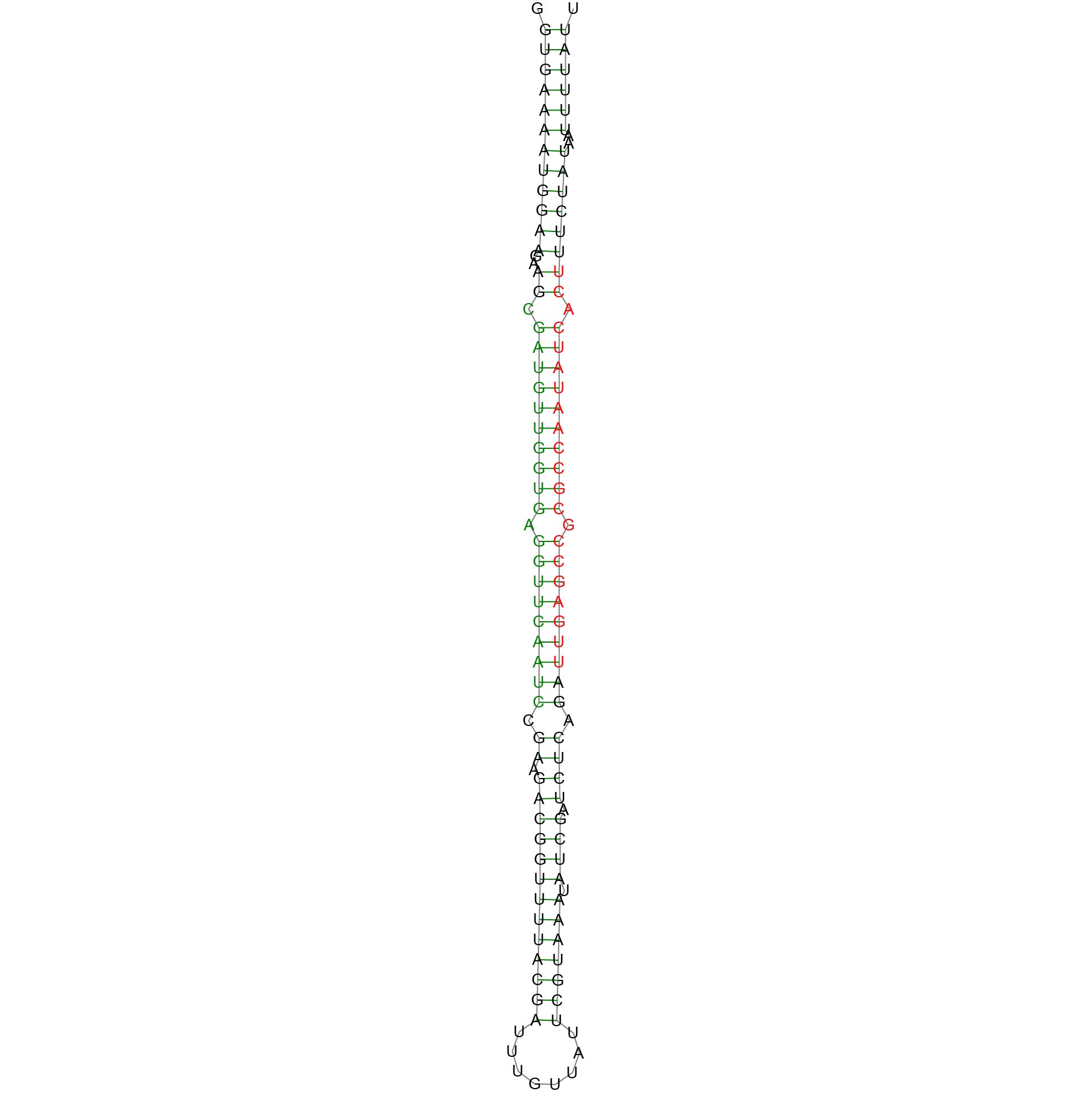

Supplement: Dataset S1 — Full list of hairpin structures in conserved miRNAs. (ZIP) [file pone.0064238.s001.zip › can-miR171i.jpg]

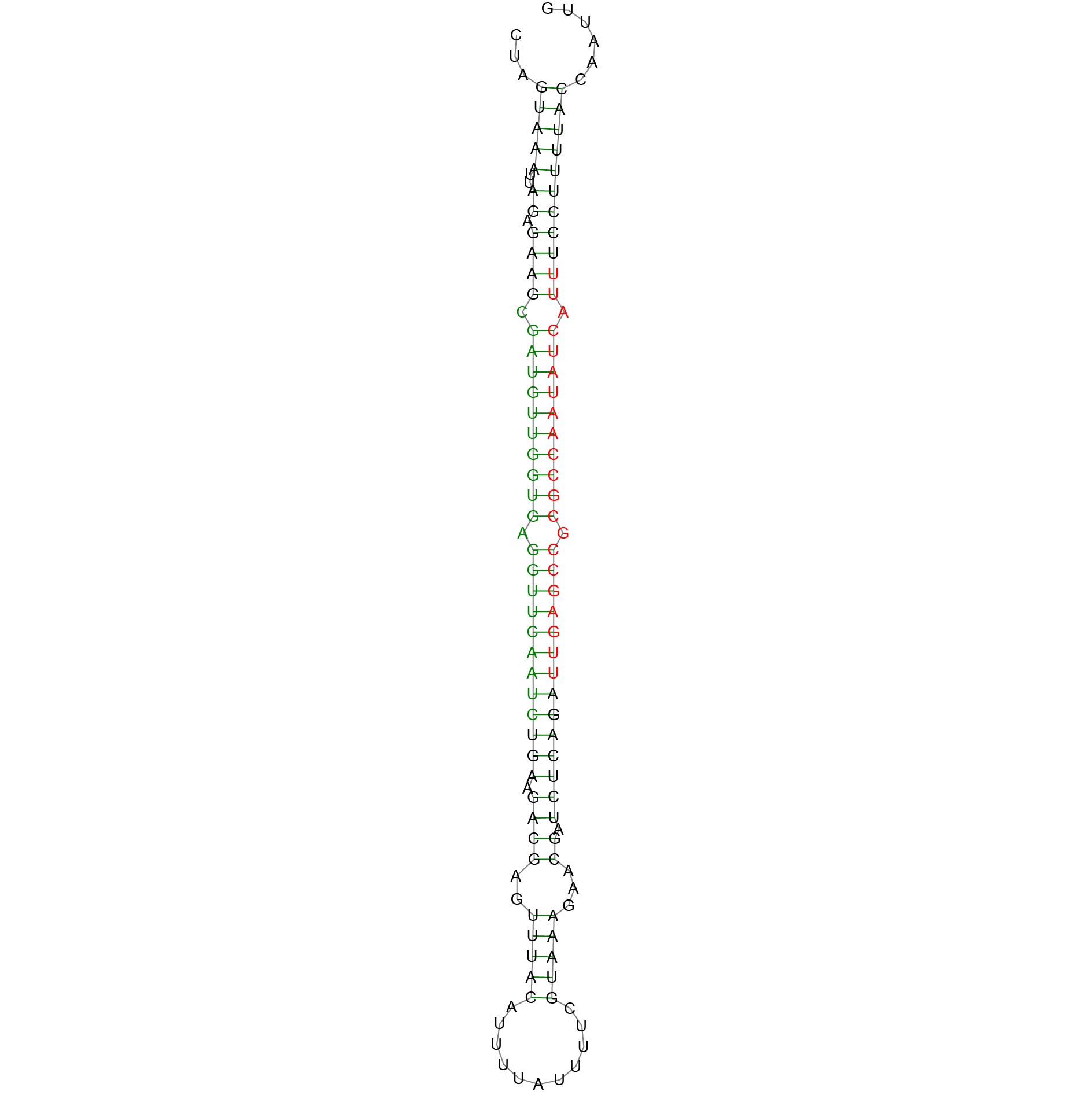

Supplement: Dataset S1 — Full list of hairpin structures in conserved miRNAs. (ZIP) [file pone.0064238.s001.zip › can-miR171j.jpg]

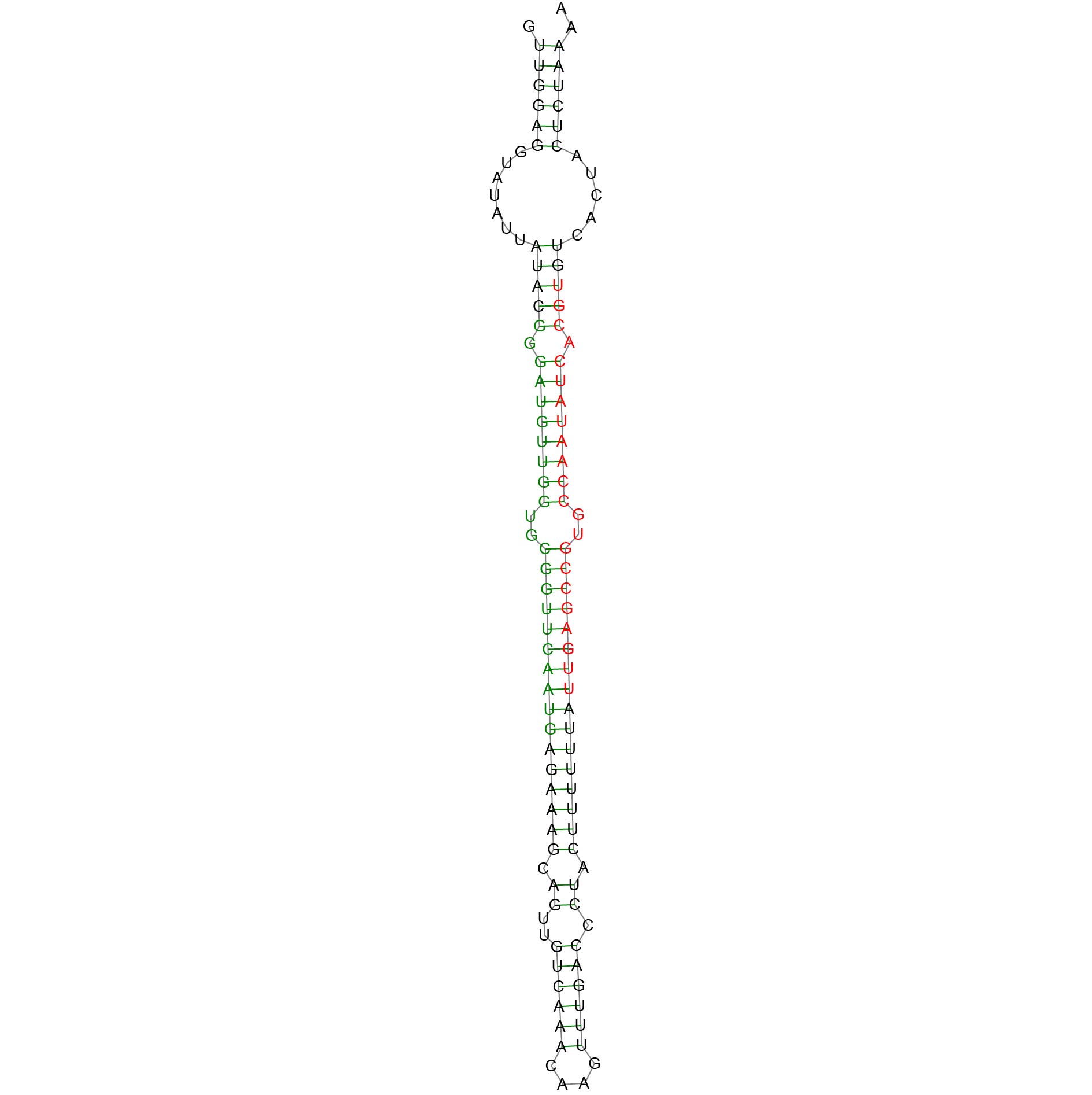

Supplement: Dataset S1 — Full list of hairpin structures in conserved miRNAs. (ZIP) [file pone.0064238.s001.zip › can-miR171k.jpg]

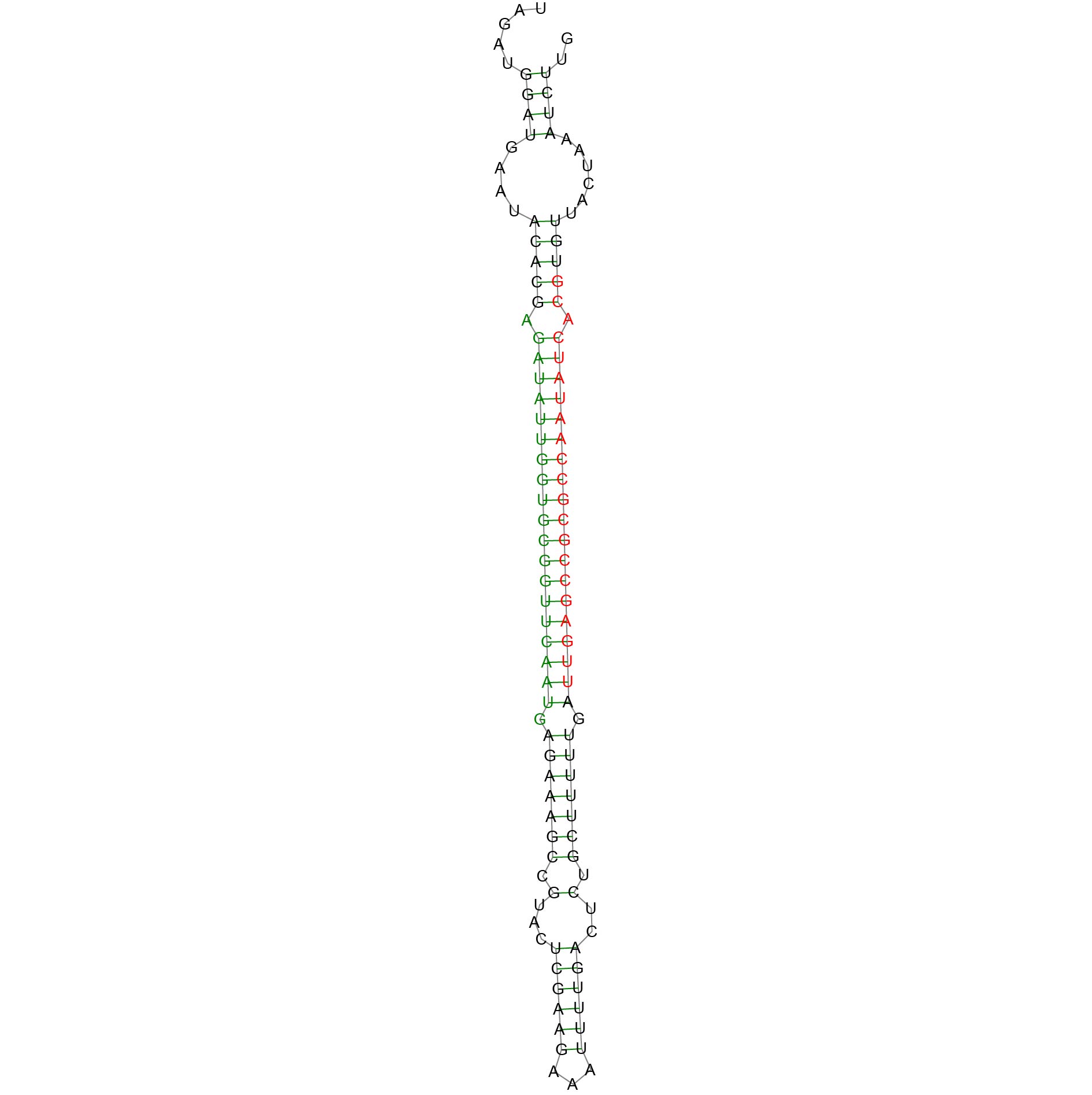

Supplement: Dataset S1 — Full list of hairpin structures in conserved miRNAs. (ZIP) [file pone.0064238.s001.zip › can-miR171l.jpg]

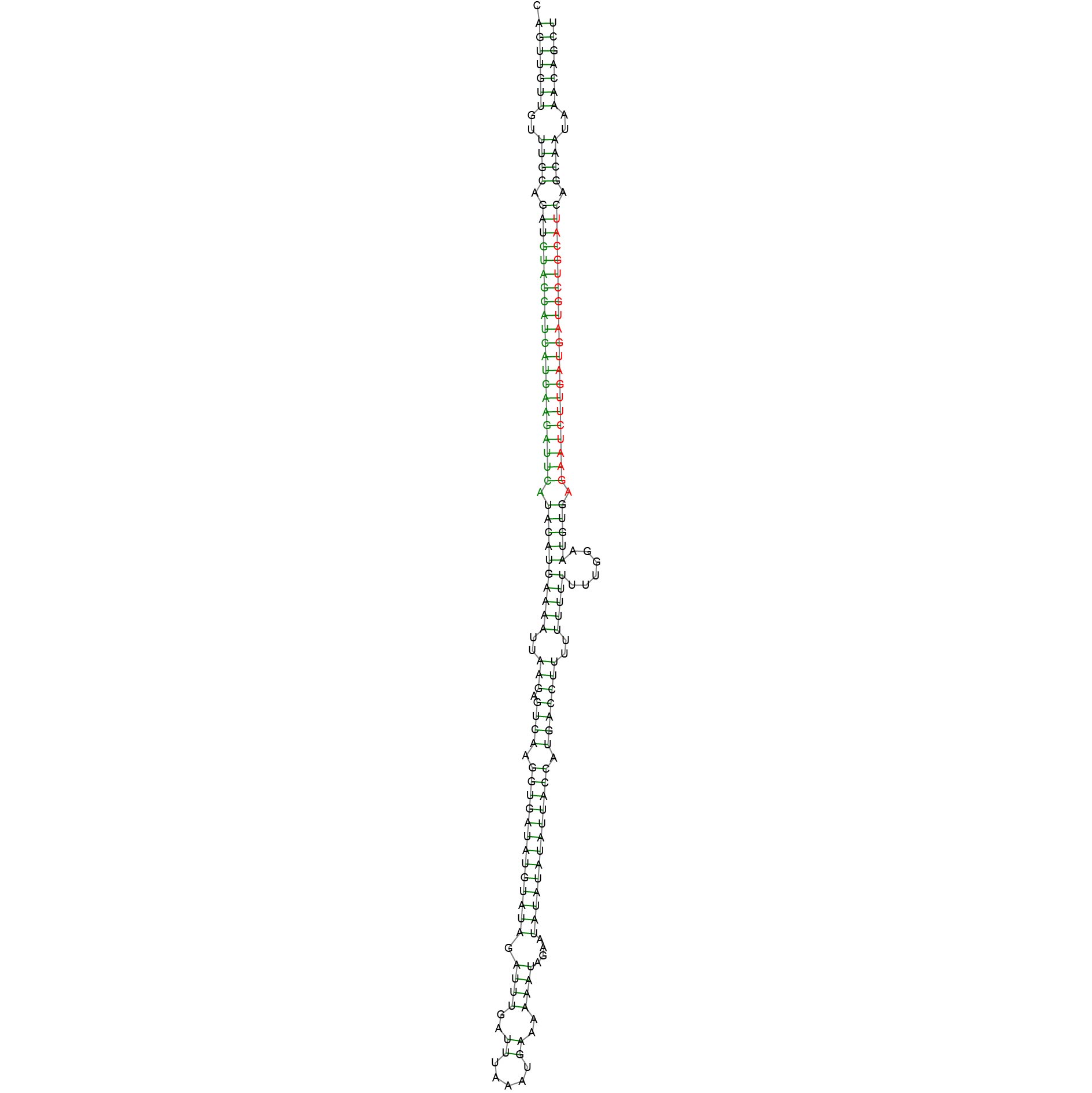

Supplement: Dataset S1 — Full list of hairpin structures in conserved miRNAs. (ZIP) [file pone.0064238.s001.zip › can-miR172a.jpg]

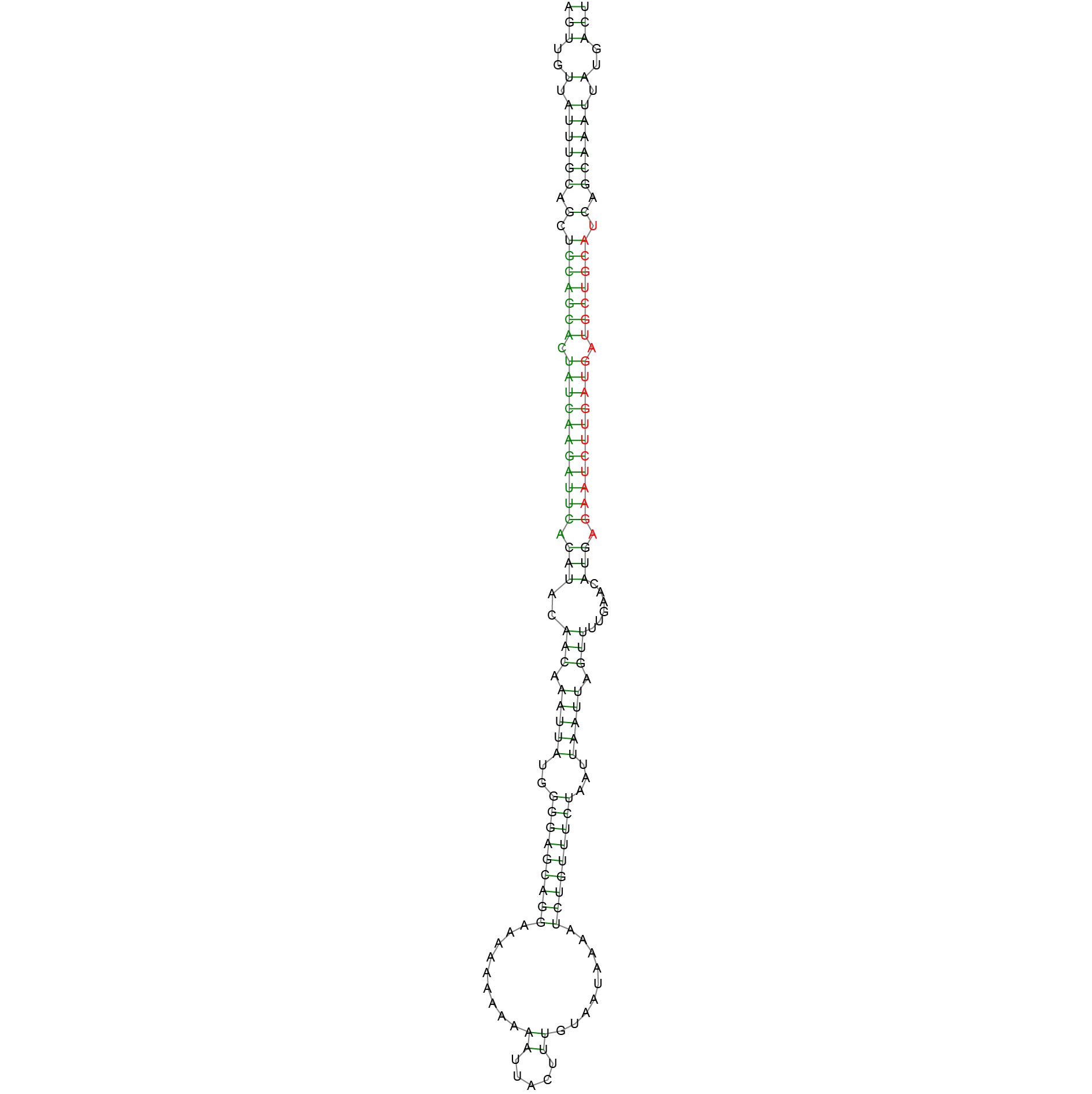

Supplement: Dataset S1 — Full list of hairpin structures in conserved miRNAs. (ZIP) [file pone.0064238.s001.zip › can-miR172b.jpg]

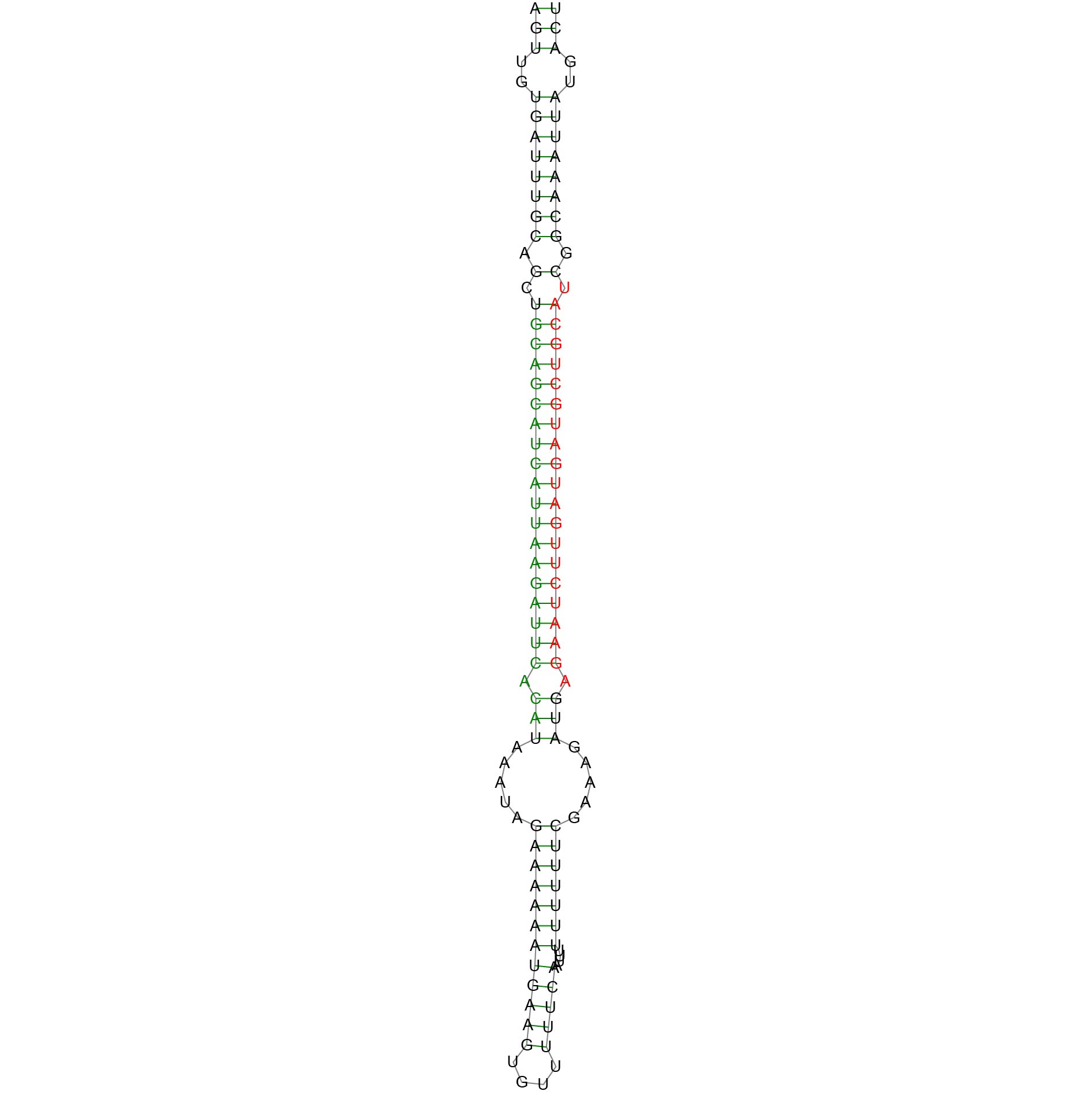

Supplement: Dataset S1 — Full list of hairpin structures in conserved miRNAs. (ZIP) [file pone.0064238.s001.zip › can-miR172c.jpg]

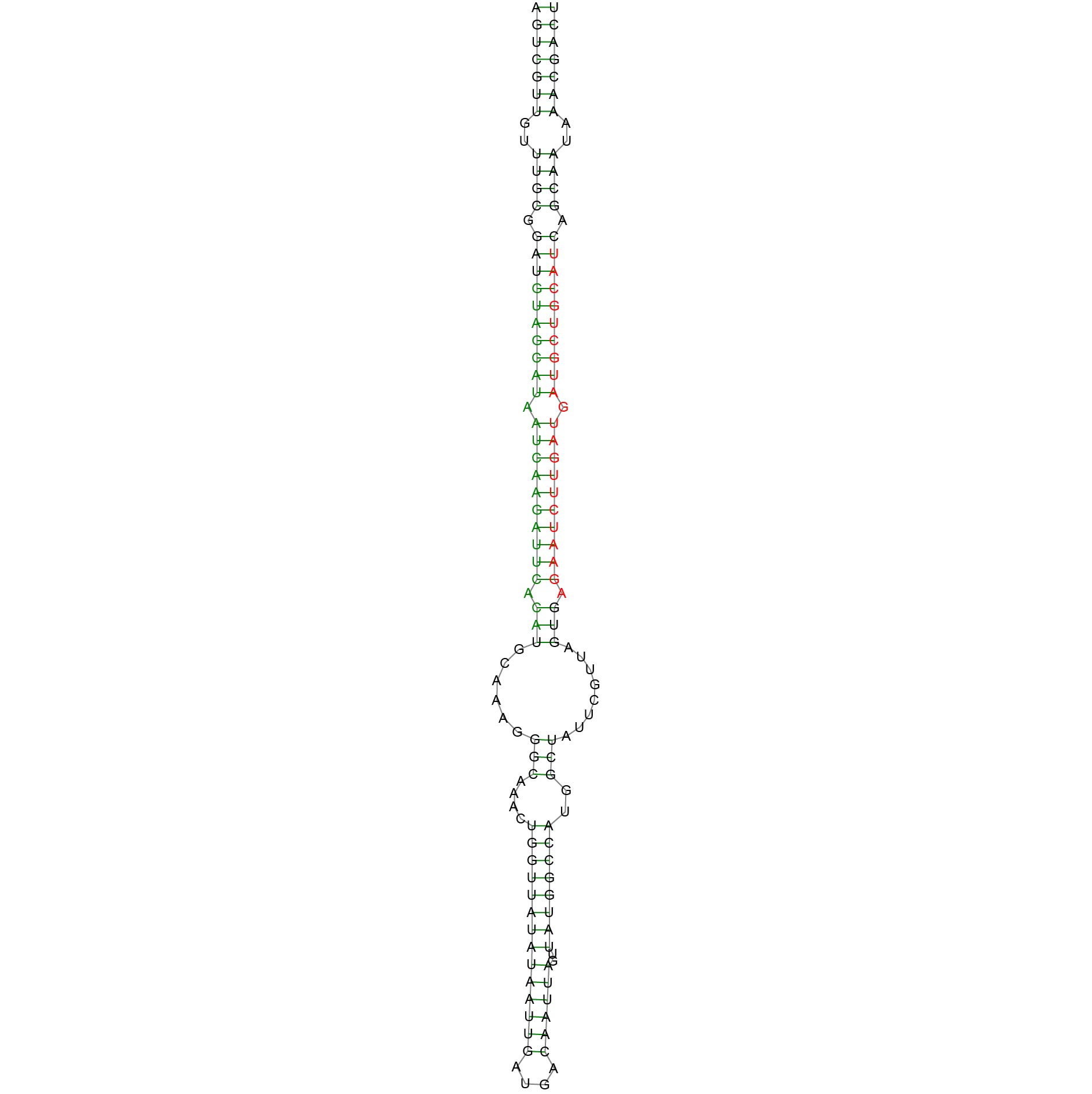

Supplement: Dataset S1 — Full list of hairpin structures in conserved miRNAs. (ZIP) [file pone.0064238.s001.zip › can-miR172d.jpg]

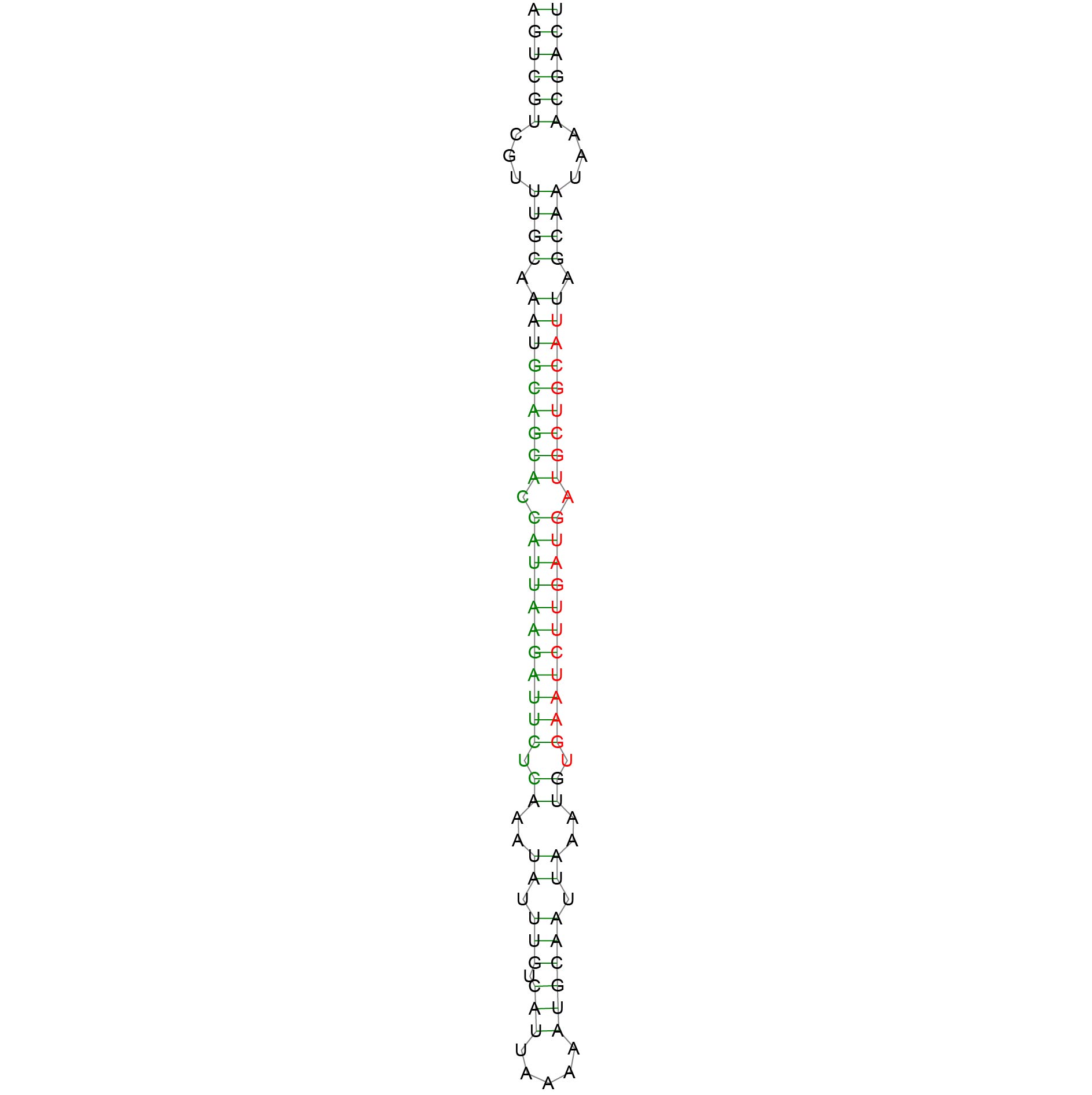

Supplement: Dataset S1 — Full list of hairpin structures in conserved miRNAs. (ZIP) [file pone.0064238.s001.zip › can-miR172e.jpg]

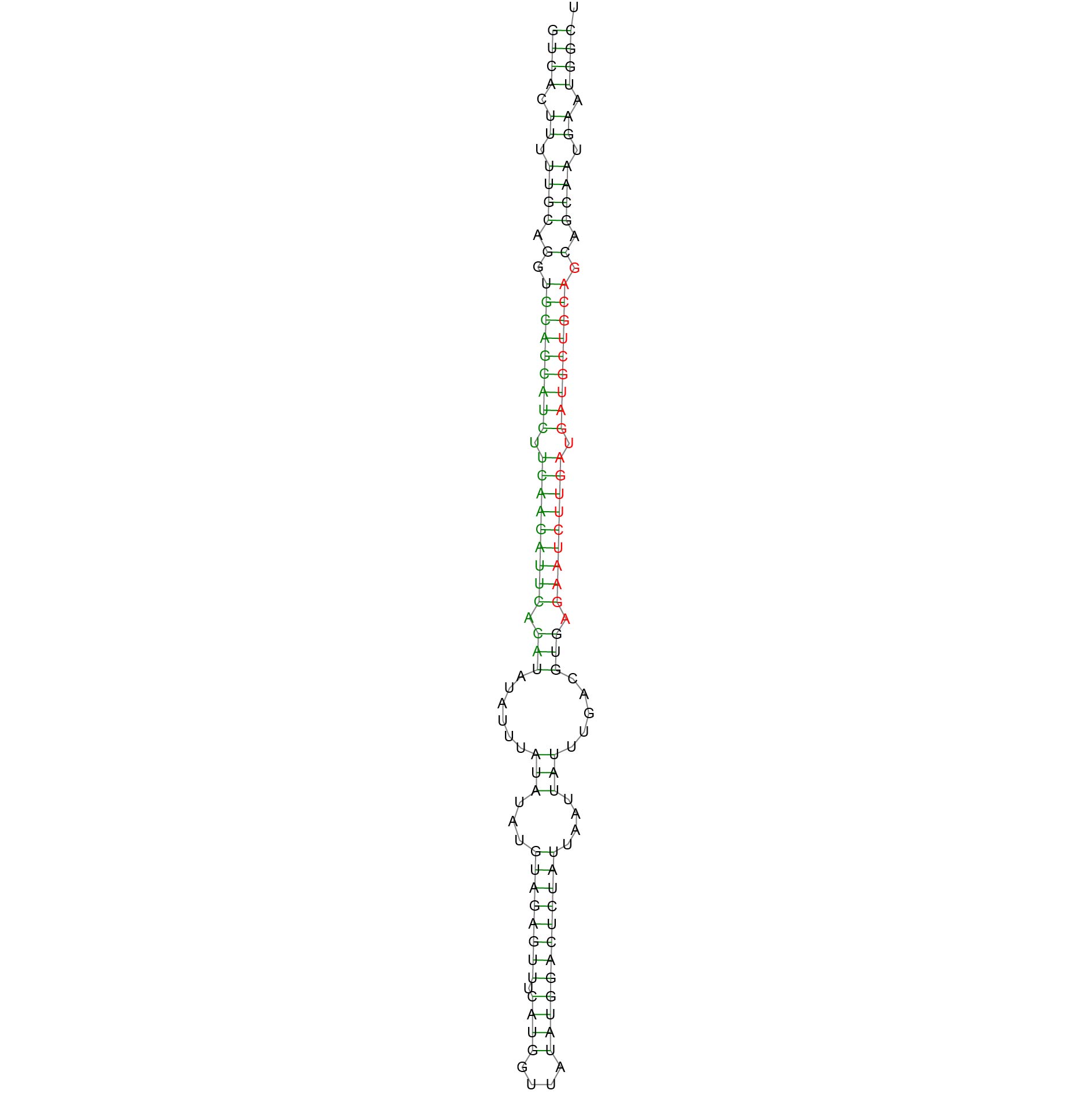

Supplement: Dataset S1 — Full list of hairpin structures in conserved miRNAs. (ZIP) [file pone.0064238.s001.zip › can-miR172f.jpg]

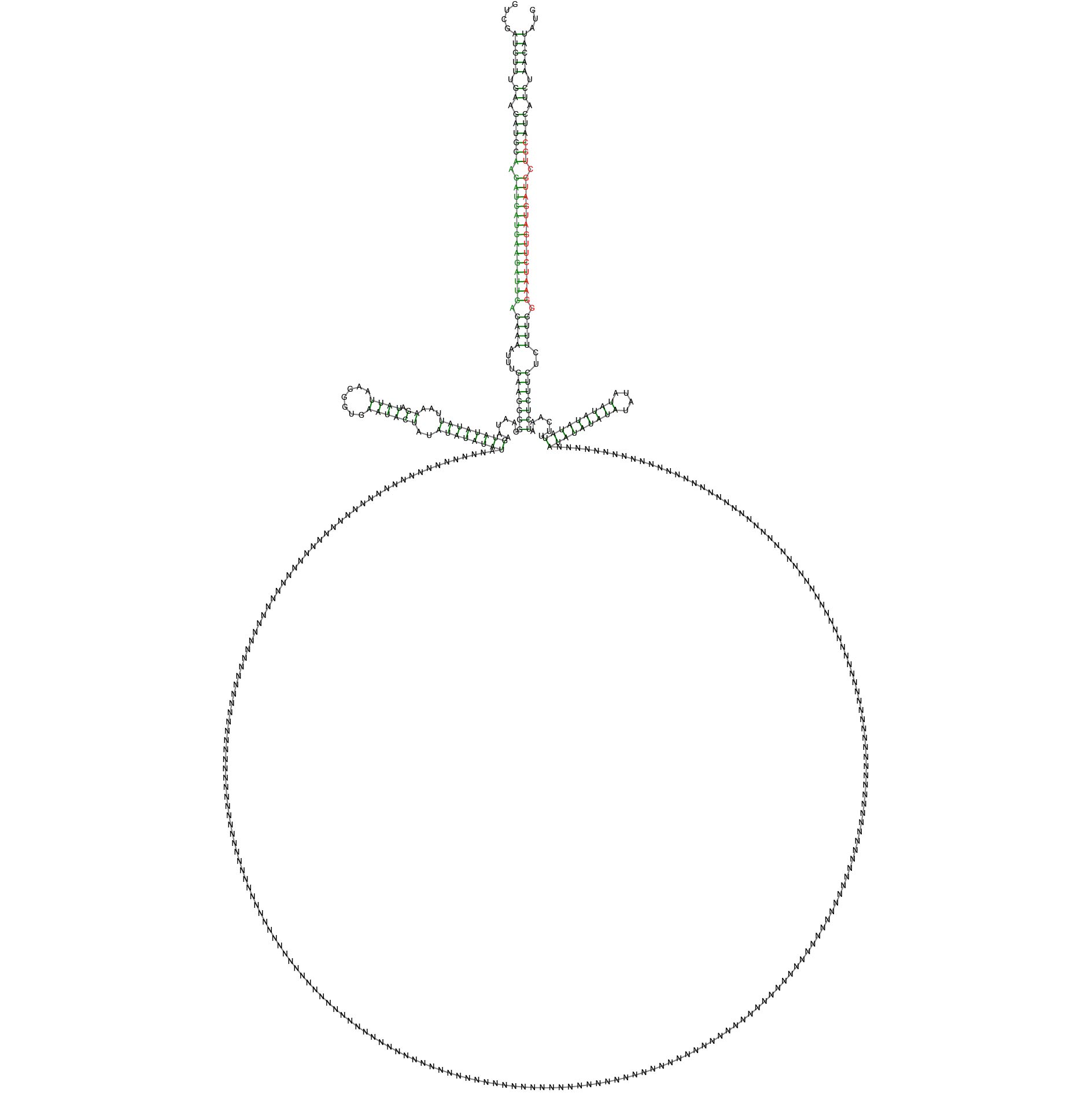

Supplement: Dataset S1 — Full list of hairpin structures in conserved miRNAs. (ZIP) [file pone.0064238.s001.zip › can-miR172g.jpg]

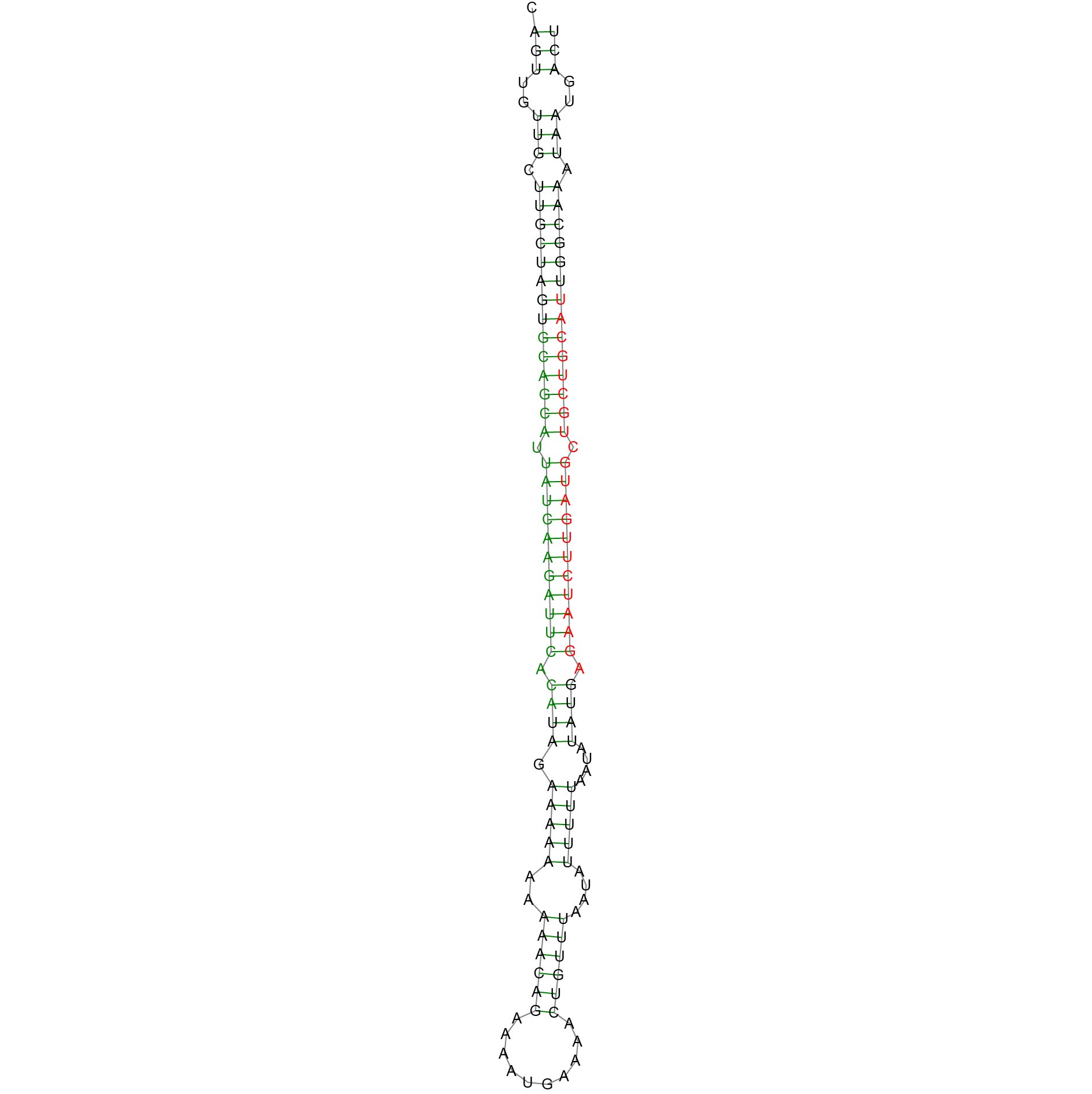

Supplement: Dataset S1 — Full list of hairpin structures in conserved miRNAs. (ZIP) [file pone.0064238.s001.zip › can-miR172h.jpg]

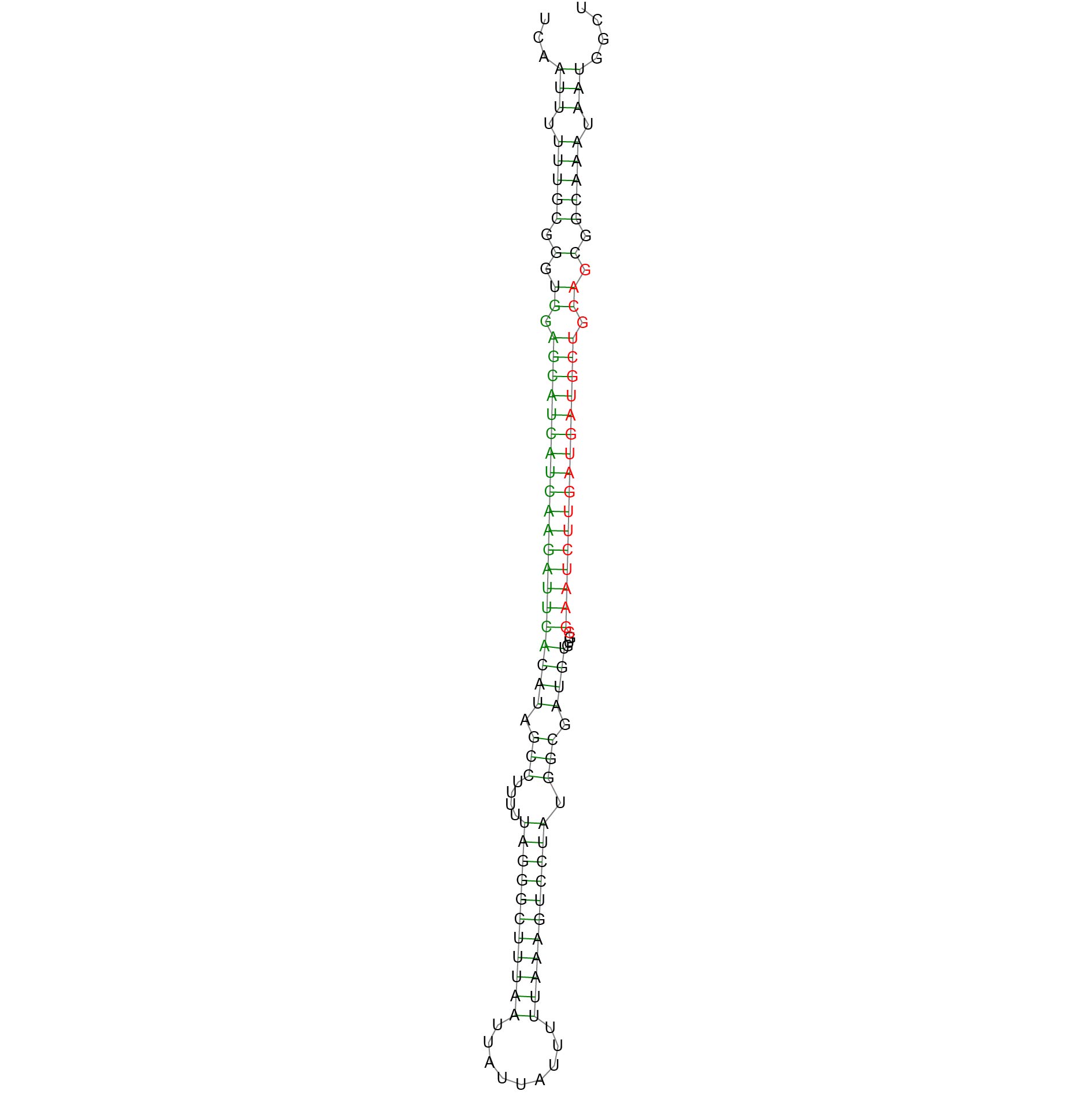

Supplement: Dataset S1 — Full list of hairpin structures in conserved miRNAs. (ZIP) [file pone.0064238.s001.zip › can-miR172i.jpg]

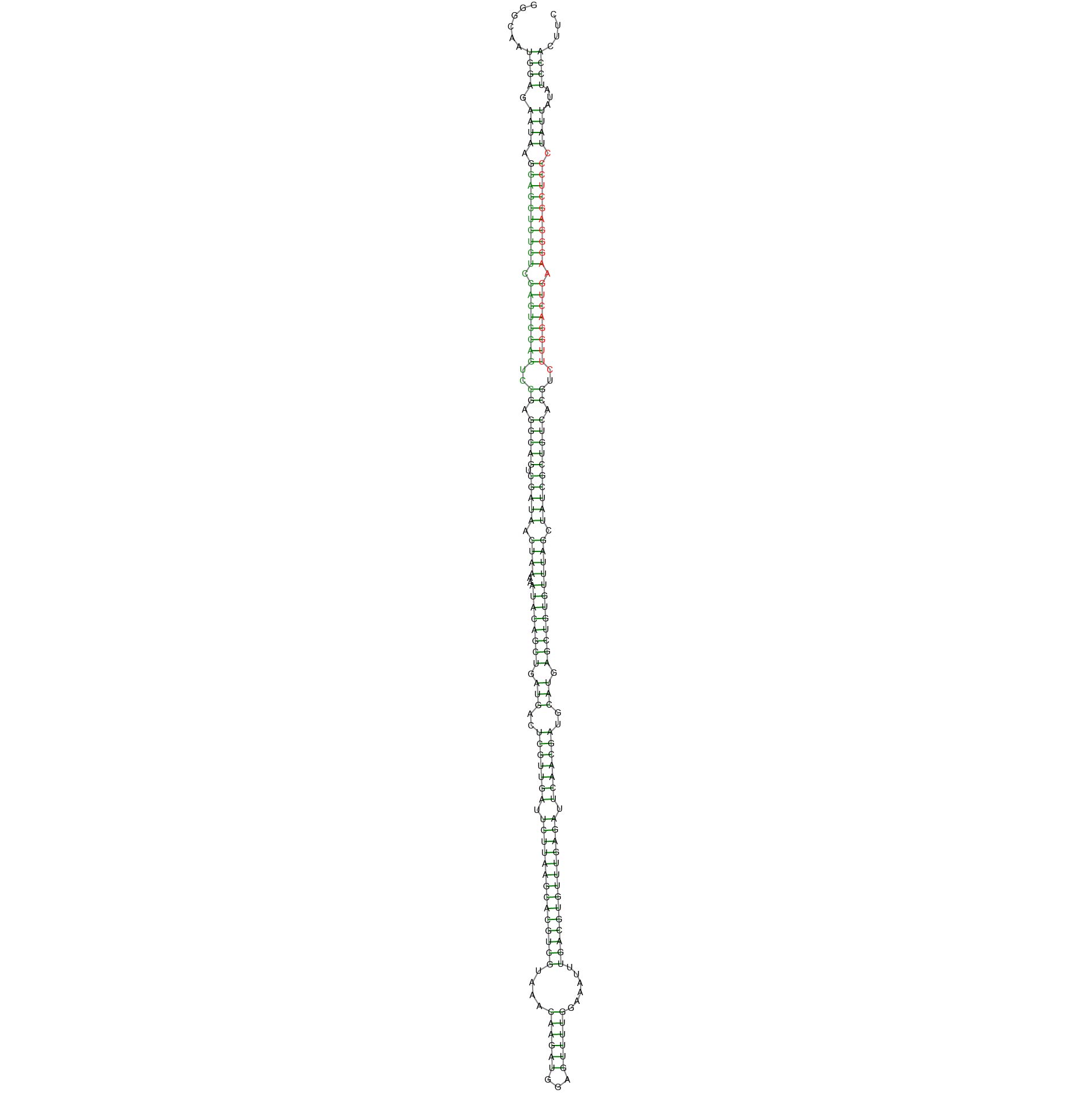

Supplement: Dataset S1 — Full list of hairpin structures in conserved miRNAs. (ZIP) [file pone.0064238.s001.zip › can-miR319a.jpg]

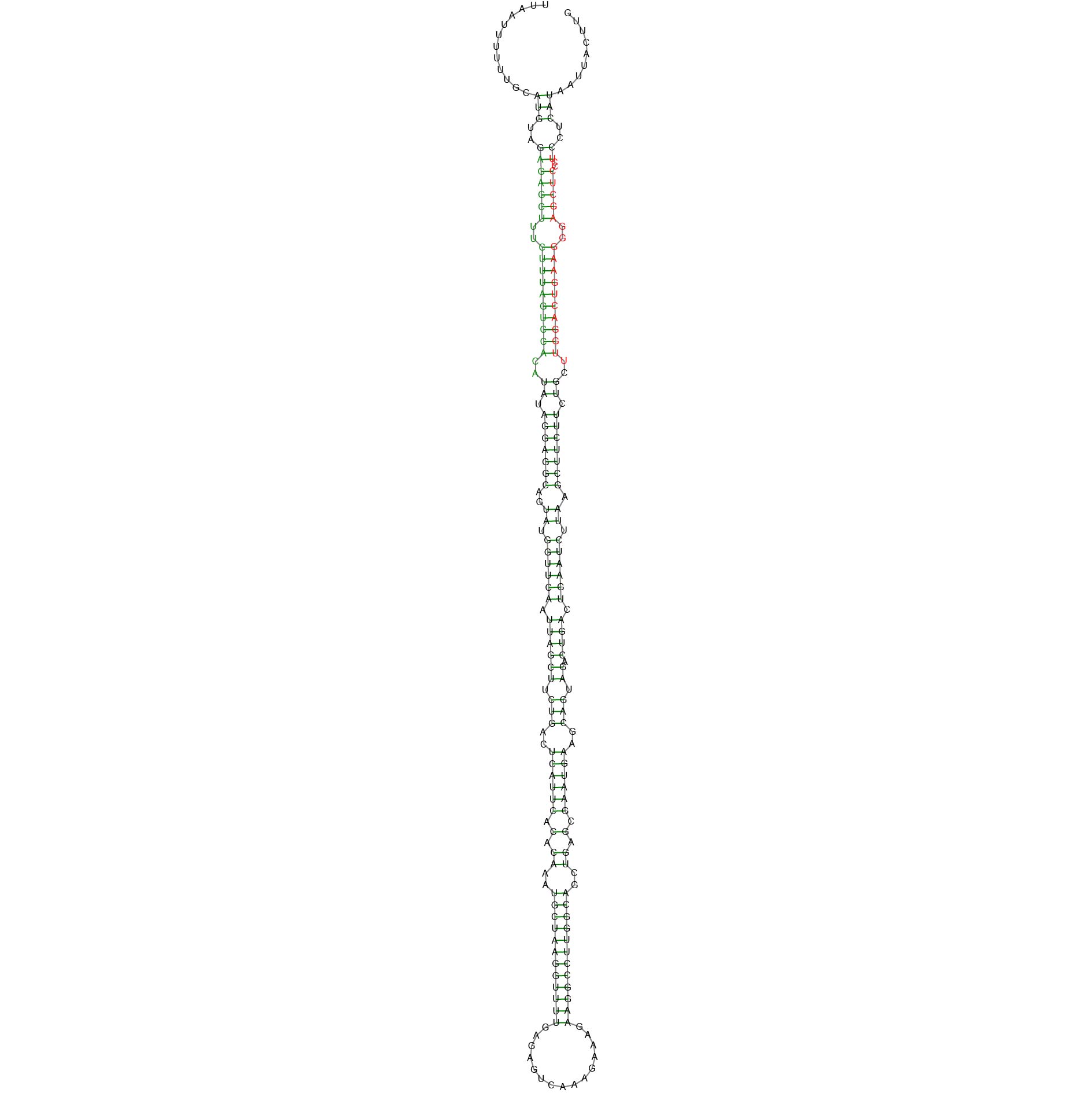

Supplement: Dataset S1 — Full list of hairpin structures in conserved miRNAs. (ZIP) [file pone.0064238.s001.zip › can-miR319b.jpg]

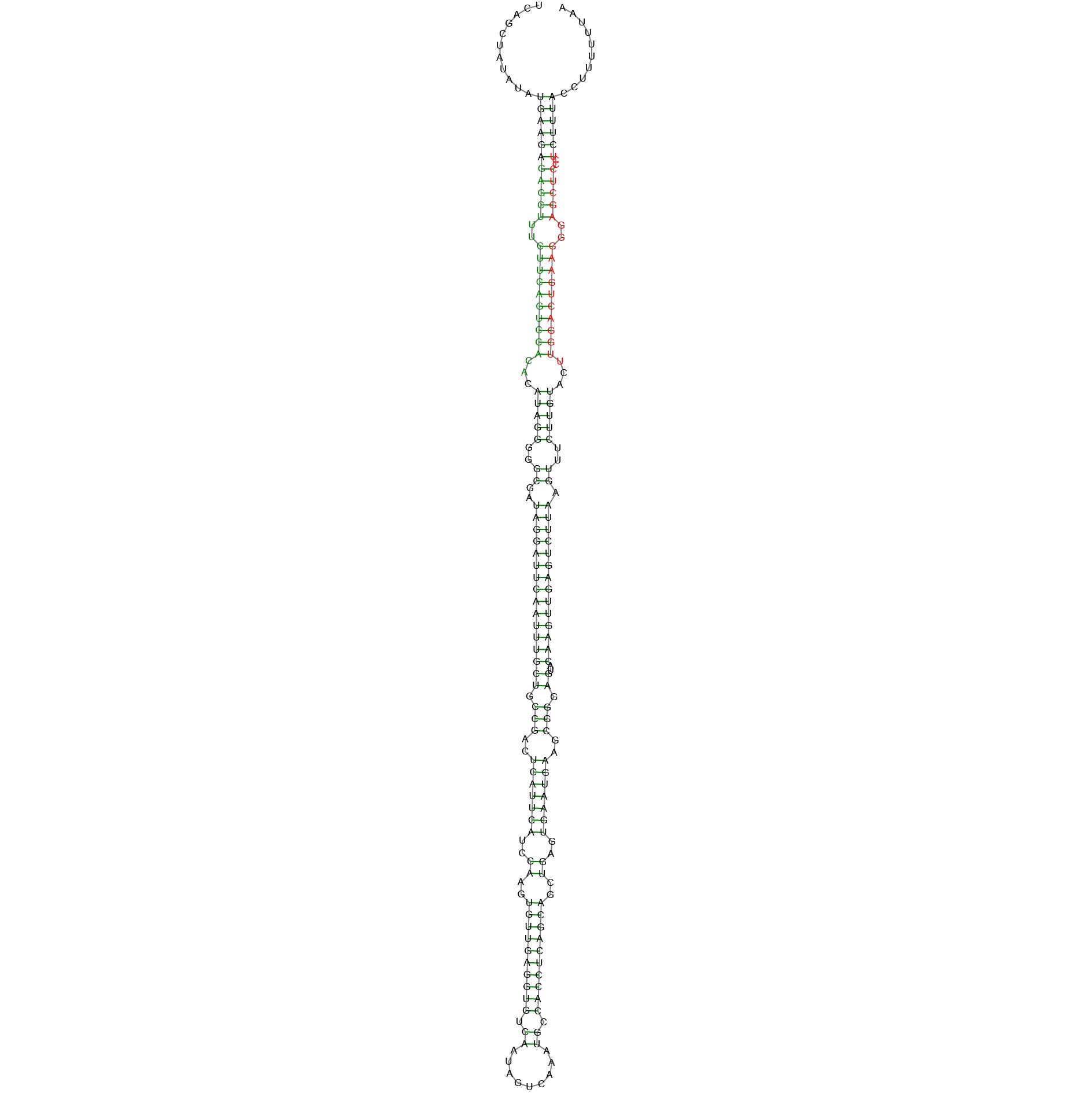

Supplement: Dataset S1 — Full list of hairpin structures in conserved miRNAs. (ZIP) [file pone.0064238.s001.zip › can-miR319c.jpg]

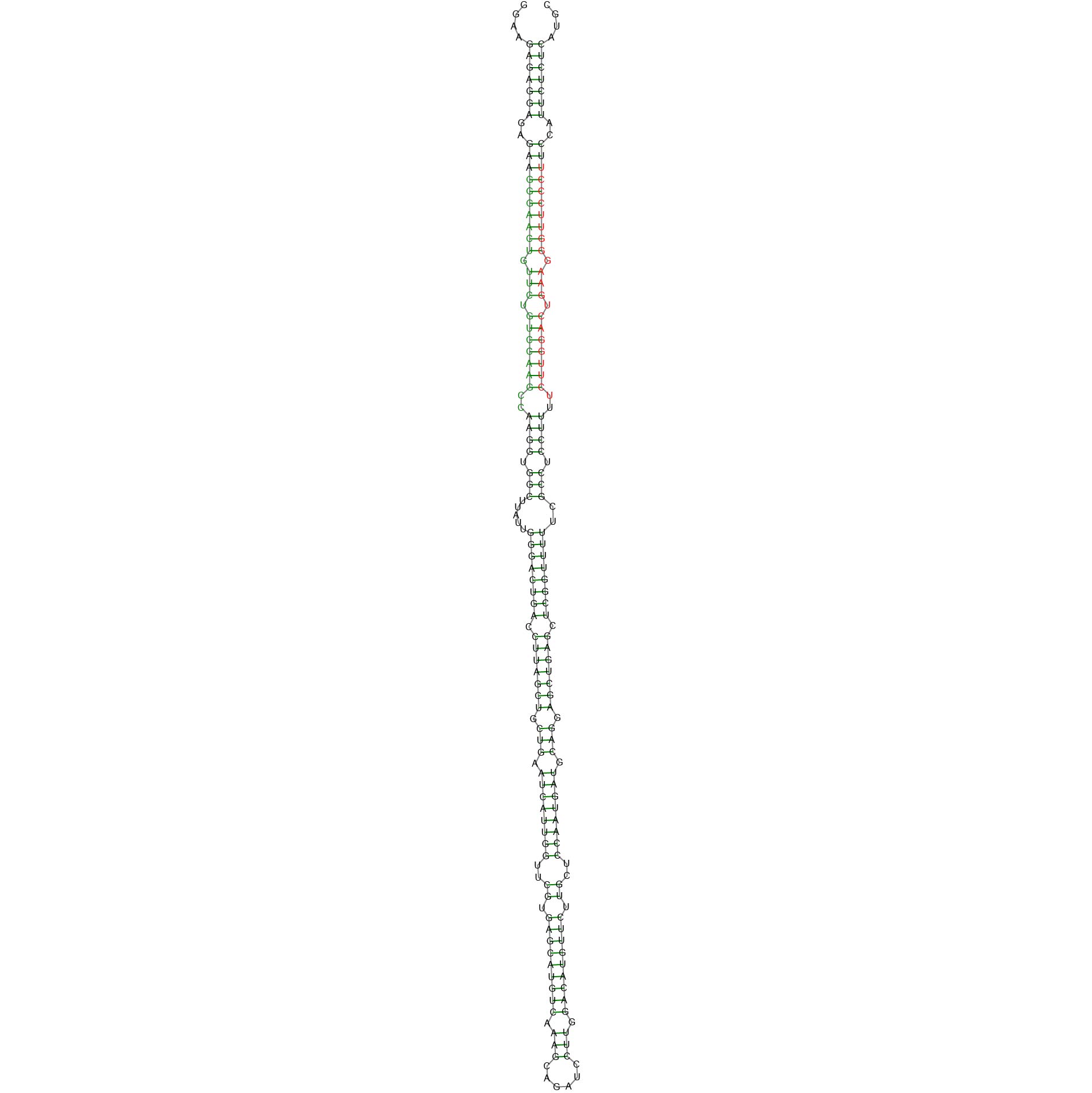

Supplement: Dataset S1 — Full list of hairpin structures in conserved miRNAs. (ZIP) [file pone.0064238.s001.zip › can-miR319d.jpg]

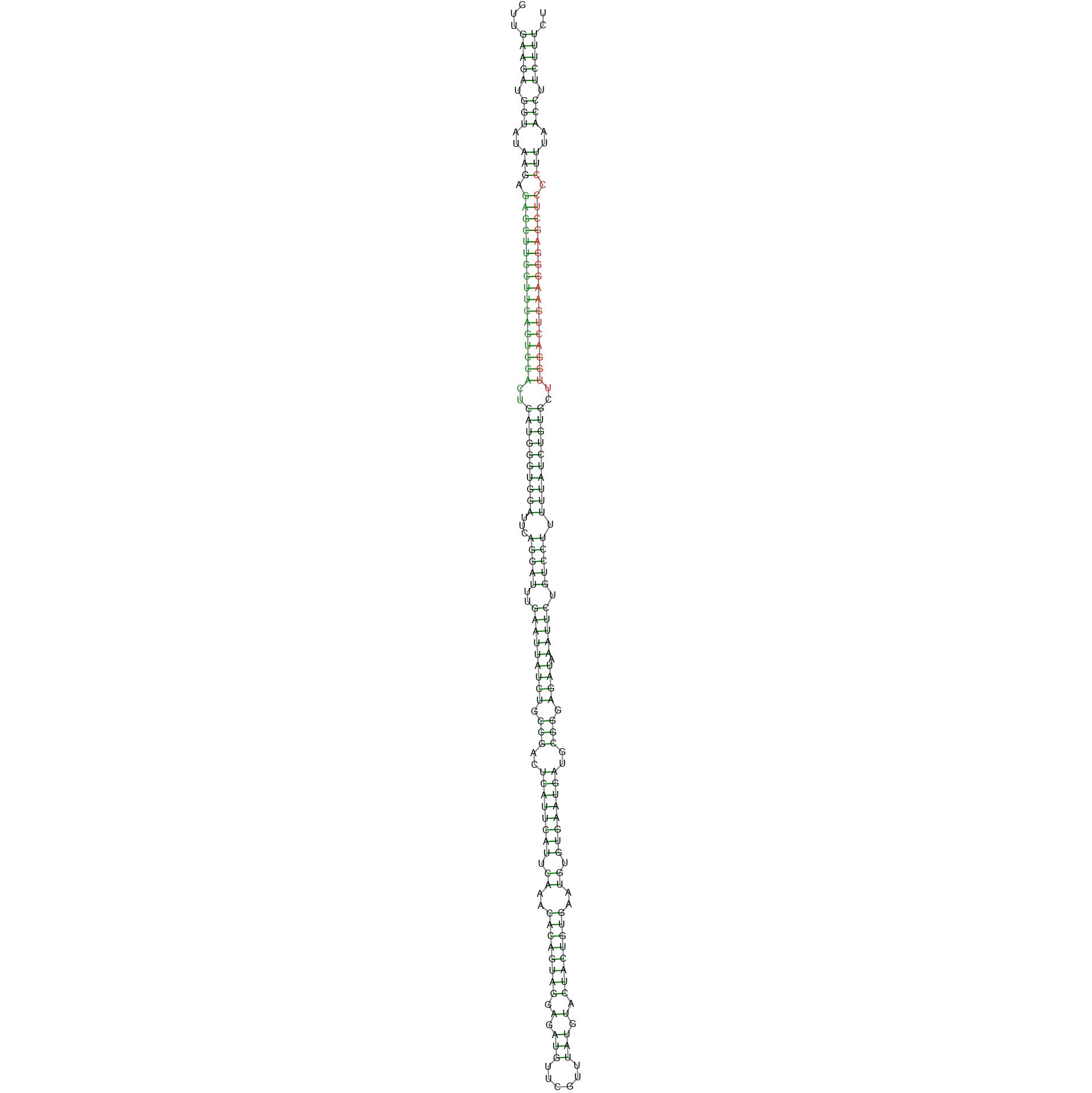

Supplement: Dataset S1 — Full list of hairpin structures in conserved miRNAs. (ZIP) [file pone.0064238.s001.zip › can-miR319e.jpg]

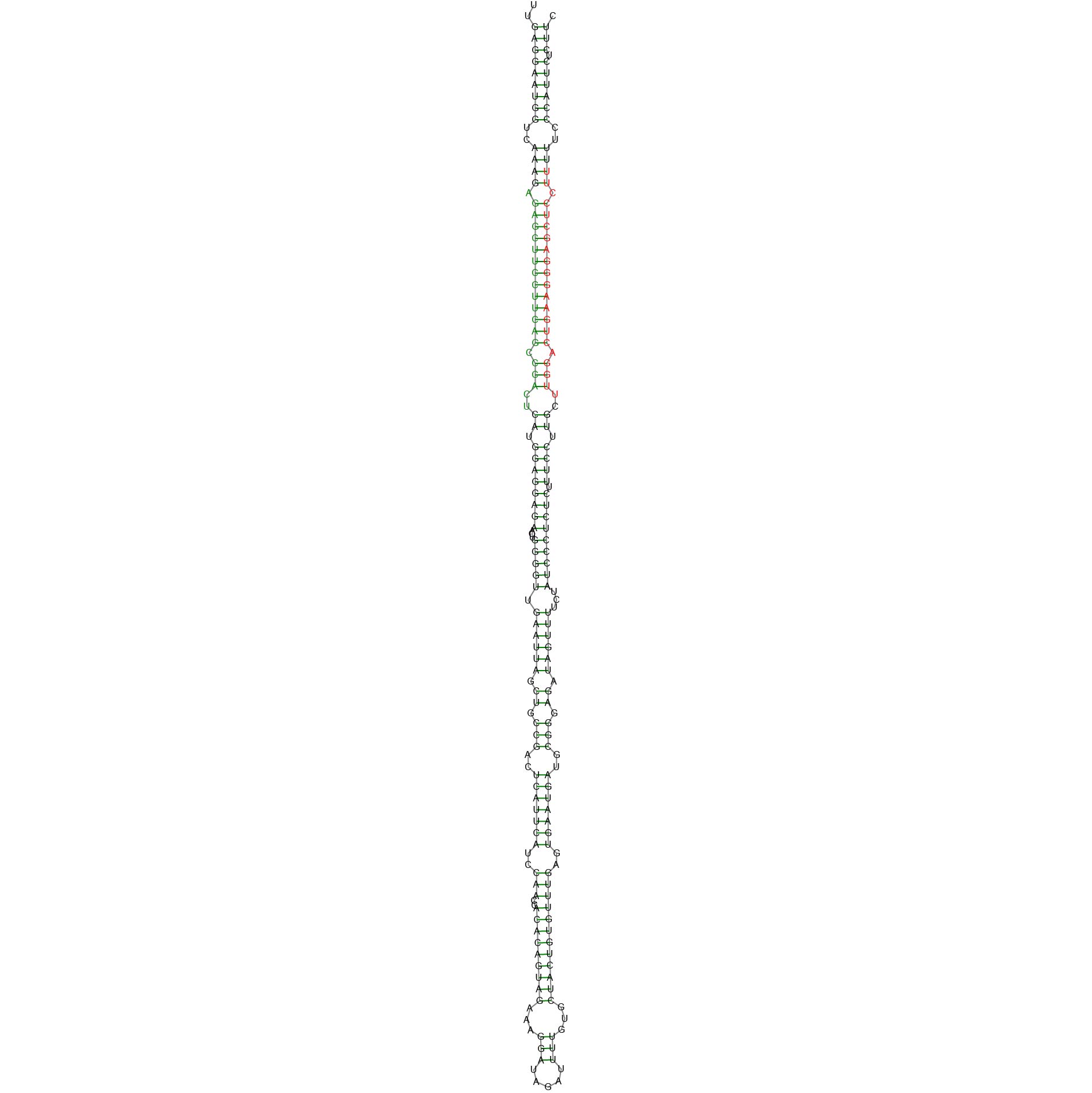

Supplement: Dataset S1 — Full list of hairpin structures in conserved miRNAs. (ZIP) [file pone.0064238.s001.zip › can-miR319f.jpg]

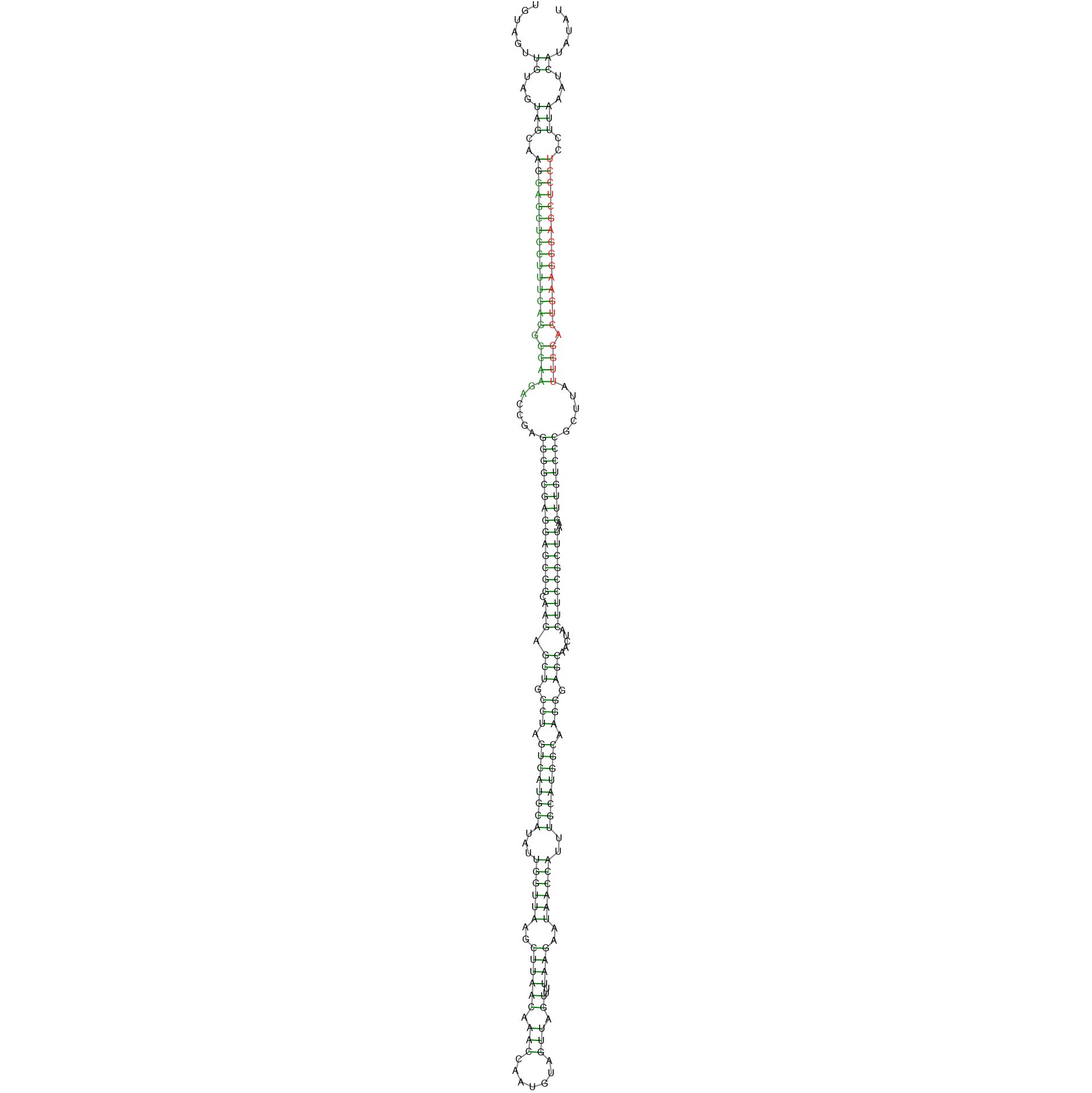

Supplement: Dataset S1 — Full list of hairpin structures in conserved miRNAs. (ZIP) [file pone.0064238.s001.zip › can-miR319g.jpg]

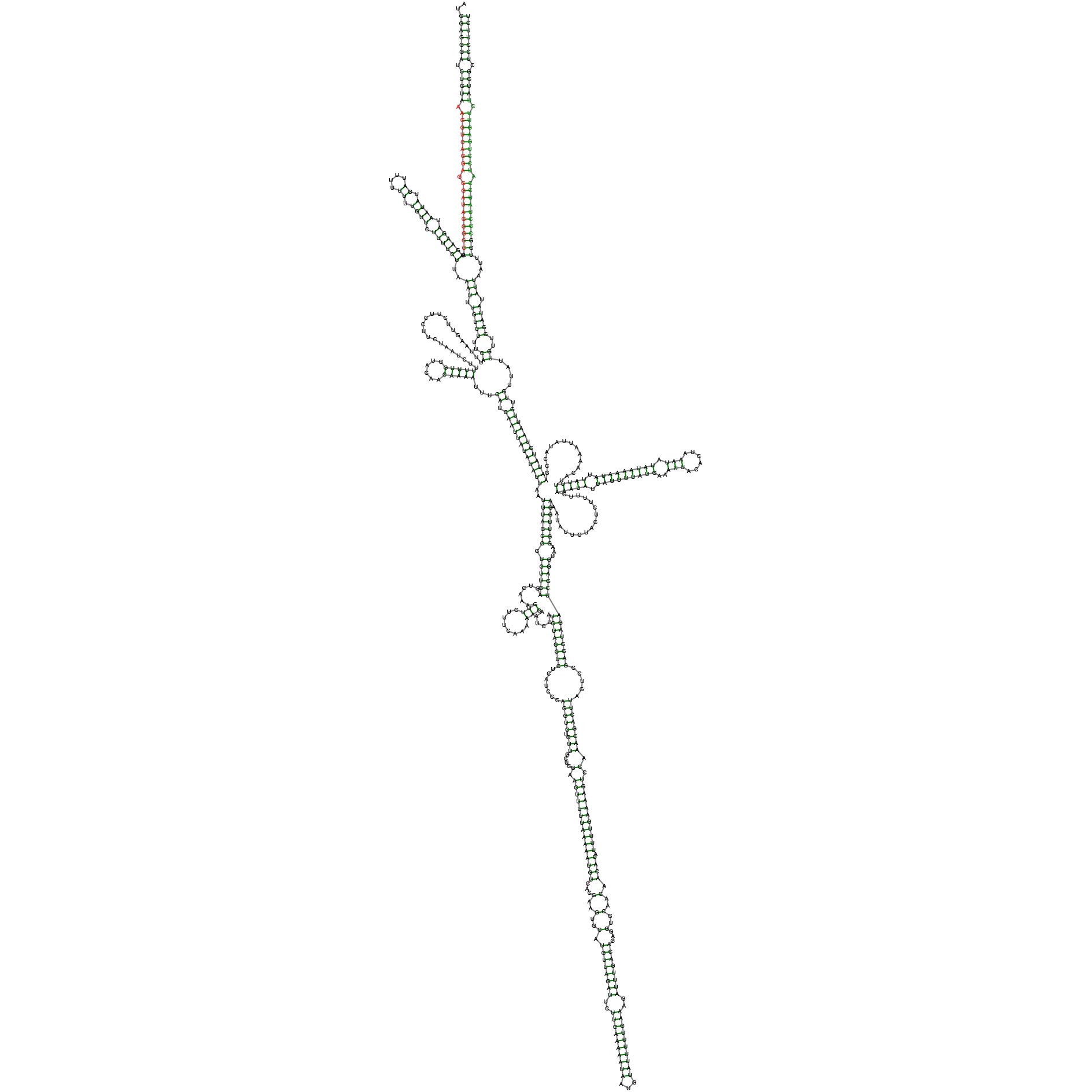

Supplement: Dataset S1 — Full list of hairpin structures in conserved miRNAs. (ZIP) [file pone.0064238.s001.zip › can-miR390a.jpg]

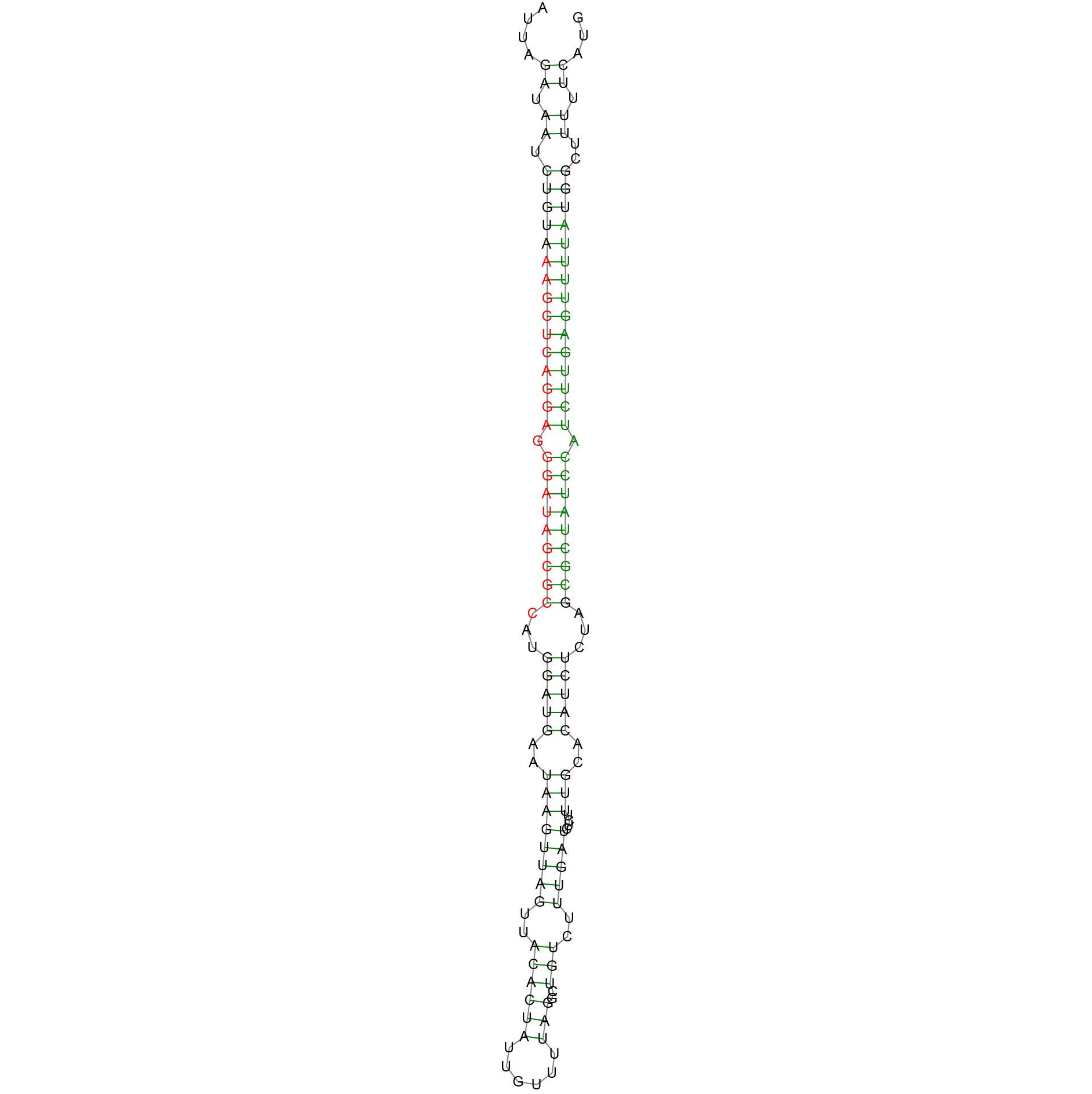

Supplement: Dataset S1 — Full list of hairpin structures in conserved miRNAs. (ZIP) [file pone.0064238.s001.zip › can-miR390b.jpg]

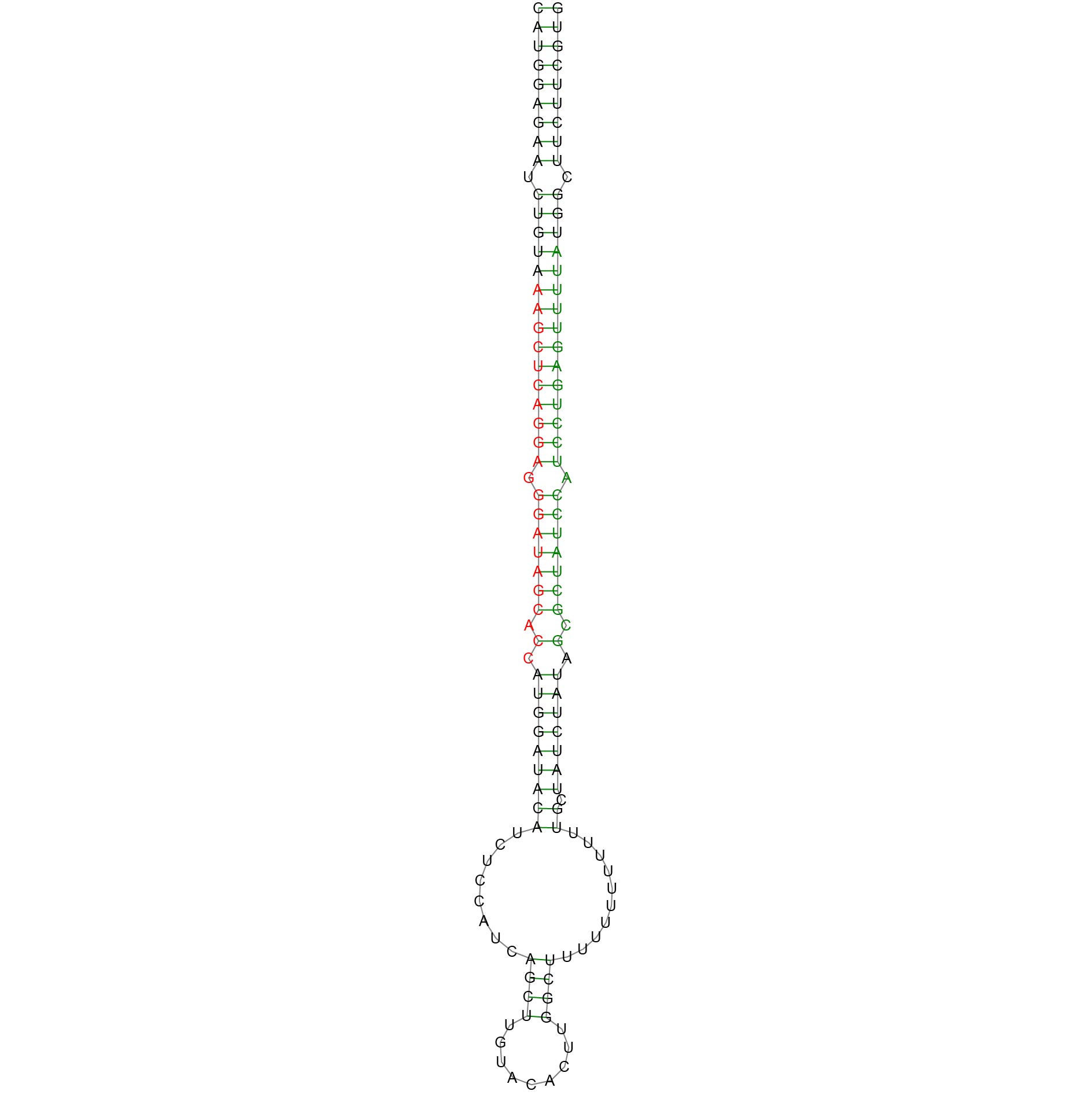

Supplement: Dataset S1 — Full list of hairpin structures in conserved miRNAs. (ZIP) [file pone.0064238.s001.zip › can-miR390c.jpg]

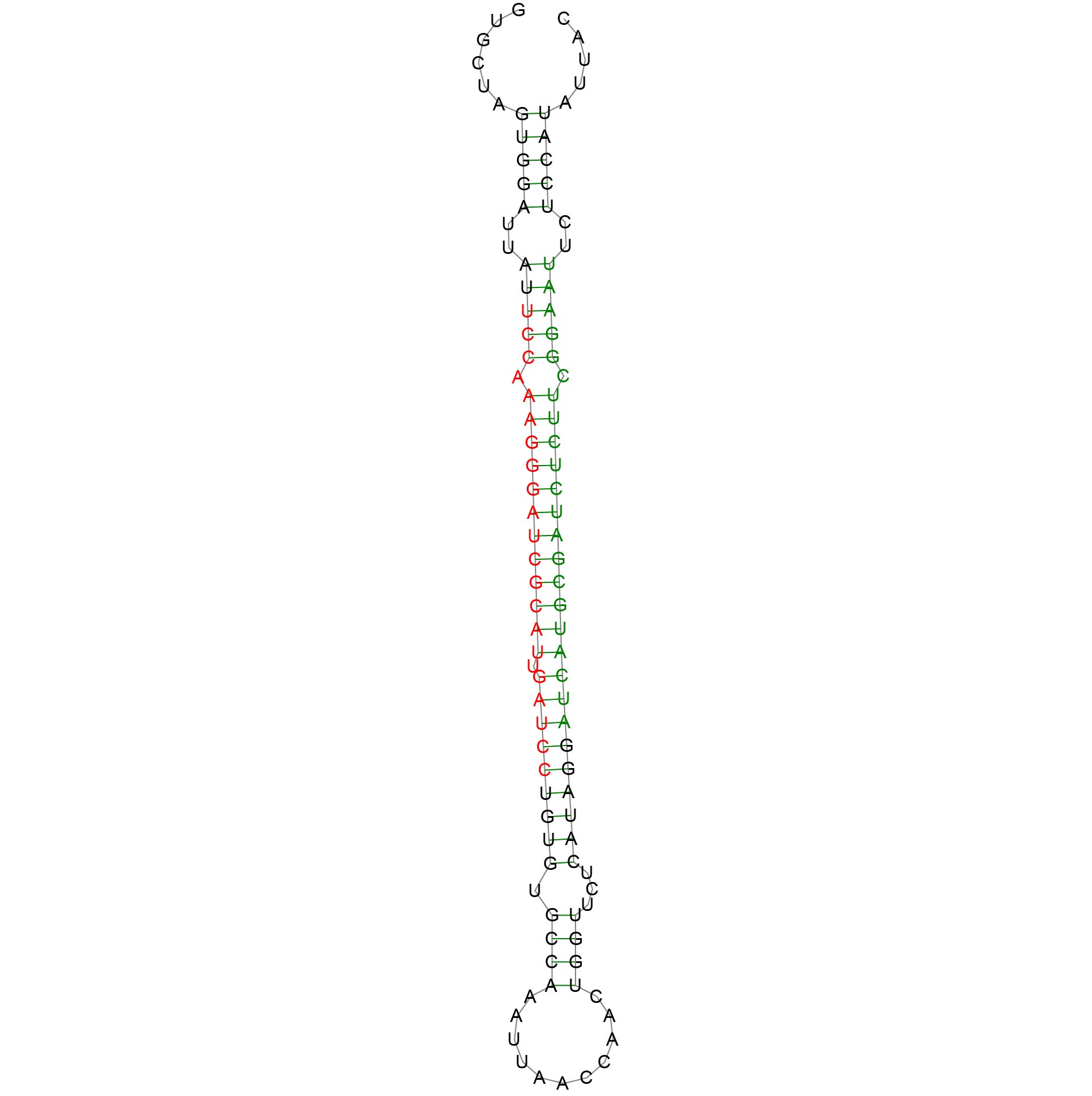

Supplement: Dataset S1 — Full list of hairpin structures in conserved miRNAs. (ZIP) [file pone.0064238.s001.zip › can-miR393a.jpg]

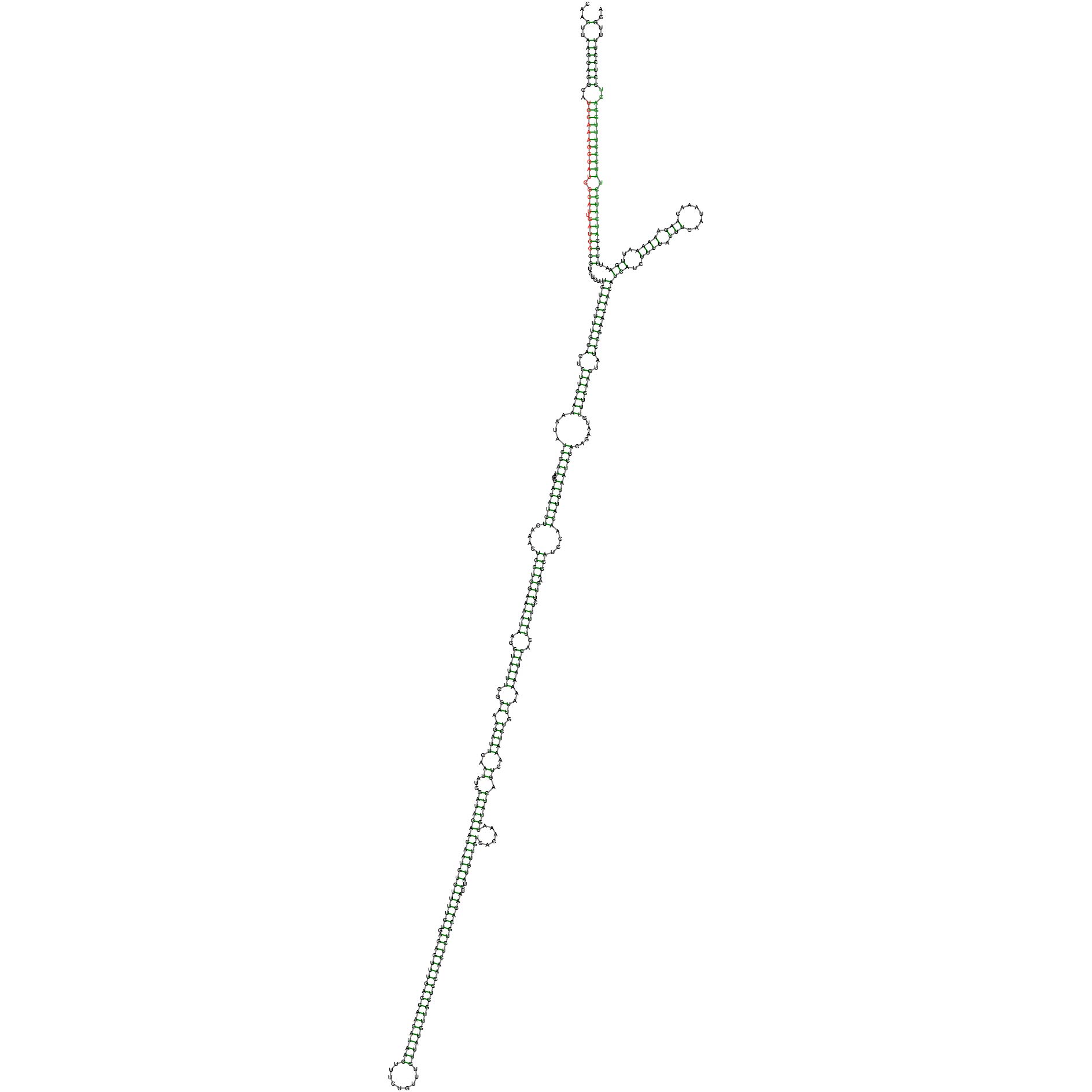

Supplement: Dataset S1 — Full list of hairpin structures in conserved miRNAs. (ZIP) [file pone.0064238.s001.zip › can-miR393b.jpg]

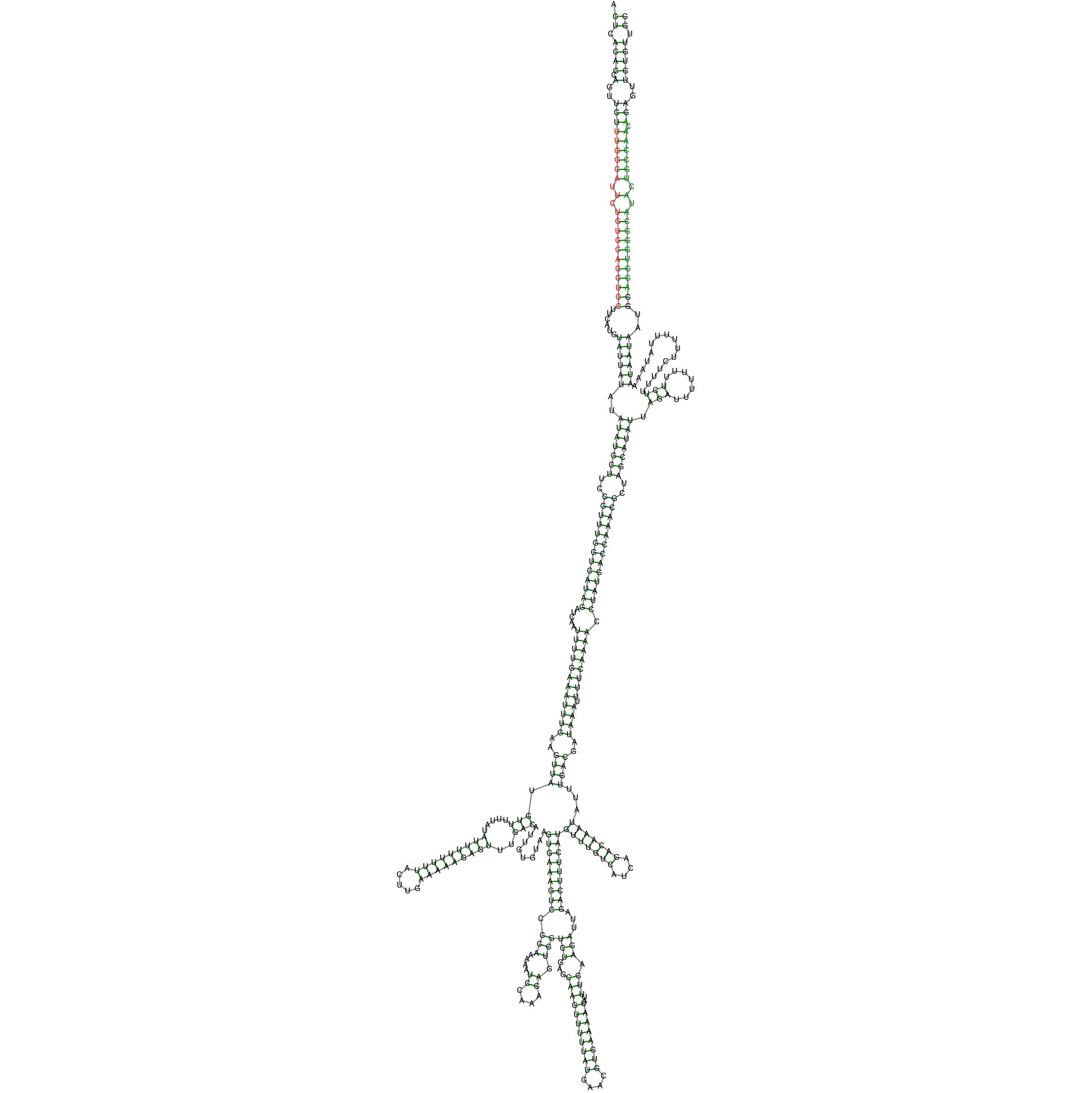

Supplement: Dataset S1 — Full list of hairpin structures in conserved miRNAs. (ZIP) [file pone.0064238.s001.zip › can-miR394a.jpg]

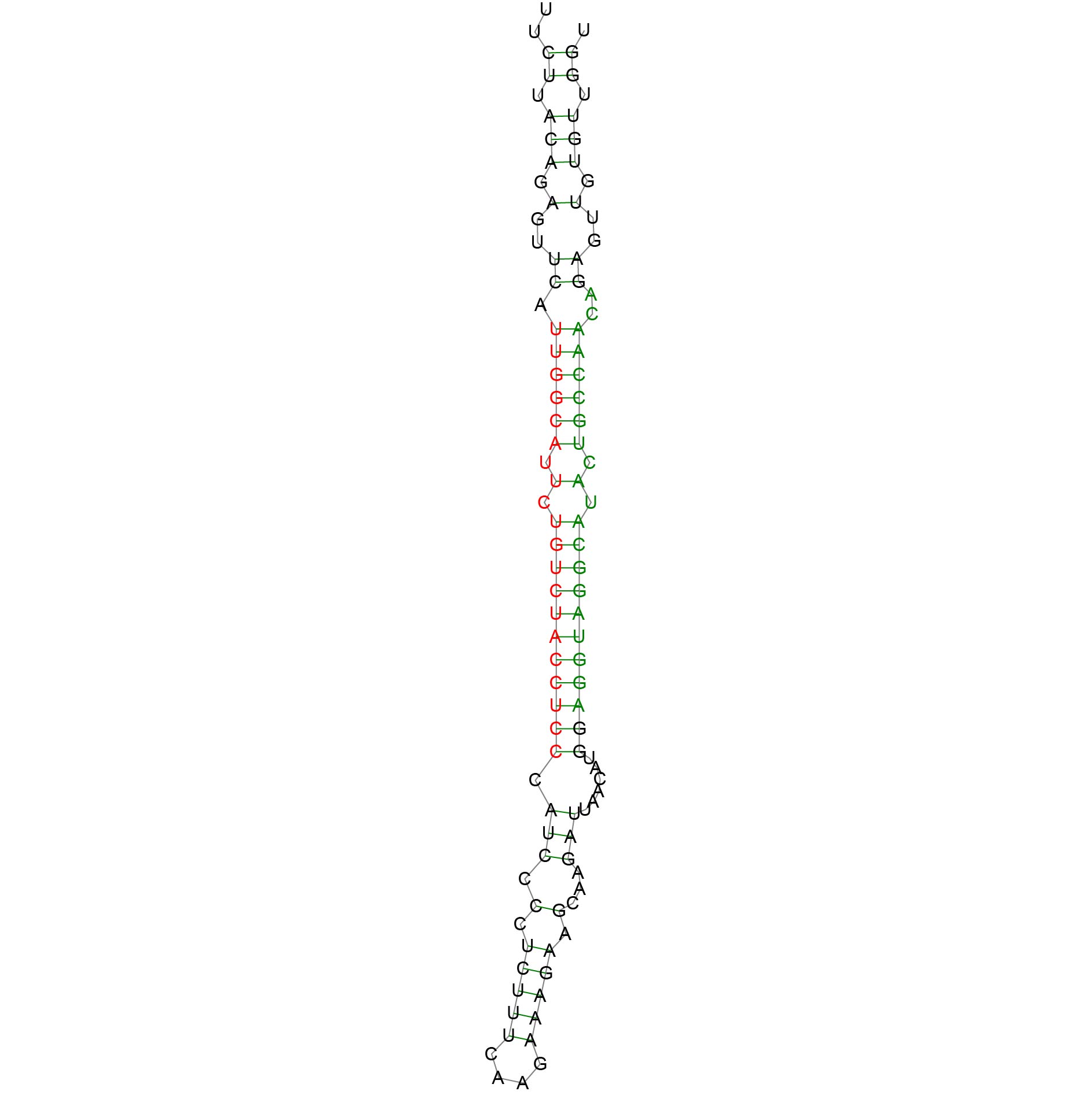

Supplement: Dataset S1 — Full list of hairpin structures in conserved miRNAs. (ZIP) [file pone.0064238.s001.zip › can-miR394b.jpg]

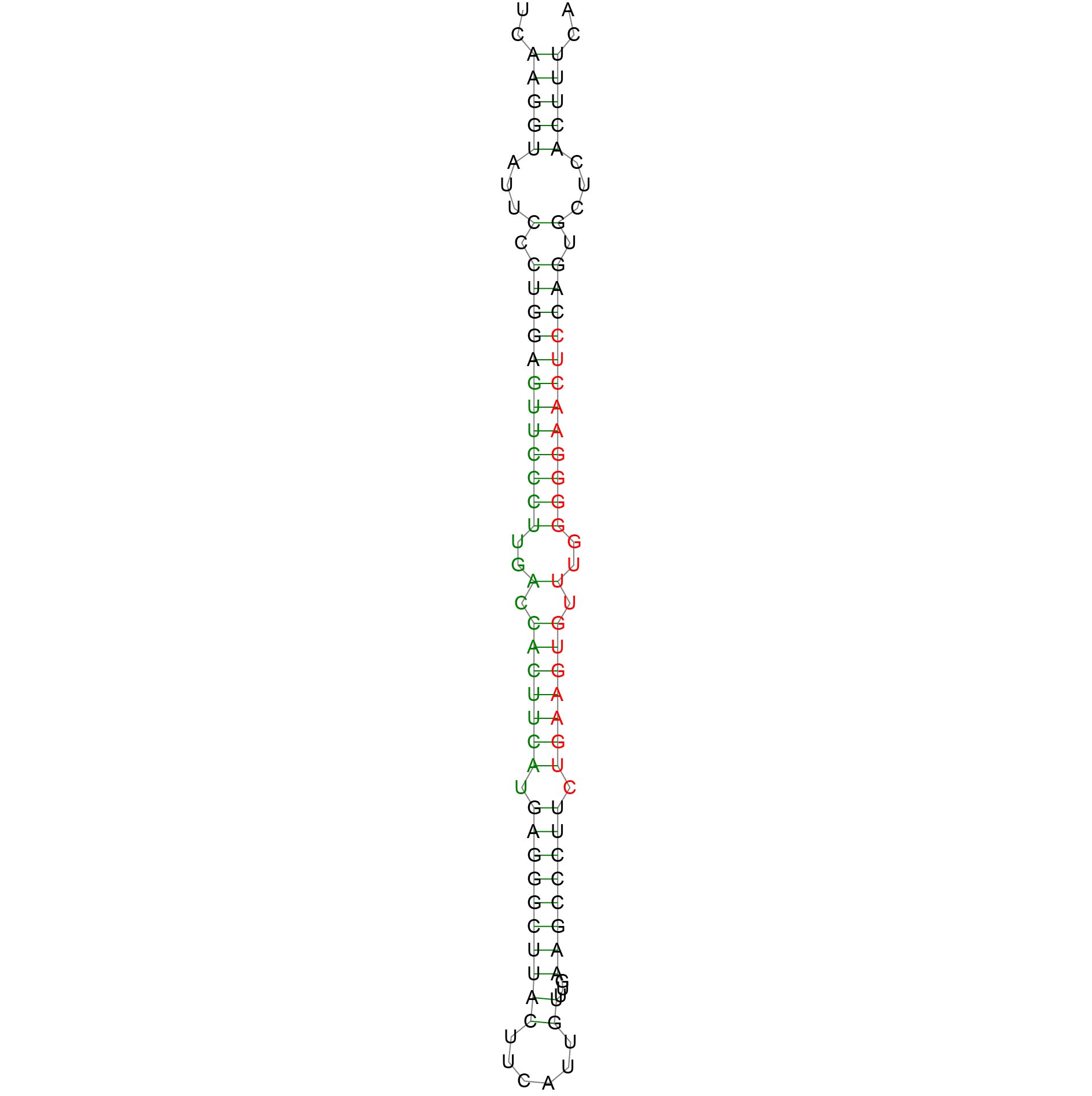

Supplement: Dataset S1 — Full list of hairpin structures in conserved miRNAs. (ZIP) [file pone.0064238.s001.zip › can-miR395a.jpg]

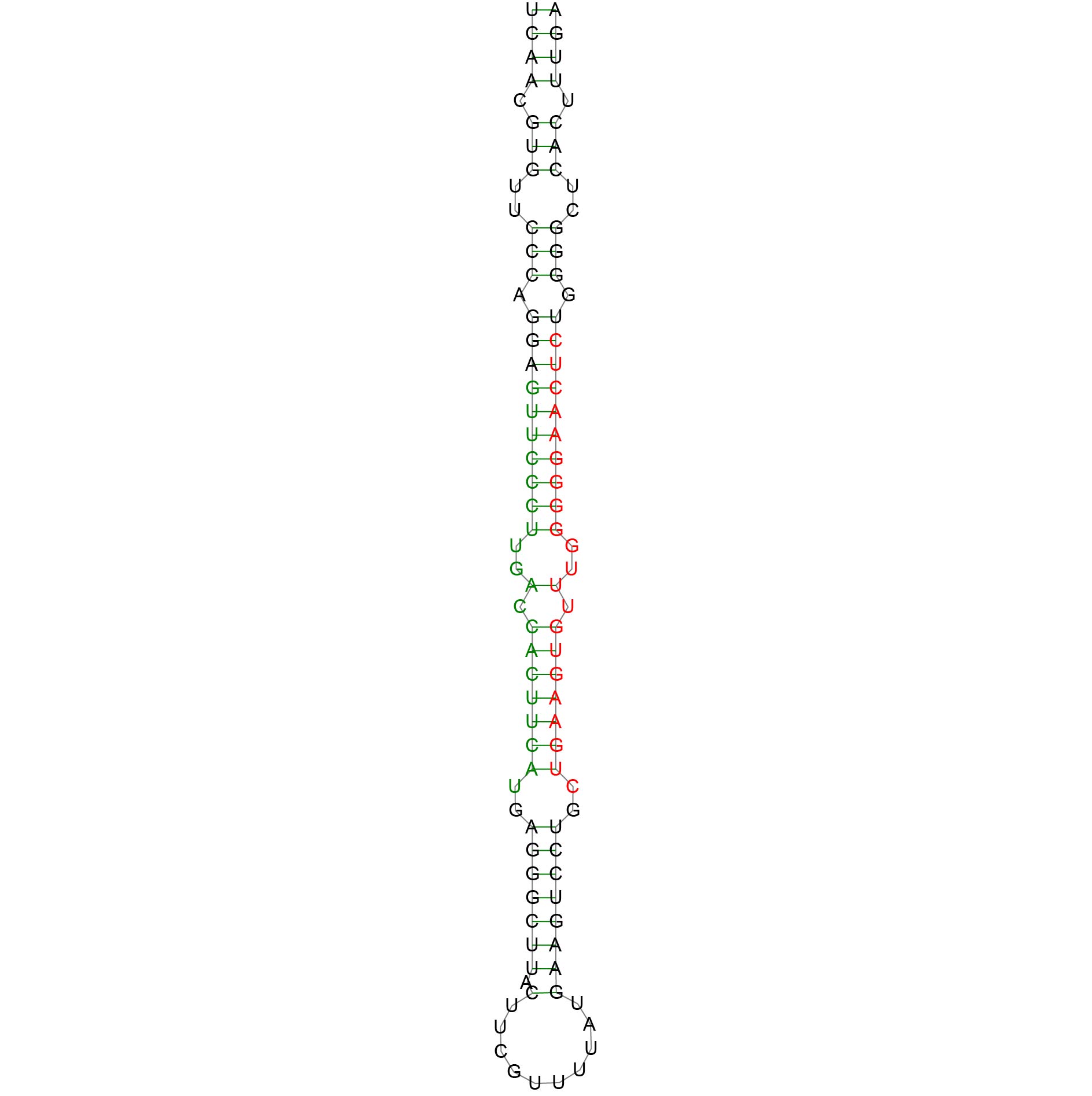

Supplement: Dataset S1 — Full list of hairpin structures in conserved miRNAs. (ZIP) [file pone.0064238.s001.zip › can-miR395b.jpg]

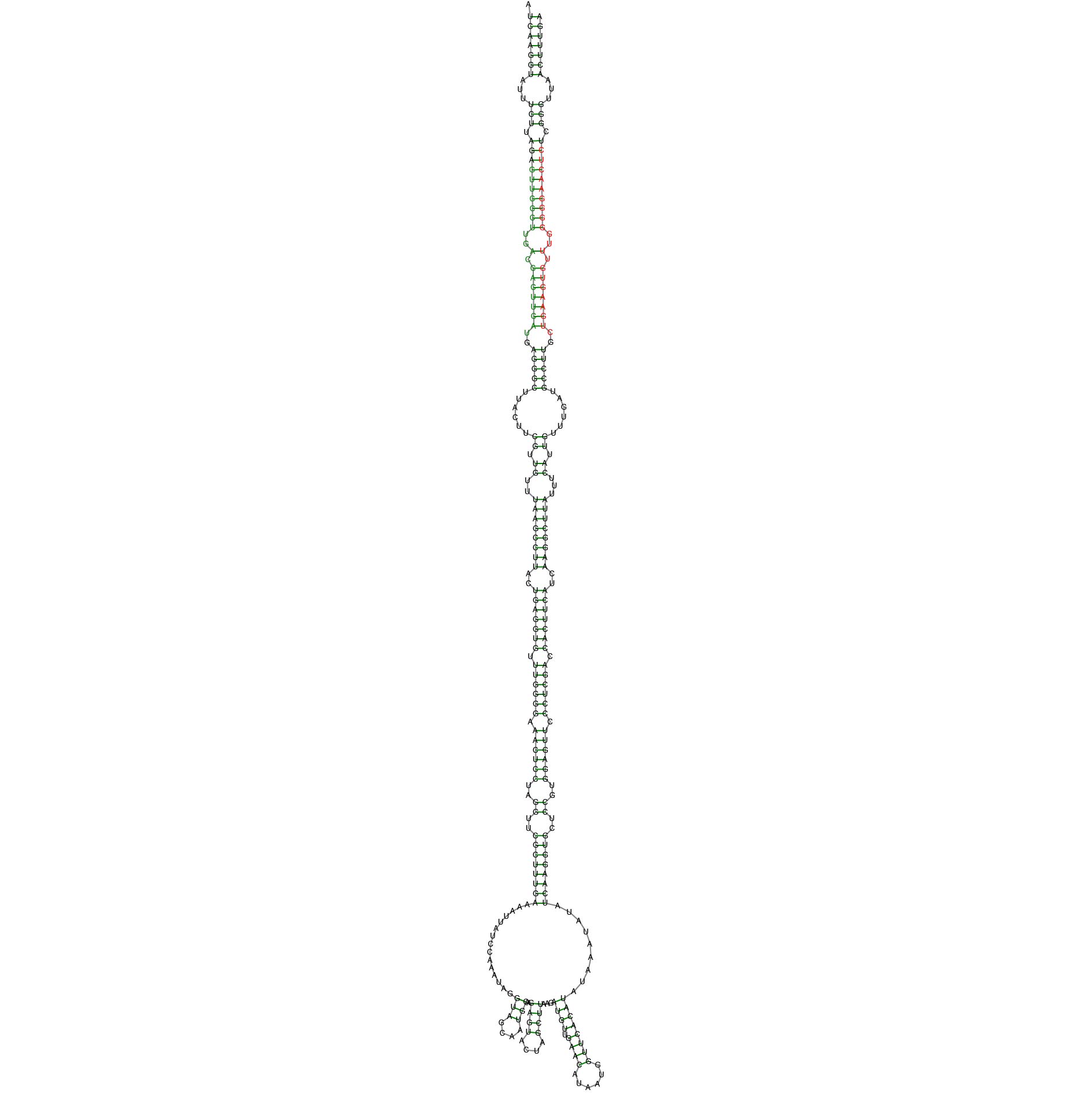

Supplement: Dataset S1 — Full list of hairpin structures in conserved miRNAs. (ZIP) [file pone.0064238.s001.zip › can-miR395c.jpg]

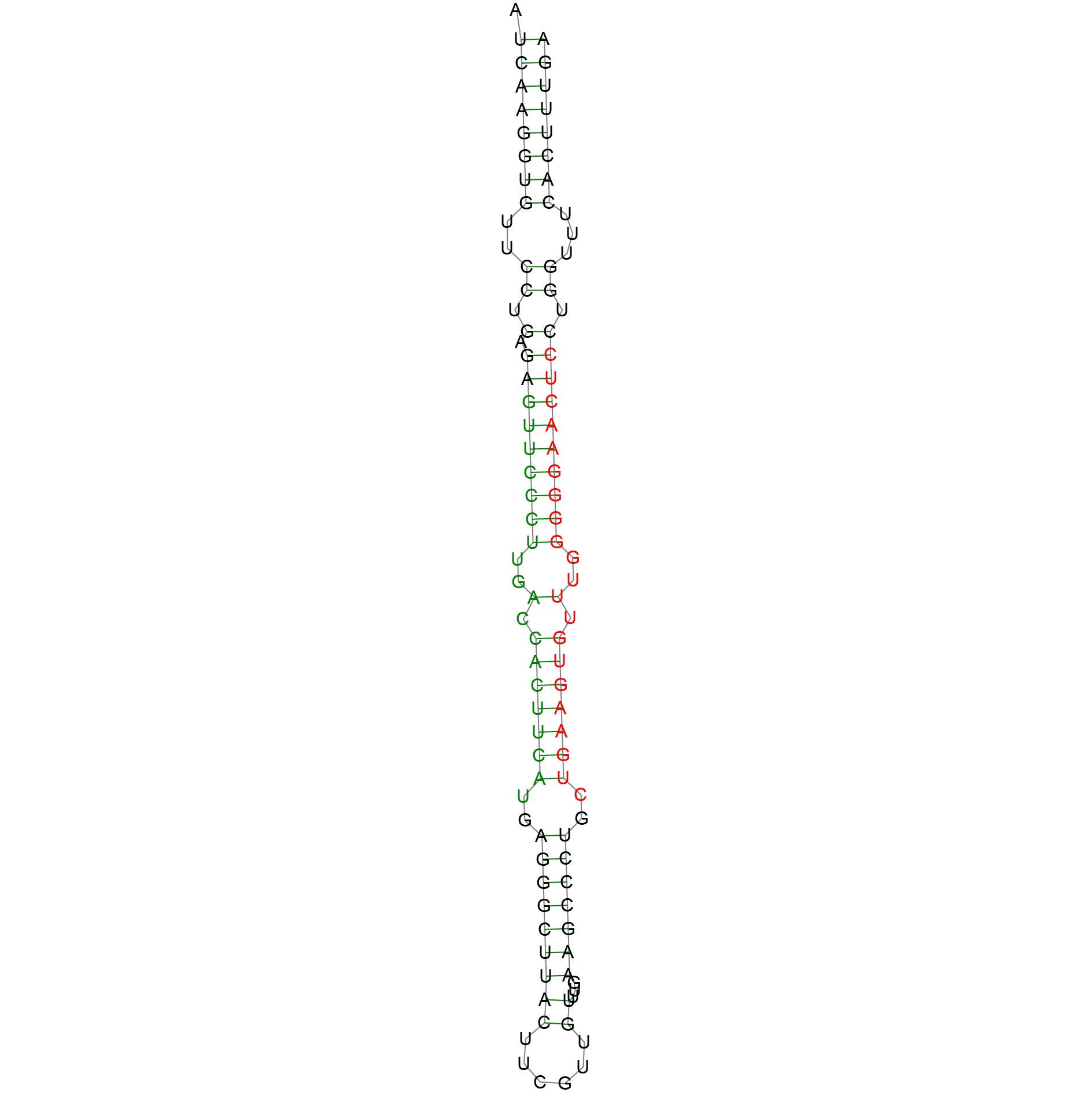

Supplement: Dataset S1 — Full list of hairpin structures in conserved miRNAs. (ZIP) [file pone.0064238.s001.zip › can-miR395d.jpg]

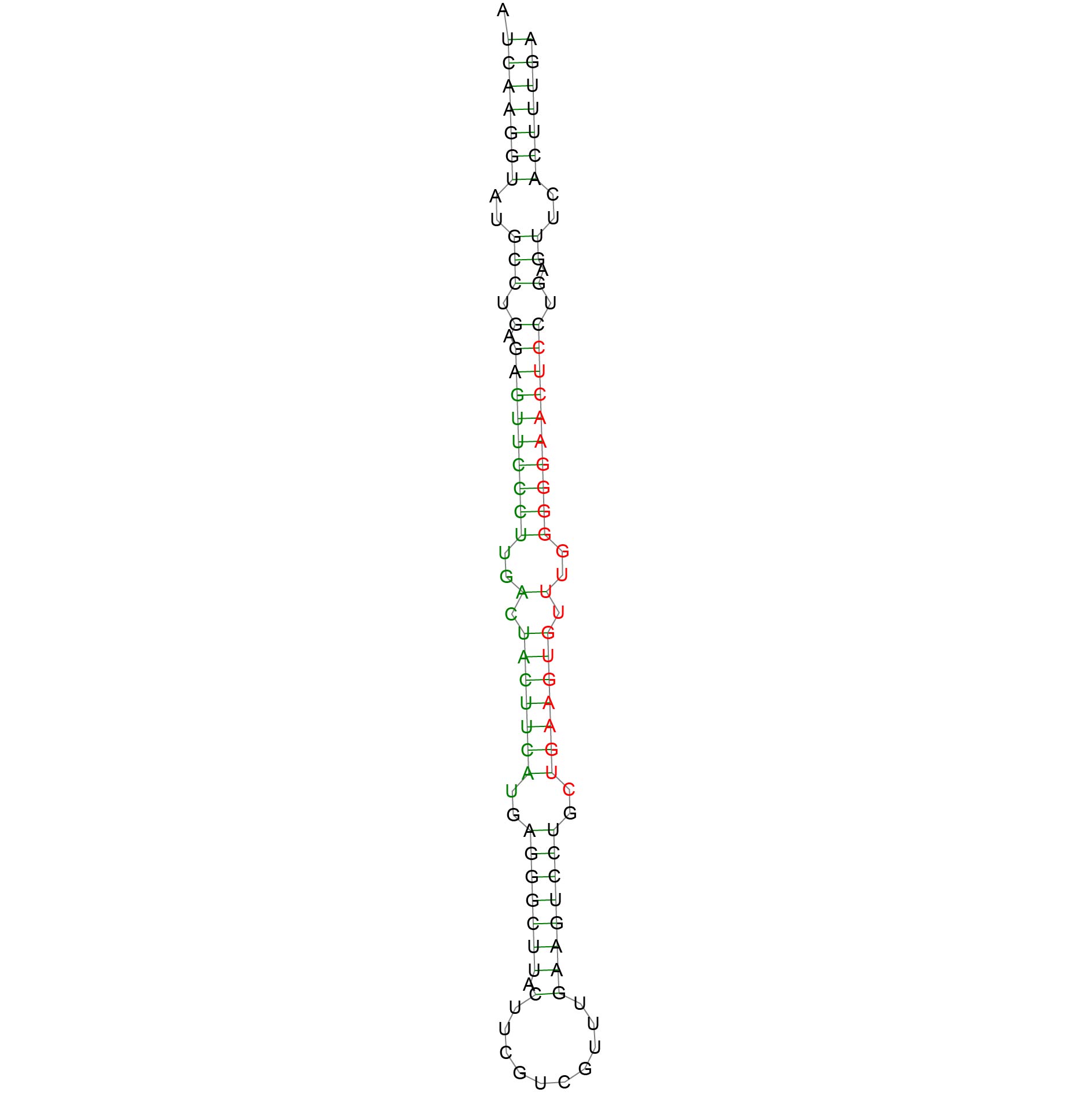

Supplement: Dataset S1 — Full list of hairpin structures in conserved miRNAs. (ZIP) [file pone.0064238.s001.zip › can-miR395e.jpg]

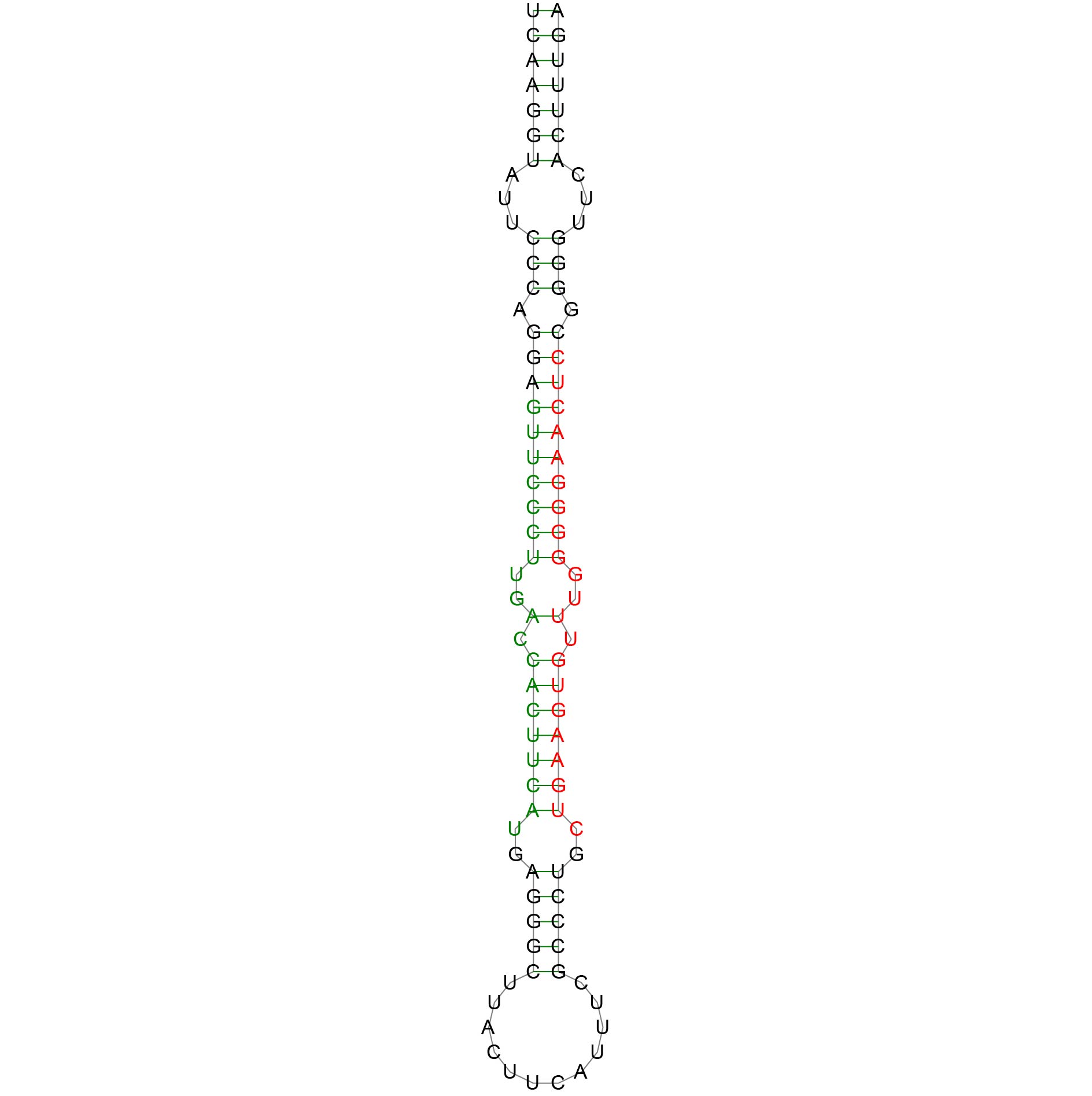

Supplement: Dataset S1 — Full list of hairpin structures in conserved miRNAs. (ZIP) [file pone.0064238.s001.zip › can-miR395f.jpg]

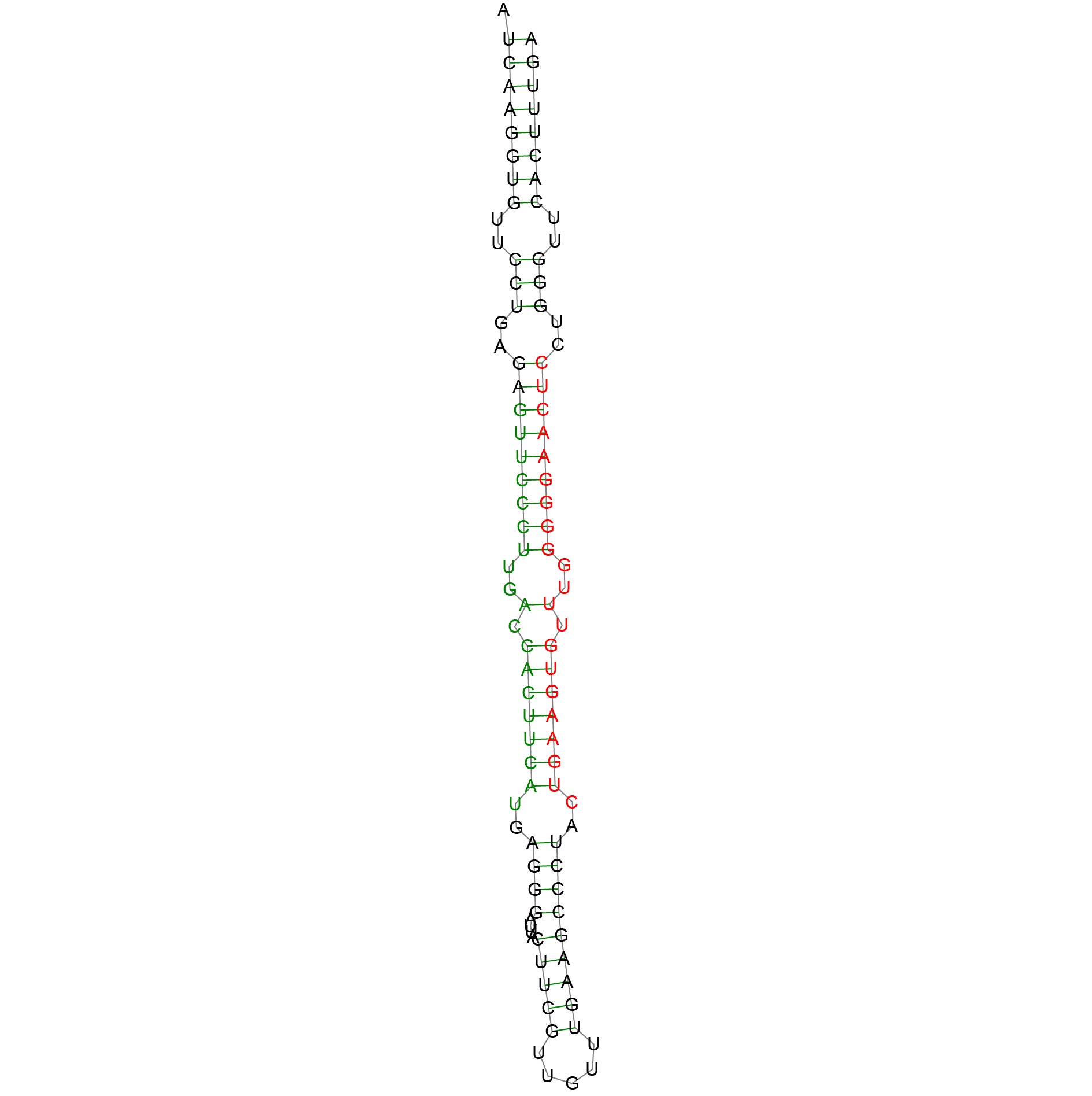

Supplement: Dataset S1 — Full list of hairpin structures in conserved miRNAs. (ZIP) [file pone.0064238.s001.zip › can-miR395g.jpg]

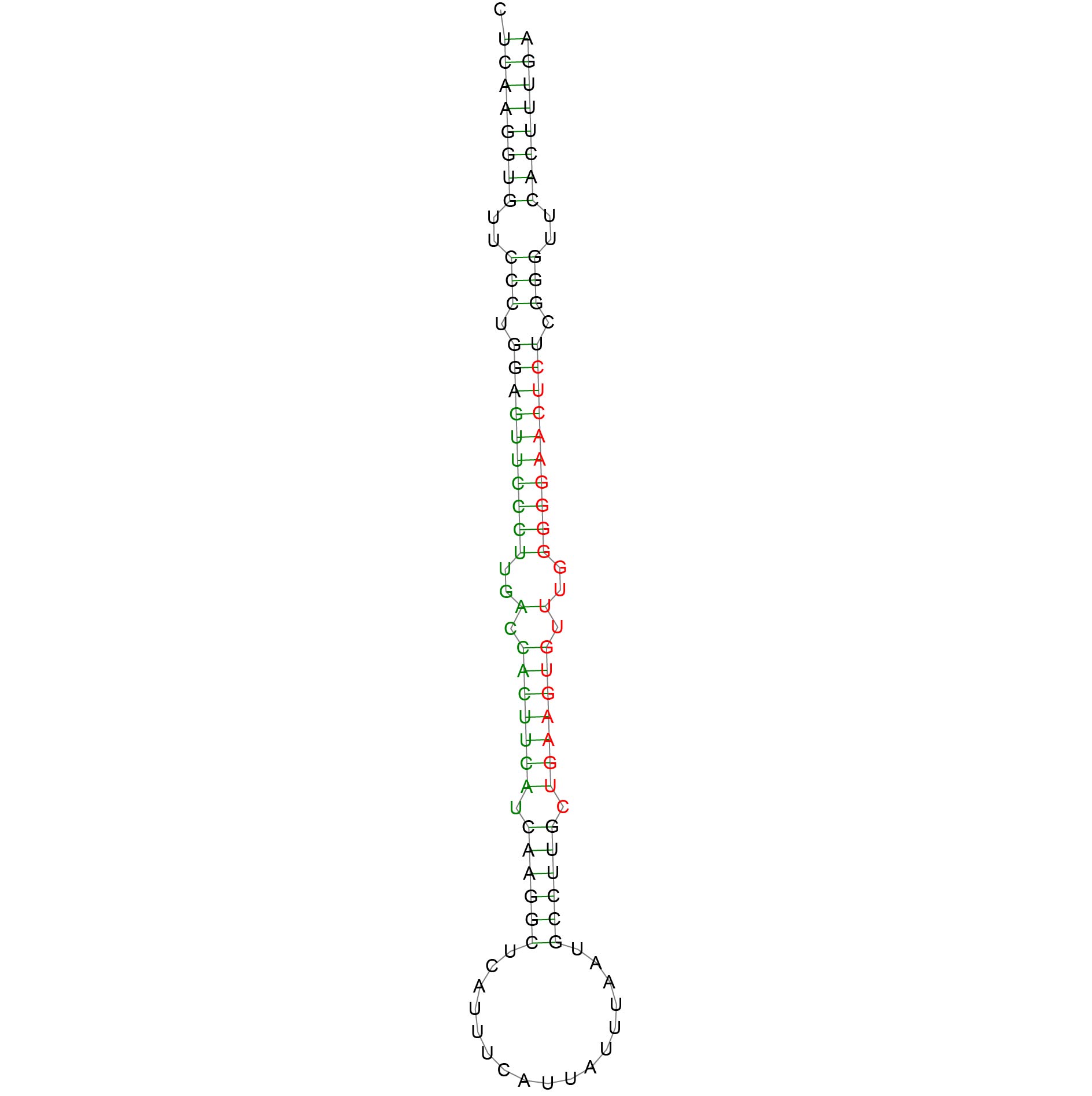

Supplement: Dataset S1 — Full list of hairpin structures in conserved miRNAs. (ZIP) [file pone.0064238.s001.zip › can-miR395h.jpg]

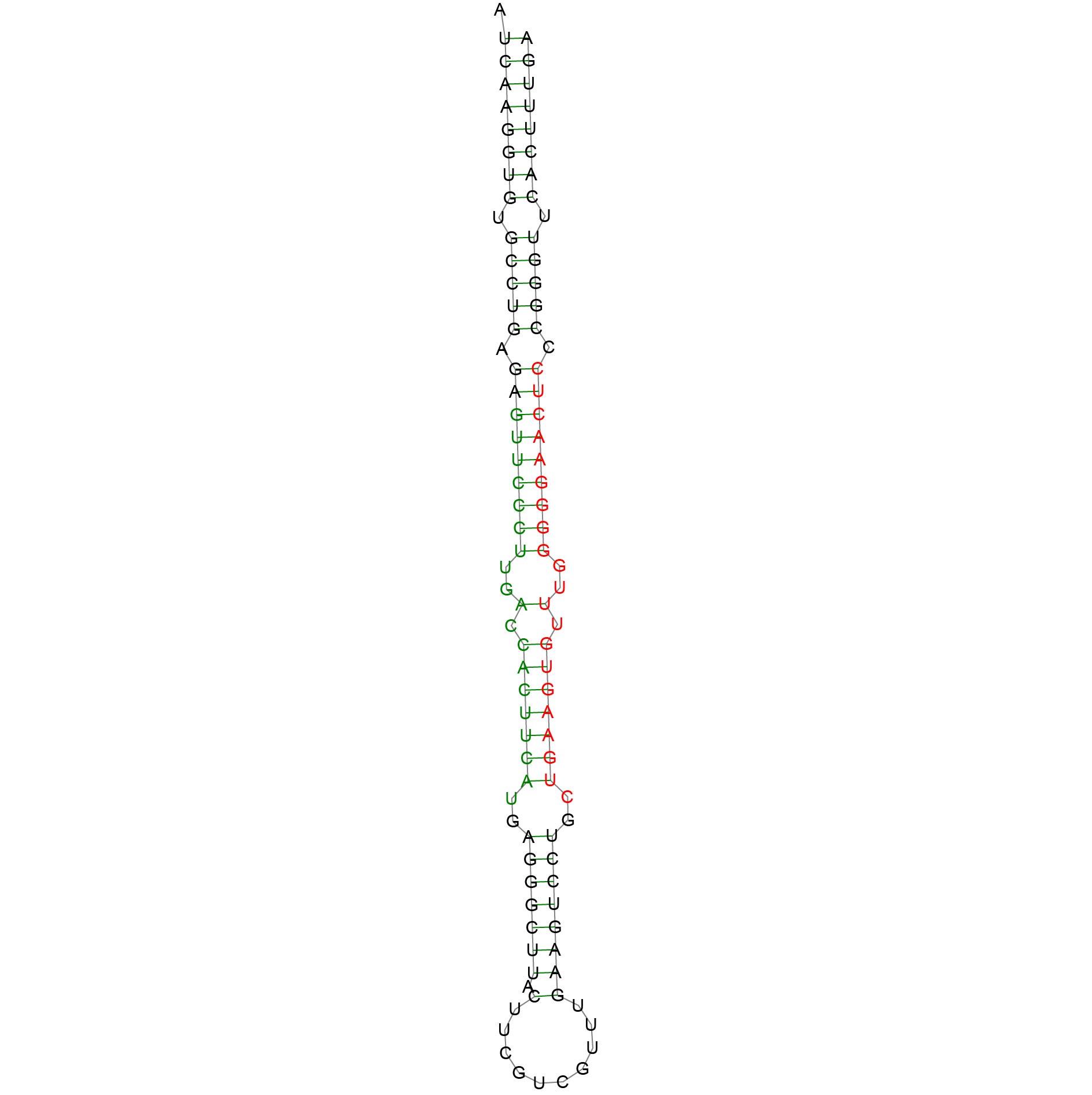

Supplement: Dataset S1 — Full list of hairpin structures in conserved miRNAs. (ZIP) [file pone.0064238.s001.zip › can-miR395i.jpg]

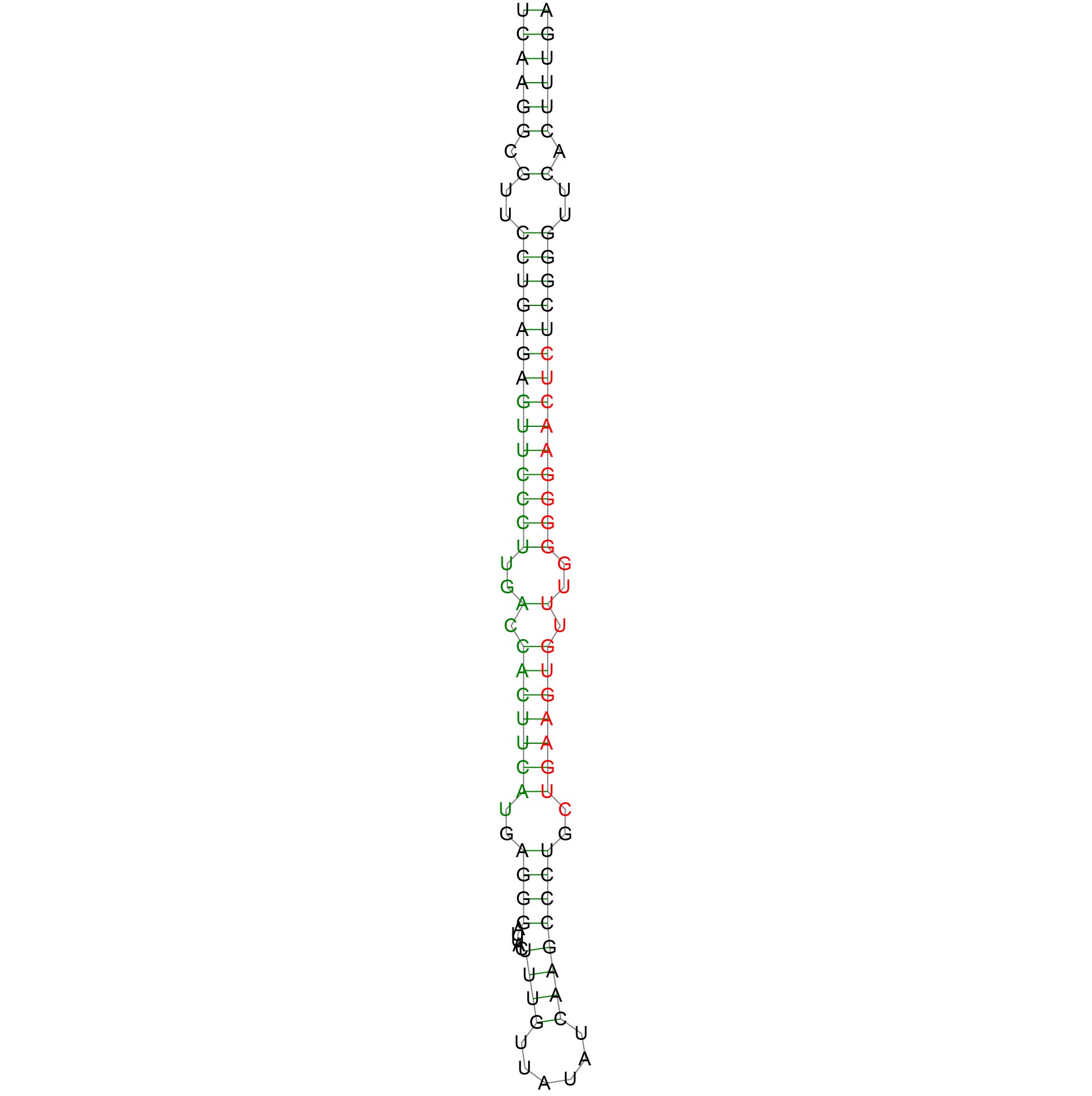

Supplement: Dataset S1 — Full list of hairpin structures in conserved miRNAs. (ZIP) [file pone.0064238.s001.zip › can-miR395j.jpg]

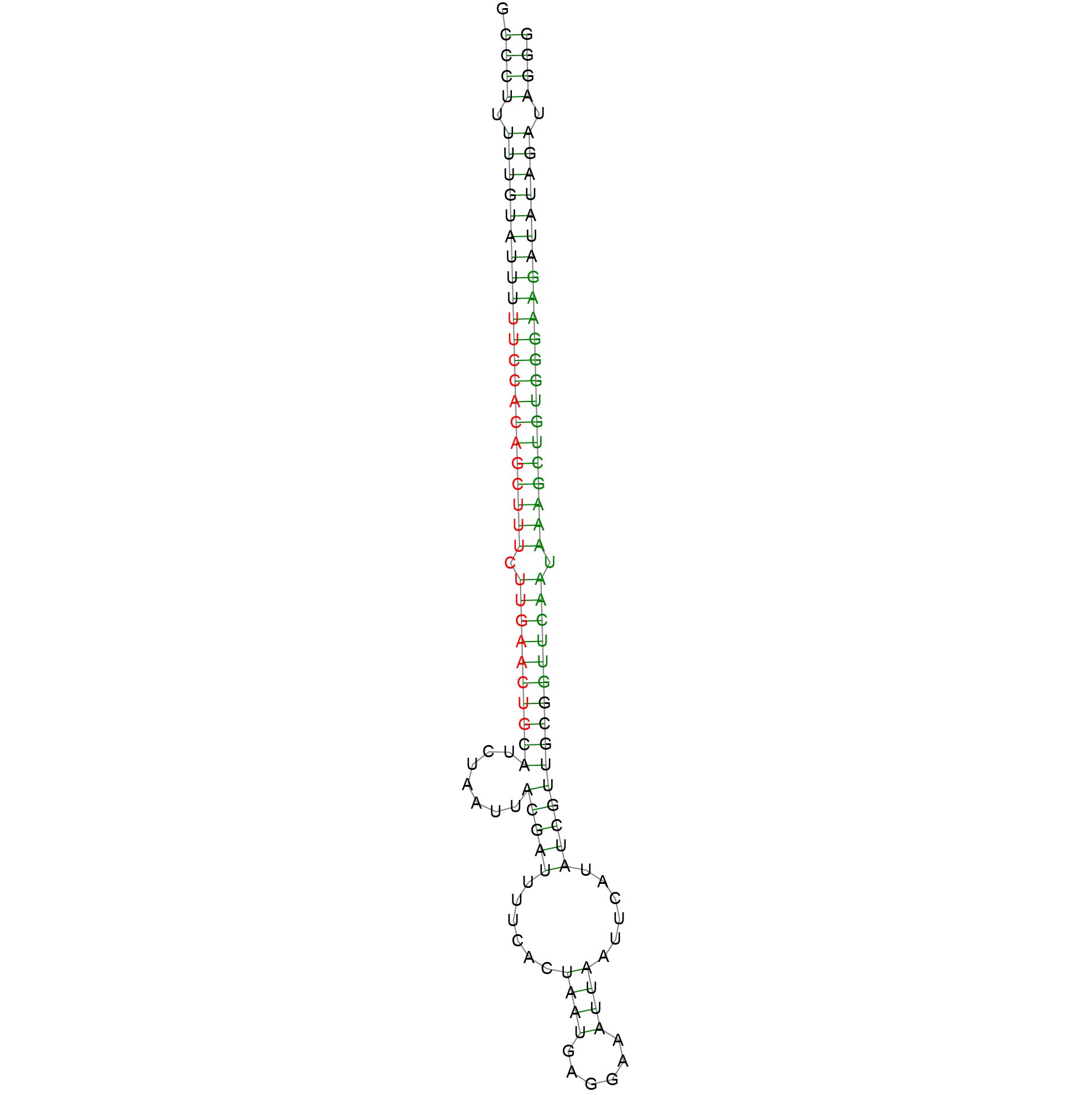

Supplement: Dataset S1 — Full list of hairpin structures in conserved miRNAs. (ZIP) [file pone.0064238.s001.zip › can-miR396a.jpg]

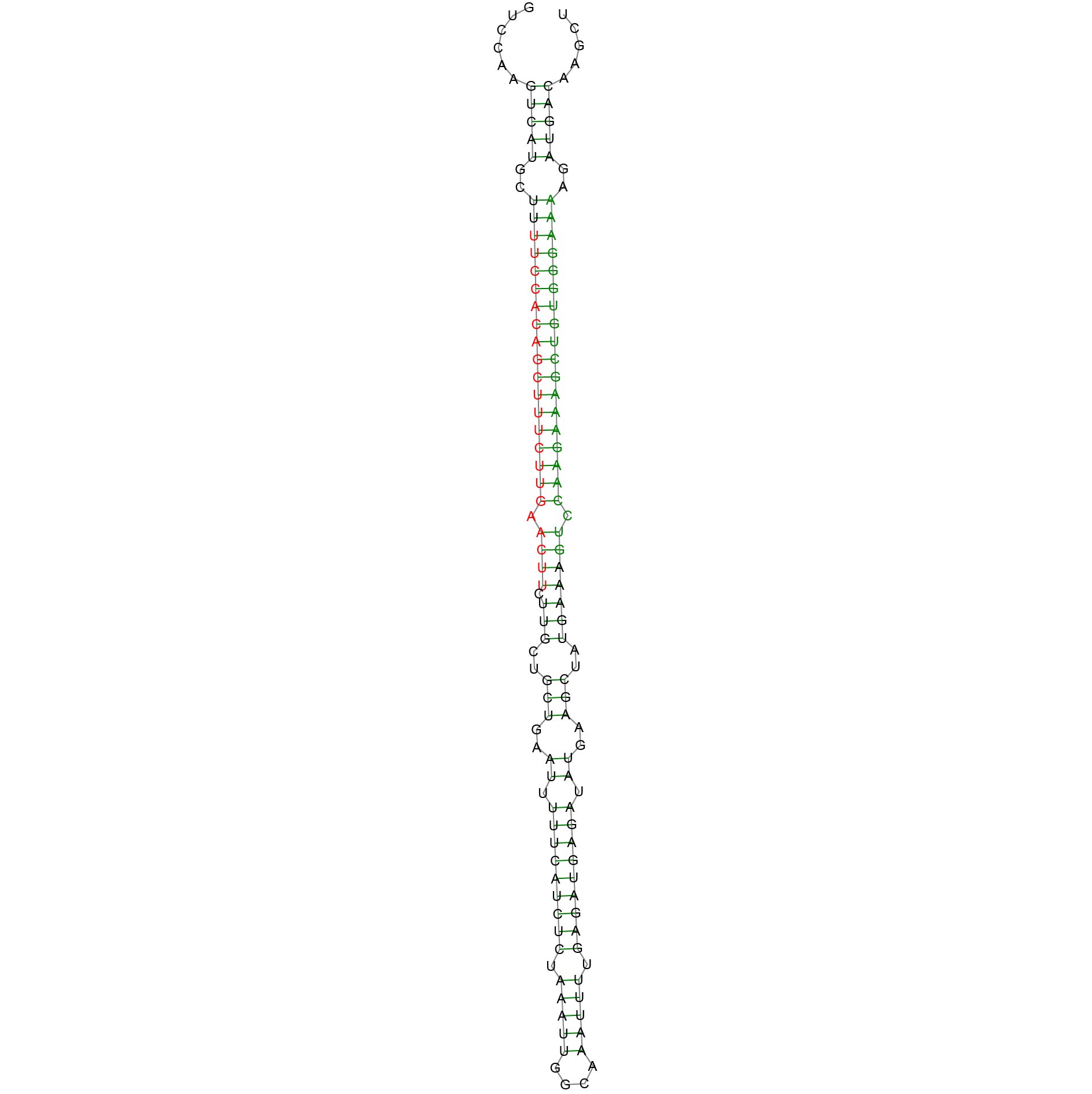

Supplement: Dataset S1 — Full list of hairpin structures in conserved miRNAs. (ZIP) [file pone.0064238.s001.zip › can-miR396b.jpg]

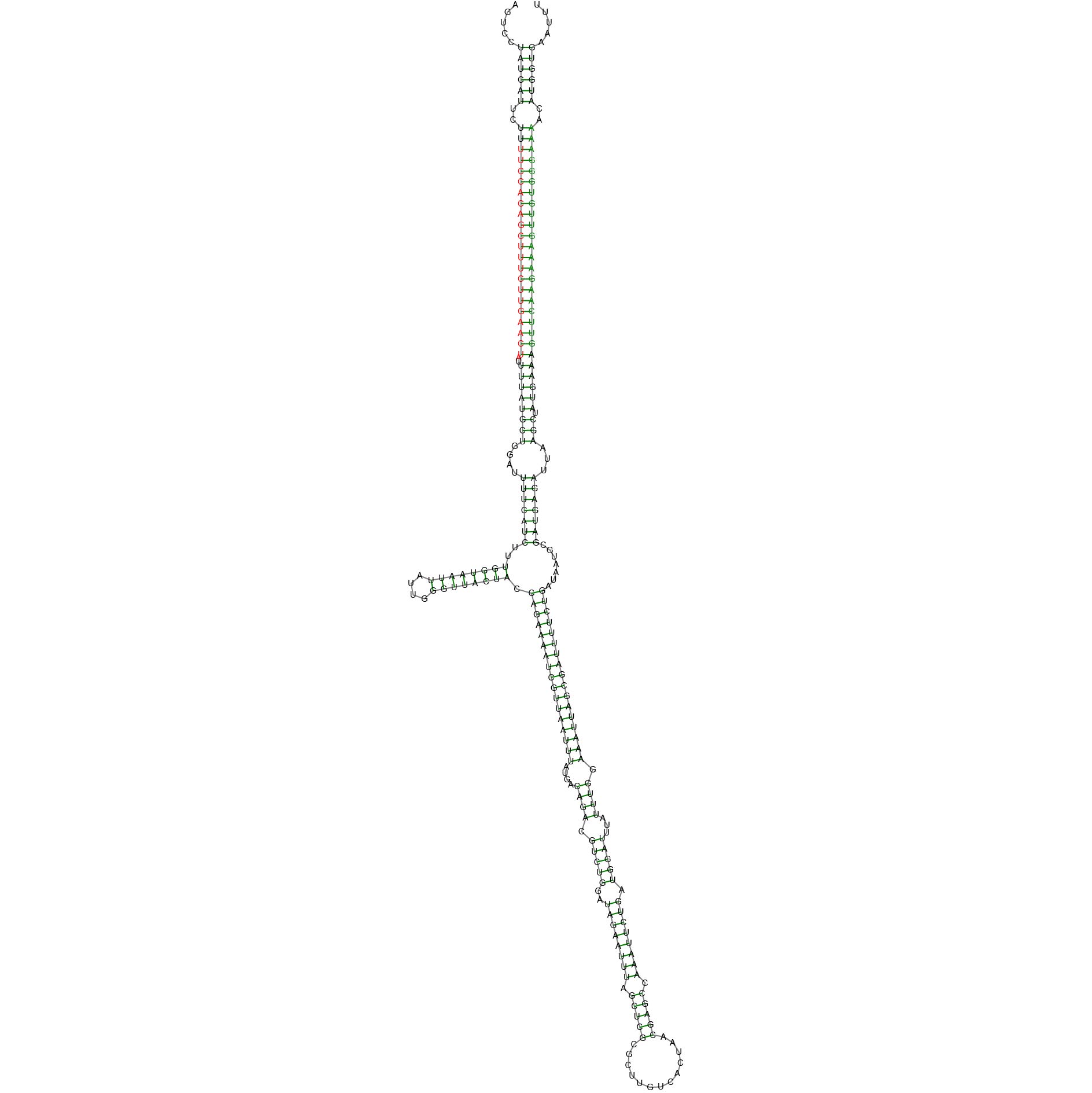

Supplement: Dataset S1 — Full list of hairpin structures in conserved miRNAs. (ZIP) [file pone.0064238.s001.zip › can-miR396c.jpg]

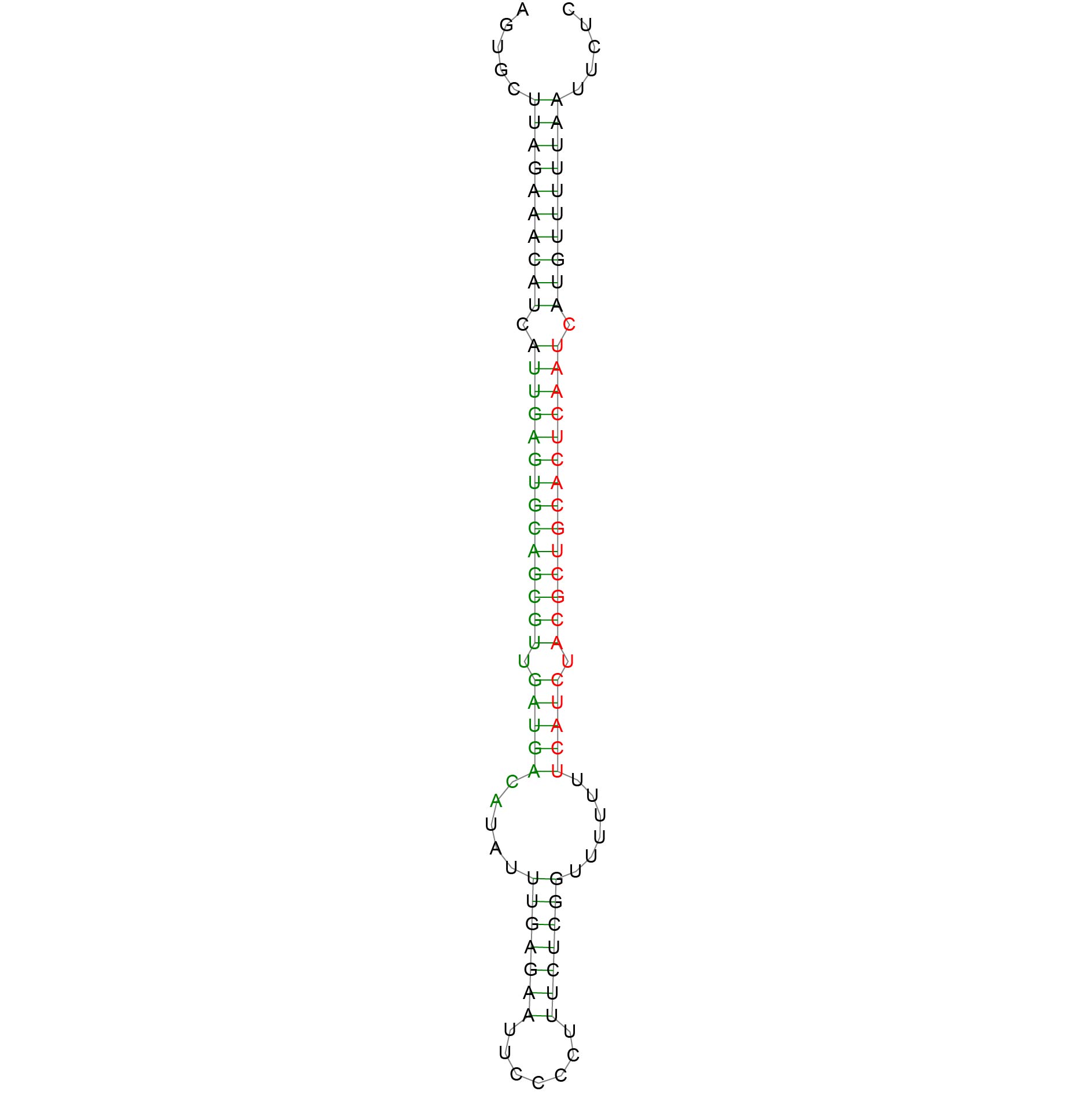

Supplement: Dataset S1 — Full list of hairpin structures in conserved miRNAs. (ZIP) [file pone.0064238.s001.zip › can-miR397a.jpg]

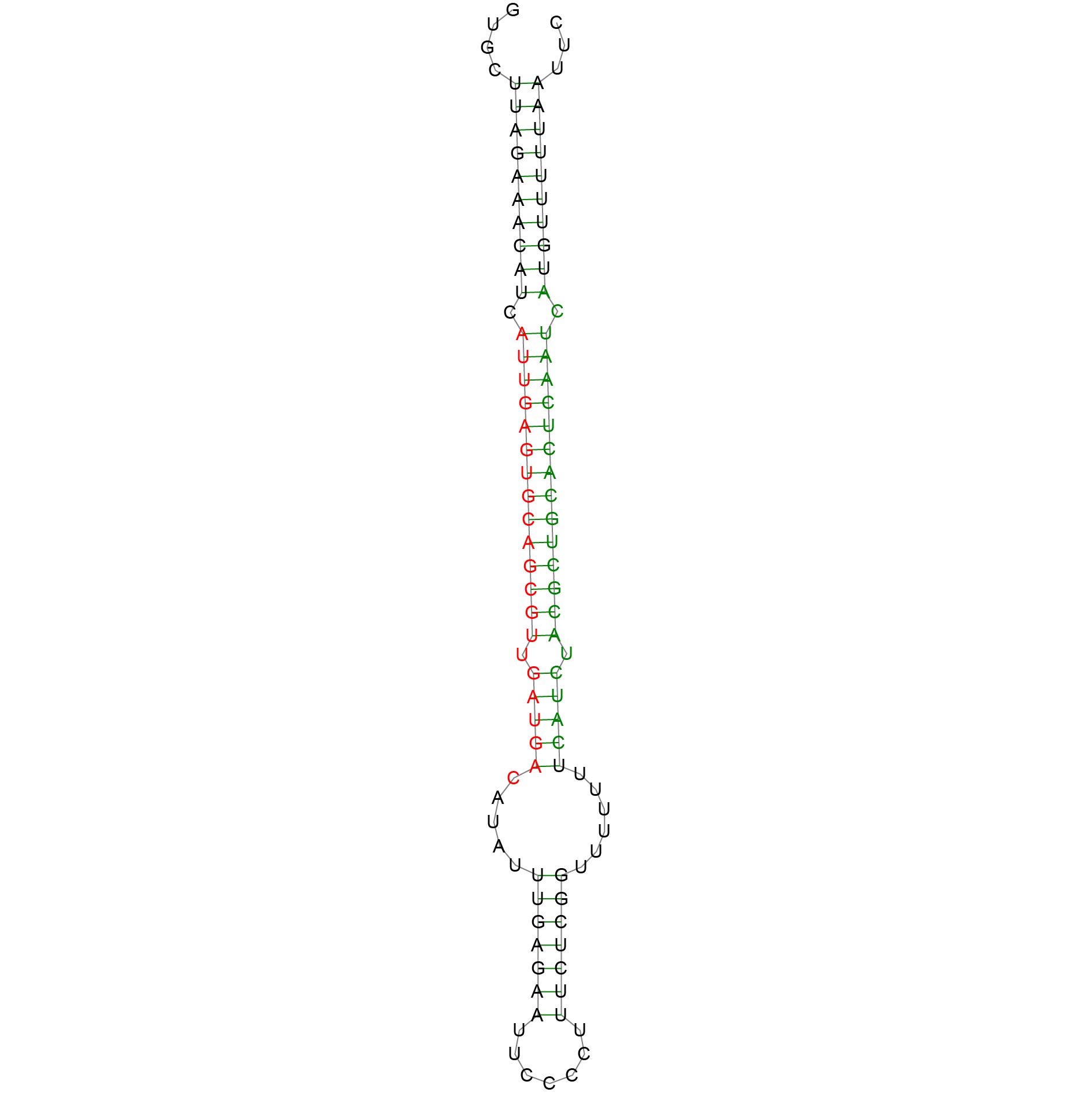

Supplement: Dataset S1 — Full list of hairpin structures in conserved miRNAs. (ZIP) [file pone.0064238.s001.zip › can-miR397b.jpg]
